# Harnessing Sulfur(VI) Fluoride Exchange Click Chemistry and Photocatalysis for Deaminative Benzylic Arylation

**DOI:** 10.1021/acscatal.3c01981

**Published:** 2023-05-15

**Authors:** Deepta Chattapadhyay, Akin Aydogan, Katarzyna Doktor, Arunava Maity, Jiun Wei Wu, Quentin Michaudel

**Affiliations:** Department of Chemistry, Texas A&M University, College Station, Texas 77843, United States

**Keywords:** SuFEx, photocatalysis, diazene, nitrogen deletion, nickel-catalyzed cross-coupling

## Abstract

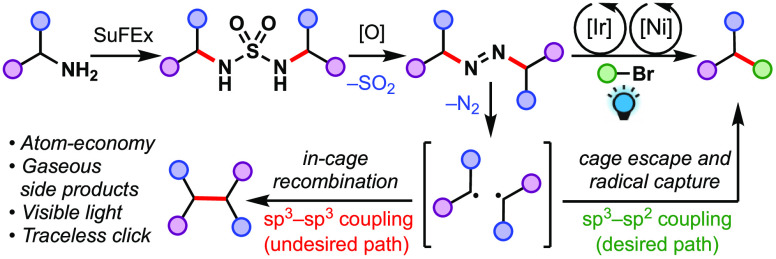

While being among the most common functional handles present in organic molecules, amines are a widely underutilized linchpin for C–C bond formation. To facilitate C–N bond cleavage, large activating groups are typically used but result in the generation of stoichiometric amounts of organic waste. Herein, we report an atom-economical activation of benzylic primary amines relying on the sulfur(VI) fluoride exchange (SuFEx) click chemistry and the *aza*-Ramberg–Bäcklund reaction. This two-step sequence allows the high-yielding generation of 1,2-dialkyldiazenes from primary amines via loss of SO_2_. Excitation of the diazenes with blue light and an Ir photocatalyst afford radical pairs upon expulsion of N_2_, which can be coaxed into the formation of C(sp^3^)–C(sp^2^) bonds upon diffusion and capture by a Ni catalyst. This arylative strategy relying on a traceless click approach was harnessed in a variety of examples, and its mechanism was investigated.

Primary amines are one of the most ubiquitous functional groups found across families of natural products—from proteins and peptides to alkaloids and amino sugars—and in synthetic pharmaceuticals.^1^ Despite their undisputed prevalence, aliphatic amines are not nearly as popular as functional handles for scaffold diversification as other functional groups such as alkyl halides, carboxylic acids, and alcohols. Amines are commonly engaged in amidation and Buchwald–Hartwig reactions, but unstrained C–N bonds are rarely seen as practical precursors for C–C cross-coupling reactions because of the inherent stability of this bond.^2−5^ Heterolytic displacement of amines is disfavored by the poor leaving group ability of this basic functional group,^6^ and synthetically useful homolytic cleavages remain elusive. Various strategies have therefore been explored to favor C–N activation via prefunctionalization (Scheme 1A). Early examples include diazonium salts,^7,8^ but these highly reactive intermediates prone to elimination typically lead to undesired side reactions and poor functional group tolerance. An interrupted diazotization strategy was recently leveraged to functionalize α-primary benzylamines with boronic acids.^9^ Benzylic ammonium salts have been coupled with boronic acids;^10^ however, the need for strongly alkylating agents potentially limits the scope of suitable substrates. Katritzky-type pyridinium salts have been employed in several photocatalytic and metal-catalyzed cross-coupling reactions, affording a powerful platform to activate primary amines bearing moderate steric hindrance.^11−23^ Electron-rich trimethoxyphenyl imine provided an alternative approach, complementary in scope (α-tertiary primary amines) to the pyridylation activation.^24,25^ Finally, a nitrogen-deletion strategy promoted by anomeric amides in the presence of secondary amines has been reported^26^ and subsequently expanded to deaminative functionalizations of primary amines,^27,28^ albeit not in C–C cross-couplings. A common trade-off for these elegant strategies is the use of large activating groups, which lowers the activation barrier for C–N bond cleavage but creates large amounts of waste.

**Scheme 1:**
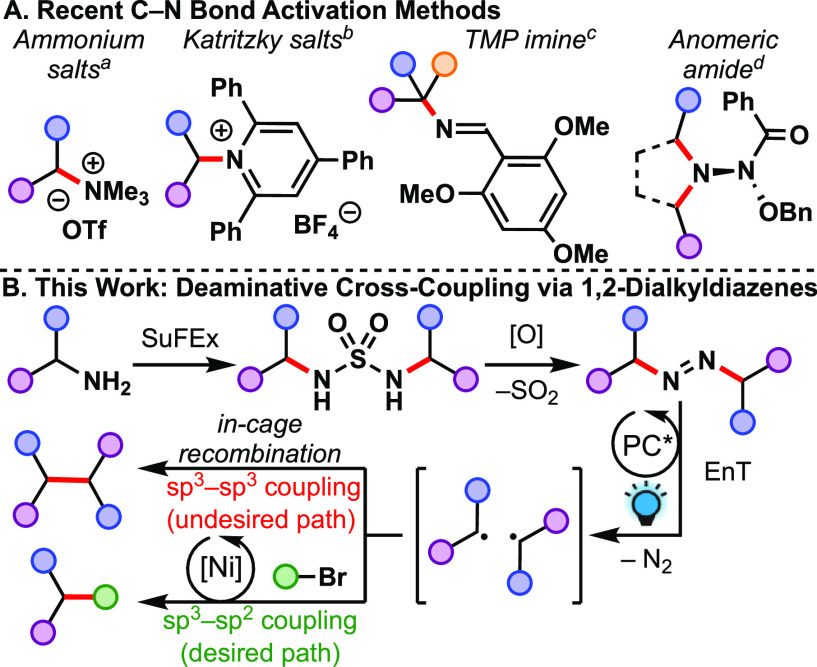
Amines as Precursors for C–C Bond Formation Reference (10). References (11−23). References (24 and 25). References (26−28).

We describe herein a mild and atom-economical deaminative arylation relying on the formation of *N*,*N′*-disubstituted sulfamides using sulfur(VI) fluoride exchange (SuFEx) click chemistry, followed by oxidative formation of a 1,2-diazene concomitant with loss of SO_2_ and Ni-catalyzed arylation (Scheme 1B). We hypothesized that fragmentation of the diazene induced by energy transfer would offer a manifold for sp^3^–sp^2^ coupling via radical capture of a Ni catalyst if radical recombination can be discouraged. The loss of small, gaseous byproducts would minimize waste and facilitate purification in contrast to the large activating groups traditionally used. While literature precedent on SuFEx^29−31^ and diazene chemistry^32^ suggests that this sequence would provide a general platform for C–N activation, the desired path is unprecedented and hinges upon optimization of elementary steps in the process to ensure that (1) cage escape outcompetes radical recombination, (2) conditions for the radical generation and Ni catalysis are orthogonal, and (3) the rate of fragmentation closely matches the rate of arylation to avoid accumulation of radicals. Overall, the development of this process is expected to provide both a traceless click method for skeleton diversification and fundamental insights into the reactivity of diazene intermediates.

Our investigation began with the search for efficient conditions to produce diazenes from primary amines and optimize the generation of the radical pair (Scheme 2). (*S*)-**1a** was selected as a test substrate because of the relative stability of benzylic radicals. Using an enantiopure amine helped streamline the optimization of the first two steps by preventing the formation of diastereomeric mixtures. SuFEx offers milder reaction conditions leading to high functional group tolerance compared to oxidative reagents such as SO_2_Cl_2_. Our previously developed two-step synthesis of sulfamides^33^ was telescoped to offer a one-pot synthesis of symmetrical compounds. Treatment of (*S*)-**1a** with imidazolium reagent **2**(34) followed by addition of DBU and heating to 50 °C delivered *N*,*N′*-disubstituted sulfamide (*S,S*)-**3a** in 90% yield. Chlorination of *N*,*N′*-dialkyl sulfamides in the presence of a base is known to promote the formation of diazenes via the intermediacy of a thiadiaziridine-1,1-dioxide and subsequent loss of SO_2_ in a mechanism reminiscent of the venerable Ramberg–Bäcklund reaction.^35−37^ This transformation has been mostly restricted to mechanistic studies on diazene fragmentation with the exception of its implementation into elegant syntheses of several cyclotryptamine alkaloids.^38−40^ Pleasingly, a combination of inexpensive TCCA and DBU delivered diazenes in high yields without epimerization (90% for (*S,S*)-**4a**).

**Scheme 2:**
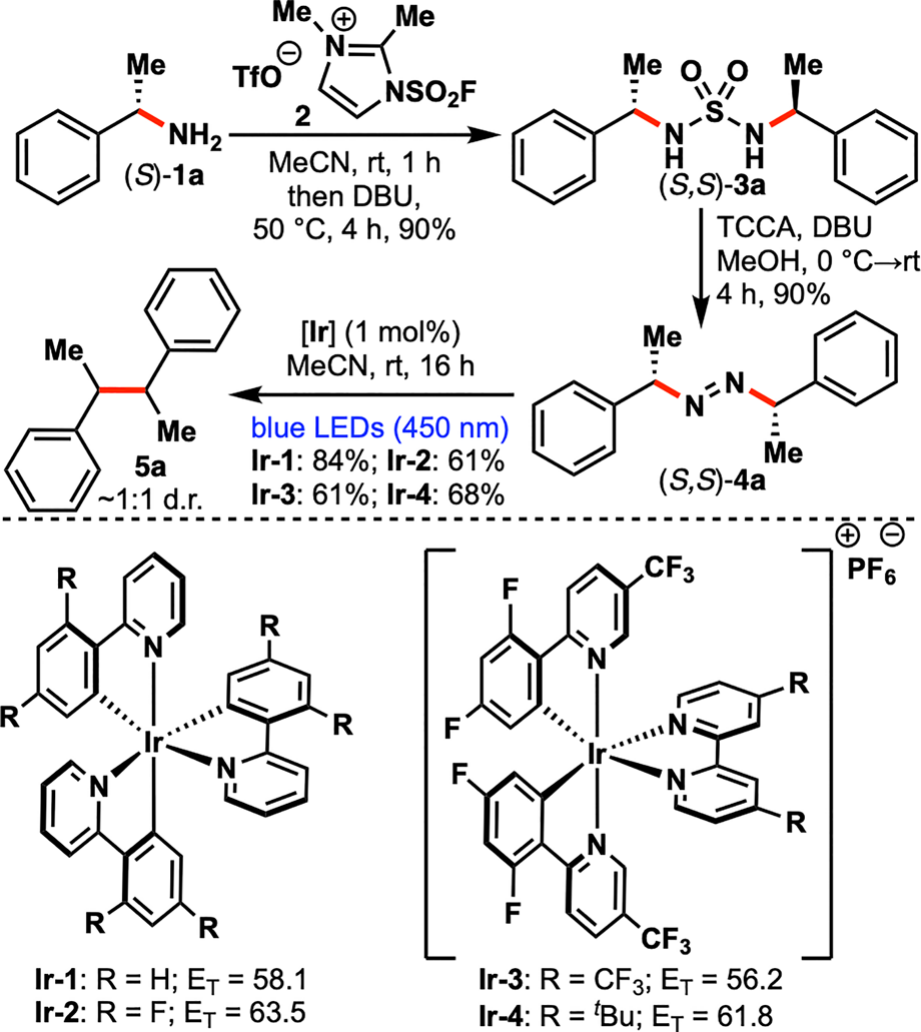
Synthesis and Photocatalyzed Fragmentation of Diazene (*S,S*)-**4a** Abbreviations: TCCA, trichloroisocyanuric acid; DBU, 1,8-diazabicyclo[5.4.0]undec-7-ene. The triplet excited state energy (*E*_T_) is given in kcal mol^–1^.^43,44^

Fragmentation of diazenes induced with high-energy UV light proceeds through the singlet excited state on a subpicosecond time scale, which favors fast recombination of the geminate radical pairs within the solvent cage.^41,42^ Aiming to divert reactivity in the presence of a Ni catalyst and favor capture of the carbon-centered radical instead, we decided to explore a triplet sensitization process using a photocatalyst and visible light. Indirect excitation was hypothesized to provide several advantages. First, generation of a geminate triplet pair that must undergo intersystem crossing prior to recombination would potentially increase the likelihood of cage escape.^32,41^ Second, visible light provides a safer alternative to UV irradiation that also minimizes undesired side reactions. Because of their known compatibility in tandem catalysis involving Ni, and their high triplet excited state energy,^43,44^ Ir photocatalysts **Ir-1**–**Ir-4** were screened for the fragmentation of (*S,S*)-**4a** (Scheme 2). MeCN was selected for its low viscosity (0.37 mPa s at 25 °C),^45^ a parameter favoring diffusion of radical pairs apart to form free radicals.^46^ Gratifyingly, all four Ir catalysts provided homocoupled product **5a** in 61–84% yield under blue light (450 nm). In all cases, racemization was observed and a mixture of meso and non-meso compounds **5a** was isolated in ∼1:1 dr. This loss of stereochemistry is similar to that previously observed in the thermal degradation of (*S,S*)-**4a** and is consistent with radical diffusion.^47^

With the conditions in hand to effect diazene fragmentation with visible light, we turned our attention to the optimization of the Ni-catalyzed arylation process using (*S,S*)-**4a** and aryl bromide **6**. Extensive screening of the reaction conditions (Tables S1–S6) revealed that NiBr_2_·(**L1**), **Ir-3**, and Zn powder in a 95:5 mixture of MeCN and DMAc led to the formation of **7a** in 60% yield, along with 27% of homodimer **5a** (Table 1, entry 1). Importantly, the yield was calculated based on two carbon-centered radicals generated for each equivalent of diazene (*S,S*)-**4a**. Therefore, only a slight effective excess of aryl bromides to the radical species (1.5:1) is necessary for efficient arylation. Replacement of NiBr_2_·(**L1**) by Ni(acac)_2_ or Ni(TMHD)_2_ resulted in a small decrease of the yield of **7a** (entries 2 and 3). Switching to bisoxazoline ligand **L2** was accompanied by a stark decrease in the yield of **7a** (entry 4). Interestingly, **Ir-2** and **Ir-4** also provided reduced efficiency for radical capture concomitant with a marked prevalence for radical recombination in the case of **Ir-4** (16% vs 61%, entries 5 and 6). A small amount of DMAc (5%) was found to be crucial to ensure efficient arylation (entry 7). Other reducing agents such as Mn did not perform as well as Zn (entry 8). Addition of KH_2_PO_4_ delivered **7a** the highest yield (65%, entry 9). Finally, control reactions showed that only a trace amount of **5a** was formed without photocatalyst and neither **5a** nor **7a** were isolated without light (entries 10 and 11).

**Table 1:**
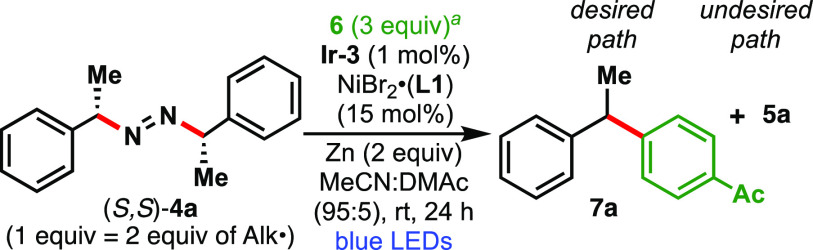
Arylation Optimization and Control Reactions^b^

entry	deviation from the initial conditions	yield^c^ of **7a** (%)	yield^c^of **5a** (%)
1	none	60	27
2	Ni(acac)_2_ instead of NiBr_2_·(**L1**)	50	13
3	Ni(TMHD)_2_ instead of NiBr_2_·(**L1**)	57	16
4	NiBr_2_·(**L2**) instead of NiBr_2_·(**L1**)	29	34
5	**Ir-2** instead of Ir-3	41	25
6	**Ir-4** instead of **Ir-3**	16	61
7	MeCN as solvent	31	22
8	Mn instead of Zn	45	27
**9**	**KH**_**2**_**PO**_**4**_ (2 equiv) as additive	**65**	**28**
10	no photocatalyst	—	4
11	no light	—	—

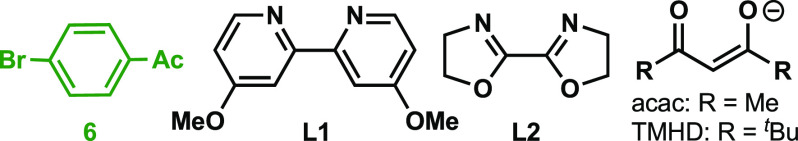

a Ratio of Alk· to **6** is 1:1.5.

b Abbreviation: DMAc, dimethylacetamide.

c Yields were determined by ^1^H NMR using phenyltrimethylsilane as standard and calculated based on (*S*,*S*)-**4a** = 2 equiv of Alk·.

Exploration of the scope of this transformation revealed that electron-deficient aryl bromides provided arylated products in high yields (Table 2). Notably, electrophilic groups including ketones (**7b**–**e**), aldehyde (**7f**), ester (**7g**), lactone (**7h**), and nitriles (**7i**–**k**) were incorporated efficiently, which renders this arylation method complementary to Ni-catalyzed cross-coupling relying on Grignard or organozinc nucleophiles.^48−50^ Pinacol boronate **7j** and fluorinated derivative **7k** were isolated in good yields, which suggests that this method could be elaborated further with Suzuki coupling and S_N_Ar, two of the most popular reactions in the arsenal of medicinal chemists for the construction of therapeutic candidates.^51^ Sulfur(VI)-containing functional groups including sulfonamide (**7l**) and sulfone (**7m**) that are prevalent in pharmacophores^52^ and agrochemicals^53^ were also compatible with this catalysis. Consistent with other related transformations, aryl bromides with strongly electron-donating substituents gave poor yields (Table S7) but, interestingly, 1,1′-biphenyl compound **7o** was isolated in 42% yield. Finally, heteroaryl bromides are suitable as well, as shown by the synthesis of pyridine-containing product **7p**.

**Table 2:**
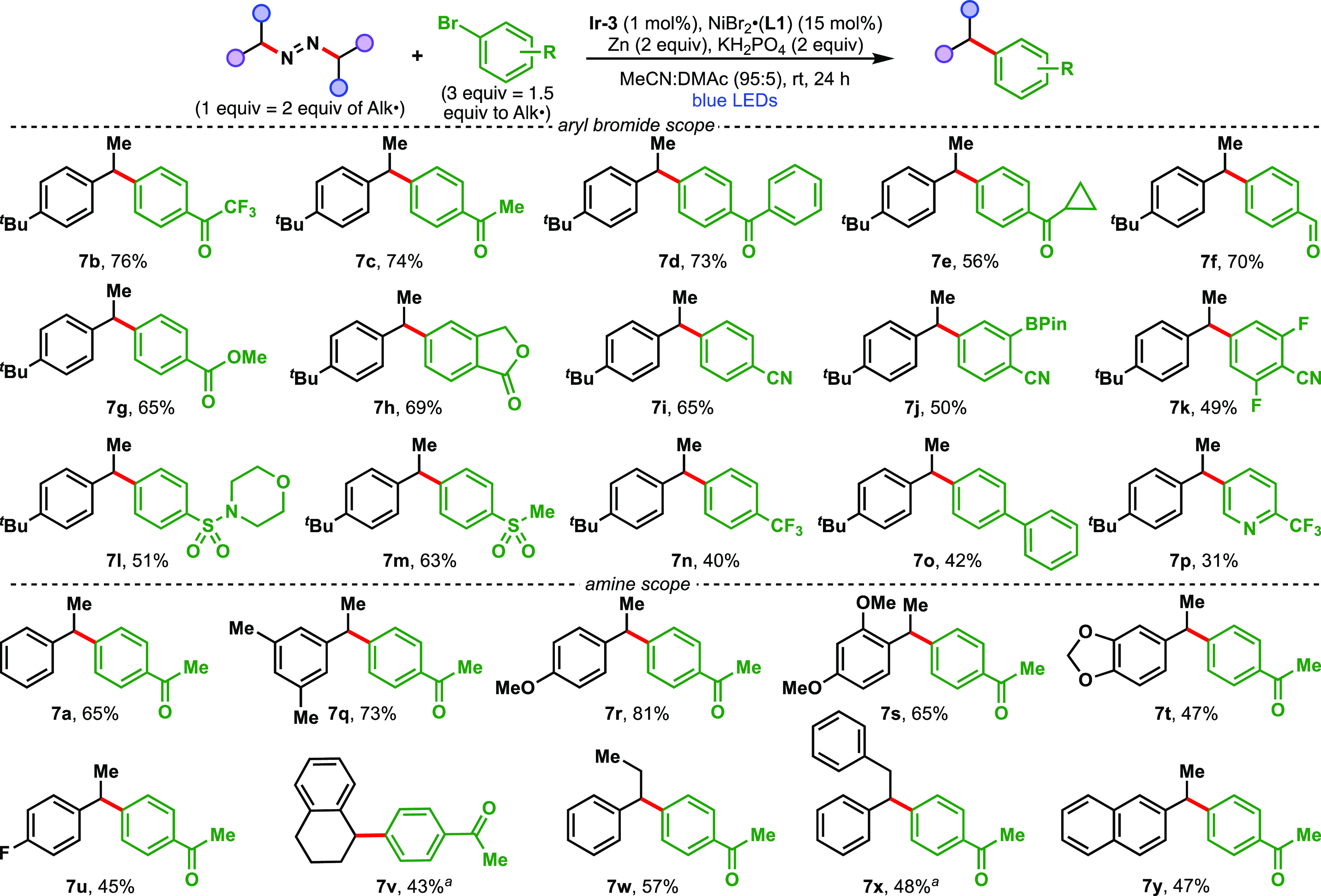
Scope of the Deaminative Ni-catalyzed Arylation^b^

a Ni(acac)_2_ was used in lieu of NiBr_2_·(**L1**).

b All reactions were run on a 0.13 mmol scale. Yields based on diazenes = 2 equiv of Alk·.

A variety of α-secondary benzylic amines were carried through the C–N activation sequence. Notably, inconsequential mixtures of diastereomers were used for the cross-coupling step. Electron-donating substituents including alkyl (**7q**) and methoxy (**7r**,**s**) provided yields up to 81% compared to that of neutral **7a** (65%), while catechol derivative **7t** was produced in 47% yield. Interestingly, fluorinated product **7u** was isolated in 45% yield. Increasing the steric hindrance around the amine resulted in moderate yields (**7v**–**x**). Finally, naphthalene derivative **7y** was isolated in 47% yield.

The photoluminescence of **Ir-1**–**Ir-4** with different concentrations of diazene (*S*,*S*)-**4a** was measured to gain a better understanding of the reaction mechanism. With all catalysts, the photoluminescence quenching by (*S*,*S*)-**4a** followed the Stern–Volmer equation (1), which, combined with literature precedent,^32^ supports the postulated photosensitization step (Figure 1).^54^
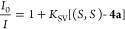
1

**Figure 1:**
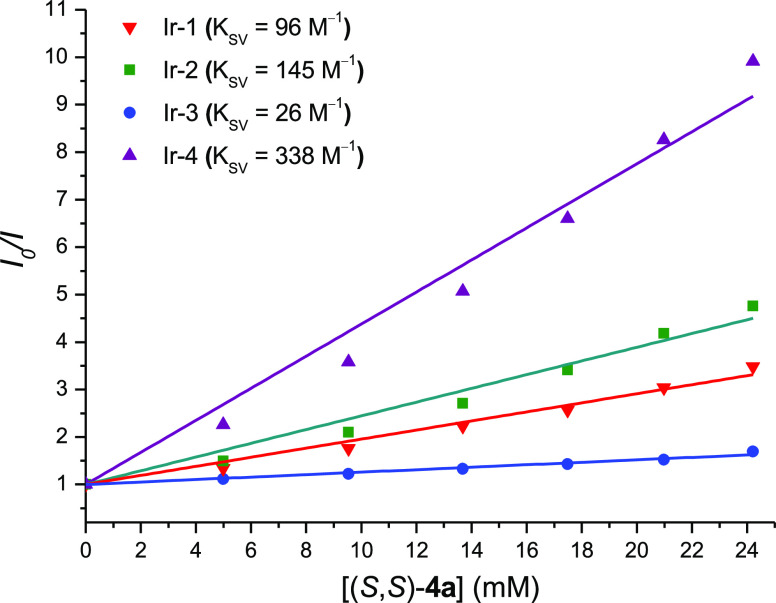
Stern–Volmer analysis of the photoluminescence quenching of **Ir-1**–**Ir-4** with diazene (*S*,*S*)-**4a**.

Importantly, the best catalyst for the arylation (**Ir-3**) corresponds to the lowest slope coefficient (*K*_SV_ = 26 M^–1^), which is in line with our hypothesis that a low concentration of radical species would increase the yield of arylation. The generation of carbon-centered radical species was supported by the isolation of adduct **8** in the presence of TEMPO (2 equiv, Scheme 3A) and of unsaturated product **7z′** following cyclopropane ring opening during the coupling of diazene **4c** and aryl bromide **6** (Scheme 3B). Finally, the stoichiometric reaction between Ni(II) complex **Ni**_**ox**_**·(L1)** and diazene **4b** without Zn powder delivered **7n** in a yield almost identical with that under catalytic conditions (Scheme 3C). A postulated mechanism based on these results is depicted in Scheme 3D. Putatively, photosensitized fragmentation of the diazene with **Ir-3** leads to a carbon-centered radical pair, which, upon diffusion, is captured by the Ni(II) complex^55^ arising from oxidative addition of the aryl bromide and Ni(0).^56^ Reductive elimination then delivers the cross-coupled product and single-electron reduction from Zn turns over the catalyst. Additional studies are required to obtain a more complete mechanistic picture, including a potential disproportionation/comproportionation step,^57−61^ off-cycle intermediates,^62,63^ or sensitization of Ni species.^48,64−66^

**Scheme 3:**
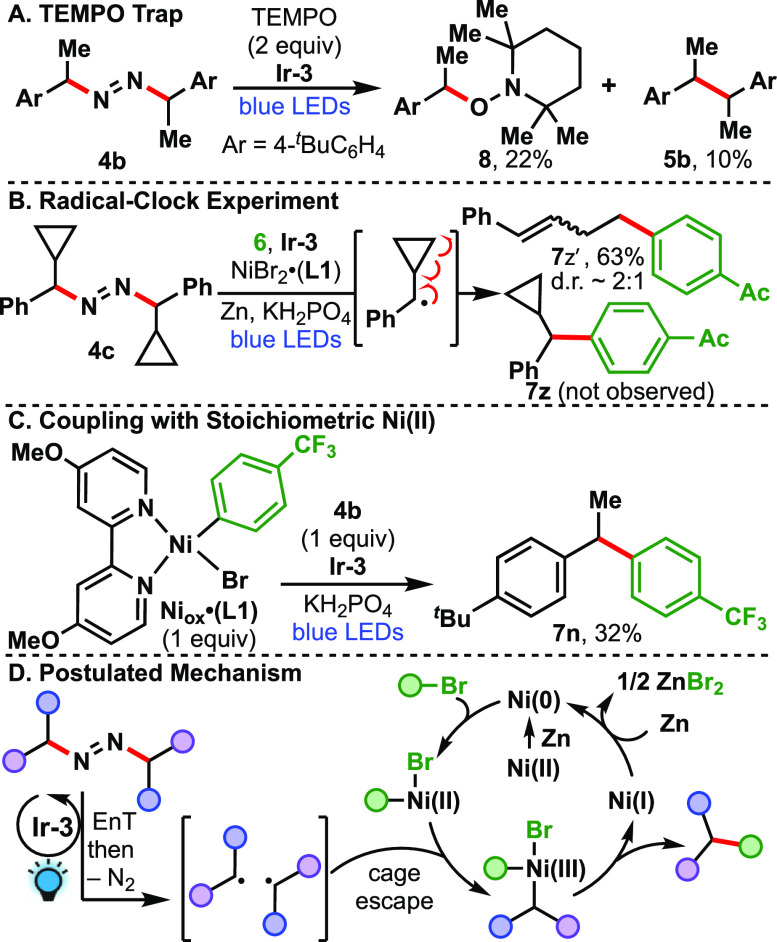
Mechanistic Investigation

In summary, we have developed a mild and atom-economical C–N activation strategy relying on SuFEx click chemistry combined with the *aza*-Ramberg–Bäcklund reaction. This sequence affords an efficient access to diazene molecules, which can be fragmented under blue light with a photocatalyst to form a radical pair. In the presence of a catalytic amount of NiBr_2_·(**L1**), an aryl bromide, and Zn, the carbon-centered radical can be directed toward the formation of the nondimeric C(sp^3^)–C(sp^2^) bond-forming pathway rather than undergo recombination. In contrast to known deaminative arylations employing a photocatalyst in tandem with a Ni catalyst, this reaction is postulated to follow a neutral redox cycle. Substrate activation via sensitization^67^ rather than single-electron transfer remains uncommon in light-mediated dual catalysis.^68^ This method therefore offers a unique manifold for reaction development, as well as a practical approach for the diversification of complex scaffolds that makes use of an underemployed type of functional handle.

## Supporting Information Available

Detailed experimental procedures, spectroscopic characterization, and additional supporting data (PDF)
