# Supplementary material for: Harnessing Sulfur(VI) Fluoride Exchange Click Chemistry and Photocatalysis for Deaminative Benzylic Arylation
Source: ACS Catal. 2023 May 15;13(11):7263–8. doi: 10.1021/acscatal.3c01981 (PMC10468006; doi:10.1021/acscatal.3c01981)

Supporting Information

Harnessing Sulfur(VI) Fluoride Exchange Click Chemistry and Photocatalysis for Deaminative Benzylic Arylation

Deepta Chattapadhyay,[‡1] Akin Aydogan,[‡1] Katarzyna Doktor,[1] Arunava Maity,[1] Jiun Wei Wu,[1] and Quentin Michaudel*[1,2]

[1]*Department of Chemistry, Texas A&M University, College Station, Texas 77843, United States*

[2]*Department of Materials Science and Engineering, Texas A&M University, College Station, Texas 77843, United States*

*Corresponding Author: quentin.michaudel@chem.tamu.edu

[‡]These authors contributed equally

General reagent information S2
General analytical information S2
Experimental procedures S3
Synthesis of sulfamides: **General Procedure A** S3
Synthesis and characterization of sulfamides S4
Synthesis of diazenes: **General Procedure B** S9
Synthesis and characterization of diazenes S10
Reaction optimization and control experiments S15
Diazene fragmentation and cross-coupling reaction: **General Procedure C** S18
Synthesis and characterization of cross-coupling products S19
Diazene fragmentation and homo-coupling reaction: **General Procedure D** S29
Synthesis and characterization of homocoupling products S30
Reactions with diazenes from α-primary amines and α-tertiary amines S31
Radical trapping with TEMPO S31
Radical clock experiment S32
Coupling with stoichiometric Ni(II) S33
Photoluminescence quenching experiments S33
References S35
NMR Spectra S37

General reagent information

All reactions were performed without any precaution for moisture and oxygen unless otherwise stated. Dry acetonitrile (MeCN) was obtained by passing the previously degassed solvents through activated alumina columns. Reagents were purchased at the highest commercial quality and used without further purification, unless otherwise stated. All starting materials were purchased at the highest commercial quality and used without further purification unless otherwise stated. 1-(fluorosulfonyl)-2,3-dimethyl-1H-imidazol-3-ium triflate (**2**),[1] amines (1-(1,3-benzodioxol-5-yl)ethanamine,[2] 1-(2,4-dimethoxyphenyl)ethan-1-amine,[2] 4-tert-Butylbenzylamine,[2] 1,2-Diphenylethylamine,[2] 1-(3,5-Dimethylphenyl)ethan-1-amine,[2] α-Ethylbenzylamine,[2] 1-(2-Naphthyl)ethylamine,[2] Cyclopropyl(phenyl)methanamine,[3] ligands (**L3**,[4] **L**4[5]), complex **Ni$_{ox}$•(L1)**,[6] and photocatalysts (**Ir-2**,[7] **Ir-3**[8]) were synthesized following known procedures. The reactions were heated using an oil bath unless otherwise stated. Yields refer to chromatographically and spectroscopically (^{1}H NMR) homogeneous material, unless otherwise stated. Reactions were monitored by thin layer chromatography (TLC) carried out on 250 μm SiliCycle SilicaPlate™ silica plates (F254), using UV light as the visualizing agent and an acidic solution of p-anisaldehyde and heat or ninhydrin and heat as developing agents. Flash silica gel chromatography was performed using SiliCycle SilicaFlash® Irregular Silica Gel (60 Å, particle size 40–63 μm). The photochemical reactions were carried out using Hepatochem EvoluChem™ PhotoRedOx Box Duo device and irradiated with two EvoluChem™ P303-30-1 LEDs (30 W, λ_{max} = 450 nm) with a cooling fan. All photochemical reactions were performed in borosilicate glass.

General analytical information

^{1}H Nuclear magnetic resonance (NMR) spectra were recorded on two Bruker Avance NEO 400 MHz and a Bruker Avance 500 MHz; ^{13}C spectra were recorded on a Bruker Avance 500 MHz and a Bruker Avance NEO 400 MHz; ^{19}F spectra were recorded using a Bruker Avance NEO 500 MHz instrument. All ^{1}H and ^{13}C spectra were calibrated using residual deuterated solvent as an internal reference ($CDCl_3$ @ 7.26 ppm ^{1}H NMR, 77.16 ppm ^{13}C NMR; DMSO-d_6 @ 2.50 ppm ^{1}H NMR, 39.52 ppm ^{13}C NMR). The following abbreviations were used to explain NMR peak multiplicities: s = singlet, d = doublet, t = triplet, q = quartet, m = multiplet, br = broad. High-

resolution mass spectra (HRMS) were recorded on an Agilent LC/MSD TOF mass spectrometer by electrospray ionization time-of-flight (ESI-TOF) reflection experiments. Preparative HPLC was performed with an Agilent 1290 Infinity II HPLC and a silica column that is 20 mm I.D. x 250 mm in length and particle size of 25 µm. A 75 W xenon lamp integrated in a PTI QuantaMaster 40 fluorescence spectrophotometer was used for photoluminescence measurements.

Experimental procedures

Preparation of starting materials

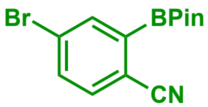

S1

S1 was synthesized following a previously reported procedure[9] with slight modifications. Pinacol (288 mg, 2.4 mmol, 1.1 equiv) and (5-bromo-2-cyanophenyl)boronic acid (0.5 g, 2.2 mmol, 1.0 equiv) were added in a 25 mL round-bottom-flask followed by 10 mL of Et_2O. The reaction mixture was stirred at room temperature overnight. Upon the reaction completion, the solution was diluted with 50 mL of Et_2O and then washed with distilled water (2×20 mL). The organic phase was dried over $MgSO_4$, concentrated under reduced pressure to afford **S1** as a white solid (647 mg, 2.1 mmol, 95%).

The spectroscopic data for this compound were identical to those reported in the literature.[10]
^{1}H NMR (400 MHz, $CDCl_3$) δ: 8.02 (d, J = 2.0 Hz, 1 H), 7.67 (dd, J = 8.3, 2.2 Hz, 1 H), 7.55 (d, J = 8.2 Hz, 1 H), 1.38 (s, 12 H) ppm.

Synthesis of sulfamides: **General Procedure A** (2.1 mmol scale is provided as an example)

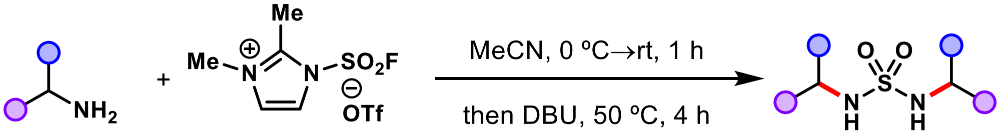

To a flame-dried 100 mL round-bottom-flask equipped with a PTFE-coated stir bar was added the starting amine (2.1 equiv, 2.1 mmol) under argon, followed by anhydrous MeCN (4 mL, C = 0.25 M). The mixture was cooled to 0 °C with an ice-bath, and **2** (328 mg, 1.0 equiv, 1.0 mmol) was

added quickly to the solution in one portion. The reaction mixture was allowed to reach room temperature (~5–10 min) and then stirred for 1 hour. 1,8-diazabicyclo(5.4.0)undec-7-ene (DBU, 0.15 mL, 1.0 mmol, 1.0 equiv) was subsequently added dropwise over 1 minute and then the reaction mixture was stirred at 50 °C. The progression of the reaction was monitored by TLC. Upon completion, the reaction was quenched by the addition of *aq.* HCl (C = 1 M, ~20 mL). The mixture was extracted with EtOAc (~3×20 mL), and the combined organic layers were washed with brine (~20 mL), dried over Na_2SO_4 and then filtered. The solvent was evaporated *in vacuo* and the crude material was purified by column chromatography to afford the desired product. With the exception of **3a** and **S7**, all sulfamides were formed as a mixture of meso and non-meso diastereomers that were typically co-eluting on column chromatography (except **S11**). The dr is indicated when it could be calculated via ^{1}H NMR analysis. Since the stereochemistry is inconsequential for the ultimate cross-coupling, diastereomeric mixture were carried through without separation to the next step.

Synthesis and characterization of sulfamides

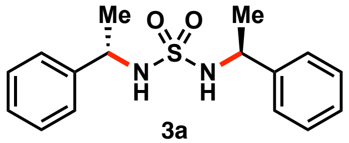

3a

3a was prepared from (*S*)-(–)-1-phenylethylamine (**1a**) (318 mg, 2.1 mmol) following **general procedure A**. Column chromatography (SiO_2, 10:90 to 15:85 EtOAc:hexanes) afforded **3a** as a white solid (272 mg, 90%).

The spectroscopic data for this compound were identical to those reported in the literature.[11]
^{1}H NMR ($CDCl_3$, 400 MHz) δ: 7.64–7.50 (m, 8 H), 4.89–4.80 (m, 2 H), 1.79–1.71 (m, 6 H), 1.58–1.47 (m, 18 H) ppm.

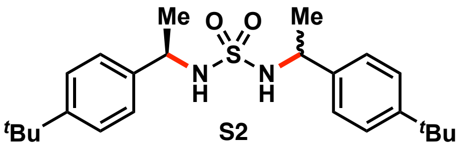

S2

S2 was prepared from 4-tert-butylbenzylamine (372 mg, 2.1 mmol) following **general procedure A**. Column chromatography (SiO_2, 20:80 EtOAc:hexanes) afforded **S2** as a white solid (341 mg, 82%).

R_f = 0.30 (20:80 EtOAc:hexanes)

^{1}H NMR ($CDCl_3$, 400 MHz) mixture of diastereomers δ: 7.35 (d, *J* = 8.4 Hz, 2 H), 7.29 (d, *J* = 8.4 Hz, 2 H), 7.21 (d, *J* = 8.4 Hz, 2 H), 7.10 (d, *J* = 8.3 Hz, 2 H), 4.47–4.37 (m, 2 H), 4.24–4.16 (m, 2 H), 1.47 (d, *J* = 6.8 Hz, 3 H), 1.31–1.29 (m, 18 H), 1.28 (d, *J* = 6.8 Hz, 3 H) ppm.
^{13}C NMR ($CDCl_3$, 101 MHz) mixture of diastereomers δ: 150.7, 140.0, 139.6, 126.1, 126.1, 125.7, 53.7, 53.5, 34.6, 31.0, 23.5, 23.4 ppm.
HRMS(+ESI) calc'd for $C_{24}H_{36}N_2O_2S$ $[M+H]^+$ 417.2570, found 417.2569.

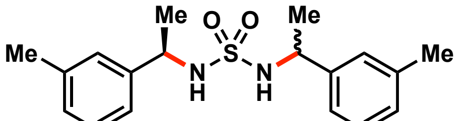

S3

S3 was prepared from 1-(3,5-dimethylphenyl)ethan-1-amine (1.0 g, 6.7 mmol) following **general procedure A**. Column chromatography (SiO_2, 20:80 EtOAc:hexanes) afforded **S3** as a white solid (870 mg, 76%).

R_f = 0.40 (20:80 EtOAc:hexanes)
^{1}H NMR ($CDCl_3$, 400 MHz) mixture of diastereomers (dr ~ 1:1.1) (major) δ: 6.89 (s, 2 H), 6.83 (br, 4 H), 5.00–4.91 (m, 2 H), 4.44–4.31 (m, 2 H), 2.30 (s, 12 H), 1.51 (d, *J* = 6.8 Hz, 6 H); (minor) δ: 6.97 (br, 4 H), 6.94 (s, 2 H), 5.00–4.91 (m, 2 H), 4.44–4.31 (m, 2 H), 2.35 (s, 12 H), 1.32 (d, *J* = 6.8 Hz, 6 H) ppm.
^{13}C NMR ($CDCl_3$, 101 MHz) mixture of diastereomers (dr ~ 1:1.1) δ: 143.3, 142.9, 138.1, 138.1, 129.1, 129.1, 124.0, 123.8, 53.7, 53.6, 23.9, 23.6, 21.3, 21.3 ppm.
HRMS(+ESI) calc'd for $C_{20}H_{28}N_2O_2S$ $[M+H]^+$ 361.1944, found 361.1941.

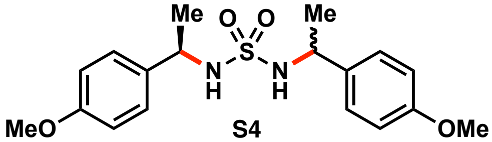

S4

S4 was prepared from 1-(4-methoxyphenyl)ethylamine (1.02 g, 6.7 mmol) following **general procedure A**. Column chromatography (SiO_2, 40:60 EtOAc:hexanes) afforded **S4** as a while solid (1.08 g, 92%).

R_f = 0.14 (20:80 EtOAc:hexanes)
^{1}H NMR ($CDCl_3$, 400 MHz) mixture of diastereomers δ: 7.20 (d, *J* = 8.7 Hz, 2 H), 7.06 (d, *J* = 8.7 Hz, 2 H), 6.85 (d, *J* = 8.7 Hz, 2 H), 6.76 (d, *J* = 8.7 Hz, 2 H), 4.51–4.46 (m, 1 H), 4.46–4.42 (m, 1 H), 4.41–4.32 (m, 2 H), 3.80–3.75 (m, 6 H), 1.44 (d, *J* = 6.7 Hz, 3 H), 1.28 (d, *J* = 6.8 Hz, 3 H) ppm.

^{13}C NMR ($CDCl_3$, 101 MHz) mixture of diastereomers δ: 159.1, 159.1, 135.2, 134.8, 127.5, 127.4, 114.1, 114.1, 55.4, 55.3, 53.4, 53.2, 23.8, 23.6 ppm.
HRMS(+ESI) calc'd for $C_{18}H_{24}N_2O_4S$ $[M+H]^+$ 365.1530, found 365.1530.

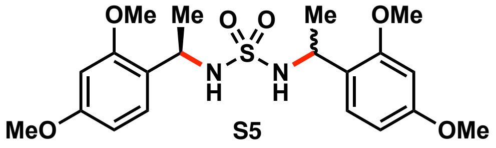

S5 was prepared from 1-(2,4-dimethoxyphenyl)ethan-1-amine (315 mg, 1.7 mmol) following **general procedure A.** Column chromatography (SiO_2, 25:75 to 40:60 EtOAc:hexanes) afforded **S5** as a colorless-sticky solid (237 mg, 68%).

R_f = 0.32 (40:60 EtOAc:hexanes)
^1H NMR ($CDCl_3$, 400 MHz) mixture of diastereomers (dr ~ 1:1.3) (major) δ: 7.04 (d, J = 8.2 Hz, 2 H), 6.46–6.38 (m, 4 H), 4.97 (br, 2 H), 4.50–4.36 (m, 2 H), 3.80 (s, 6 H), 3.79 (s, 6 H), 1.29 (d, J = 6.9 Hz, 6 H); (minor) δ: 6.83 (d, J = 8.2 Hz, 2 H), 6.33–6.25 (m, 4 H), 4.97 (br, 2 H), 4.50–4.36 (m, 2 H), 3.77 (s, 6 H), 3.73 (s, 6 H), 1.43 (d, J = 6.9 Hz, 6 H) ppm.
^{13}C NMR ($CDCl_3$, 101 MHz) mixture of diastereomers (dr ~ 1:1.3) δ: 160.0, 159.8, 157.5, 157.3, 128.3, 128.0, 123.7, 123.4, 104.1, 104.0, 98.9, 98.7, 55.3, 55.2, 55.2, 55.1, 51.0, 50.9, 22.9, 22.4 ppm.
HRMS(+ESI) calc'd for $C_{20}H_{28}N_2O_6S$ $[M+H]^+$ 425.1741, found 425.1728.

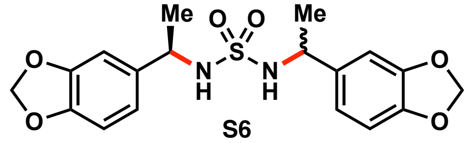

S6 was prepared from 1-(1,3-benzodioxol-5-yl)ethanamine (210 mg, 1.26 mmol) following **general procedure A**. Column chromatography (SiO_2, 20:80 EtOAc:hexanes) afforded **S6** as a white solid (191 mg, 81%).

R_f = 0.33 (25:75 EtOAc:hexanes)
^1H NMR ($CDCl_3$, 400 MHz) mixture of diastereomers δ: 6.77–6.73 (m, 3 H), 6.68–6.65 (m, 1 H), 6.62–6.59 (m, 2 H), 5.59–5.91 (m, 4 H), 4.38–4.24 (m, 4 H), 1.44 (d, J = 6.6 Hz, 3 H), 1.30 (d, J = 6.6 Hz, 3 H) ppm.
^{13}C NMR ($CDCl_3$, 126 MHz) δ: 148.1, 148.0, 147.2, 147.1, 137.0, 136.6, 119.7, 119.6, 108.4, 108.3, 106.8, 106.6, 101.3, 53.8, 53.7, 24.0, 23.8 ppm.
HRMS(+ESI) calc'd for $C_{18}H_{20}N_2O_6S$ $[M+H]^+$ 393.1115, found 393.1116.

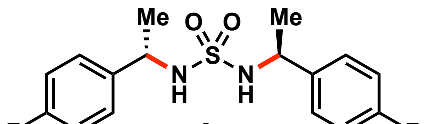

S7

S7 was prepared from (*S*)-(–)-1-phenylethylamine (292 mg, 2.1 mmol) following **general procedure A**. Column chromatography (SiO_2, 20:80 EtOAc:hexanes) afforded **S7** as a white solid (314 mg, 92%).

R_f = 0.10 (20:80 EtOAc:hexanes)

^{1}H NMR ($CDCl_3$, 400 MHz) δ: 7.13–7.08 (m, 4 H), 6.97–6.90 (m, 4 H), 4.41 (p, *J* = 6.7 Hz, 2 H), 4.35–4.26 (m, 2 H), 1.45 (d, *J* = 6.7 Hz, 6 H) ppm.

^{13}C NMR ($CDCl_3$, 126 MHz) δ:162.3 (d, *J* = 246.4 Hz), 138.4 (d, *J* = 3.3 Hz), 127.8 (d, *J* = 8.0 Hz), 115.7 (d, *J* = 21.4 Hz), 53.3, 23.9 ppm.

^{19}F NMR ($CDCl_3$, 470 MHz) δ: –(114.4–114.5) (m) ppm.

HRMS(-ESI) calc'd for $C_{16}H_{18}FN_2O_2S$ $[M–H]^-$ 339.0973, found 339.0987.

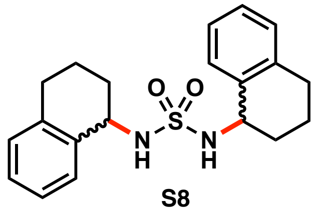

S8

S8 was prepared from 1,2,3,4-tetrahydro-1-naphthylamine (368 mg, 2.1 mmol) following **general procedure A**. Column chromatography (SiO_2, 20:80 EtOAc:hexanes) afforded **S8** as a white solid (227 mg, 64%).

R_f = 0.18 (10:90 EtOAc:hexanes)

^{1}H NMR ($CDCl_3$, 400 MHz) δ: 7.55–7.45 (m, 2 H), 7.23–7.14 (m, 4 H), 7.14–7.06 (m, 2 H), 4.75–4.66 (m, 2 H), 4.35 (d, *J* = 8.0 Hz, 2 H), 2.88–2.70 (m, 4 H), 2.20–1.98 (m, 4 H), 1.96–1.79 (m, 4 H) ppm.

^{13}C NMR ($CDCl_3$, 126 MHz) δ: 137.8, 137.7, 136.1, 136.1, 129.4, 129.4, 129.1, 129.1, 127.8, 126.5, 126.5, 52.5, 30.9, 29.1, 29.1, 19.4 ppm.

HRMS(+ESI) calc'd for $C_{20}H_{24}N_2O_2S$ $[M+H]^+$ 357.1631 , found 357.1623.

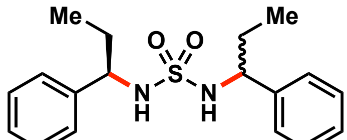

S9

S9 was prepared from α-ethylbenzylamine (1.75 g, 13.0 mmol) following **general procedure A**. Column chromatography (SiO_2, 25:75 EtOAc:hexanes) afforded **S9** as a yellow oil (1.52 g, 74%).

R_f = 0.23 (20:80 EtOAc:hexanes)

^{1}H NMR ($CDCl_3$, 400 MHz) mixture of diastereomers δ: 7.35–7.17 (m, 8 H, *overlaps with* $CDCl_3$), 7.06–7.02 (m, 2 H), 4.40 (d, *J* = 6.2 Hz, 1 H), 4.29 (d, *J* = 6.5 Hz, 1 H), 4.15–4.04 (m, 2 H), 1.90–1.78 (m, 1 H), 1.74–1.61 (m, 1 H), 1.55–1.47 (m, 2 H), 0.74 (t, *J* = 7.3 Hz, 3 H), 0.67 (t, *J* = 7.4 Hz, 3 H) ppm.
^{13}C NMR ($CDCl_3$, 126 MHz) δ: 141.6, 141.2, 128.7, 128.7, 127.8, 127.7, 127.0, 126.9, 60.1, 59.8, 30.4, 30.3, 10.4, 10.4 ppm.
HRMS(+ESI) calc'd for $C_{18}H_{24}N_2O_2S$ $[M+H]^+$ 333.1631, found 333.1631.

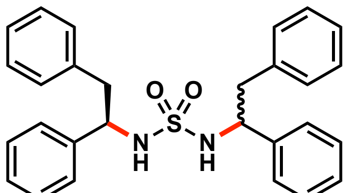

S10

S10 was prepared from 1,2-diphenylethylamine (1.5 g, 7.6 mmol) following **general procedure A**. Column chromatography (SiO_2, 30:70 EtOAc:hexanes) afforded **S10** as an off-white solid (1.6 g, 94%).

R_f = 0.29 (20:80 EtOAc:hexanes)
^{1}H NMR ($CDCl_3$, 400 MHz) mixture of diastereomers δ: 7.32–6.80 (m, 20 H, *overlaps with* $CDCl_3$), 4.55–4.23 (m, 4 H), 3.09–2.77 (m, 4 H) ppm.
^{13}C NMR ($CDCl_3$, 126 MHz) δ: 140.9, 140.4, 136.6, 136.3, 129.7, 129.7, 128.6, 128.6, 128.5, 128.5, 128.5, 127.8, 127.8, 127.0, 127.0, 126.9, 59.5, 59.0, 43.9, 43.7 ppm.
HRMS(+ESI) calc'd for $C_{28}H_{28}N_2O_2S$ $[M+H]^+$ 457.1944, found 457.1946.

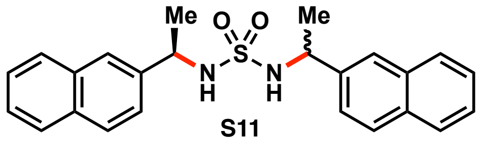

S11

S11 was prepared from 1-(2-naphthyl)ethylamine (171 mg, 1.0 mmol) following **general procedure A**. Column chromatography (SiO_2, 40:60 EtOAc:hexanes) afforded **S11** as a white solid (117 mg, 61%).

R_f = 0.20 (20:80 EtOAc:hexanes)
^{1}H NMR (DMSO-d_6, 400 MHz) mixture of diastereomers δ: 7.94–7.39 (m, 14 H), 4.66–4.26 (m, 2 H), 1.51–1.16 (m, 6 H) ppm.
^{13}C NMR (DMSO-d_6, 126 MHz) δ: 142.3, 142.2, 132.8, 132.7, 132.1, 132.0, 127.7, 127.6, 127.6, 127.6, 127.4, 127.3, 126.0, 125.9, 125.5, 125.5, 124.8, 124.3, 124.2, 52.5, 52.5, 23.8, 23.7 ppm.
HRMS(–APCI) calc'd for $C_{24}H_{24}N_2O_2S$ $[M–H]^-$ 403.1475, found 403.1485.

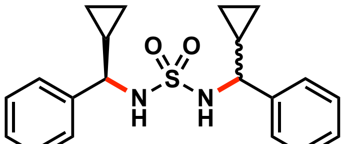

S12

S12 was prepared from cyclopropyl(phenyl)methanamine (1.18 g, 8.0 mmol) following **general procedure A**. Column chromatography (SiO_2, 30:70 EtOAc:hexanes) afforded **S12** as a white solid (0.94 g, 73%).

R_f = 0.19 (20:80 EtOAc:hexanes)

^{1}H NMR ($CDCl_3$, 400 MHz) mixture of diastereomers δ: 7.35–7.22 (m, 8 H, *overlaps with CDCl₃*), 7.19–7.14 (m, 2 H), 4.44 (br, 2 H), 3.72–3.63 (m, 2 H), 1.18–1.07 (m, 1 H), 1.01–0.90 (m, 1 H), 0.63–0.55 (m, 1 H), 0.53–0.36 (m, 4 H), 0.31–0.16 (m, 3 H) ppm.

^{13}C NMR ($CDCl_3$, 126 MHz) δ: 141.1, 141.1, 128.6, 127.9, 127.8, 127.2, 127.1, 62.9, 62.7, 18.1, 18.0, 5.0, 4.7, 3.6, 3.5 ppm.

HRMS(+ESI) calc'd for $C_{20}H_{24}N_2O_2S$ $[M+H]^+$ 357.1631, found 357.1630.

Synthesis of diazenes: **General Procedure B** (1.6 mmol scale is provided as an example)

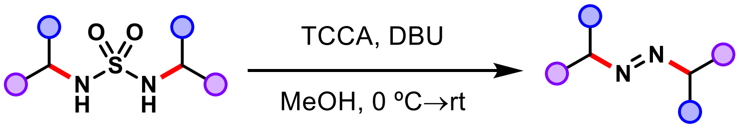

The starting sulfamide (1.6 mmol, 1.0 equiv) was placed in a flame-dried 50 mL round-bottom-flask equipped with a PTFE-coated stir bar. The flask was evacuated and placed under Ar. Following addition of dry MeOH (10.7 mL, C = 0.15 M) and DBU (1.2 mL, 8.0 mmol, 5.0 equiv), the mixture was cooled to 0 °C with an ice-bath and a solution of trichloroisocyanuric acid (TCCA, 409 mg, 1.8 mmol, 1.1 equiv) in dry MeOH (5.3 mL, C = 0.30 M) under argon was added using a syringe over 5 min at 0 °C. The reaction mixture was then warmed to room temperature and stirred until full conversion was reached as shown by TLC. The volatiles were removed *in vacuo* with the temperature of the rotary evaporator bath kept below 30 °C, and the resulting residues were purified by column chromatography to afford the desired product. With the exception of **4a** and **S17**, all sulfamides were formed as a mixture of meso and non-meso diastereomers that were typically co-eluting on column chromatography (except **S21**). The dr is indicated when it could be calculated via ^{1}H NMR analysis. Since the stereochemistry is inconsequential for the ultimate cross-coupling, diastereomeric mixture were carried through without separation to the next step.

Note: While we have never observed any explosive or uncontrolled reactions with this procedure, using a blast shield is recommended because of the high reactivity of diazenes. All diazenes were stored at –20 °C in the dark. No decomposition was observed in these conditions over the course of several weeks. Slow decomposition was observed at room temperature over the course of several days.

Synthesis and characterization of diazenes

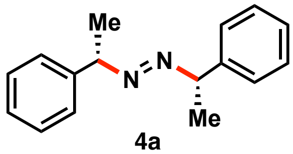

4a

4a was prepared from **3a** (500 mg, 1.6 mmol) following **general procedure B**. Column chromatography (SiO_2, 5:95 EtOAc:hexanes) afforded **4a** as a yellow oil (350 mg, 90%).

R_f = 0.64 (5:95 EtOAc:hexanes)

^{1}H NMR ($CDCl_3$, 400 MHz) δ: 7.39–7.31 (m, 8 H), 7.30–7.24 (m, 2 H, *overlaps with $CDCl_3$*), 4.65 (q, J = 6.8 Hz, 2 H), 1.57 (d, J = 6.8 Hz, 6 H) ppm.

^{13}C NMR ($CDCl_3$, 126 MHz) δ: 141.3, 128.6, 127.5, 127.5, 76.6, 20.5 ppm.

HRMS(+ESI) calc'd for $C_{16}H_{18}N_2$ $[M+H]^+$ 293.1543, found 293.1535.

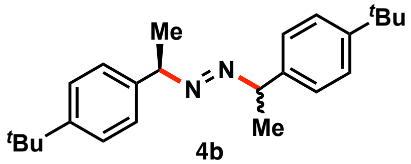

4b

4b was prepared from **S2** (730 mg, 1.75 mmol) following **general procedure B**. Column chromatography (SiO_2, 5:95 EtOAc:hexanes) afforded **4b** as a white solid (576 mg, 94%).

R_f = 0.65 (5:95 EtOAc:hexanes)

^{1}H NMR ($CDCl_3$, 400 MHz) mixture of diastereomers δ: 7.64–7.50 (m, 8 H), 4.89–4.80 (m, 2 H), 1.79–1.71 (m, 6 H), 1.58–1.47 (m, 18 H) ppm.

^{13}C NMR ($CDCl_3$, 126 MHz) mixture of diastereomers δ: 150.1, 150.1, 138.3, 138.3, 127.1, 127.1, 125.5, 125.5, 76.3, 76.1, 34.5, 34.5, 31.5, 31.5, 20.5, 20.2 ppm.

HRMS(+ESI) calc'd for $C_{24}H_{34}N_2$ $[M+H]^+$ 351.2795, found 351.2789.

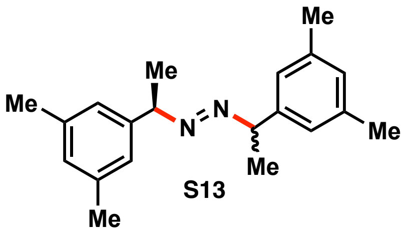

S13 was prepared from **S3** (500 mg, 1.2 mmol) following **general procedure B**. Column chromatography (SiO_2, 5:95 EtOAc:hexanes) afforded **S13** as a white solid (328 mg, 93%).

R_f = 0.68 (5:95 EtOAc:hexanes)

^{1}H NMR ($CDCl_3$, 400 MHz) mixture of diastereomers (dr ~ 1:1.2) (major) δ: 7.04 (s, 2 H), 6.99 (s, 2 H), 6.91 (s, 2 H), 4.58 (q, J = 6.8 Hz, 2 H), 2.30 (s, 12 H), 1.57 (d, J = 6.8 Hz, 6 H); (minor) δ: 7.04 (s, 2 H), 6.99 (s, 2 H), 6.94 (s, 2 H), 4.58 (q, J = 6.8 Hz, 2 H), 2.33 (s, 12 H), 1.52 (d, J = 6.9 Hz, 6 H) ppm.

^{13}C NMR ($CDCl_3$, 126 MHz) mixture of diastereomers (dr ~ 1:1.2) δ: 141.3, 141.2, 138.1, 138.1, 129.1, 129.1, 125.3, 76.8, 76.5, 21.4, 21.4, 20.3 ppm.

HRMS(+ESI) calc'd for $C_{20}H_{26}N_2$ $[M+H]^+$ 295.2169, found 295.2167.

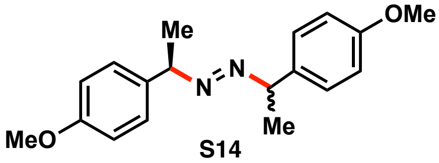

S14 was prepared from **S4** (394 mg, 1.1 mmol) following **general procedure B**. Column chromatography (SiO_2, 5:95 EtOAc:hexanes) afforded **S14** as a transparent oil (240 mg, 74%).

R_f = 0.32 (5:95 EtOAc:hexanes)

^{1}H NMR ($CDCl_3$, 400 MHz) mixture of diastereomers δ: 7.34–7.26 (m, 4 H, *overlaps with* $CDCl_3$), 6.94–6.84 (m, 4 H), 4.61–4.51 (m, 2 H), 3.84–3.75 (m, 6 H), 1.55–1.49 (m, 6 H) ppm.

^{13}C NMR ($CDCl_3$, 126 MHz) mixture of diastereomers δ: 159.1, 159.0, 133.4, 133.4, 128.6, 114.1, 114.1, 76.1, 75.9, 55.4, 55.4, 20.3, 20.1 ppm.

HRMS(+ESI) calc'd for $C_{18}H_{22}N_2O_2$ $[M+H]^+$ 299.1751, found 299.1751.

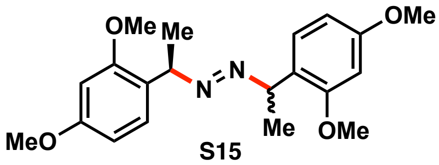

S15 was prepared from **S5** (380 mg, 0.9 mmol) following **general procedure B**. Column chromatography (SiO_2, 5:95 EtOAc:hexanes) afforded **S15** as a transparent oil (258 mg, 80%).

R_f = 0.13 (5:95 EtOAc:hexanes)

^{1}H NMR ($CDCl_3$, 400 MHz) mixture of diastereomers δ: 7.42–7.36 (m, 2 H), 6.53–6.48 (m, 2 H), 6.46 (d, J = 2.5 Hz, 1 H), 6.42 (d, J = 2.0 Hz, 1 H), 5.04–4.98 (m, 2 H), 3.81 (s, 3 H), 3.80 (s, 3 H), 3.76 (s, 3 H), 3.69 (s, 3 H), 1.47–1.40 (m, 6 H) ppm.

^{13}C NMR ($CDCl_3$, 126 MHz) mixture of diastereomers δ: 160.0, 160.0, 158.3, 158.3, 128.4, 128.3, 123.1, 123.1, 104.4, 104.4, 98.8, 98.7, 69.3, 69.2, 55.5, 55.5, 55.5, 55.4, 19.6 ppm.

HRMS(+ESI) calc'd for $C_{20}H_{26}N_2O_4$ $[M+H]^+$ 359.1965, found 359.1951.

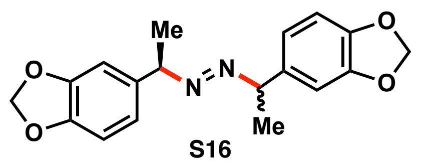

S16 was prepared from **S6** (503 mg, 1.2 mmol) following **general procedure B**. Column chromatography (SiO_2, 5:95 EtOAc:hexanes) afforded **S16** as a white solid (333 mg, 84%).

R_f = 0.39 (10:90 EtOAc:hexanes)

^{1}H NMR ($CDCl_3$, 400 MHz) mixture of diastereomers δ: 6.93–6.86 (m, 2 H), 6.85–6.76 (m, 4 H), 5.96–5.93 (m, 4 H), 4.57–4.48 (m, 2 H), 1.52–1.47 (m, 6 H) ppm.

^{13}C NMR ($CDCl_3$, 126 MHz) mixture of diastereomers δ: 147.9, 147.8, 146.9, 146.9, 135.0, 135.0, 120.7, 120.7, 108.4, 108.3, 107.9, 107.9, 101.0, 101.0, 76.4, 76.2, 20.4, 20.2 ppm.

HRMS(+ESI) calc'd for $C_{24}H_{22}N_2$ $[M+H]^+$ 327.1339, found 327.1336.

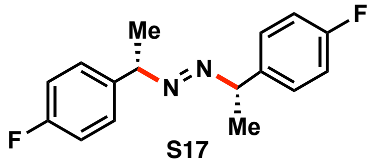

S17 was prepared from **S7** (400 mg, 1.18 mmol) following **general procedure B**. Column chromatography (SiO_2, 5:95 EtOAc:hexanes) afforded **S17** as a transparent oil (298 mg, 92%).

R_f = 0.33 (10:90 EtOAc:hexanes)

^{1}H NMR ($CDCl_3$, 400 MHz) δ: 7.34–7.28 (m, 4 H), 7.05–6.99 (m, 4 H), 4.60 (q, J = 6.9 Hz, 2 H), 1.54 (d, J = 6.8 Hz, 6 H) ppm.

^{13}C NMR ($CDCl_3$, 126 MHz) δ: 162.3 (d, J = 245.7 Hz), 137.0 (d, J = 3.2 Hz), 129.1 (d, J = 7.9 Hz), 115.5 (d, J = 21.3 Hz), 75.9, 20.5 ppm.

^{19}F NMR ($CDCl_3$, 470 MHz) δ: –(115.2–115.3) (m) ppm.

HRMS(+ESI) calc'd for $C_{16}H_{16}F_2N_2$ $[M+H]^+$ 275.1354, found 275.1348.

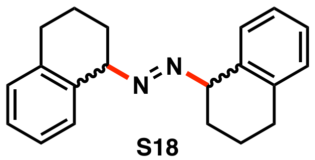

S18

S18 was prepared from **S8** (310 mg, 0.87 mmol) following **general procedure B**. Column chromatography (SiO_2, 5:95 EtOAc:hexanes) afforded **S18** as a transparent oil (213 mg, 84%).

R_f = 0.61 (5:95 EtOAc:hexanes)

^{1}H NMR ($CDCl_3$, 400 MHz) mixture of diastereomers δ: 7.27–7.11 (m, 6 H, *overlaps with $CDCl_3$*), 7.01–6.92 (m, 2 H), 4.61 (t, J = 6.0 Hz, 2 H), 3.04–2.95 (m, 2 H), 2.94–2.85 (m, 2 H), 2.30–2.08 (m, 4 H), 2.07–1.98 (m, 2 H), 1.94–1.85 (m, 2 H) ppm.

^{13}C NMR ($CDCl_3$, 126 MHz) mixture of diastereomers δ: 138.0, 134.0, 133.8, 129.7, 129.6, 129.6, 129.2, 127.6, 127.5, 126.0, 125.9, 75.1, 75.1, 29.7, 29.7, 28.4, 28.3, 20.1 ppm.

HRMS(+ESI) calc'd for $C_{20}H_{22}N_2$ $[M+H]^+$ 291.1856, found 291.1854.

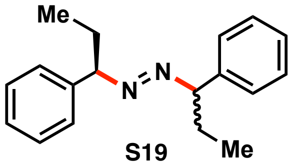

S19

S19 was prepared from **S9** (293 mg, 0.87 mmol) following **general procedure B**. Column chromatography (SiO_2, 5:95 EtOAc:hexanes) afforded **S19** as a transparent oil (141 mg, 61%).

R_f = 0.62 (5:95 EtOAc:hexanes)

^{1}H NMR ($CDCl_3$, 400 MHz) mixture of diastereomers δ: 7.52–7.26 (m, 10 H), 4.35 (q, J = 7.3 Hz, 2 H), 2.13–1.95 (m, 4 H), 0.87 (t, J = 7.4 Hz, 3 H), 0.71 (t, J = 7.4 Hz, 3 H) ppm.

^{13}C NMR ($CDCl_3$, 126 MHz) mixture of diastereomers δ: 140.6, 140.4, 128.6, 128.5, 128.0, 128.0, 127.5, 127.4, 84.1, 84.0, 28.5, 28.2, 10.7, 10.6 ppm.

HRMS(+ESI) calc'd for $C_{18}H_{22}N_2$ $[M+H]^+$ 267.1856, found 267.1853.

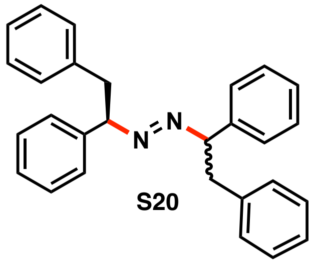

S20

S20 was prepared from **S10** (1.55 g, 3.4 mmol) following **general procedure B**. Column chromatography (SiO_2 pre-deactivated with 5% Et_3N, 5:95 EtOAc:hexanes) afforded **S20** as an off-white solid (800 mg, 61%).

R_f = 0.67 (5:95 EtOAc:hexanes)

^{1}H NMR ($CDCl_3$, 400 MHz) mixture of diastereomers δ: 7.31–7.11 (m, 14 H, *overlaps with $CDCl_3$*), 7.04–6.85 (m, 6 H), 4.85–4.67 (m, 2 H), 3.40–3.10 (m, 4 H) ppm.

^{13}C NMR ($CDCl_3$, 126 MHz) mixture of diastereomers δ: 139.7, 139.5, 138.0, 137.8, 129.7, 129.6, 128.6, 128.5, 128.5, 128.4, 128.3, 128.2, 128.1, 127.8, 127.5, 127.4, 126.3, 126.1, 83.4, 83.3, 41.9, 41.4 ppm.

HRMS(+ESI) calc'd for $C_{28}H_{26}N_2$ $[M+H]^+$ 391.2169, found 391.2168.

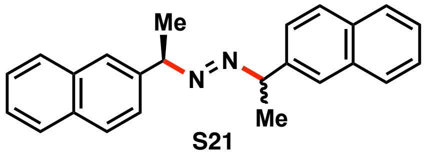
S21

S21 was prepared from **S11** (100 mg, 0.25 mmol) following **general procedure B**. Column chromatography (SiO_2, 5:95 EtOAc:hexanes) afforded **S21** as a white solid (71 mg, 84%).

The spectroscopic data for this compound were identical to those reported in the literature.[12]

^1H NMR ($CDCl_3$, 400 MHz) mixture of diastereomers δ: 7.89–7.83 (m, 8 H), 7.58–7.54 (m, 2 H), 7.50–7.46 (m, 4 H), 4.83 (q, J = 6.8 Hz, 2 H), 1.63 (d, J = 6.9 Hz, 6 H) ppm.

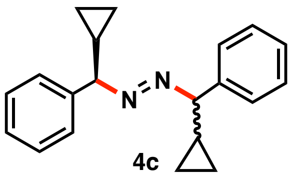
4c

4c was prepared from **S12** (900 mg, 2.52 mmol) following **general procedure B**. Column chromatography (SiO_2 pre-deactivated with 5% Et_3N, 5:95 EtOAc:hexanes) afforded **4c** as a transparent oil (620 mg, 85%).

R_f = 0.60 (10:90 Et_2O:hexanes)

^1H NMR ($CDCl_3$, 400 MHz) mixture of diastereomers δ: 7.59–7.29 (m, 10 H), 3.91–3.82 (m, 2 H), 1.76–1.58 (m, 2 H), 0.77–0.61 (m, 3 H), 0.59–0.37 (m, 4 H), 0.35–0.24 (m, 2 H) ppm.

^{13}C NMR ($CDCl_3$, 126 MHz) mixture of diastereomers δ: 140.2, 140.0, 128.5, 127.8, 127.8, 127.5, 127.5, 86.1, 86.1, 16.0, 15.6, 3.5, 3.5, 3.5, 3.2 ppm.

HRMS(+ESI) calc'd for $C_{20}H_{22}N_2$ $[M+H]^+$ 291.1856, found 291.1854.

Reaction optimization and control experiments

Table S1. Effect of the **solvents** on the cross-coupling reaction of **4a** and **6**

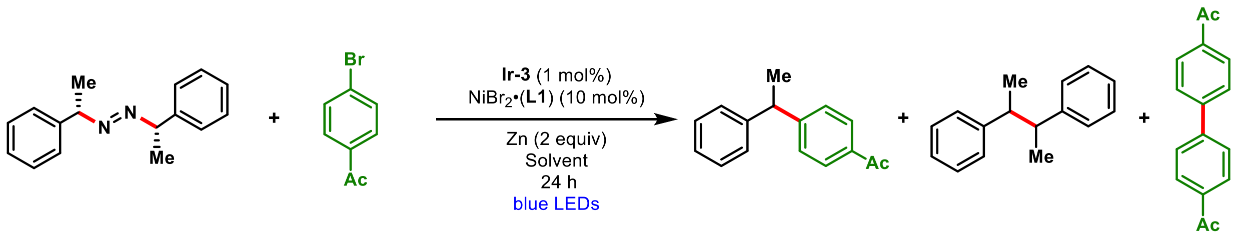

(*S*,*S*)-**4a**, 1 equiv **6**, 3 equiv **7a** **5a** **S22**

Entry	Solvent	Yield **7a**	Yield **5a**	Yield **S22**
1	MeCN	31%	22%	5%
2	Me_2CO_3	n.d.	62%	traces
3	EtOAc	n.d.	65%	traces
4	DMAc (reproducibility issues)	0–40%	Not reproducible	Not reproducible
5	60:40 MeCN:DMAc	30%	10%	12%
6	80:20 MeCN:DMAc	45%	16%	12%
**7**	**95:5 MeCN:DMAc**	**54%**	**18%**	**10%**
8	95:5 MeCN:DMF	50%	9%	4%
9	95:5 MeCN:DMSO	27%	13%	9%

Using **Ir-3** (1 mol%), $NiBr_2$ (0.10 equiv), **L1** (0.10 equiv), Zn powder (2 equiv) under Ar atmosphere at rt in presence of Blue LEDs (0.13 mmol scale). The yields were determined by ^{1}H NMR spectroscopy (400 MHz, $CDCl_3$) using phenyltrimethylsilane as an internal standard.

Table S2. Effect of the **photosensitizers** on the cross-coupling reaction of **4a** and **6**

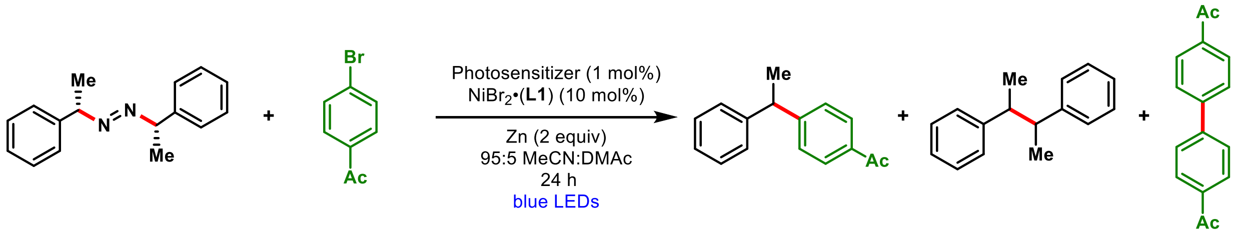

(*S*,*S*)-**4a**, 1 equiv **6**, 3 equiv **7a** **5a** **S22**

Entry	Photosensitizer	Yield **7a**	Yield **5a**	Yield **S22**
1	$Ru(bpy)_3(PF_6)_2$	n.d.	n.d.	5%
2	$Ir(ppy)_3$	n.d.	8%	n.d.
3	$[Ir(dF(CF_3)ppy)_2(bpy)]PF_6$	40%	40%	8%
4	$[Ir(dF(CF_3)ppy)_2(4,4'\text{-dOMebpy})]PF_6$	22%	35%	11%
5	$[Ir(dF(CF_3)ppy)_2(dtbbpy)]PF_6$	16%	61%	4%
6	$Ir(dFppy)_3$	48%	23%	9%
7	4-CzIPN	13%	14%	7%

Using $NiBr_2$ (0.10 equiv), **L1** (0.10 equiv), Zn powder (2 equiv) under Ar atmosphere at rt in presence of Blue LEDs (0.13 mmol scale). The yields were determined by ^{1}H NMR spectroscopy (400 MHz, $CDCl_3$) using phenyltrimethylsilane as an internal standard.

Table S3. Effect of the **Ni source** and **ligand** on the cross-coupling reaction of **4a** and **6**

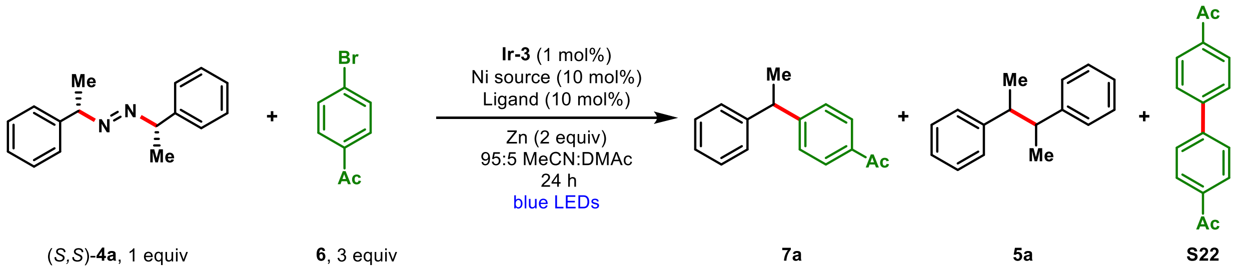

Entry	Nickel Source	Ligand	Yield **7a**	Yield **5a**	Yield **S22**
1	$NiBr_2$	**L1**	38%	16%	traces
2	$NiCl_2$•glyme	**L1**	47%	18%	3%
3	$Ni(COD)_2$	**L1**	45%	21%	6%
4	$Ni(acac)_2$	-	50%	13%	14%
5	$Ni(TMHD)_2$	-	57%	16%	8%
6	$Ni(OTf)_2$	**L1**	32%	43%	traces
7	$NiBr_2$	**L2**	28%	34%	4%
8	$NiBr_2$	**L3**	45%	15%	6%
9	$NiBr_2$	**L4**	42%	25%	traces
10	$NiBr_2$	**L5**	traces	30%	traces

Using **Ir-3** (1 mol%), Ni source (0.10 equiv), ligand (0.10 equiv), Zn powder (2 equiv) under Ar atmosphere at rt in presence of Blue LEDs (0.13 mmol scale). The yields were determined by ^{1}H NMR spectroscopy (400 MHz, $CDCl_3$) using phenyltrimethylsilane as an internal standard.

Table S4. Effect of the **reducing agent** on the cross-coupling reaction of **4a** and **6**

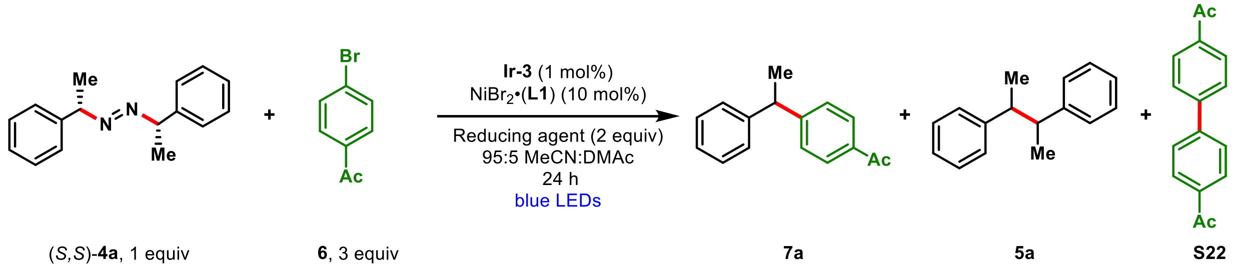

Entry	Reducing agent	Yield **7a**	Yield **5a**	Yield **S22**
1	Zn	65%	28%	4%
2	Mn	45%	27%	8%
3	Hantzsch ester	n.d.	traces	5%
4	Et_3N	n.d.	traces	4%
5	TDAE	n.d.	traces	traces

Using **Ir-3** (1 mol%), $NiBr_2$•**(L1)** (0.15 equiv), Zn powder (2 equiv) under Ar atmosphere at rt in presence of Blue LEDs (0.13 mmol scale). The yields were determined by ^1H NMR spectroscopy (400 MHz, $CDCl_3$) using phenyltrimethylsilane as an internal standard.

Table S5. Effect of the **Ni catalyst loading** on the cross-coupling reaction of **4a** and **6**

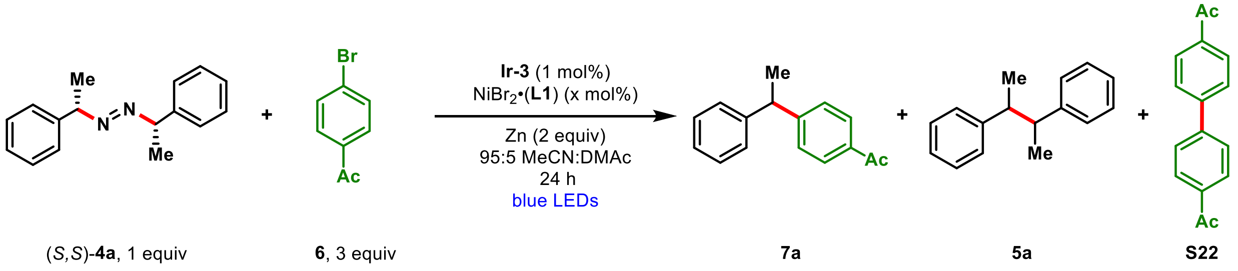

Entry	$NiBr_2$•**(L1)** loading	Yield **7a**	Yield **5a**	Yield **S22**
1	15 mol%	60%	27%	4%
2	20 mol%	52%	13%	9%

Using **Ir-3** (1 mol%), Zn powder (2 equiv) under Ar atmosphere at rt in presence of Blue LEDs (0.13 mmol scale). The yields were determined by ^1H NMR spectroscopy (400 MHz, $CDCl_3$) using phenyltrimethylsilane as an internal standard.

Table S6. Effect of the **additives** on the cross-coupling reaction of **4a** and **6**

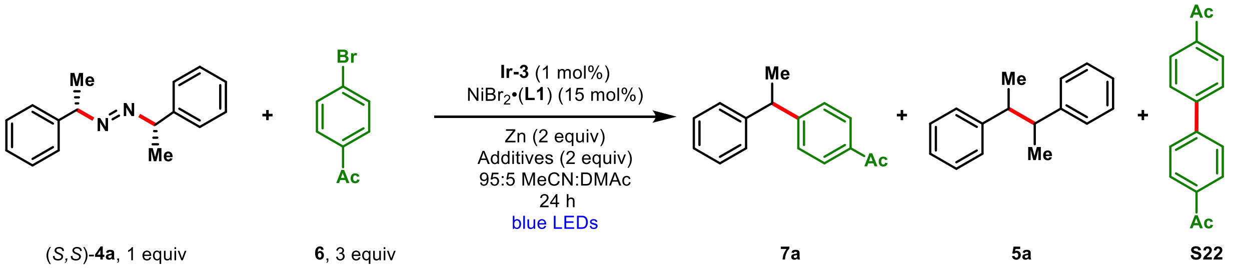

Entry	Additives	Yield **7a**	Yield **5a**	Yield **S22**
1	LiCl	12%	traces	16%
2	$^nBu_4NCl$	15%	traces	8%
3	Cs_2CO_3	60%	29%	5%
4	KH_2PO_4	65%	28%	4%

Using **Ir-3** (1 mol%), $NiBr_2$•**(L1)** (0.15 equiv), Zn powder (2 equiv) under Ar atmosphere at rt in presence of Blue LEDs (0.13 mmol scale). The yields were determined by ^1H NMR spectroscopy (400 MHz, $CDCl_3$) using phenyltrimethylsilane as an internal standard.

Diazene fragmentation and cross-coupling reaction: **General Procedure C**

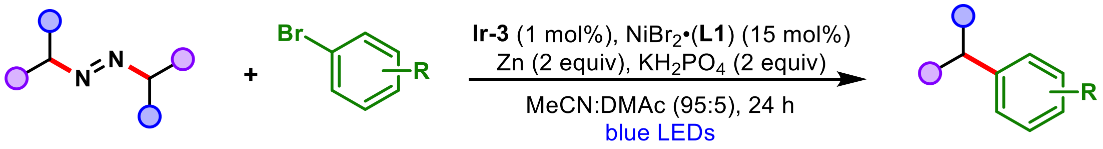

A flame-dried and argon-purged 3 mL vial equipped with a PTFE-coated stir bar was charged with coupling partner (0.38 mmol, 3.0 equiv), photocatalyst **Ir-3** (1.4 mg, 1 mol%), $NiBr_2$**•(di-OMe-bpy)** (8.2 mg, 15 mol%), KH_2PO_4 (34 mg, 0.25 mmol, 2 equiv), and zinc powder (16.5 mg, 2.0 equiv). (Solid diazenes were also added at this stage). Upon addition of all solids, the vial was evacuated under high vacuum for 15 min and then backfilled with argon. Anhydrous degassed MeCN (0.57 mL) was added with a syringe under an argon atmosphere followed by anhydrous degassed DMAc (0.03 mL) with a micro-syringe (liquid diazenes were added at this stage). The vial was then irradiated using blue LEDs (30 W, 450 nm), while the reaction was stirred at 600 rpm. After 24 hours of irradiation, the reaction mixture was diluted with EtOAc (5 mL) and filtered through a silica plug (~ 2.0 cm), which was subsequently flushed with additional EtOAc (50 mL). The volatiles were removed *in vacuo,* and the crude residues were purified by column chromatography.

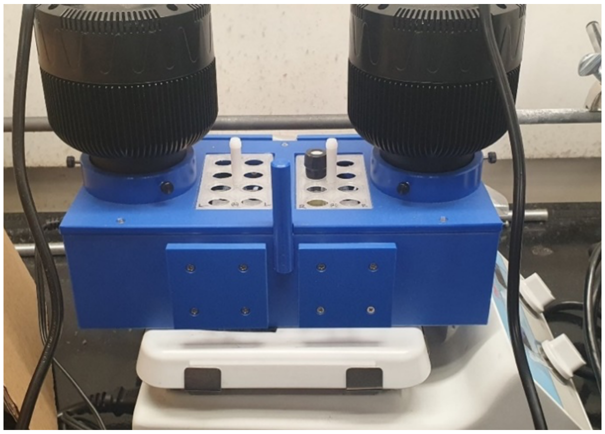

Figure S1. Hepatochem EvoluChemTM PhotoRedOx Box Duo with light sources (EvoluChem™ P303-30-1 LEDs) and vial holders.

Synthesis and characterization of cross-coupling products

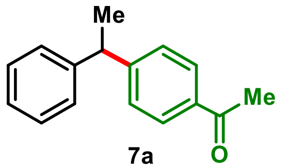

7a

7a was prepared from **4a** (30 mg, 0.13 mmol) and 4′-Bromoacetophenone (75 mg, 0.39 mmol) following **general procedure C**. Column chromatography (SiO_2, 3:97 Et_2O:hexanes) afforded **7a** as a transparent oil (37 mg, 65%).

The spectroscopic data for this compound were identical to those reported in the literature.[13]

^{1}H NMR ($CDCl_3$, 400 MHz) δ: 7.89 (d, J = 8.3 Hz, 2 H), 7.34–7.27 (m, 4 H), 7.24–7.18 (m, 3 H), 4.22 (q, J = 7.2 Hz, 1 H), 2.57 (s, 3 H), 1.67 (d, J = 7.2 Hz, 3 H) ppm.

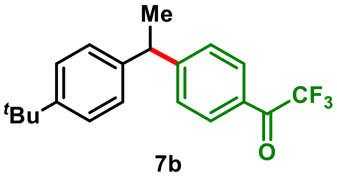

7b

7b was prepared from **4b** (44 mg, 0.13 mmol) and 4′-Bromo-2,2,2-trifluoroacetophenone (96 mg, 0.39 mmol) following **general procedure C**. Column chromatography (SiO_2, 2:98 Et_2O:hexanes) afforded **7b** as a transparent oil (64 mg, 76%).

R_f = 0.63 (10:90 Et_2O:hexanes)

^{1}H NMR ($CDCl_3$, 400 MHz) δ: 8.01 (d, J = 8.8 Hz, 2 H), 7.42 (d, J = 8.5 Hz, 2 H), 7.33 (d, J = 8.4 Hz, 2 H), 7.14 (d, J = 8.3 Hz, 2 H), 4.22 (q, J = 7.2, Hz, 1 H), 1.67 (d, J = 7.2 Hz, 3 H), 1.31 (s, 9 H) ppm.

^{13}C NMR ($CDCl_3$, 126 MHz) δ: 180.2 (q, J = 34.8 Hz), 155.5, 149.6, 141.6, 130.6 (q, J = 2.2 Hz), 128.6, 128.0, 127.3, 125.7, 116.9 (q, J = 291.4 Hz), 44.8, 34.6, 31.5, 21.5 ppm.

^{19}F NMR ($CDCl_3$, 470 MHz) δ: –71.3 ppm.

HRMS(-ESI) calc'd for $C_{20}H_{21}F_3O$ $[M-H]^-$ 333.1461, found 333.1470.

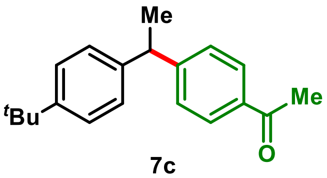

7c

7c was prepared from **4b** (44 mg, 0.13 mmol) and 4′-Bromoacetophenone (75 mg, 0.39 mmol) following **general procedure C**. Column chromatography (SiO_2, hexanes to 2:98 Et_2O:hexanes) afforded **7c** as a transparent oil (52 mg, 74%).

R_f = 0.36 (10:90 Et_2O:hexanes)

^{1}H NMR ($CDCl_3$, 400 MHz) δ: 7.89 (d, J = 8.2 Hz, 2 H), 7.35–7.29 (m, 4 H), 7.14 (d, J = 8.1 Hz, 2 H), 4.18 (q, J = 7.2, Hz, 1 H), 2.57 (s, 3 H), 1.66 (d, J = 7.2 Hz, 3 H), 1.30 (s, 9 H) ppm.

^{13}C NMR ($CDCl_3$, 126 MHz) δ: 197.9, 152.4, 149.3, 142.3, 135.3, 128.7, 128.0, 127.3, 125.5, 44.5, 34.5, 31.5, 26.7, 21.7 ppm.

HRMS(+ESI) calc'd for $C_{20}H_{24}O$ $[M+H]^+$ 281.1900, found 281.1888.

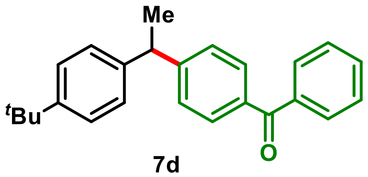

7d

7d was prepared from **4b** (44 mg, 0.13 mmol) and 4-Bromobenzophenone (99 mg, 0.39 mmol) following **general procedure C**. Column chromatography (SiO_2, 1:99 Et_2O:hexanes) afforded **7d** as a transparent oil (63 mg, 73%).

R_f = 0.60 (20:80 EtOAc:hexanes)

^{1}H NMR ($CDCl_3$, 400 MHz) δ: 7.82–7.73 (m, 4 H), 7.60–7.55 (m, 1 H), 7.50–7.44 (m, 2 H), 7.37–7.31 (m, 4 H), 7.18 (d, J = 8.3 Hz, 2 H), 4.21 (q, J = 7.3 Hz, 1 H), 1.68 (d, J = 7.2 Hz, 3 H), 1.32 (s, 9 H) ppm.

^{13}C NMR ($CDCl_3$, 126 MHz) δ: 196.5, 151.8, 149.3, 142.4, 138.0, 135.5, 132.3, 130.5, 130.1, 128.3, 127.7, 127.3, 125.5, 44.6, 34.5, 31.5, 21.8 ppm.

HRMS(+ESI) calc'd for $C_{25}H_{26}O$ $[M+H]^+$ 343.2056, found 343.2054.

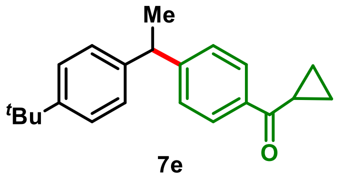

7e

7e was prepared from **4b** (44 mg, 0.13 mmol) and (4-Bromophenyl)(cyclopropyl)methanone (85 mg, 0.39 mmol) following **general procedure C**. Column chromatography (SiO_2, 2:98 to 5:95 Et_2O:hexanes) afforded **7e** as a transparent oil (44 mg, 56%).

R_f = 0.69 (20:80 Et_2O:hexanes)

^{1}H NMR ($CDCl_3$, 400 MHz) δ: 7.95 (d, J = 8.4 Hz, 2 H), 7.36–7.29 (m, 4 H), 7.15 (d, J = 8.2 Hz, 2 H), 4.19 (q, J = 7.2, Hz, 1 H), 2.69–2.61 (m, 1 H), 1.66 (d, J = 7.2 Hz, 3 H), 1.30 (s, 9 H), 1.24–1.19 (m, 2 H), 1.04–0.98 (m, 2 H) ppm.

^{13}C NMR ($CDCl_3$, 126 MHz) δ: 200.3, 152.0, 149.2, 142.5, 136.1, 128.4, 127.9, 127.3, 125.5, 44.5, 34.5, 31.5, 21.7, 17.1, 11.6 ppm.

HRMS(+ESI) calc'd for $C_{22}H_{26}O$ $[M+H]^+$ 307.2056, found 307.2051.

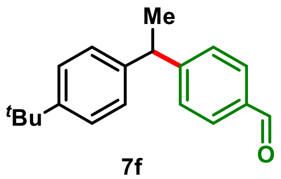

7f

7f was prepared from **4b** (44 mg, 0.13 mmol) and 4-Bromobenzaldehyde (70 mg, 0.39 mmol) following **general procedure C**. Column chromatography (SiO_2, 0.5:99.5 EtO_2:hexanes) afforded **7f** as a colorless oil (47 mg, 70%).

R_f = 0.42 (10:90 Et_2O:hexanes)

^1H NMR ($CDCl_3$, 400 MHz) δ: 9.97 (s, 1H), 7.81 (d, *J* = 8.3 Hz, 2 H), 7.40 (d, *J* = 8.1 Hz, 2 H), 7.32 (d, *J* = 8.4 Hz, 2 H), 7.14 (d, *J* = 8.4 Hz, 2 H), 4.21 (q, *J* = 7.2, Hz, 1 H), 1.67 (d, *J* = 7.2 Hz, 3 H), 1.31 (s, 9 H) ppm.

^{13}C NMR ($CDCl_3$, 126 MHz) δ: 192.1, 154.0, 149.4, 142.1, 134.7, 130.1, 128.5, 127.3, 125.6, 44.7, 34.5, 31.5, 21.7 ppm.

HRMS(+APCI) calc'd for $C_{19}H_{22}O$ $[M+H]^+$ 267.1735 , found 267.1743.

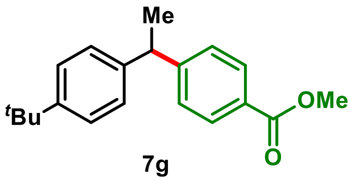

7g

7g was prepared from **4b** (44 mg, 0.13 mmol) and Methyl 4-bromobenzoate (81 mg, 0.39 mmol) following **general procedure C**. Column chromatography (SiO_2, hexanes to 1:99 Et_2O:hexanes) afforded **7g** as a pale yellow solid (48 mg, 65%)

R_f = 0.71 (20:80 Et_2O:hexanes)

^1H NMR ($CDCl_3$, 400 MHz) δ: 7.95 (d, *J* = 8.1 Hz, 2 H), 7.34–7.28 (m, 4 H), 7.13 (d, *J* = 8.2 Hz, 2 H), 4.17 (q, *J* = 7.2, Hz, 1 H), 3.89 (s, 3 H), 1.65 (d, *J* = 7.2 Hz, 3 H), 1.30 (s, 9 H) ppm.

^{13}C NMR ($CDCl_3$, 126 MHz) δ: 167.2, 152.1, 149.2, 142.5, 129.9, 128.1, 127.8, 127.3, 125.5, 52.1, 44.5, 34.5, 31.5, 21.8 ppm.

HRMS(+ESI) calc'd for $C_{20}H_{24}O_2$ $[M+H]^+$ 297.1849, found 297.1844.

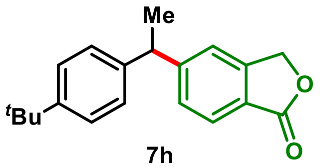

7h

7h was prepared from **4b** (44 mg, 0.13 mmol) and 5-Bromophthalide (80 mg, 0.39 mmol) following **general procedure C**. Column chromatography (SiO_2, 15:85 Et_2O:hexanes) afforded **7h** as a white solid (51 mg, 69%).

R_f = 0.27 (25:75 Et_2O:hexanes)

^1H NMR ($CDCl_3$, 400 MHz) δ: 7.82 (d, J = 8.0 Hz, 1 H), 7.42 (d, J = 8.0 Hz, 1 H), 7.35–7.30 (m, 3 H), 7.14 (d, J = 8.3 Hz, 2 H), 5.26 (s, 2 H), 4.25 (q, J = 7.2 Hz, 1 H), 1.68 (d, J = 7.3 Hz, 3 H), 1.30 (s, 9 H) ppm.

^{13}C NMR ($CDCl_3$, 126 MHz) δ: 171.2, 154.1, 149.6, 147.2, 141.8, 129.1, 127.3, 125.8, 125.7, 123.8, 121.1, 69.7, 44.8, 34.5, 31.5, 21.9 ppm.

HRMS(+ESI) calc'd for $C_{20}H_{22}O_2$ $[M+H]^+$ 295.1693, found 295.1680.

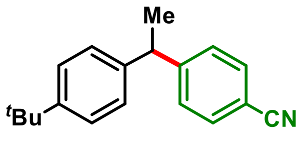

7i

7i was prepared from **4b** (44 mg, 0.13 mmol) and 4-Bromobenzonitrile (69 mg, 0.39 mmol) following **general procedure C**. Column chromatography (SiO_2, 3:97 Et_2O:hexanes) afforded **7i** as a transparent oil (43 mg, 65%).

R_f = 0.37 (5:95 Et_2O:hexanes)

^1H NMR ($CDCl_3$, 400 MHz) δ: 7.57 (d, J = 8.4 Hz, 2 H), 7.35–7.29 (m, 4 H), 7.11 (d, J = 8.2 Hz, 2 H), 4.17 (q, J = 7.2 Hz, 1 H), 1.64 (d, J = 7.2 Hz, 3 H), 1.30 (s, 9 H) ppm.

^{13}C NMR ($CDCl_3$, 126 MHz) δ: 152.3, 149.6, 141.7, 132.4, 128.6, 127.3, 125.7, 119.2, 110.0, 44.6, 34.5, 31.5, 21.6 ppm.

HRMS(+APCI) calc'd for $C_{19}H_{21}N$ $[M+H]^+$ 264.1747, found 264.1742.

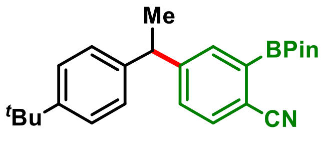

7j

7j was prepared from **4b** (44 mg, 0.13 mmol) and **S1** (116 mg, 0.39 mmol) following **general procedure C**. Column chromatography (SiO_2, 1:1:5:93 TFA:Et_2O:DCM:hexanes) afforded **7j** as a reddish oil (49 mg, 50%).

R_f = 0.10 (5:95 EtOAc:hexanes)

^{1}H NMR ($CDCl_3$, 500 MHz) δ: 7.78 (s, 1 H), 7.60 (d, *J* = 7.8 Hz, 1 H), 7.34–7.27 (m, 3 H), 7.11 (d, *J* = 7.5 Hz, 2 H), 4.17 (q, *J* = 7.3 Hz, 1 H), 1.63 (d, *J* = 7.4 Hz, 3 H), 1.38 (s, 12 H), 1.29 (s, 9 H) ppm.

^{13}C NMR ($CDCl_3$, 126 MHz) δ: 150.8, 149.4, 141.9, 135.3, 133.8, 130.6, 127.3, 125.6, 119.4, 115.0, 84.9, 44.5, 34.5, 31.5, 25.0, 21.7 ppm.

HRMS(+ESI) calc'd for $C_{19}H_{20}N$ $[M+NH_4]^+$ 407.2864, found 407.2854.

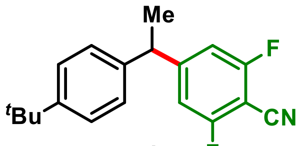

7k

7k was prepared from **4b** (44 mg, 0.13 mmol) and 4-Bromo-2,6-difluorobenzonitrile (82 mg, 0.39 mmol) following **general procedure C**. Column chromatography (SiO_2, 1:99 Et_2O:hexanes) afforded **7k** as a transparent oil (37 mg, 49%).

R_f = 0.46 (5:95 Et_2O:hexanes)

^{1}H NMR ($CDCl_3$, 400 MHz) δ: 7.35 (d, *J* = 8.4 Hz, 2 H), 7.09 (d, *J* = 8.3 Hz, 2 H), 6.90 (d, *J* = 8.7 Hz, 2 H), 4.13 (q, *J* = 7.2 Hz, 1 H), 1.62 (d, *J* = 7.3 Hz, 3 H), 1.31 (s, 9 H) ppm.

^{13}C NMR ($CDCl_3$, 126 MHz) δ: 163.3 (d, *J* = 260.8 Hz), 157.0 (t, *J* = 8.5 Hz), 150.3, 140.2, 127.2, 126.0, 111.5 (d, *J* = 19.5 Hz), 109.5, 90.0 (t, *J* = 19.4 Hz), 44.8, 34.6, 31.5, 21.2 ppm.

^{19}F NMR ($CDCl_3$, 470 MHz) δ: –104.1 (d, *J* = 9.1 Hz) ppm.

HRMS(+APCI) calc'd for $C_{19}H_{19}F_2N$ $[M+H]^+$ 300.1558, found 300.1555.

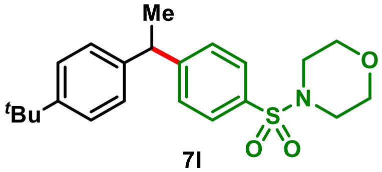

7l

7l was prepared from **4b** (44 mg, 0.13 mmol) and 4-[(4-Bromophenyl)sulfonyl]morpholine (116 mg, 0.39 mmol) following **general procedure C**. Column chromatography (SiO_2, 40:60 Et_2O:hexanes) followed by preparative HPLC (SiO_2, 5:95 ^{i}PrOH:hexanes) afforded **7l** as a white solid (49 mg, 51%).

R_f = 0.15 (20:80 Et_2O:hexanes)

^{1}H NMR ($CDCl_3$, 400 MHz) δ: 7.66 (d, *J* = 8.4 Hz, 2H), 7.39 (d, *J* = 8.2 Hz, 2H), 7.33 (d, *J* = 8.4 Hz, 2H), 7.12 (d, *J* = 8.2 Hz, 2H), 4.20 (q, *J* = 7.2 Hz, 1H), 3.77–3.70 (m, 4H), 3.03–2.96 (m, 4H), 1.65 (d, *J* = 7.2 Hz, 3H), 1.31 (s, 9H) ppm.

^{13}C NMR ($CDCl_3$, 126 MHz) δ: 152.5, 149.6, 141.8, 132.8, 128.5, 128.2, 127.3, 125.7, 66.3, 46.1, 44.5, 34.5, 31.5, 21.8 ppm.
HRMS(+ESI) calc'd for $C_{22}H_{30}NO_2$ $[M+H]^+$ 388.1941, found 388.1937.

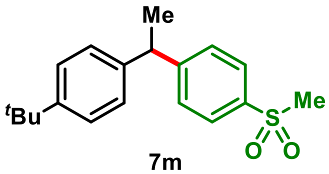

7m

7m was prepared from **4b** (44 mg, 0.13 mmol) and 4-Bromophenyl methyl sulfone (89 mg, 0.39 mmol) following **general procedure C**. Column chromatography (SiO_2, hexanes to 10:90 ^{i}PrOH:hexanes) afforded **7m** as a white solid (50 mg, 63%).

R_f = 0.33 (40:60 Et_2O:hexanes)
^{1}H NMR ($CDCl_3$, 400 MHz) δ: 7.85 (d, *J* = 8.5 Hz, 2 H), 7.42 (d, *J* = 8.3 Hz, 2 H), 7.33 (d, *J* = 8.4 Hz, 2 H), 7.13 (d, *J* = 8.4 Hz, 2 H), 4.21 (q, *J* = 7.2, Hz, 1 H), 3.03 (s, 3 H), 1.66 (d, *J* = 7.2 Hz, 3 H), 1.30 (s, 9 H) ppm.
^{13}C NMR ($CDCl_3$, 126 MHz) δ: 153.3, 149.6, 141.7, 138.3, 128.7, 127.7, 127.3, 125.6, 44.7, 44.5, 34.5, 31.5, 21.7 ppm.
HRMS(+APCI) calc'd for $C_{19}H_{24}O_2S$ $[M+H]^+$ 317.1570, found 317.1564.

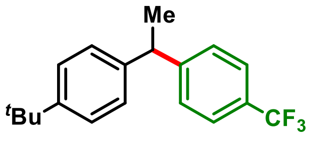

7n

7n was prepared from **4b** (44 mg, 0.13 mmol) and 4-Bromobenzotrifluoride (85 mg, 0.39 mmol) following **general procedure C**. Column chromatography (SiO_2, hexanes) afforded **7n** as a transparent oil (31 mg, 40%).

The spectroscopic data for this compound were identical to those reported in the literature.[14]
^{1}H NMR ($CDCl_3$, 400 MHz) δ: 7.53 (d, *J* = 8.1 Hz, 2 H), 7.36–7.29 (m, 4 H), 7.13(d, *J* = 8.3 Hz, 2 H), 4.18 (q, *J* = 7.2 Hz, 1 H), 1.65 (d, *J* = 7.2 Hz, 3 H), 1.30 (s, 9 H) ppm.
HRMS(+APCI) calc'd for $C_{19}H_{21}F_3$ $[M–2H+H]^{+\bullet}$ 305.1512, found 305.1510.

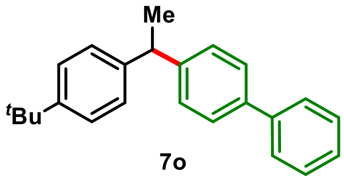

7o

7o was prepared from **4b** (44 mg, 0.13 mmol) and 4-Bromobiphenyl (88 mg, 0.39 mmol) following **general procedure C**. Column chromatography (SiO_2, 1:99 Et_2O:hexanes) afforded **7o** as a white solid (33 mg, 42%).

R_f = 0.46 (1:99 Et_2O:hexanes)

^{1}H NMR ($CDCl_3$, 400 MHz) δ: 7.59–7.54 (m, 2 H), 7.53–7.49 (m, 2 H), 7.44–7.39 (m, 2 H), 7.35–7.28 (m, 5 H), 7.22–7.17 (m, 2 H), 4.17 (q, J = 7.2 Hz, 1 H), 1.67 (d, J = 7.2 Hz, 3 H), 1.30 (s, 9 H) ppm.

^{13}C NMR ($CDCl_3$, 126 MHz) δ: 149.0, 145.9, 143.3, 141.2, 139.0, 128.8, 128.2, 127.3, 127.2, 127.2, 125.4, 44.2, 34.5, 31.5, 22.1 ppm.

HRMS(+APCI) calc'd for $C_{24}H_{26}$ $[M–2H+H]^{+\bullet}$ 313.1951, found 313.1944.

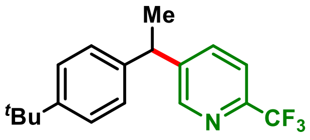

7p

7p was prepared from **4b** (44 mg, 0.13 mmol) and 5-Bromo-2-(trifluoromethyl)pyridine (85 mg, 0.39 mmol) following **general procedure C**. Column chromatography (SiO_2, 1:99 Et_2O:hexanes) afforded **7p** as a transparent oil (24 mg, 31%).

R_f = 0.64 (20:80 EtOAc:hexanes)

^{1}H NMR ($CDCl_3$, 400 MHz) δ: 8.63 (s, 1 H), 7.59 (dd, J = 8.2, 2.2 Hz, 1 H), 7.51 (d, J = 8.2 Hz, 1 H), 7.34 (d, J = 8.4 Hz, 2 H), 7.13 (d, J = 8.2 Hz, 2 H), 4.23 (q, J = 7.3 Hz, 1 H), 1.69 (d, J = 7.2 Hz, 3 H), 1.30 (s, 9 H) ppm.

^{13}C NMR ($CDCl_3$, 126 MHz) δ: 149.9, 149.8, 146.2 (q, J = 34.7 Hz), 145.4, 141.0, 136.2, 127.3, 125.8, 121.8 (q, J = 273.7 Hz), 120.3 (q, J = 2.6 Hz), 42.1, 34.6, 31.5, 21.5 ppm.

^{19}F NMR ($CDCl_3$, 470 MHz) δ: –67.7 ppm.

HRMS(+APCI) calc'd for $C_{18}H_{20}F_3N$ $[M+H]^+$ 308.1621, found 308.1603.

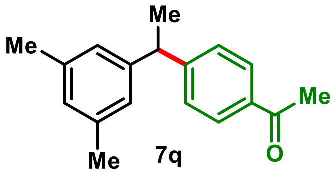

7q

7q was prepared from **S13** (37 mg, 0.13 mmol) 4′-Bromoacetophenone (75 mg, 0.39 mmol) following **general procedure C**. Column chromatography (SiO_2, 3:97 Et_2O:hexanes) afforded **7q** as a transparent oil (46 mg, 73%).

R_f = 0.29 (5:95 Et_2O:hexanes)

^{1}H NMR ($CDCl_3$, 400 MHz) δ: 7.89 (d, J = 8.4 Hz, 2 H), 7.33 (d, J = 8.3 Hz, 2 H), 6.86 (s, 1 H), 6.83 (s, 2 H), 4.14 (q, J = 7.2 Hz, 1 H), 2.58 (s, 3 H), 2.29 (s, 6 H), 1.65 (d, J = 7.2 Hz, 3 H) ppm.

^{13}C NMR ($CDCl_3$, 101 MHz) δ: 197.9, 152.4, 145.4, 138.1, 135.2, 128.7, 128.2, 127.9, 125.5, 44.8, 26.7, 21.7, 21.5 ppm.

HRMS(+APCI) calc'd for $C_{18}H_{20}O$ $[M+H]^+$ 253.1587, found 253.1582.

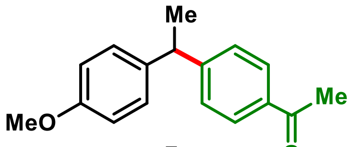

7r

7r was prepared from **S14** (38 mg, 0.13 mmol) and 4′-Bromoacetophenone (75 mg, 0.39 mmol) following **general procedure C**. Column chromatography (SiO_2, 3:97 to 15:85 Et_2O:hexanes) afforded **7r** as a transparent oil (52 mg, 81%).

$R_f = 0.20$ (5:95 EtOAc:hexanes)

^1H NMR ($CDCl_3$, 400 MHz) δ: 7.88 (d, $J = 8.3$ Hz, 2 H), 7.30 (d, $J = 8.4$ Hz, 2 H), 7.13 (d, $J = 8.7$ Hz, 2 H), 6.84 (d, $J = 8.7$ Hz, 2 H), 4.16 (q, $J = 7.2$, Hz, 1 H), 3.78 (s, 3 H), 2.57 (s, 3 H), 1.64 (d, $J = 7.2$ Hz, 3 H) ppm.

^{13}C NMR ($CDCl_3$, 126 MHz) δ: 197.9, 158.2, 152.6, 137.6, 135.2, 128.7, 128.6, 127.9, 114.0, 55.4, 44.1, 26.7, 21.9 ppm.

HRMS(+APCI) calc'd for $C_{17}H_{18}O_2$ $[M+H]^+$ 255.1380, found 255.1375.

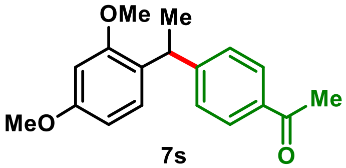

7s

7s was prepared from **S15** (45 mg, 0.13 mmol) and 4′-Bromoacetophenone (75 mg, 0.39 mmol) following **general procedure C**. Column chromatography (SiO_2, 15:85 Et_2O:hexanes) afforded **7s** as a transparent oil (46 mg, 65%).

$R_f = 0.34$ (10:90 EtOAc:hexanes)

^1H NMR ($CDCl_3$, 400 MHz) δ: 7.85 (d, $J = 8.4$ Hz, 2 H), 7.30 (d, $J = 8.3$ Hz, 2 H), 7.06 (d, $J = 8.3$ Hz, 1 H), 6.49–6.40 (m, 2 H), 4.49 (q, $J = 7.2$ Hz, 1 H), 3.79 (s, 3 H), 3.72 (s, 3 H), 2.56 (s, 3 H), 1.57 (d, $J = 7.3$ Hz, 3 H) ppm.

^{13}C NMR ($CDCl_3$, 126 MHz) δ: 198.1, 159.5, 157.9, 153.0, 135.0, 128.5, 127.9, 127.9, 126.4, 104.1, 98.8, 55.5, 55.5, 37.5, 26.7, 20.9 ppm.

HRMS(+APCI) calc'd for $C_{18}H_{20}O_3$ $[M+H]^+$ 285.1485, found 285.1479.

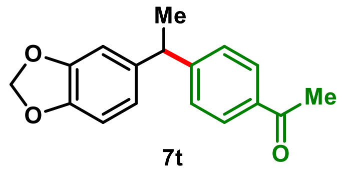

7t

7t was prepared from **S16** (41 mg, 0.13 mmol) and 4′-Bromoacetophenone (75 mg, 0.39 mmol) following **general procedure C**. Column chromatography (SiO_2, 4:96 Et_2O:hexanes) afforded **7t** as waxy oil (32 mg, 47%).

R_f = 0.15 (10:90 Et_2O:hexanes)

^1H NMR ($CDCl_3$, 400 MHz) δ: 7.88 (d, J = 8.4 Hz, 2 H), 7.30 (d, J = 8.2 Hz, 2 H), 6.77–6.64 (m, 3 H), 5.93 (s, 2 H), 4.12 (q, J = 7.2 Hz, 1 H), 2.57 (s, 3 H), 1.61 (d, J = 7.3 Hz, 3 H) ppm.

^{13}C NMR ($CDCl_3$, 126 MHz) δ: 197.9, 152.2, 147.9, 146.1, 139.5, 135.4, 128.7, 127.8, 120.6, 108.3, 108.2, 101.1, 44.6, 26.7, 21.8 ppm.

HRMS(+APCI) calc'd for $C_{17}H_{16}O_3$ $[M+H]^+$ 269.1172, found 269.1169.

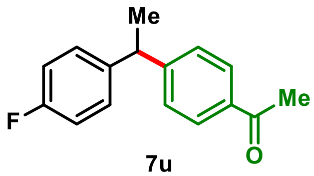

7u

7u was prepared from **S17** (35 mg, 0.13 mmol) and 4′-Bromoacetophenone (75 mg, 0.39 mmol) following **general procedure C**. Column chromatography (SiO_2, 1:99 Et_2O:hexanes) afforded **7u** as a transparent oil (27 mg, 45%).

R_f = 0.40 (20:80 EtOAc:hexanes)

^1H NMR ($CDCl_3$, 400 MHz) δ: 7.89 (d, J = 8.5 Hz, 2 H), 7.29 (d, J = 8.1 Hz, 2 H), 7.16 (dd, J = 8.5, 5.4 Hz, 2 H), 7.01–6.94 (m, 2 H), 4.19 (q, J = 7.2 Hz, 1 H), 2.57 (s, 3 H), 1.64 (d, J = 7.2 Hz, 3 H) ppm.

^{13}C NMR ($CDCl_3$, 126 MHz) δ: 197.9, 161.5 (d, J = 244.7 Hz), 151.9, 141.1 (d, J = 3.3 Hz), 135.4, 129.1 (d, J = 7.9 Hz), 128.8, 127.9, 115.4 (d, J = 21.3 Hz), 44.2, 26.7, 21.8 ppm.

^{19}F NMR ($CDCl_3$, 470 MHz) δ: –(116.7–116.8) (m) ppm.

HRMS(+ESI) calc'd for $C_{16}H_{15}FO$ $[M+H]^+$ 243.1180, found 243.1178.

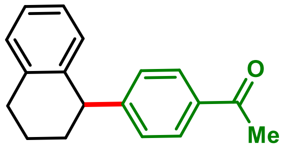

7v

7v was prepared from **S18** (37 mg, 0.13 mmol) and 4′-Bromoacetophenone (75 mg, 0.39 mmol) following **general procedure C** with one exception [$Ni(acac)_2$ (4.9 mg, 15 mol%) was used in lieu of $NiBr_2$**•(di-OMe-bpy)]**. Column chromatography (SiO_2, hexanes to 2:98 Et_2O:hexanes) afforded **7v** as a white oil (27 mg, 43%).

The spectroscopic data for this compound were identical to those reported in the literature.[15]

^{1}H NMR ($CDCl_3$, 400 MHz) δ: 7.88 (d, J = 8.3 Hz, 2 H), 7.20 (d, J = 8.4 Hz, 2 H), 7.17–7.11 (m, 2 H), 7.06–7.01 (m, 1 H), 6.79 (d, J = 7.7 Hz, 1 H), 4.91 (t, J = 6.8 Hz, 1 H), 2.99–2.80 (m, 2 H), 2.58 (s, 3 H), 2.25–2.13 (m, 1 H), 1.95–1.70 (m, 3 H) ppm.

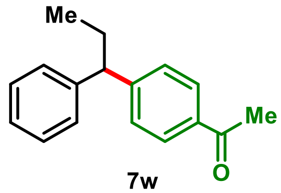

7w

7w was prepared from **S19** (34 mg, 0.13 mmol) and 4′-Bromoacetophenone (75 mg, 0.39 mmol) following **general procedure C**. Column chromatography (SiO_2, 3:97 Et_2O:hexanes) afforded **7w** as a transparent oil (34 mg, 57%).

R_f = 0.31 (5:95 EtOAc:hexanes)

^{1}H NMR ($CDCl_3$, 400 MHz) δ: 7.88 (d, J = 8.0 Hz, 2 H), 7.36–7.27 (m, 4 H), 7.25–7.16 (m, 3 H), 3.86 (t, J = 7.8 Hz, 1 H), 2.56 (s, 3 H), 2.10 (p, J = 7.4 Hz, 2 H), 0.91 (t, J = 7.3 Hz, 3 H) ppm.

^{13}C NMR ($CDCl_3$, 126 MHz) δ: 197.9, 151.0, 144.3, 135.3, 128.7, 128.7, 128.3, 128.0, 126.5, 53.4, 28.4, 26.7, 12.8 ppm.

HRMS(+APCI) calc'd for $C_{17}H_{18}O$ $[M+H]^+$ 239.1430, found 239.1427.

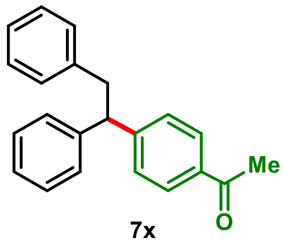

7x

7x was prepared from **S20** (49 mg, 0.13 mmol) and 4′-Bromoacetophenone (75 mg, 0.39 mmol) following **general procedure C** with one exception [Ni(acac)$_2$ (4.9 mg, 15 mol%) was used in lieu of $NiBr_2$**•(di-OMe-bpy)**]. Column chromatography (SiO_2, 4:96 Et_2O:hexanes) afforded **7x** as a transparent oil (36 mg, 48%).

R_f = 0.35 (20:80 Et_2O:hexanes)

^{1}H NMR ($CDCl_3$, 400 MHz) δ: 7.84 (d, J = 8.2 Hz, 2 H), 7.31–7.10 (m, 12 H, *overlaps with $CDCl_3$*), 7.03–6.98 (m, 2 H), 4.30 (t, J = 7.8 Hz, 1 H), 3.45–3.32 (m, 2 H), 2.55 (s, 3 H) ppm.

^{13}C NMR ($CDCl_3$, 126 MHz) δ: 197.9, 150.1, 143.7, 139.8, 135.4, 129.1, 128.7, 128.7, 128.5, 128.3, 128.1, 126.7, 126.2, 53.2, 41.9, 26.7 ppm.

HRMS(+ESI) calc'd for $C_{22}H_{21}O$ $[M+H]^+$ 301.1587, found 301.1583.

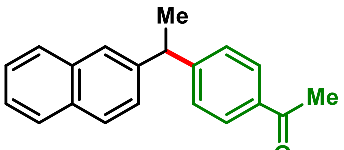

7y

7y was prepared from **S21** (43 mg, 0.13 mmol) and 4′-Bromoacetophenone (75 mg, 0.39 mmol) following **general procedure C**. Column chromatography (SiO_2, 2:98 Et_2O:hexanes) afforded **7y** as a waxy oil (32 mg, 47%).

The spectroscopic data for this compound were identical to those reported in the literature.[16]
^{1}H NMR ($CDCl_3$, 400 MHz) δ: 7.89 (d, J = 8.0 Hz, 2 H), 7.85–7.78 (m, 2 H), 7.75 (d, J = 8.5 Hz, 1 H), 7.70 (s, 1 H), 7.51–7.40 (m, 2 H), 7.35 (d, J = 8.0 Hz, 2 H), 7.31–7.23 (m, 1 H, *overlaps with* $CDCl_3$), 4.37 (q, J = 7.2 Hz, 1 H), 2.57 (s, 3 H), 1.76 (d, J = 7.1 Hz, 3 H) ppm.

Table S7. Deaminative cross-coupling attempts with electron-rich aryl bromides

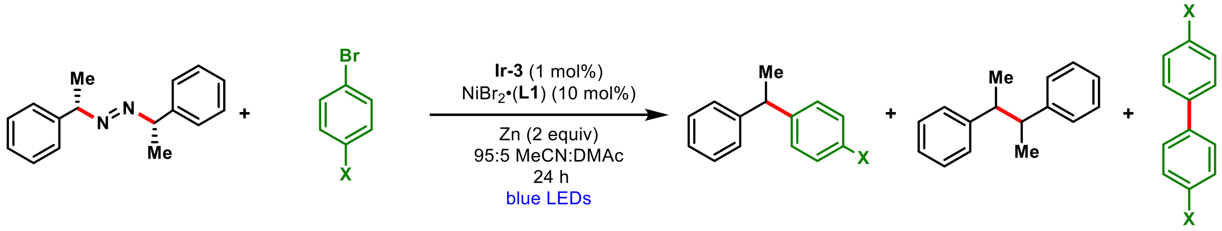

(*S*,*S*)-**4a**, 1 equiv 3 equiv **5a**

Entry	Substituent	Yield desired product	Yield **5a**	Yield biaryl
1	X = H	7%	36%	n.d.
2	X = Me	5%	5%	traces
3	X = ^{t}Bu	10%	33%	6%

<u>Diazene fragmentation and homocoupling via radical recombination: **General Procedure D**</u>

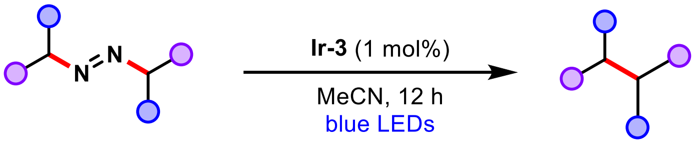

A flame-dried and argon-purged 3 mL vial equipped with a PTFE-coated stir bar was charged with photocatalyst **Ir-3** (1.4 mg, 1 mol%) and a diazene (0.13 mol). The vial was then evacuated under high vacuum for 15 min and then backfilled with argon. Anhydrous degassed MeCN (C = 0.21 M, 0.60 mL) was added with a syringe under an argon atmosphere. The vial was then irradiated using

blue LEDs (30 W, 450 nm), while the reaction was stirred at 600 rpm. After 12 hours of irradiation, the volatiles were removed in vacuo, and the crude residues were purified by column chromatography.

Synthesis and characterization of homocoupling products

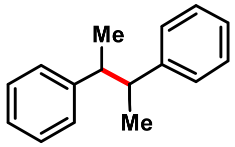
5a

5a was prepared from **4a** (30 mg, 0.13 mmol) following **general procedure D**. Column chromatography (SiO_2, hexanes) afforded **5a** as a white solid (16 mg, 61%).

The spectroscopic data for this compound were identical to those reported in the literature.[17]
^1H NMR ($CDCl_3$, 400 MHz) mixture of diastereomers δ: 7.35–6.97 (m, 10 H, *overlaps with CDCl3*), 2.99–2.89 (m, 1 H), 2.84–2.75 (m, 1 H), 1.29 (d, J = 5.3 Hz, 3 H), 1.03 (d, J = 5.3 Hz, 3 H) ppm

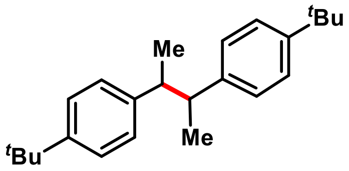
5b

5b was prepared from **4b** (44 mg, 0.13 mmol) following **general procedure D**. Column chromatography (SiO_2, hexanes) afforded **5b** as a white solid (19 mg, 48%).

R_f = 0.47 (hexanes)
^1H NMR ($CDCl_3$, 500 MHz) mixture of diastereomers (dr. ~ 1:2) (major) δ: 7.32 (d, J = 8.2 Hz, 4 H), 7.16 (d, J = 8.2 Hz, 4 H), 2.78–2.69 (m, 2 H), 1.33 (s, 18 H), 1.04–0.95 (m, 6 H); (minor) δ: 7.22 (d, J = 8.2 Hz, 4 H), 7.01 (d, J = 8.2 Hz, 4 H), 3.00–2.91 (m, 2 H), 1.29 (s, 18 H), 1.23–1.17 (m, 6 H) ppm.
^{13}C NMR ($CDCl_3$, 126 MHz) mixture of diastereomers (dr. ~ 1:2) δ: 148.8, 148.5, 143.7, 142.9, 127.6, 127.3, 125.2, 124.7, 47.0, 45.7, 34.5, 31.6, 31.6, 21.5, 16.0 ppm.
HRMS(+APCI) calc'd for $C_{20}H_{26}$ $[M-2H+H]^{+\bullet}$ 265.1951, found 265.1941.

Reactions with diazenes from α-primary amines and α-tertiary amines

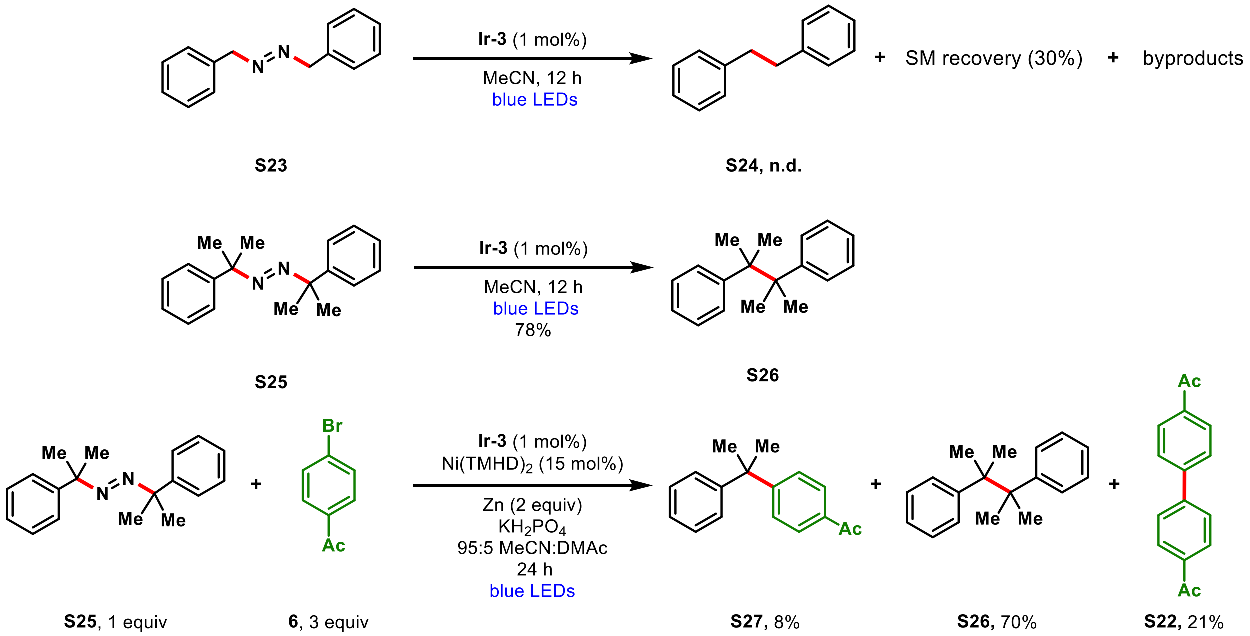

Figure S2. Efforts to extend the scope for diazenes derived from α-primary amines and α-tertiary amines. [Compounds **S23**[18] and **S25**[19] were prepared following the reported protocols]

Radical trapping with TEMPO

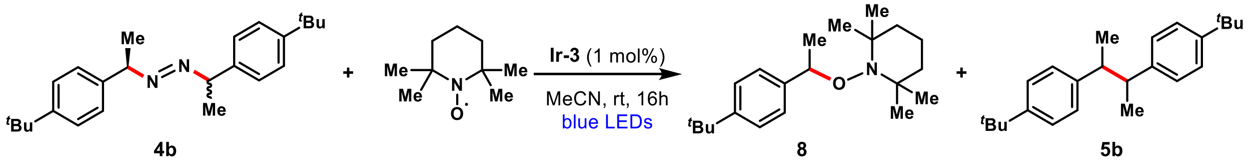

A flame-dried and argon-purged 3 mL vial equipped with a PTFE-coated stir bar was charged with **4b** (41 mg, 0.13 mmol, 1.0 equiv), TEMPO (39 mg, 0.25 mmol, 2.0 equiv), and photocatalyst **Ir-3** (1.4 mg, 1 mol%). The vial was then evacuated under high vacuum for 15 min and then backfilled with argon. Anhydrous degassed MeCN (0.6 mL) was added with a syringe under an argon atmosphere. The vial was then irradiated using blue LEDs (30 W, 450 nm), while the reaction was stirred at 600 rpm. After 24 hours of irradiation, the volatiles were removed under reduced pressure and the crude residues were purified by column chromatography (SiO_2, 70:30 DCM:hexanes) affording **8** as a transparent oil (18 mg, 22%).

R_f = 0.60 (70:30 DCM:hexanes)

^{1}H NMR (400 MHz, $CDCl_3$) δ 7.31 (d, *J* = 8.3 Hz, 2 H), 7.24 (d, *J* = 8.2 Hz, 2 H), 4.77 (q, *J* = 6.6 Hz, 1 H), 1.47 (d, *J* = 6.6 Hz, 3 H), 1.30–1.23 (m, 18 H), 1.16 (s, 3 H), 1.04 (s, 3 H), 0.70 (s, 3 H) ppm.

^{13}C NMR ($CDCl_3$, 101 MHz) δ: 149.7, 142.7, 126.4, 124.9, 82.6, 77.4, 40.5, 34.6, 31.6, 31.1, 23.4, 17.4 ppm.

HRMS(+ESI) calc'd for $C_{21}H_{35}NO$ $[M+H]^+$ 318.2791, found 318.2789.

Radical clock experiment

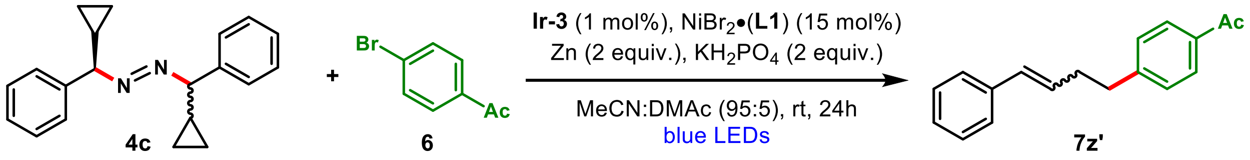

A flame-dried and argon-purged 3 mL vial equipped with a PTFE-coated stir bar was charged with **4c** (37 mg, 0.13 mmol, 1.0 equiv), 4-Bromoacetophenone (75 mg, 0.38 mmol, 3.0 equiv), photocatalyst **Ir-3** (1.4 mg, 1 mol%), $NiBr_2$**•(di-OMe-bpy)** (8.2 mg, 15 mol%), KH_2PO_4 (34 mg, 0.25 mmol, 2 equiv) and zinc powder (16.5 mg, 2.0 equiv). Upon addition of all solids, the vial was evacuated under high vacuum for 15 min and then backfilled with argon. Anhydrous degassed MeCN (0.57 mL) was added with a syringe under an argon atmosphere followed by anhydrous degassed DMAc (0.03 mL) with a micro-syringe. The vial was then irradiated using blue LEDs (30 W, 450 nm), while the reaction was stirred at 600 rpm. After 24 hours of irradiation, the reaction mixture was diluted with EtOAc (5 mL) and filtered through a silica plug (~ 2.0 cm), which was subsequently flushed with additional EtOAc (50 mL). The volatiles were removed under reduced pressure and the crude residues were purified by column chromatography (SiO_2, 2:98 Et_2O:hexanes) afforded **7z'** as a transparent oil and as a mixture of *E*/*Z* stereoisomers (40 mg, 63%).

R_f = 0.30 (10:90 Et_2O:hexanes)

^{1}H NMR ($CDCl_3$, 400 MHz) mixture of stereoisomers (2:1) δ: (major): 7.89 (m, 2 H), 7.37–7.27 (m, 4 H), 7.28–7.18 (m, 3 H), 6.46 (d, *J* = 11.7 Hz, 1 H), 5.67 (dt, *J* = 11.6, 7.1 Hz, 1 H), 2.90–

2.78 (m, 2 H), 2.73–2.62 (m, 1 H), 2.59 (m, 4 H); (minor): 7.89 (m, 2 H), 7.37–7.27 (m, 4 H), 7.28–7.18 (m, 3 H), 6.41 (d, J = 15.8 Hz, 1 H), 6.22 (dt, J = 15.8, 6.8 Hz, 1 H), 2.90–2.78 (m, 2 H), 2.73–2.62 (m, 1 H), 2.59 (m, 4 H) ppm.

^{13}C NMR ($CDCl_3$, 101 MHz) mixture of stereoisomers δ: 198.0, 198.0, 147.7, 147.6, 137.6, 137.5, 135.3, 131.2, 131.0, 130.0, 129.3, 128.9, 128.8, 128.8, 128.7, 128.7, 128.6, 128.3, 127.2, 126.8, 126.1, 77.5, 77.2, 76.8, 36.2, 36.0, 34.5, 30.0, 29.9, 26.7 ppm.

HRMS(+APCI) calc'd for $C_{18}H_{18}O$ $[M+H]^+$ 251.1430, found 251.1417.

Coupling with stoichiometric Ni(II)

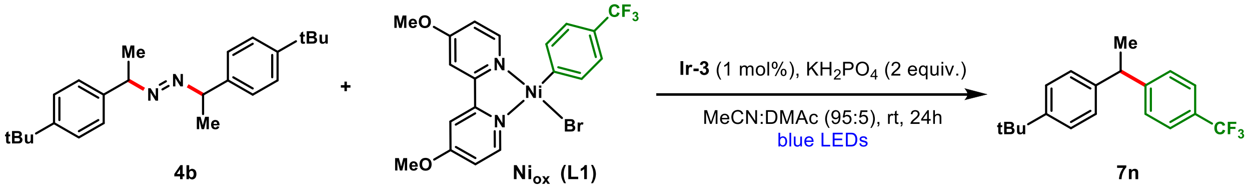

A flame-dried and argon-purged 3 mL vial equipped with a PTFE-coated stir bar was charged with **4b** (37 mg, 0.13 mmol, 1 equiv), photocatalyst **Ir-3** (1.4 mg, 1 mol%) and KH_2PO_4 (34 mg, 0.25 mmol, 2 equiv). The vial was subsequently transferred into a N_2-filled glovebox and **Ni$_{ox}$•(L1)**[6] (57.5 mg, 0.13 mmol, 1 equiv) was added. The vial was then removed from the glovebox and anhydrous degassed MeCN (0.57 mL) was added with a syringe under an argon atmosphere followed by anhydrous degassed DMAc (0.03 mL) with a micro-syringe. The vial was then irradiated using blue LEDs (30 W, 450 nm) while the reaction was stirred at 600 rpm. After 24 hours of irradiation, the reaction mixture was diluted with EtOAc (5 mL) and filtered through a silica plug (~ 2.0 cm), which was subsequently flushed with additional EtOAc (50 mL). The volatiles were removed under reduced pressure and the yield was determined by ^{1}H NMR spectroscopy (400 MHz, $CDCl_3$) using trimethyl(phenyl)silane as an internal standard.

Photoluminescence quenching experiments

A stock solution of photocatalyst (**Ir-1–Ir-4**) was prepared in a mixture of MeCN:DMAc (95:5) at a concentration corresponding to an absorbance of ~ 0.1 at the excitation wavelength (see Figure S3). A solution of diazene (*S*,*S*)-**4a** (C = 0.1 M) was prepared using the stock solution of **Ir-1–Ir-4** in MeCN:DMAc (95:5). Increasing portion of the solution of **4a** and **Ir-1–Ir-4** in MeCN:DMAc

(95:5) was added to the initial stock solution of photocatalyst and the photoluminescence was monitored after each addition (Figure S3). The quenching of photoluminescence caused by (*S*,*S*)-**4a** was found to follow the Stern-Volmer equation below (Equation S1).

$$\frac{I_0}{I} = 1 + K_{SV}[Q] \quad \text{Equation S1}$$

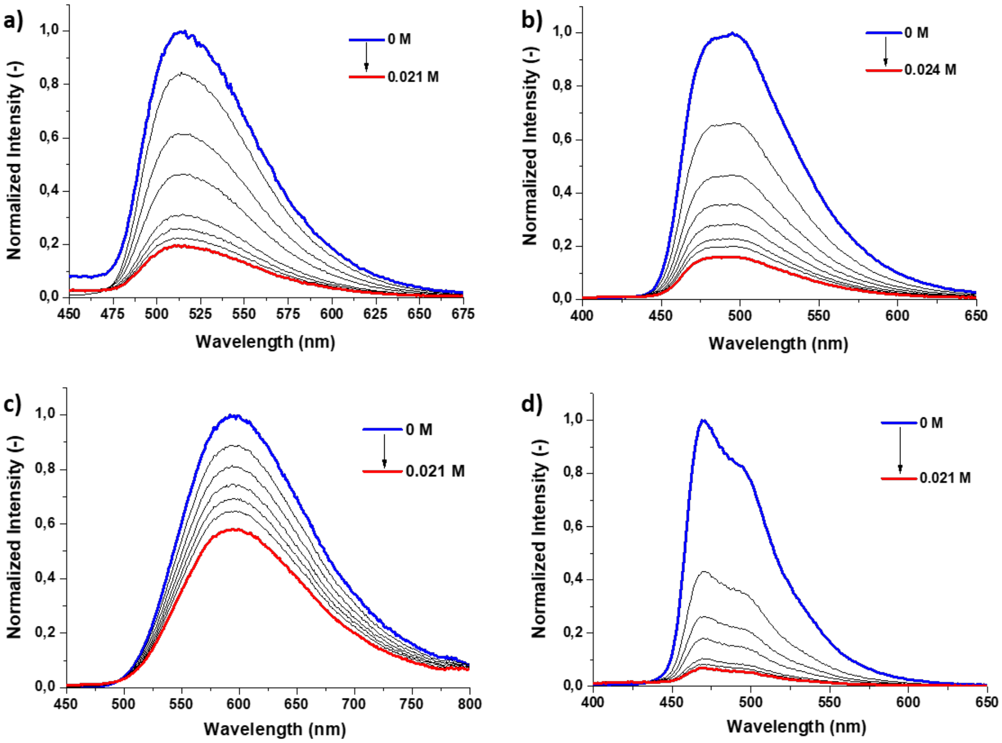

Figure S3. Photoluminescence of **Ir-1** (a, λ_{exc} = 400 nm), **Ir-2** (b, λ_{exc} = 350 nm), **Ir-3** (c, λ_{exc} = 400 nm) and **Ir-4** (d, λ_{exc} = 350 nm) in the presence of different concentrations of diazene **4a** in argon-purged MeCN:DMAc (95:5) mixture.

References:

(1) Kulow, R. W.; Wu, J. W.; Kim, C.; Michaudel, Q. Synthesis of Unsymmetrical Sulfamides and Polysulfamides via SuFEx Click Chemistry. *Chem. Sci.* **2020**, *11*, 7807–7812.

(2) Miriyala, B.; Bhattacharyya, S.; Williamson, J. S. Chemoselective Reductive Alkylation of Ammonia with Carbonyl Compounds: Synthesis of Primary and Symmetrical Secondary Amines. *Tetrahedron* **2004**, *60*, 1463–1471.

(3) Courtemanche, G.; Gautier, C.; Gully, D.; Roger, P.; Valette, G.; Wermuth C. G. Branched Alkylamino Derivatives of Thiazole, Processes for Preparing Them and Pharmaceutical Compositions Containing Them. Patent US5464847A, November 7, 1995.

(4) Bera, S.; Mao, R.; Hu, X. Enantioselective C(sp^3)–C(sp^3) Cross-coupling of Non-Activated Alkyl Electrophiles via Nickel Hydride Catalysis. *Nat. Chem.* **2021**, *13*, 270–277.

(5) Lux, M. C.; Boby, M. L.; Brooks, J. L.; Tan, D. S. Synthesis of Bicyclic Ethers by a Palladium-catalyzed Oxidative Cyclization-Redox Relay-π-Allyl-Pd Cyclization Cascade Reaction. *Chem. Commun.* **2019**, *55*, 7013.

(6) Sun, S.-Z.; Martin, R. Nickel-Catalyzed Umpolung Arylation of Ambiphilic α-Bromoalkyl Boronic Esters. *Angew. Chem. Int. Ed.* **2018**, *57*, 3622–3625.

(7) Tamayo, A. B.; Alleyne, B. D.; Djurovich, P. I.; Lamansky, S.; Tsyba, I.; Ho, N. N.; Bau, R.; Thompson, M. E. Synthesis and Characterization of Facial and Meridional Tris-Cyclometalated Iridium(III) Complexes. *J. Am. Chem. Soc.* **2003**, *125*, 7377–7387.

(8) Choi, G. J.; Zhu, Q.; Miller, D. C.; Gu, C. J.; Knowles, R. R. Catalytic Alkylation of Remote C–H Bonds Enabled by Proton-coupled Electron Transfer. *Nature* **2016**, *539*, 268–271.

(9) Kalvet, I.; Sperger, T.; Scattolin, T.; Magnin, G.; Schoenebeck, F. Palladium(I) Dimer Enabled Extremely Rapid and Chemoselective Alkylation of Aryl Bromides over Triflates and Chlorides in Air. *Angew. Chem. Int. Ed.* **2017**, *56*, 7078–7082.

(10) Chotana, G. A.; Rak, M. A.; Smith, M. R. Sterically Directed Functionalization of Aromatic C–H Bonds: Selective Borylation Ortho to Cyano Groups in Arenes and Heterocycles. *J. Am. Chem. Soc.* **2005**, *127*, 10539–10544.

(11) Hawkins, J. M.; Sharpless, K. B. Threo-N,N′-Bis(alpha-methylbenzyl)sulfamide: a Readily Available Chiral Ligand for Asymmetric Lithium Aluminum Hydride Reductions. *J. Org. Chem.* **1984**, *49*, 3861–3862.

(12) Grizzle, P. L.; Miller, D. W.; Scheppele, S. E. Convenient Synthesis of Protiated and Specifically Deuterated Secondary Azoalkanes. *J. Org. Chem.* **1975**, *40*, 1902–1906.

(13) Hu, L.; Liu, Y.; Fang, X.; Zheng, Y.; Liao, R.-z.; Li, M.; Xie, Y. An Intermolecular Hydroarylation of Highly Deactivated Styrenes Catalyzed by $Re_2O_7/HReO_4$ in Hexafluoroisopropanol. *ACS Catal.* **2022**, *12*, 5857–5863.

(14) Xiao, L.-J.; Cheng, L.; Feng, W.-M.; Li, M.-L.; Xie, J.-H.; Zhou, Q.-L. Nickel(0)-Catalyzed Hydroarylation of Styrenes and 1,3-Dienes with Organoboron Compounds. *Angew. Chem. Int. Ed.* **2018**, *57*, 461–464.

(15) Caldwell, R. A.; Diaz, J. F.; Hrncir, D. C.; Unett, D. J. Alkene Triplets as 1,2-Biradicals: The Photoaddition of *p*-Acetylstyrene to Styrene. *J. Am. Chem. Soc.* **1994**, *116*, 8138–8145.

(16) Su, X.-L.; Jiang, S.-P.; Ye, L.; Xu, G.-X.; Chen, J.-J.; Gu, Q.-S.; Li, Z.-L.; Liu, X.-Y. A General Copper-catalyzed Radical $C(sp^3)–C(sp^2)$ Cross-Coupling to Access 1,1-Diarylalkanes under Ambient Conditions. *Tetrahedron* **2021**, *89*, 132152.

(17) Ohkura, R.; Ohtsuka, M.; Yim, J. C. H.; Nambo, M.; Crudden, C. M. Photocatalytic Desulfonylative Homocoupling of Benzylic Sulfone Derivatives. *Synlett* **2023**, *34*, 81–85.

(18) Squillacote, M.; De Felippis, J. 1-Thia-3,4-diazolidine-2,5-dione Functionality : A Photochemical Synthon for the Azo Group. *J. Org. Chem.* **1994**, *59*, 3564–3571.

(19) Leffler, J. E.; Zupancic, J. J. Decomposition of Azocumene on Silica Surfaces. *J. Am. Chem. Soc.* **1980**, *102*, 259–267.

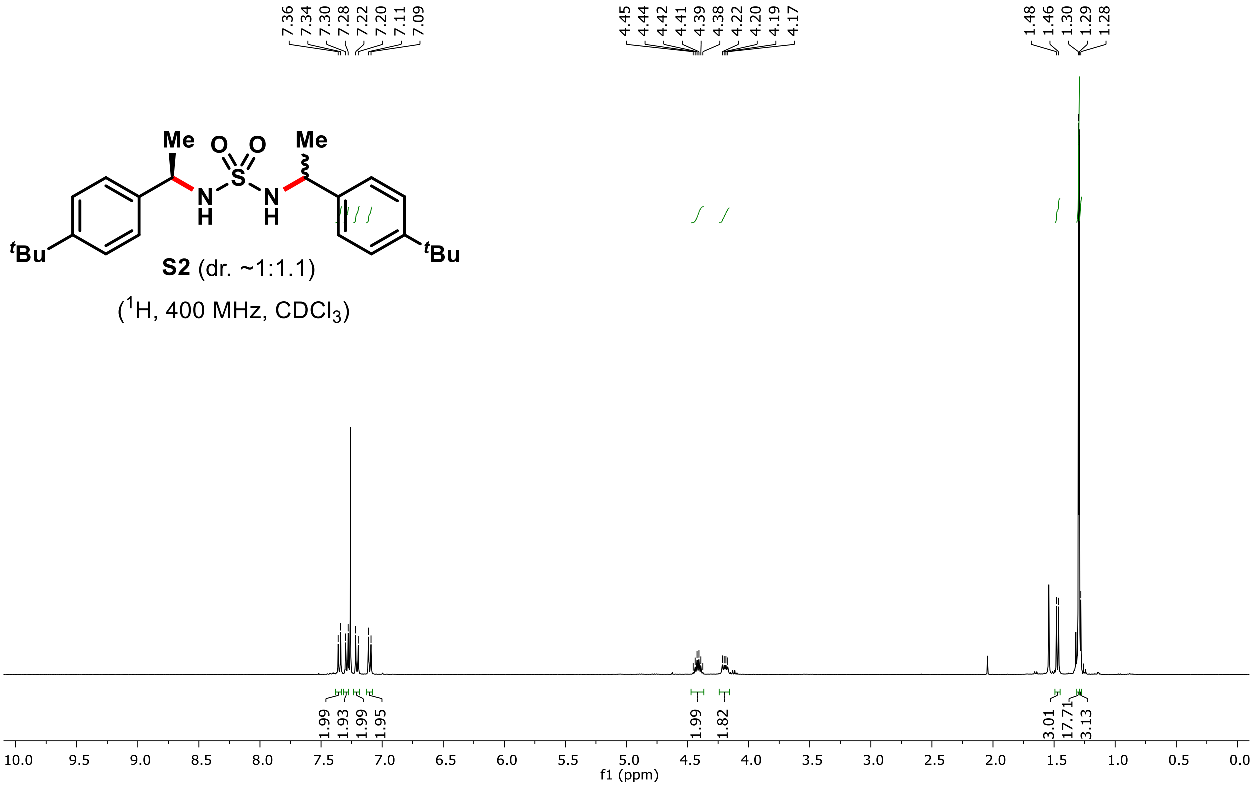

S2 (dr. ~1:1.1)

(^{1}H, 400 MHz, $CDCl_3$)

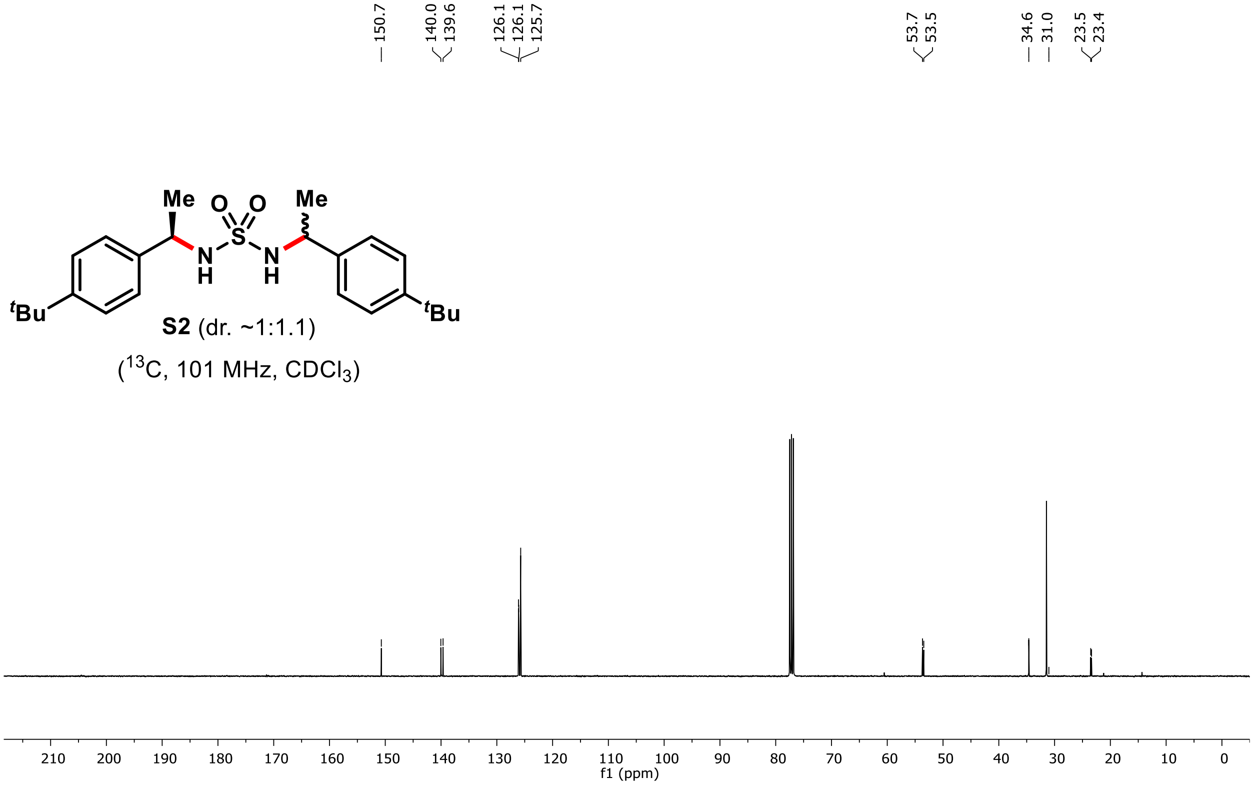

S2 (dr. ~1:1.1)

(^{13}C, 101 MHz, $CDCl_3$)

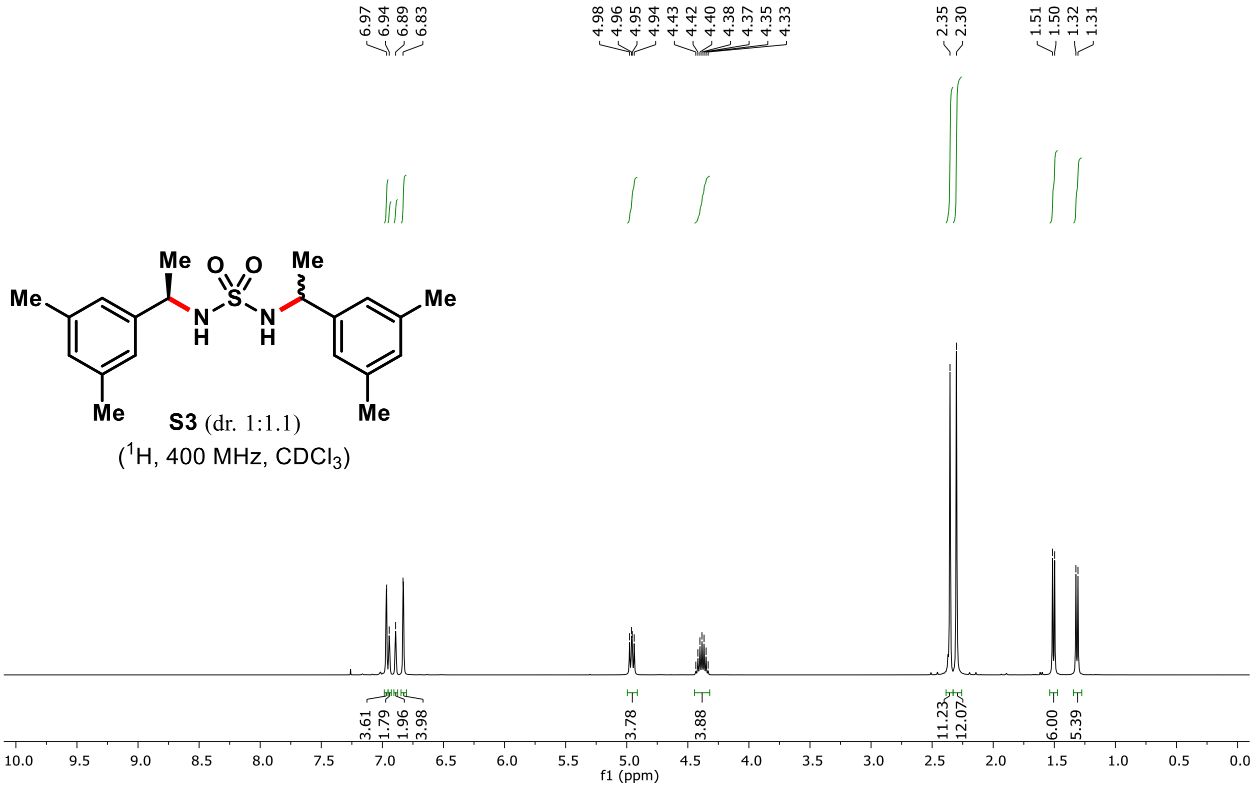
6.97
6.94
6.89
6.83
4.98
4.96
4.95
4.94
4.43
4.42
4.40
4.38
4.37
4.35
4.33
2.35
2.30
1.51
1.50
1.32
1.31
S3 (dr. 1:1.1)
(1H, 400 MHz, CDCl3)
3.61
1.79
1.96
3.98
3.78
3.88
11.23
12.07
6.00
5.39
f1 (ppm)

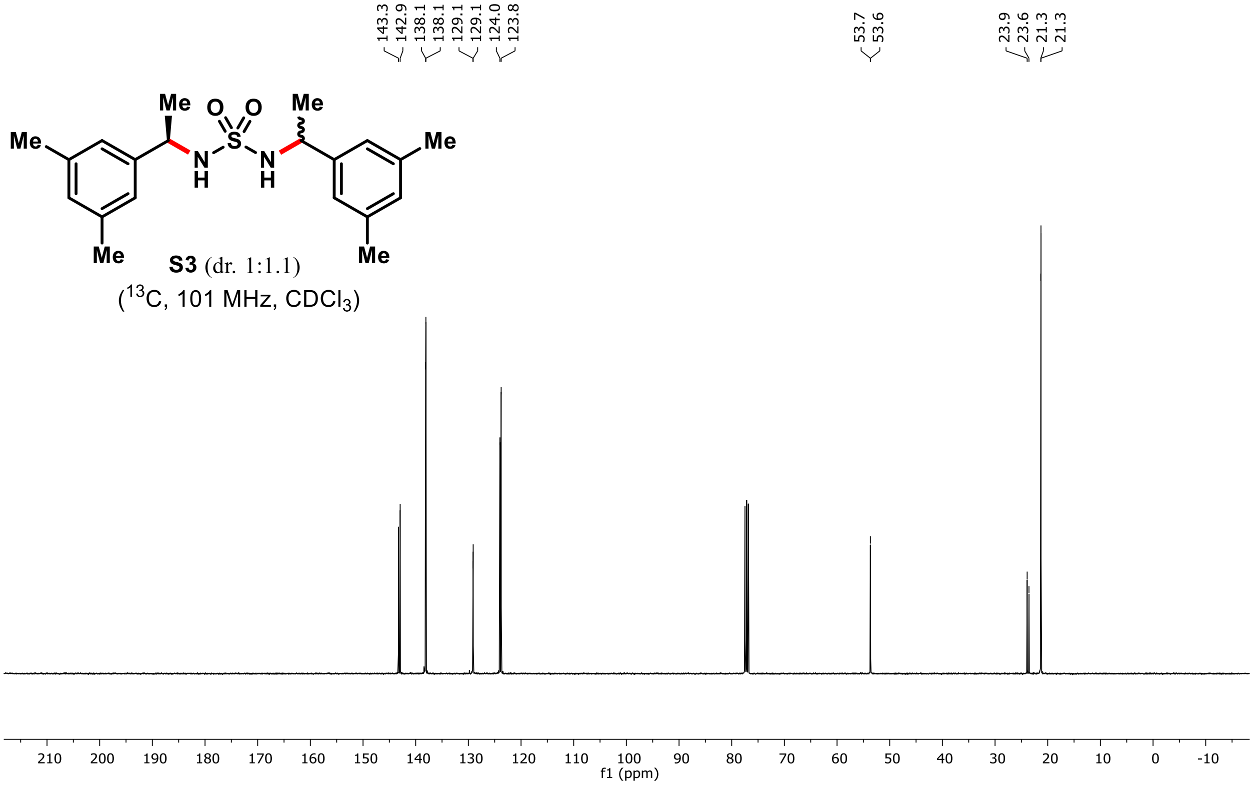
143.3
142.9
138.1
138.1
129.1
129.1
124.0
123.8
53.7
53.6
23.9
23.6
21.3
21.3
S3 (dr. 1:1.1)
(13C, 101 MHz, CDCl3)
f1 (ppm)

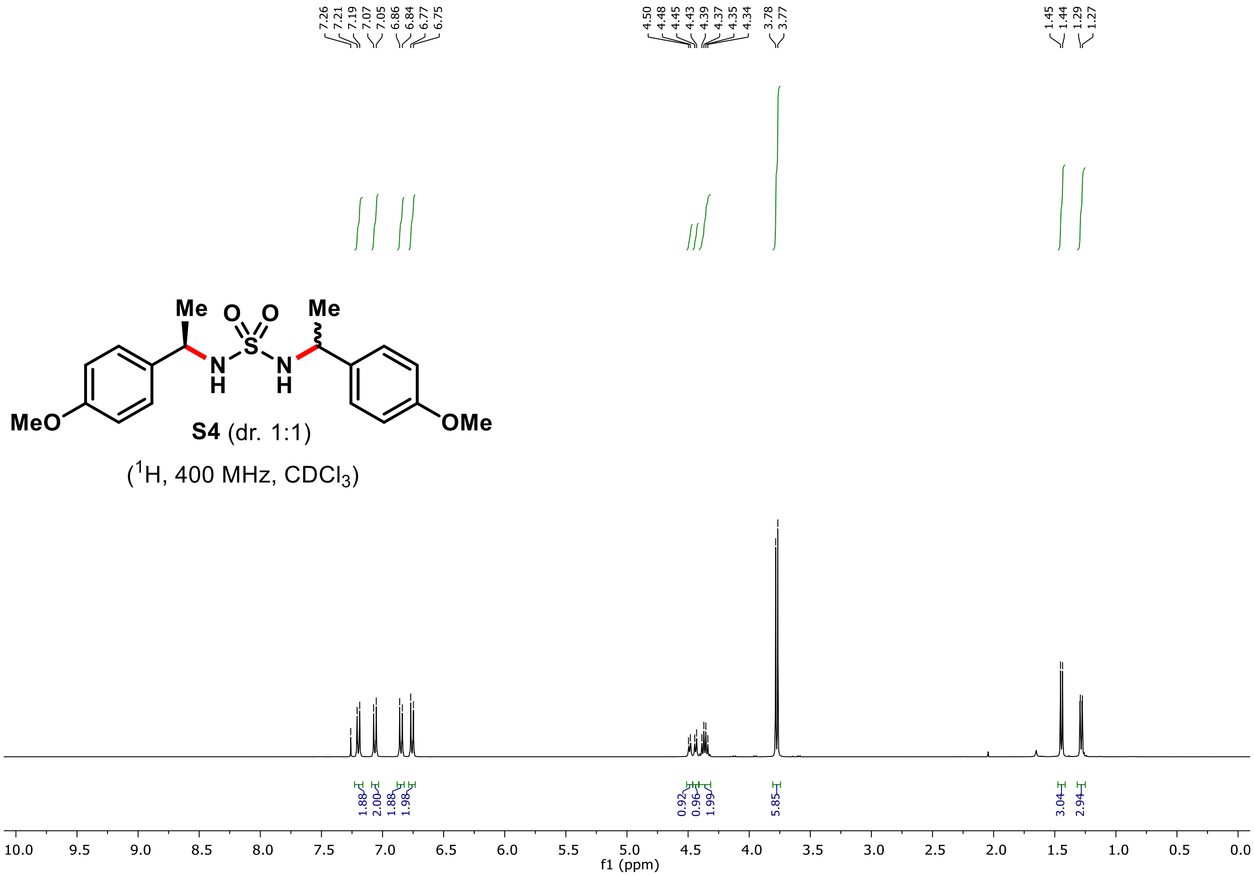

S4 (dr. 1:1)

(^{1}H, 400 MHz, $CDCl_3$)

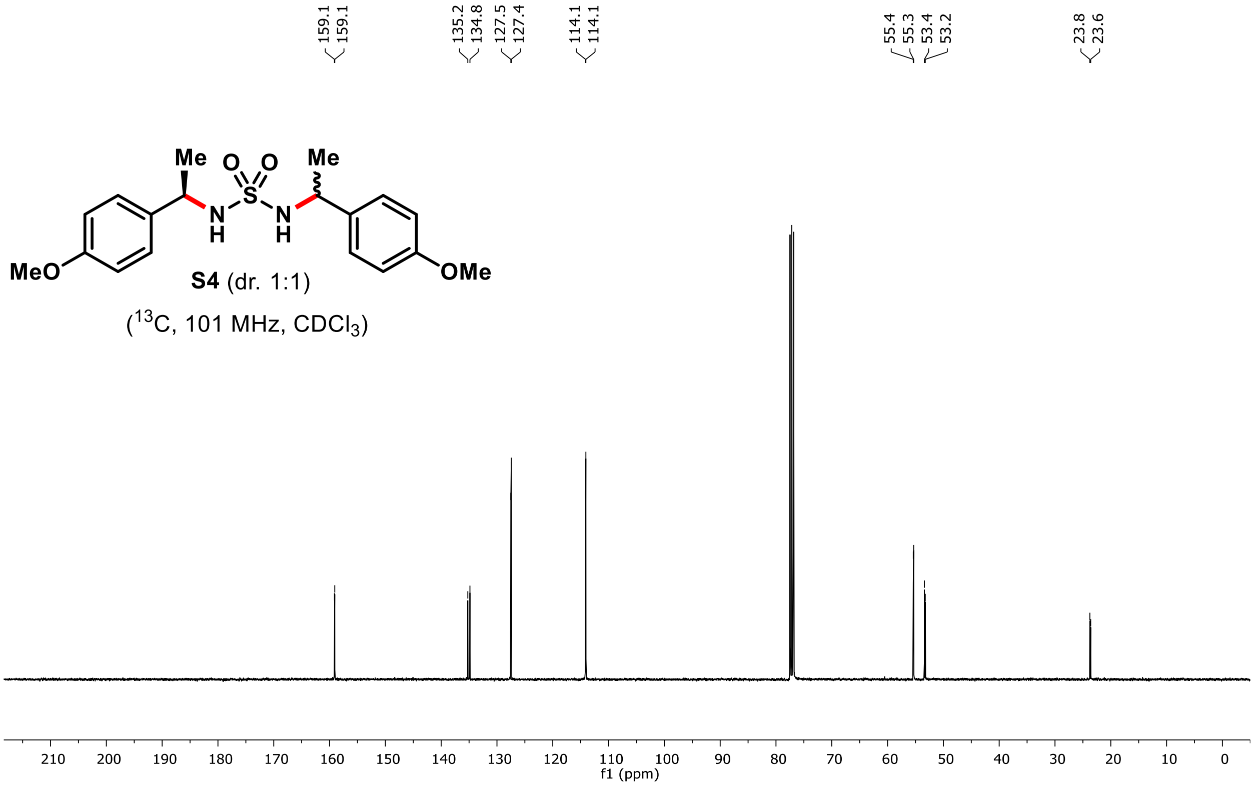

S4 (dr. 1:1)

(^{13}C, 101 MHz, $CDCl_3$)

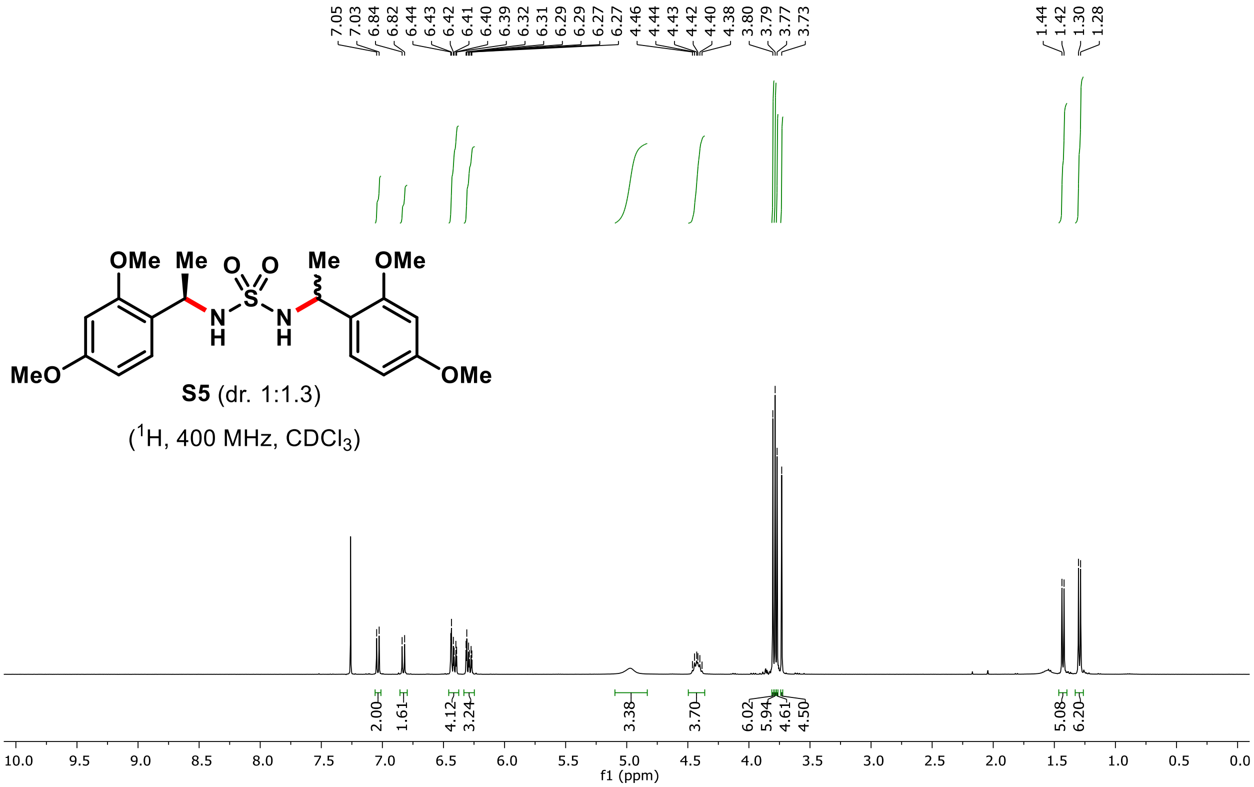
7.05 7.03 6.84 6.82 6.44 6.43 6.42 6.41 6.40 6.39 6.32 6.31 6.29 6.29 6.27 6.27 4.46 4.44 4.43 4.42 4.40 4.38 3.80 3.79 3.77 3.73 1.44 1.42 1.30 1.28
OMe Me O O Me OMe
MeO N H S N H OMe
S5 (dr. 1:1.3)
(^{1}H, 400 MHz, CDCl$_3$)
2.00 1.61 4.12 3.24 3.38 3.70 6.02 5.94 4.61 4.50 5.08 6.20
f1 (ppm)

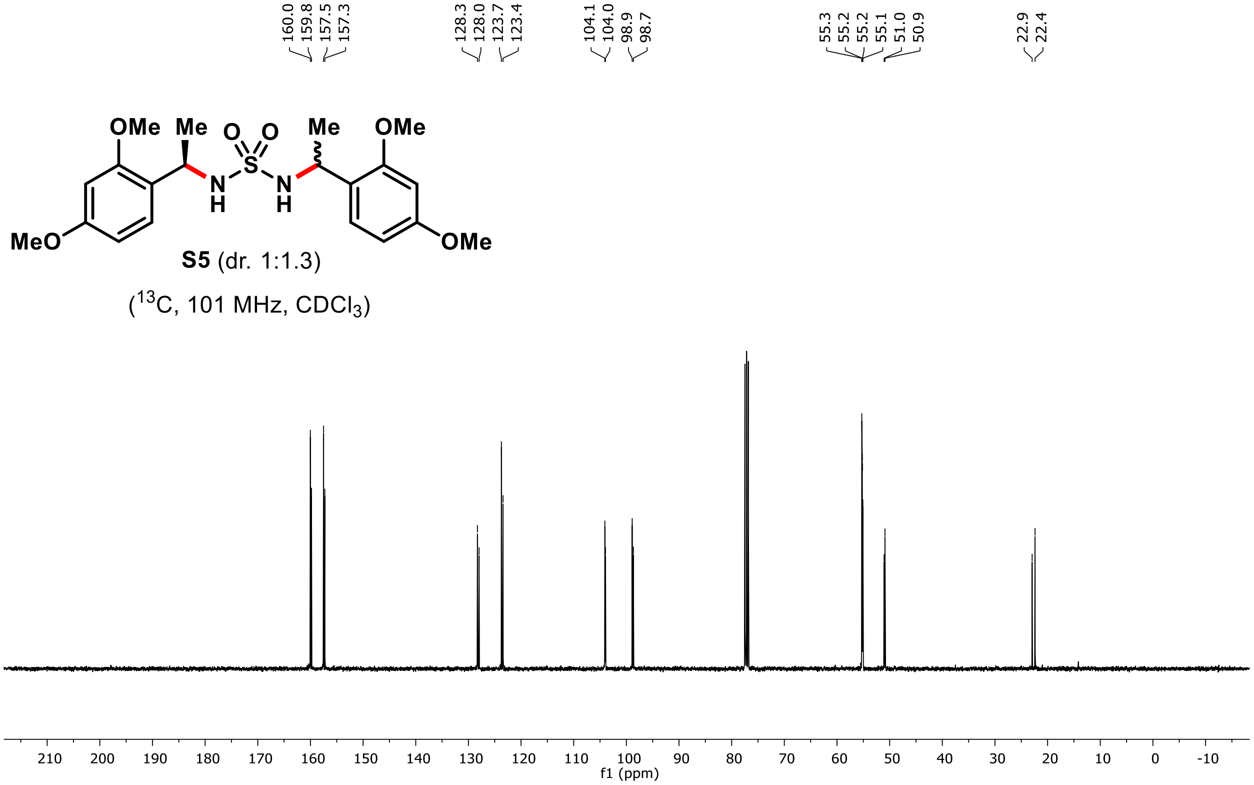
160.0 159.8 157.5 157.3 128.3 128.0 123.7 123.4 104.1 104.0 98.9 98.7 55.3 55.2 55.2 55.1 51.0 50.9 22.9 22.4
OMe Me O O Me OMe
MeO N H S N H OMe
S5 (dr. 1:1.3)
(^{13}C, 101 MHz, CDCl$_3$)
f1 (ppm)

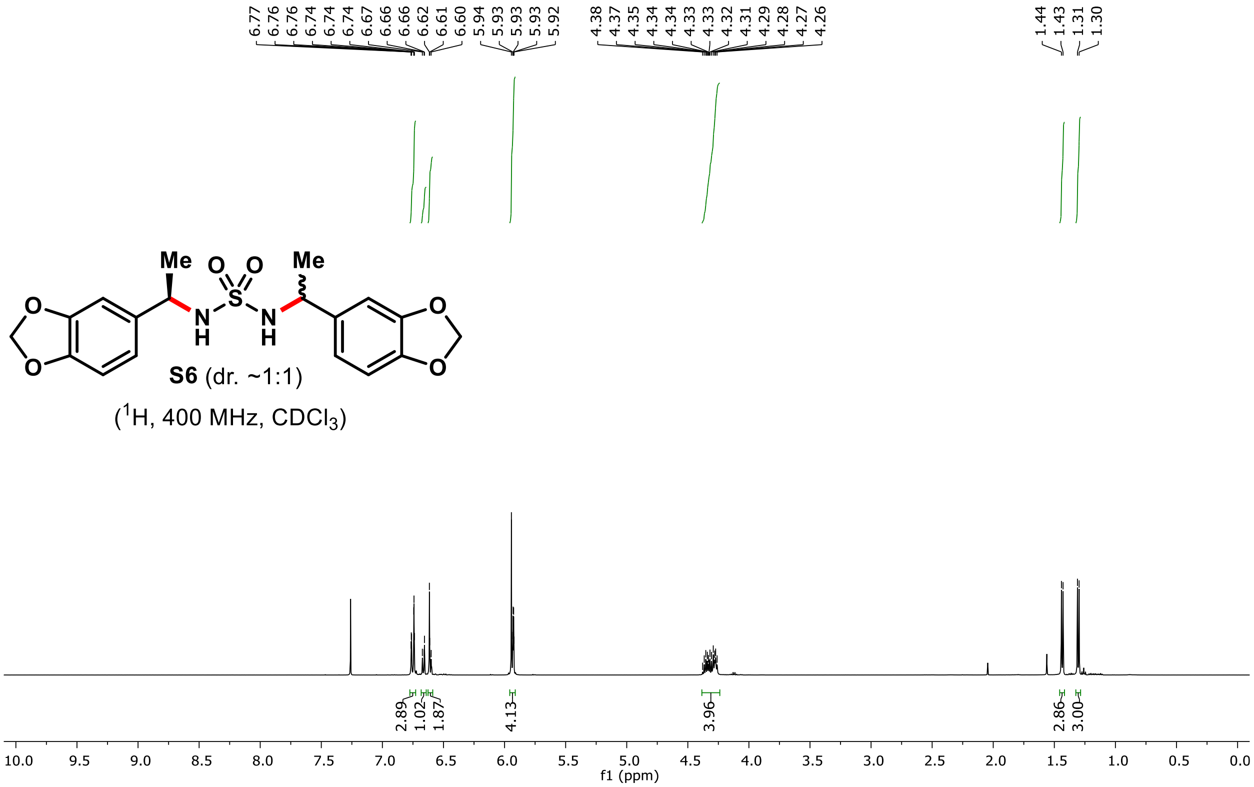
S6 (dr. ~1:1)
(^{1}H, 400 MHz, $CDCl_3$)

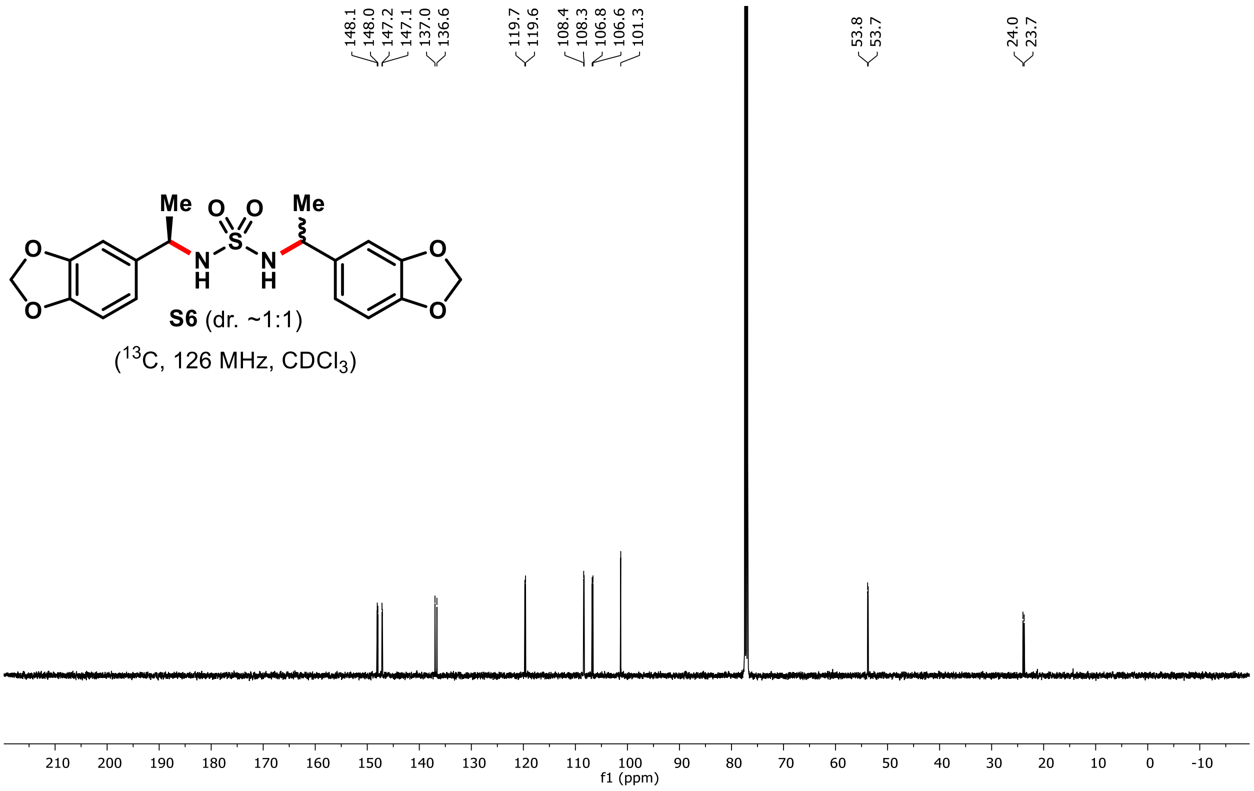
S6 (dr. ~1:1)
(^{13}C, 126 MHz, $CDCl_3$)

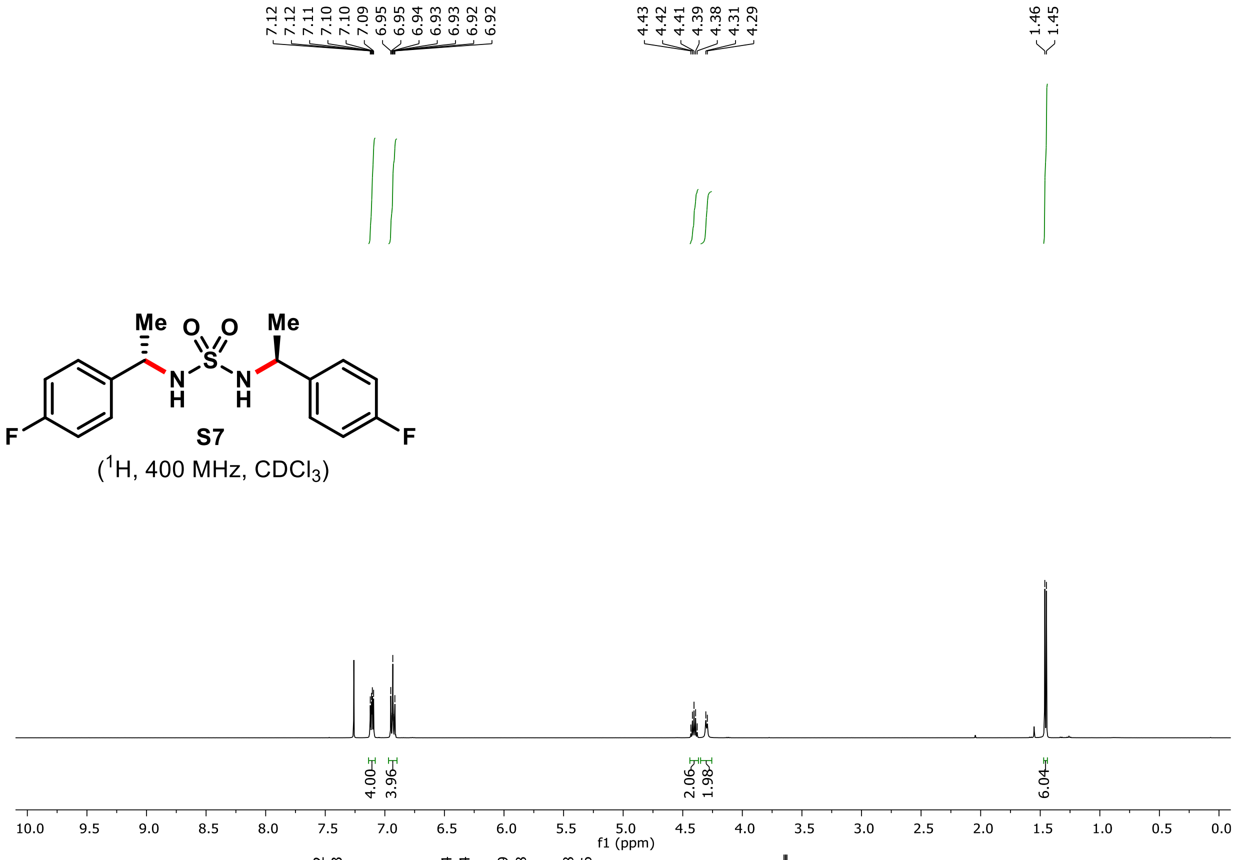
7.12
7.12
7.11
7.10
7.10
7.09
6.95
6.95
6.94
6.93
6.93
6.92
6.92
4.43
4.42
4.41
4.39
4.38
4.31
4.29
1.46
1.45
Me
O
O
Me
S
N
H
N
H
F
F
S7
(1H, 400 MHz, CDCl3)
4.00
3.96
2.06
1.98
6.04
f1 (ppm)

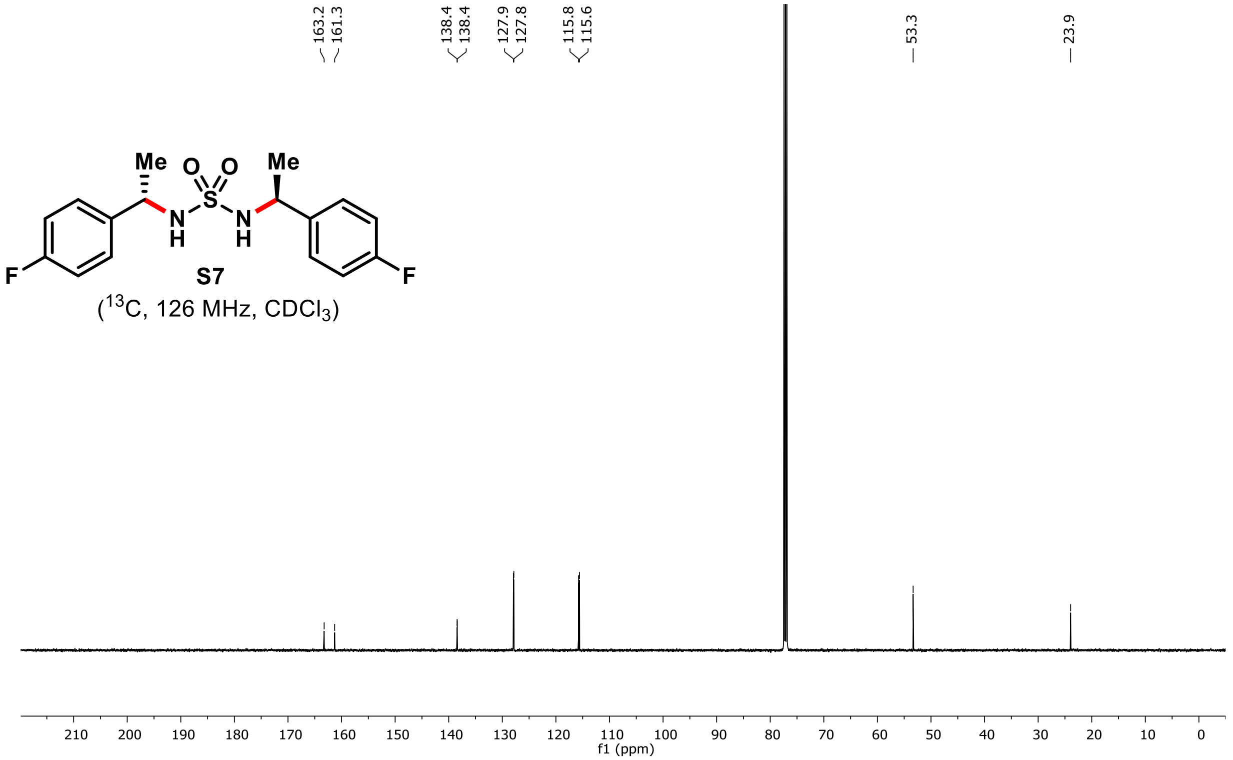
163.2
161.3
138.4
138.4
127.9
127.8
115.8
115.6
53.3
23.9
S7
(13C, 126 MHz, CDCl3)
f1 (ppm)

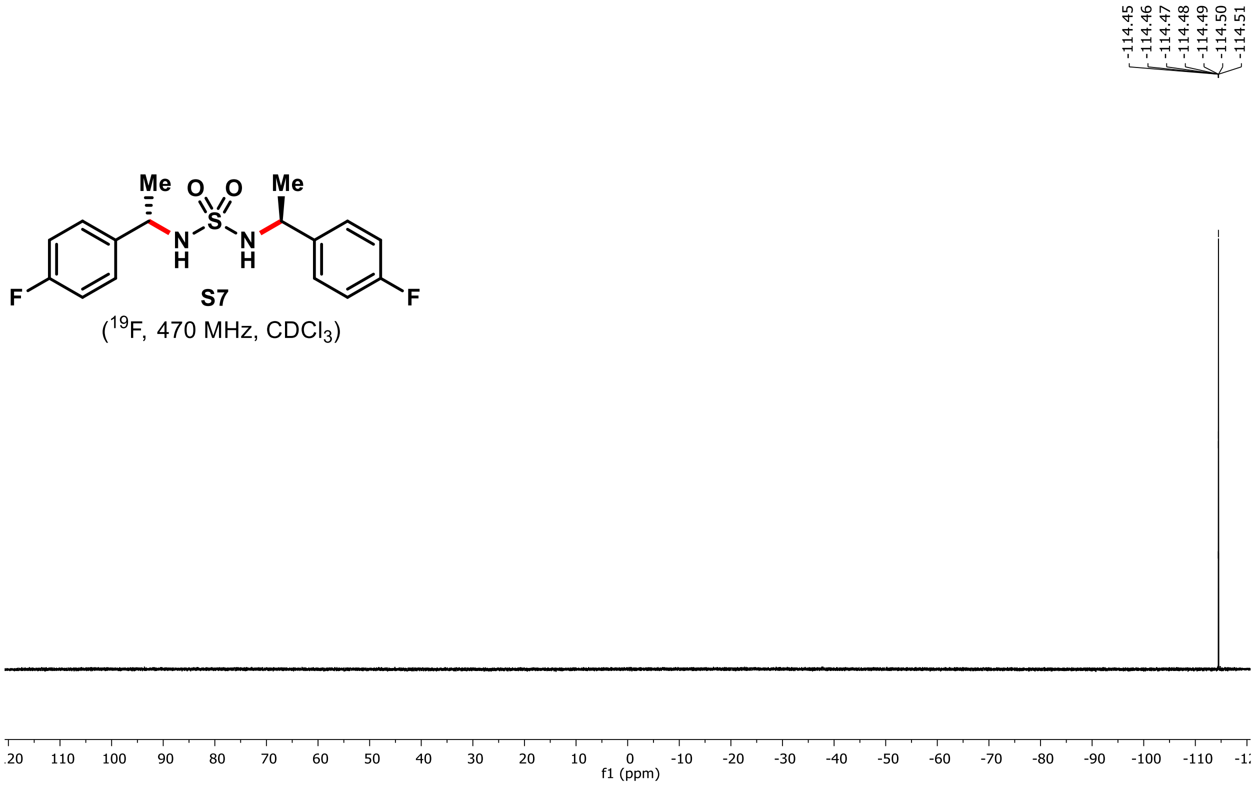
S7
(^{19}F, 470 MHz, $CDCl_3$)

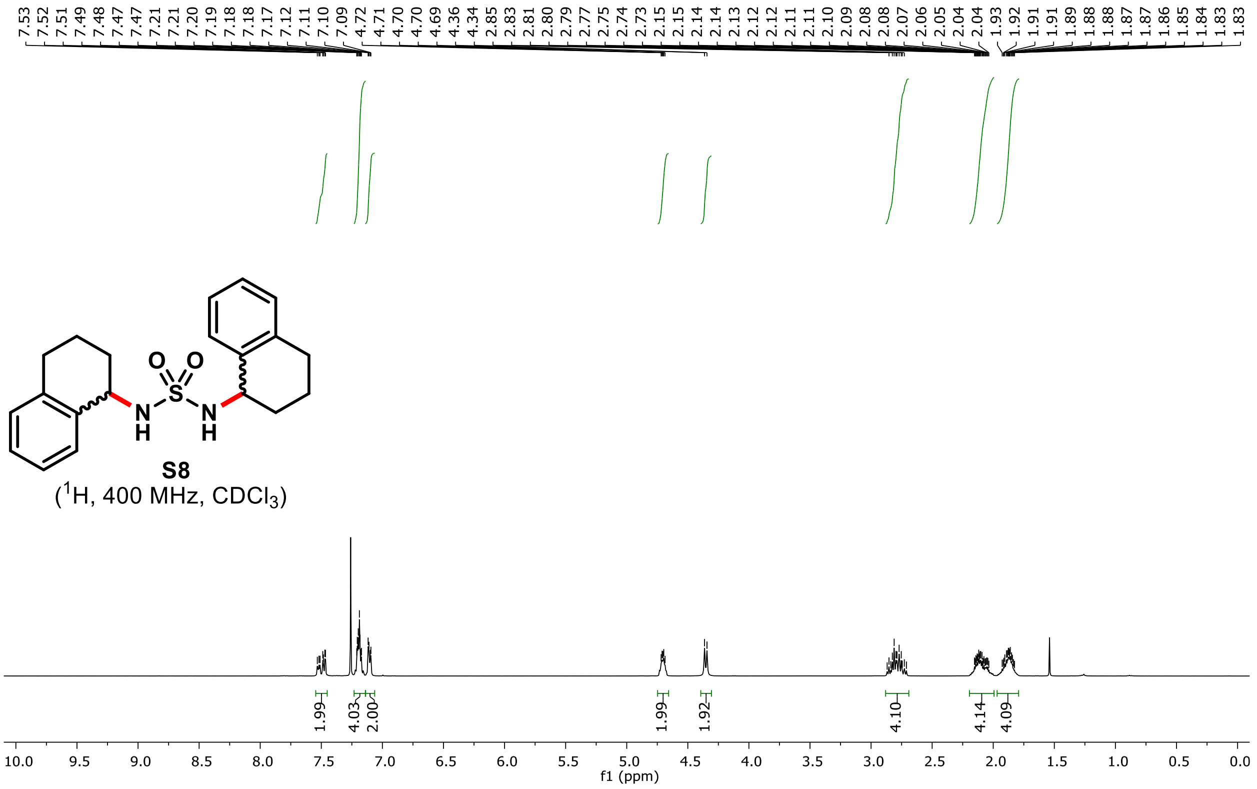
S8
(^{1}H, 400 MHz, $CDCl_3$)

S8
(^{13}C, 126 MHz, $CDCl_3$)

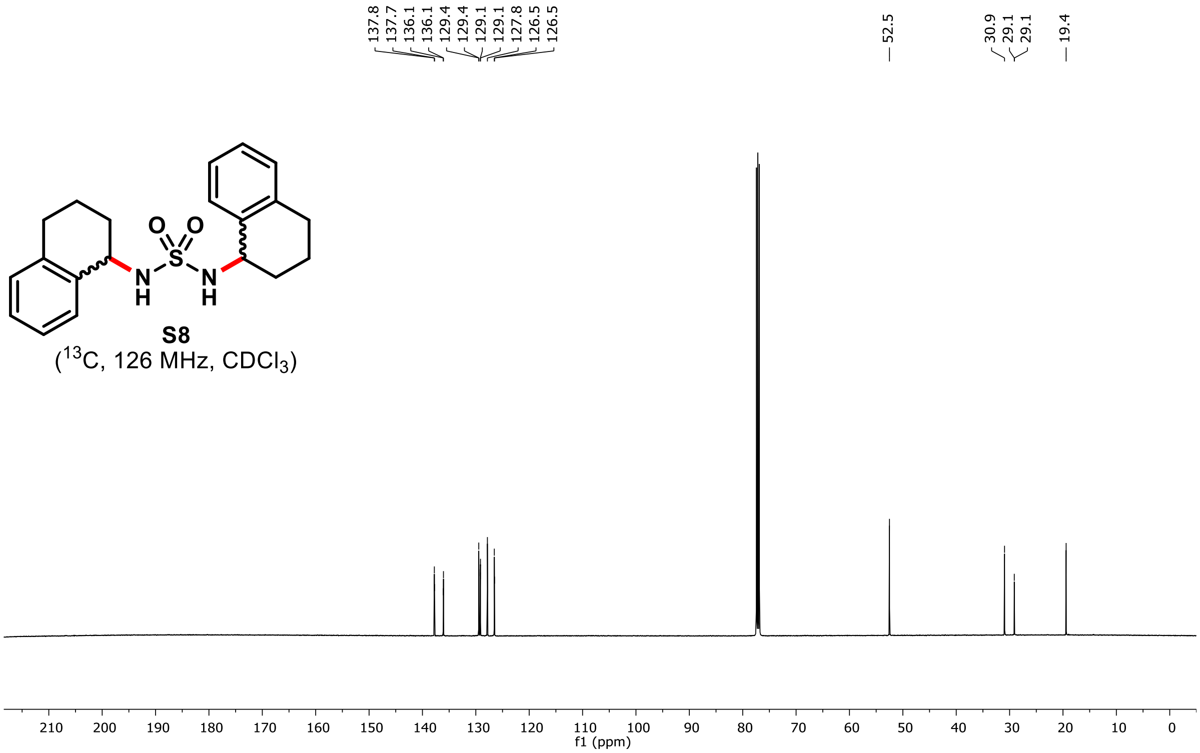

S9 (dr. 1:1)
(^{1}H, 400 MHz, $CDCl_3$)

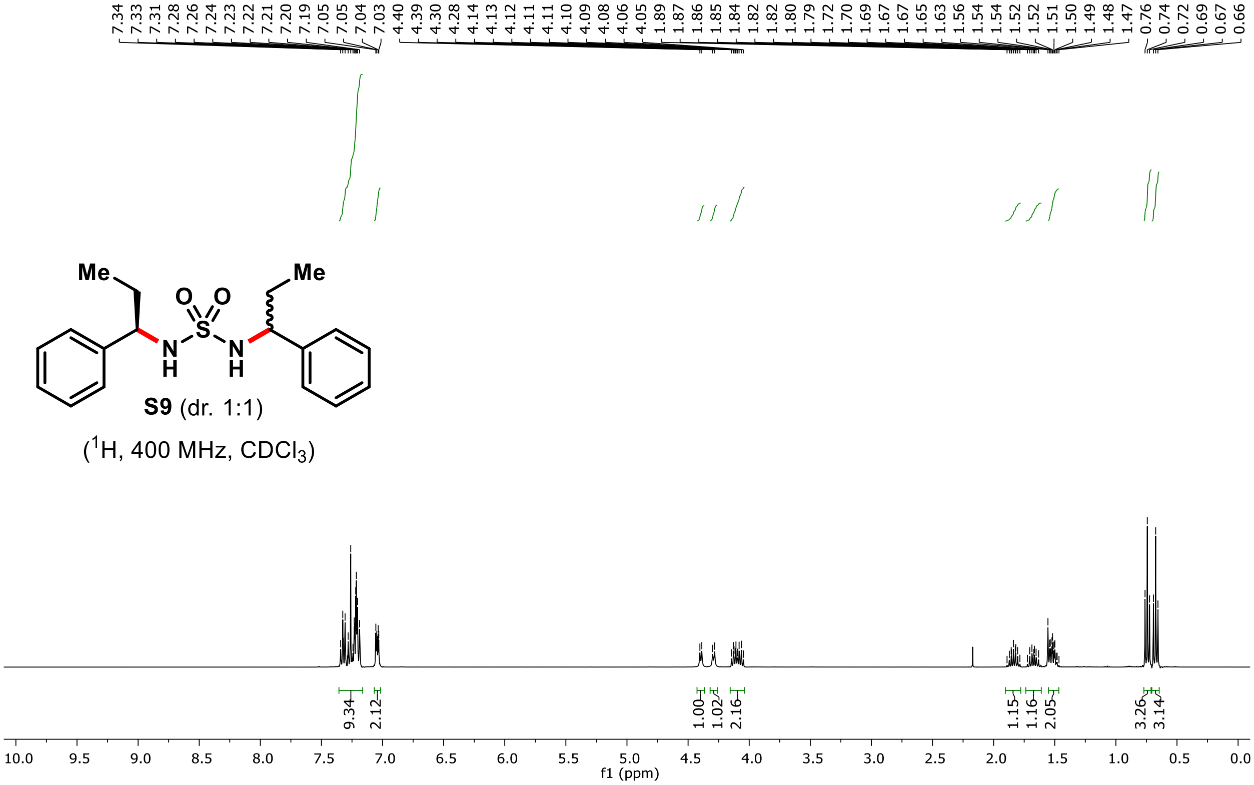

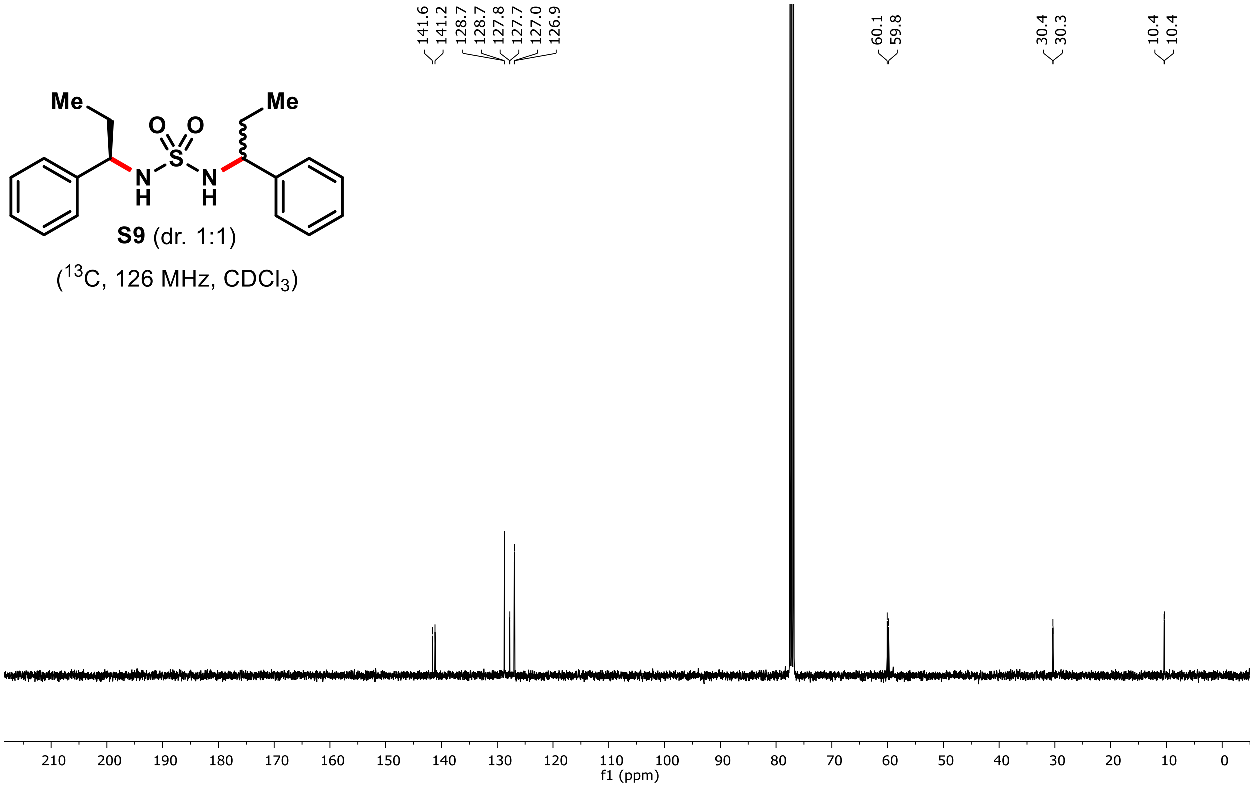

S9 (dr. 1:1)

(^{13}C, 126 MHz, $CDCl_3$)

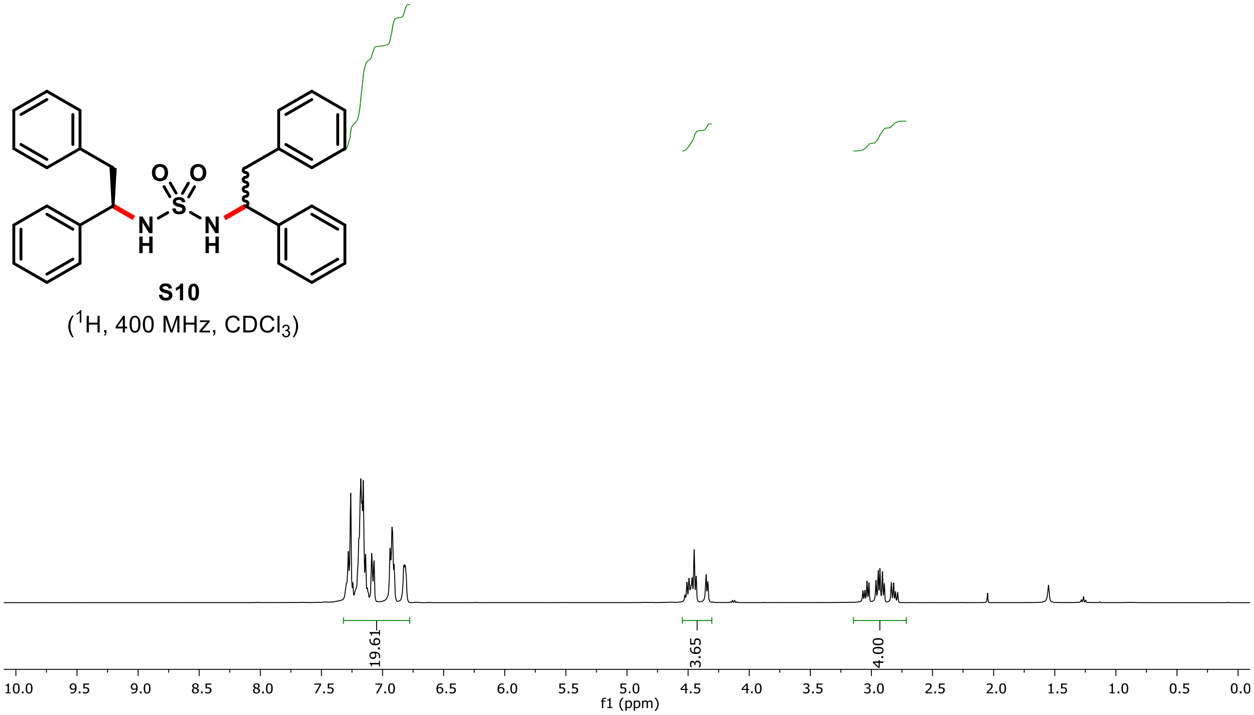

S10

(^{1}H, 400 MHz, $CDCl_3$)

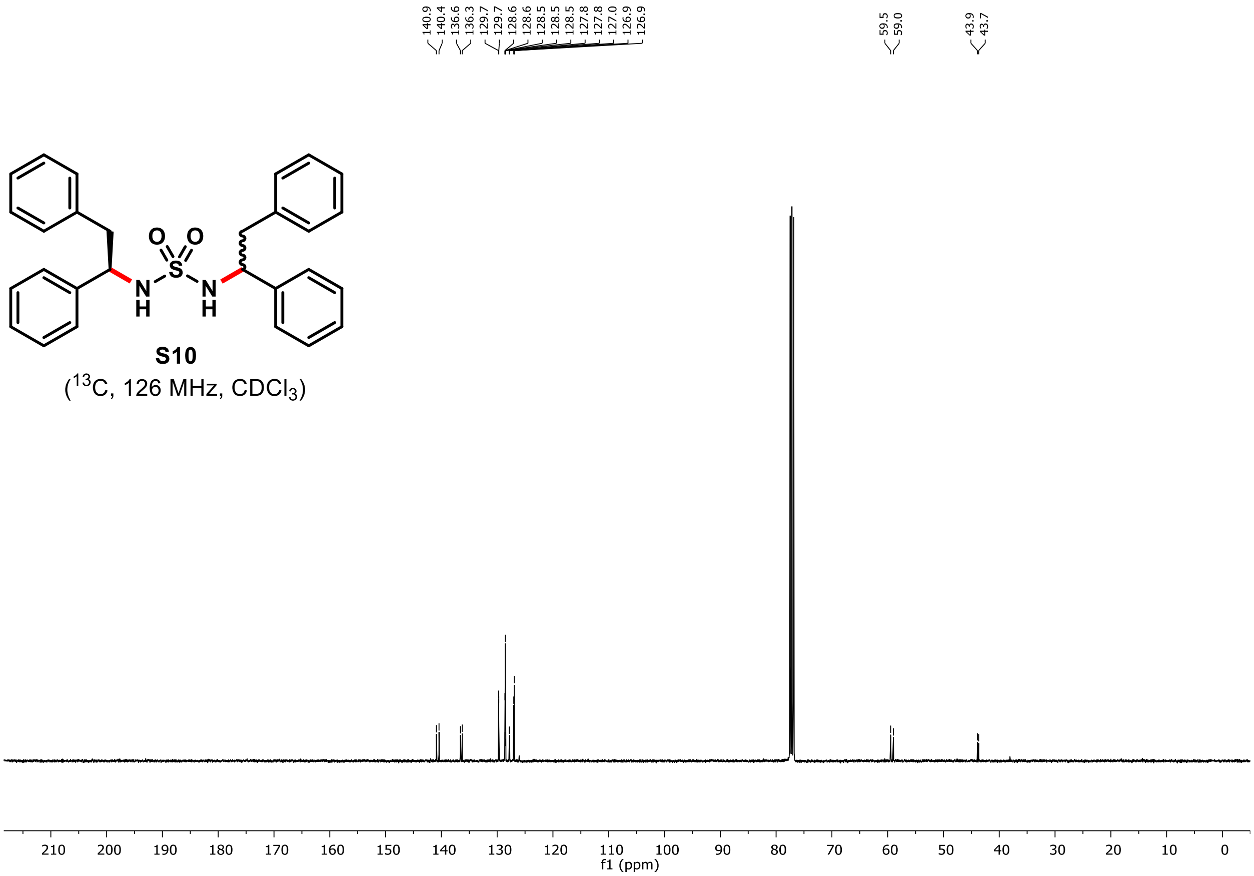

S10
(^{13}C, 126 MHz, $CDCl_3$)

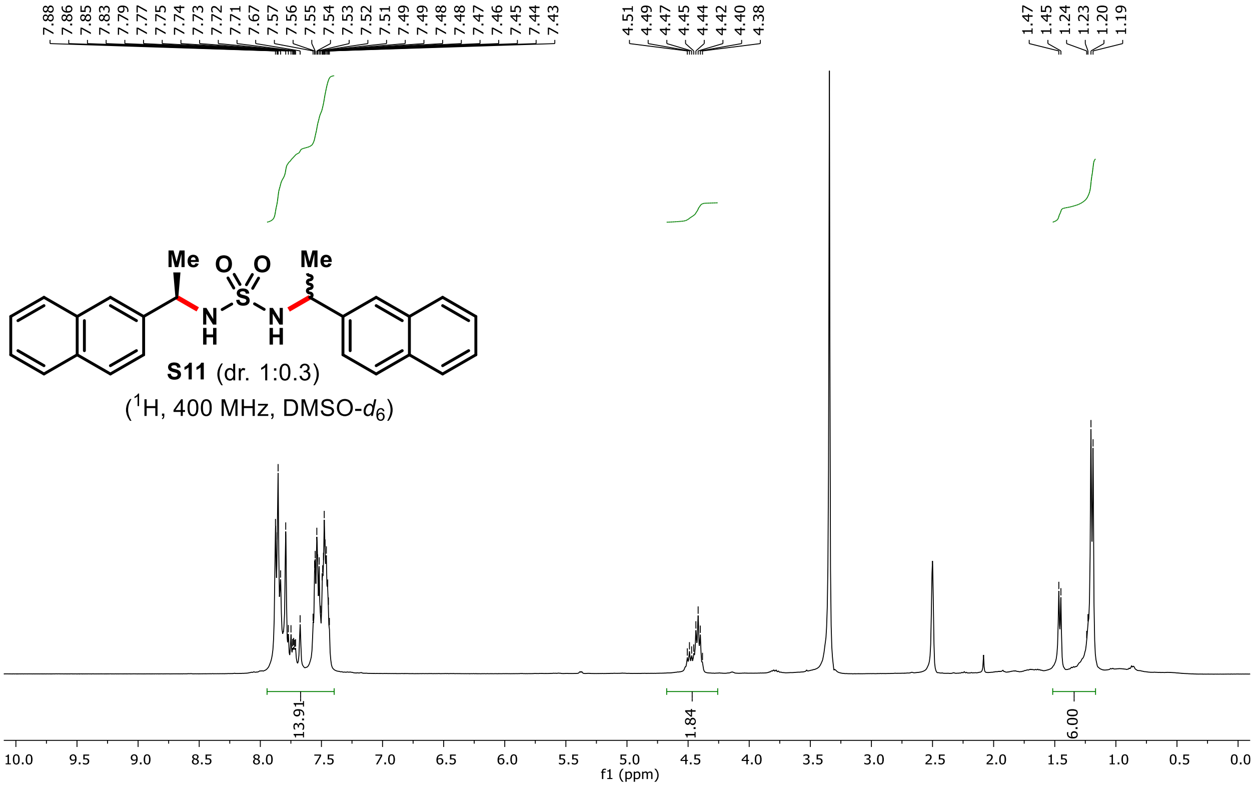

S11 (dr. 1:0.3)
(^{1}H, 400 MHz, DMSO-d_6)

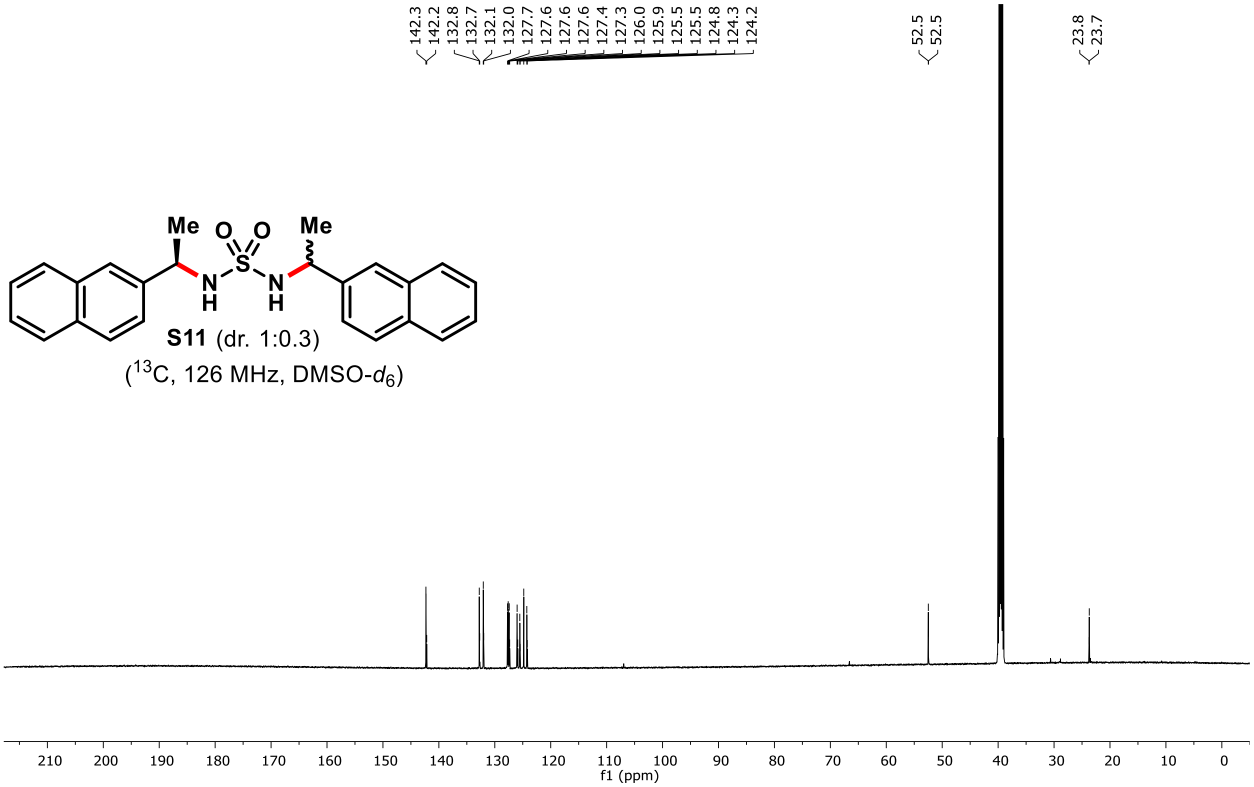
142.3
142.2
132.8
132.7
132.1
132.0
127.7
127.6
127.6
127.4
127.3
126.0
125.9
125.5
125.5
124.8
124.3
124.2
52.5
52.5
23.8
23.7
Me
O
O
S
N
H
Me
N
H
S11 (dr. 1:0.3)
(13C, 126 MHz, DMSO-d6)
f1 (ppm)

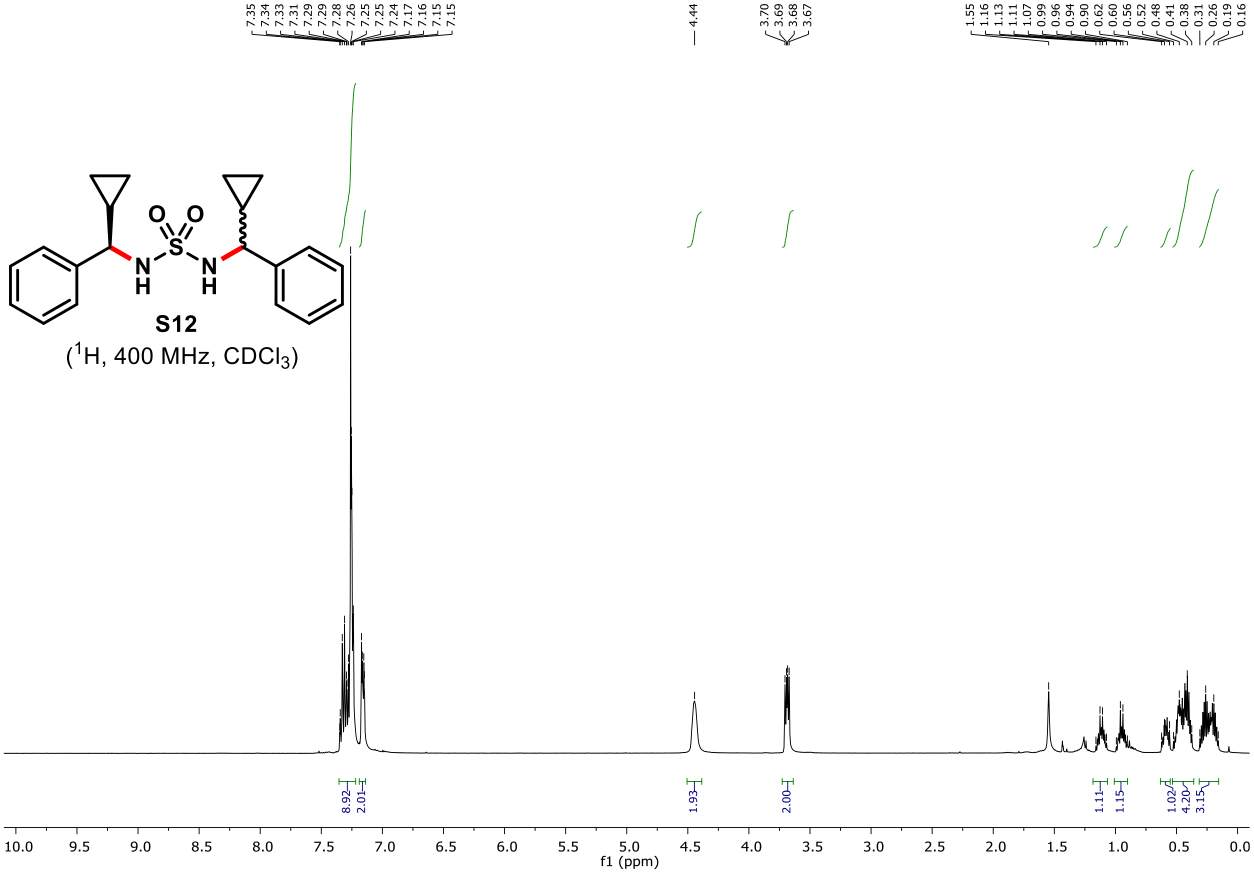
7.35
7.34
7.33
7.31
7.29
7.29
7.28
7.26
7.25
7.25
7.24
7.17
7.16
7.15
7.15
4.44
3.70
3.69
3.68
3.67
1.55
1.16
1.13
1.11
1.07
0.99
0.96
0.94
0.90
0.62
0.60
0.56
0.52
0.48
0.41
0.38
0.31
0.26
0.19
0.16
O
O
S
N
H
N
H
S12
(1H, 400 MHz, CDCl3)
8.92
2.01
1.93
2.00
1.11
1.15
1.02
4.20
3.15
f1 (ppm)

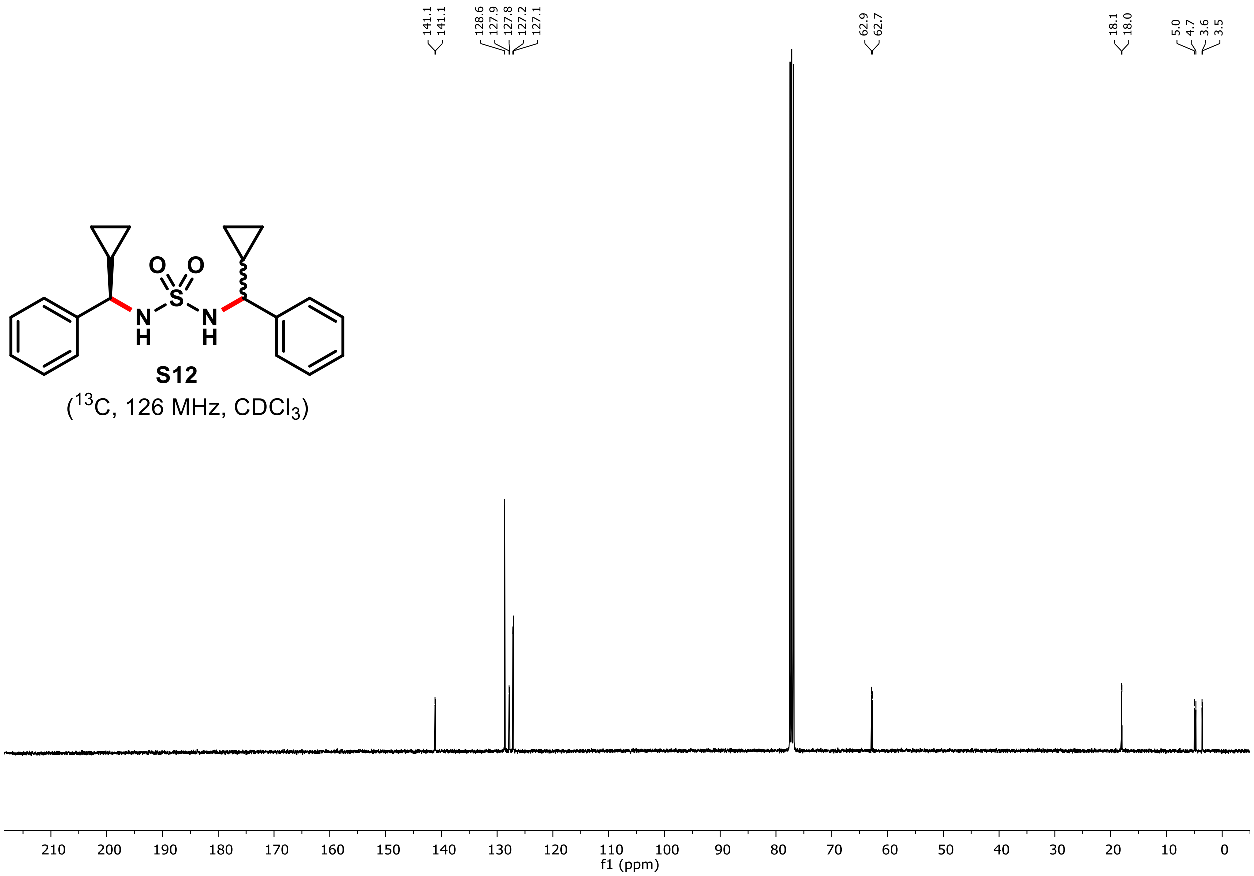
141.1
141.1
128.6
127.9
127.8
127.2
127.1
62.9
62.7
18.1
18.0
5.0
4.7
3.6
3.5
O
O
S
N
H
N
H
S12
(^{13}C, 126 MHz, $CDCl_3$)
210 200 190 180 170 160 150 140 130 120 110 100 90 80 70 60 50 40 30 20 10 0
f1 (ppm)

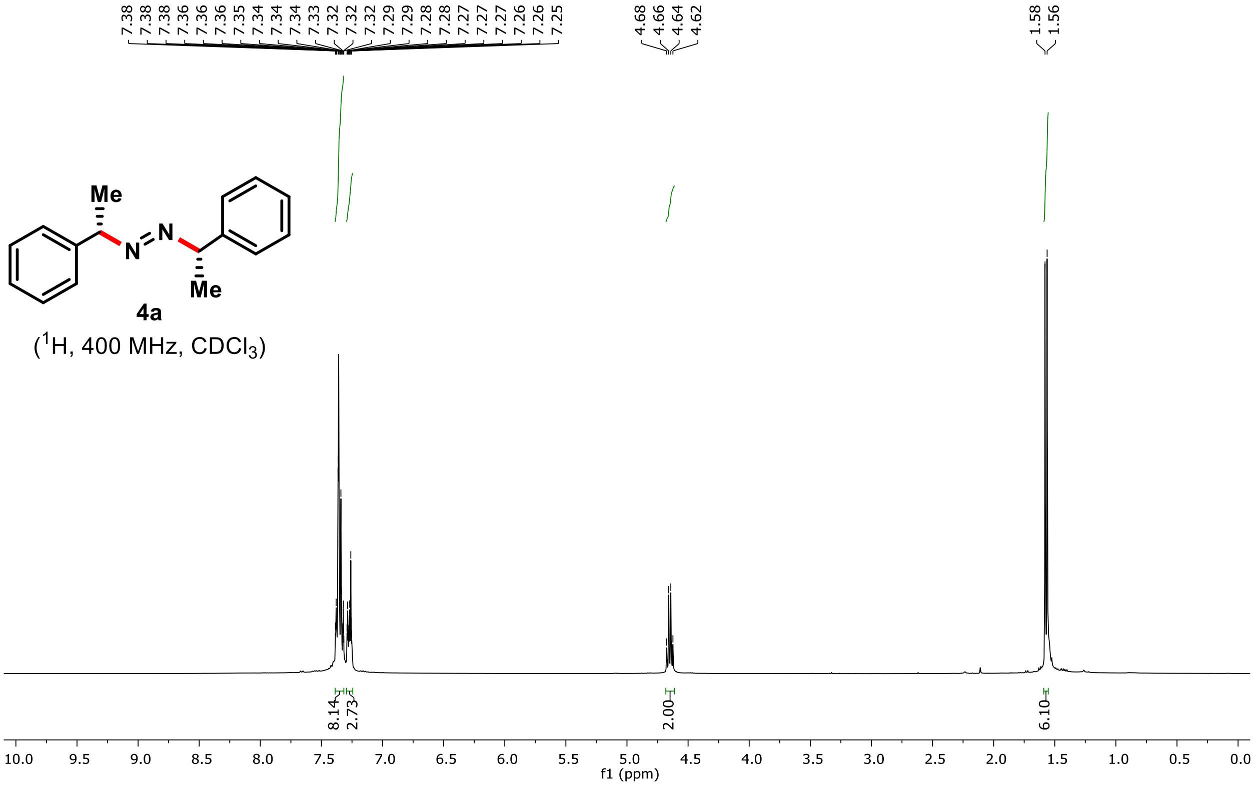
7.38
7.38
7.38
7.36
7.36
7.36
7.35
7.34
7.34
7.34
7.33
7.32
7.32
7.32
7.29
7.29
7.28
7.28
7.27
7.27
7.27
7.26
7.26
7.25
4.68
4.66
4.64
4.62
1.58
1.56
Me
N
N
Me
4a
(^{1}H, 400 MHz, $CDCl_3$)
8.14
2.73
2.00
6.10
10.0 9.5 9.0 8.5 8.0 7.5 7.0 6.5 6.0 5.5 5.0 4.5 4.0 3.5 3.0 2.5 2.0 1.5 1.0 0.5 0.0
f1 (ppm)

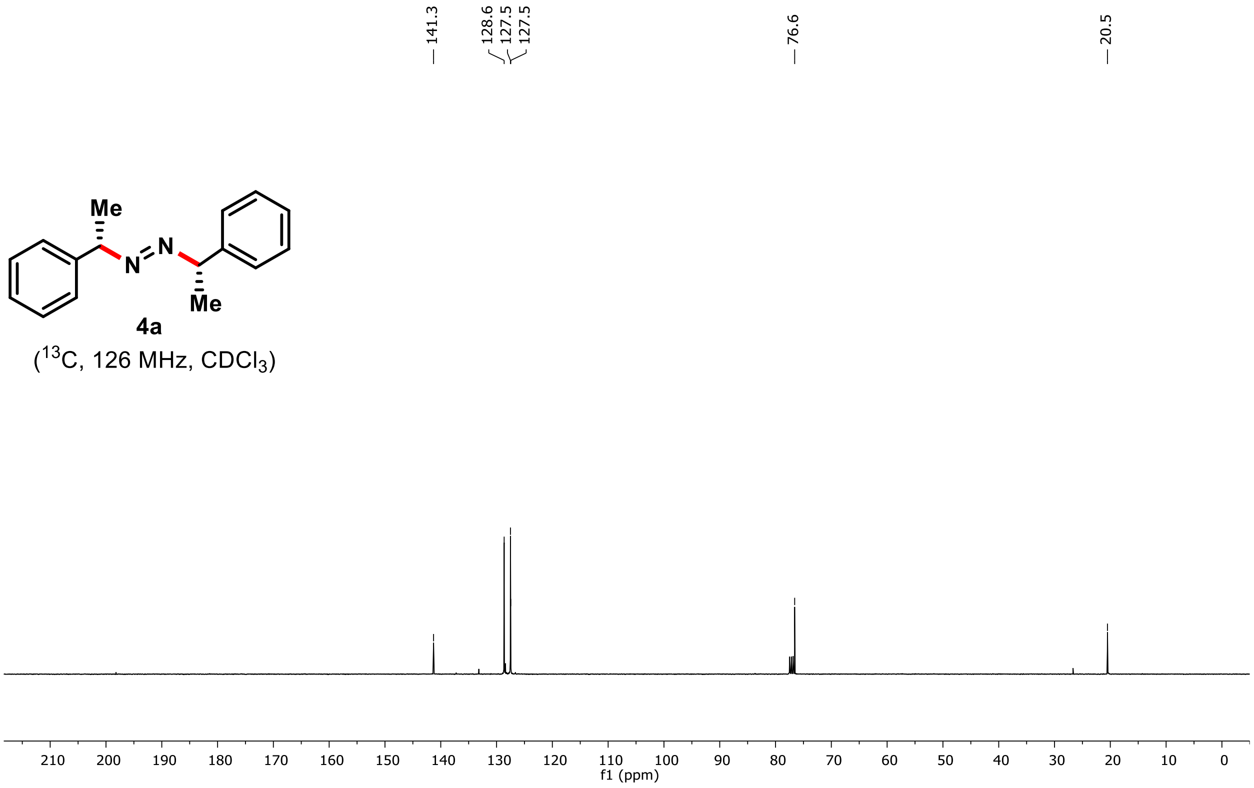
141.3
128.6
127.5
127.5
76.6
20.5
Me
N
N
Me
4a
(13C, 126 MHz, CDCl3)
210 200 190 180 170 160 150 140 130 120 110 100 90 80 70 60 50 40 30 20 10 0
f1 (ppm)

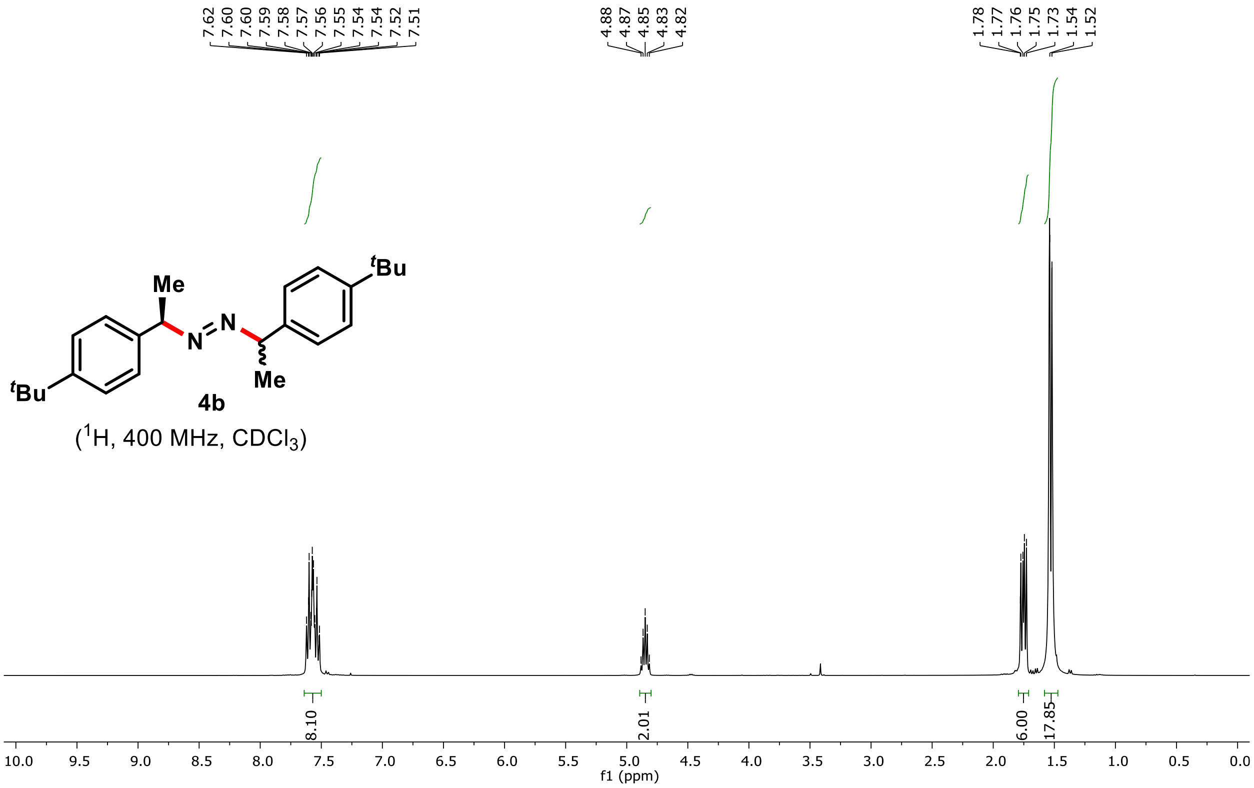
7.62
7.60
7.59
7.58
7.57
7.56
7.55
7.54
7.54
7.52
7.51
4.88
4.87
4.85
4.83
4.82
1.78
1.77
1.76
1.75
1.73
1.54
1.52
tBu
Me
N
N
tBu
Me
4b
(1H, 400 MHz, CDCl3)
8.10
2.01
6.00
17.85
10.0 9.5 9.0 8.5 8.0 7.5 7.0 6.5 6.0 5.5 5.0 4.5 4.0 3.5 3.0 2.5 2.0 1.5 1.0 0.5 0.0
f1 (ppm)

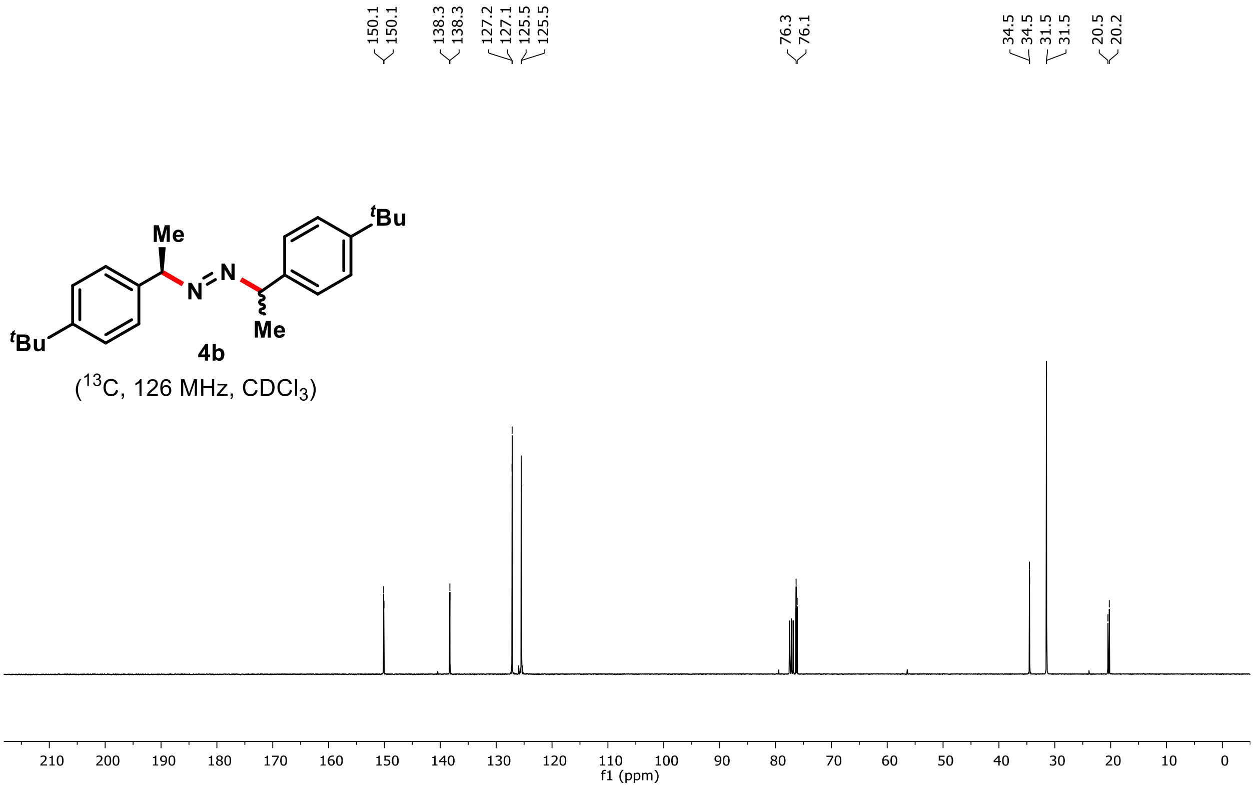
150.1
150.1
138.3
138.3
127.2
127.1
125.5
125.5
76.3
76.1
34.5
34.5
31.5
31.5
20.5
20.2
Me
tBu
N
N
Me
tBu
4b
(13C, 126 MHz, CDCl3)
210 200 190 180 170 160 150 140 130 120 110 100 90 80 70 60 50 40 30 20 10 0
f1 (ppm)

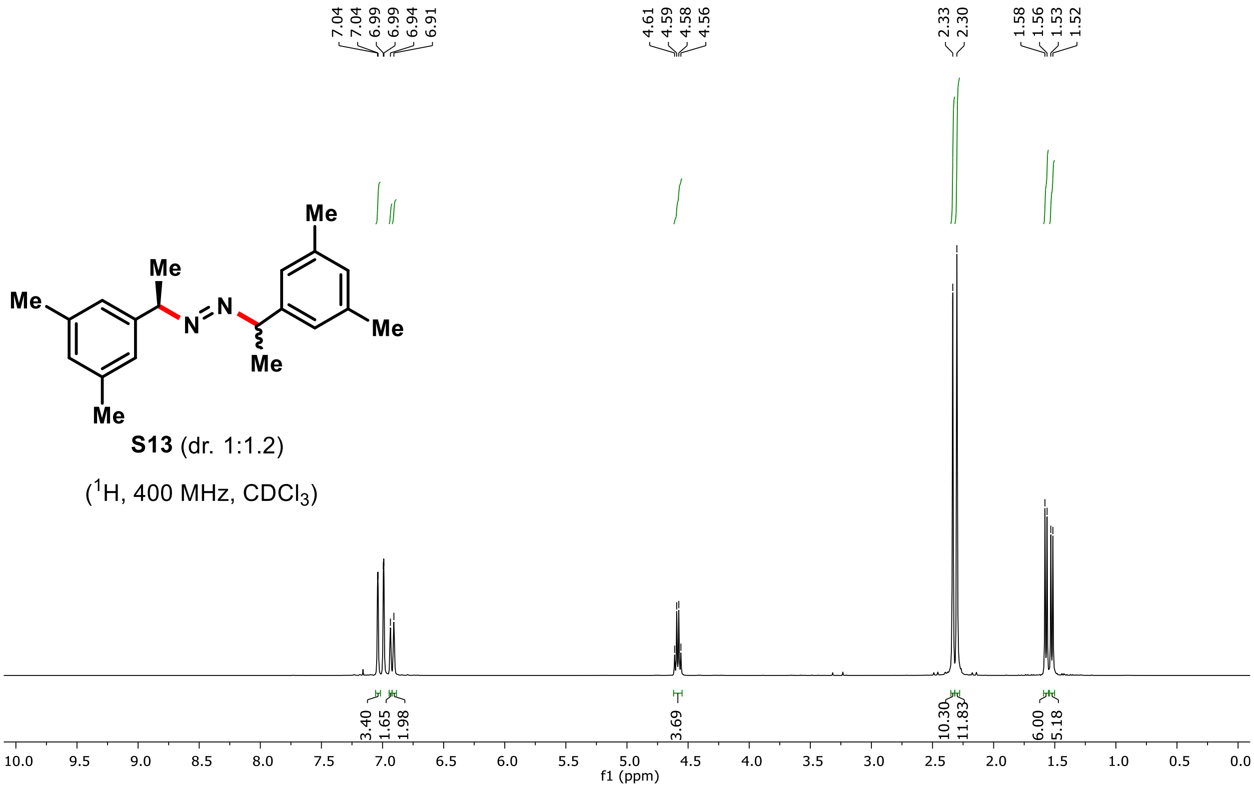
7.04
7.04
6.99
6.99
6.94
6.91
4.61
4.59
4.58
4.56
2.33
2.30
1.58
1.56
1.53
1.52
Me
Me
Me
N
N
Me
Me
Me
S13 (dr. 1:1.2)
(1H, 400 MHz, CDCl3)
3.40
1.65
1.98
3.69
10.30
11.83
6.00
5.18
10.0 9.5 9.0 8.5 8.0 7.5 7.0 6.5 6.0 5.5 5.0 4.5 4.0 3.5 3.0 2.5 2.0 1.5 1.0 0.5 0.0
f1 (ppm)

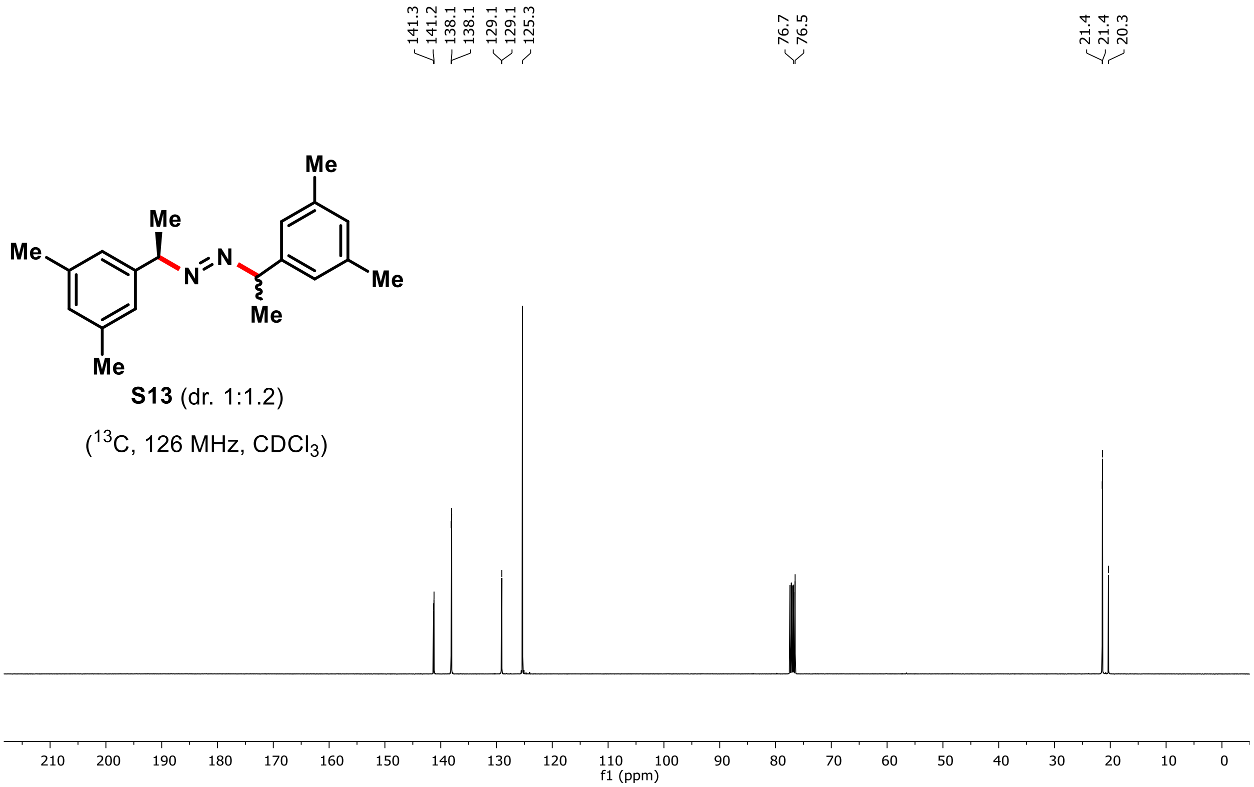
141.3
141.2
138.1
138.1
129.1
129.1
125.3
76.7
76.5
21.4
21.4
20.3
Me
Me
N
N
Me
Me
Me
Me
S13 (dr. 1:1.2)
(^{13}C, 126 MHz, $CDCl_3$)
210 200 190 180 170 160 150 140 130 120 110 100 90 80 70 60 50 40 30 20 10 0
f1 (ppm)

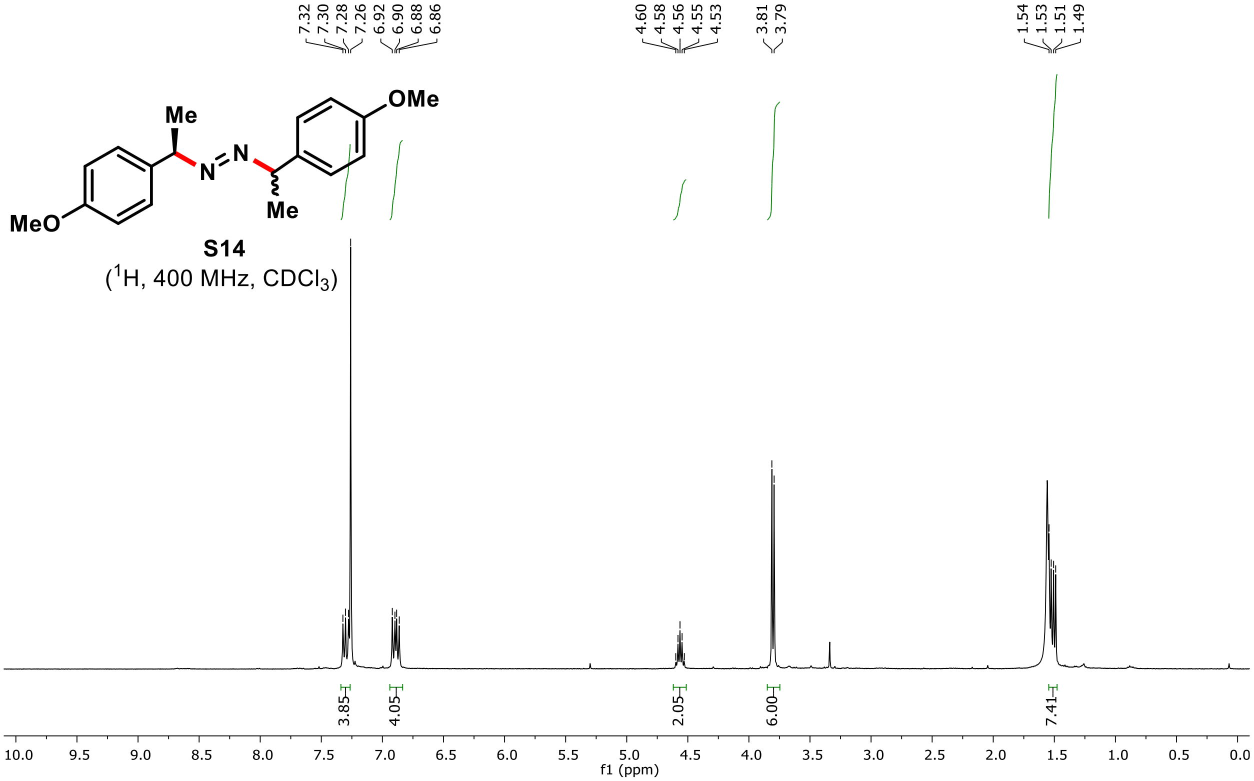
7.32
7.30
7.28
7.26
6.92
6.90
6.88
6.86
4.60
4.58
4.56
4.55
4.53
3.81
3.79
1.54
1.53
1.51
1.49
OMe
Me
N
N
MeO
Me
S14
(^{1}H, 400 MHz, $CDCl_3$)
3.85
4.05
2.05
6.00
7.41
10.0 9.5 9.0 8.5 8.0 7.5 7.0 6.5 6.0 5.5 5.0 4.5 4.0 3.5 3.0 2.5 2.0 1.5 1.0 0.5 0.0
f1 (ppm)

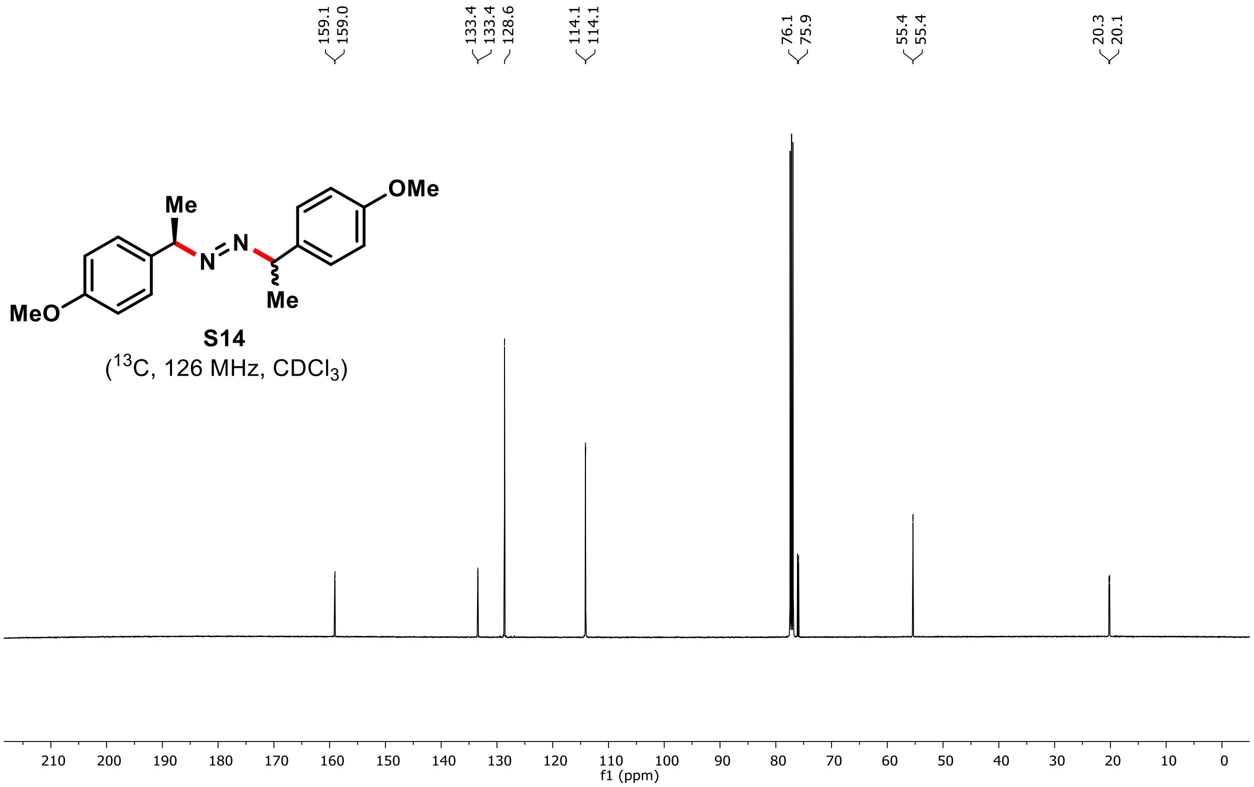
159.1
159.0
133.4
133.4
128.6
114.1
114.1
76.1
75.9
55.4
55.4
20.3
20.1
OMe
Me
N
N
MeO
Me
S14
(^{13}C, 126 MHz, $CDCl_3$)
f1 (ppm)

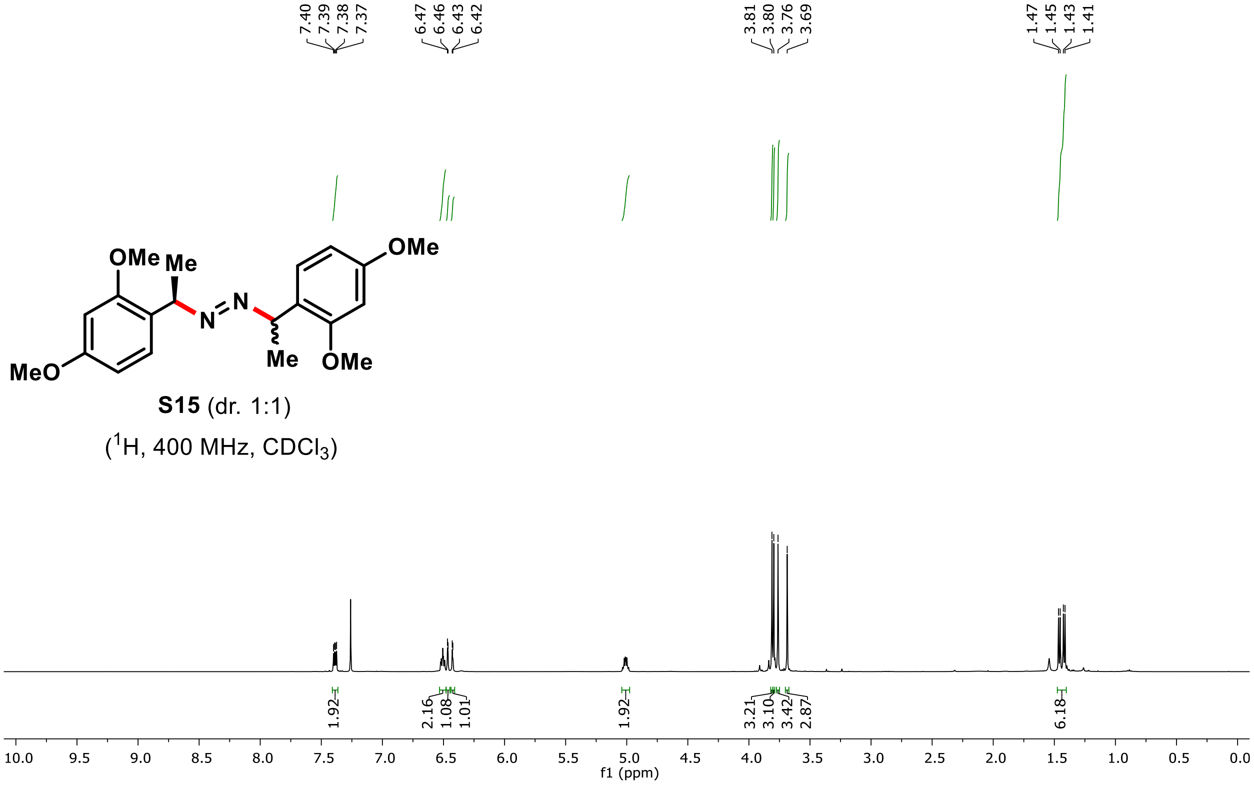
7.40
7.39
7.38
7.37
6.47
6.46
6.43
6.42
3.81
3.80
3.76
3.69
1.47
1.45
1.43
1.41
OMe
Me
N
N
OMe
MeO
Me
OMe
S15 (dr. 1:1)
(^{1}H, 400 MHz, $CDCl_3$)
1.92
2.16
1.08
1.01
1.92
3.21
3.10
3.42
2.87
6.18
f1 (ppm)

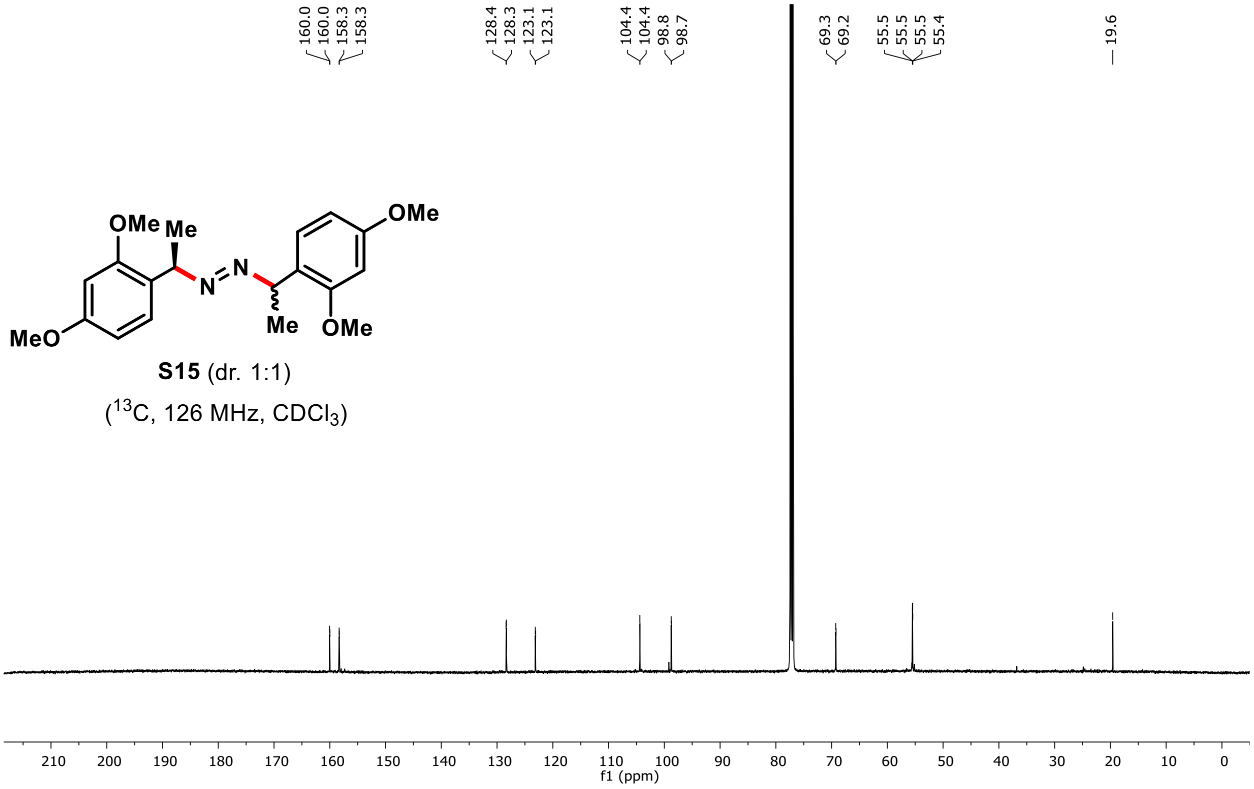
160.0
160.0
158.3
158.3
128.4
128.3
123.1
123.1
104.4
104.4
98.8
98.7
69.3
69.2
55.5
55.5
55.5
55.4
19.6
OMe
Me
N
N
OMe
MeO
Me
OMe
S15 (dr. 1:1)
(^{13}C, 126 MHz, $CDCl_3$)
210 200 190 180 170 160 150 140 130 120 110 100 90 80 70 60 50 40 30 20 10 0
f1 (ppm)

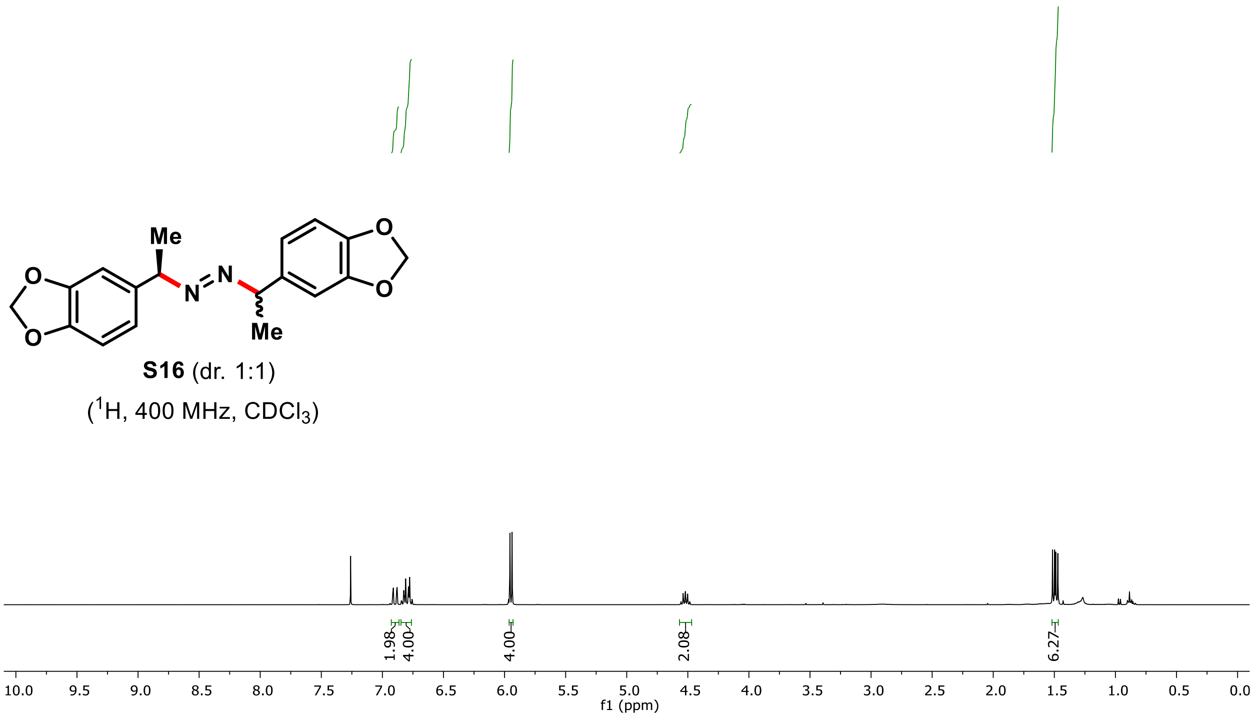
Me
O
O
N
N
O
O
Me
S16 (dr. 1:1)
(^{1}H, 400 MHz, $CDCl_3$)
1.98
4.00
4.00
2.08
6.27
10.0 9.5 9.0 8.5 8.0 7.5 7.0 6.5 6.0 5.5 5.0 4.5 4.0 3.5 3.0 2.5 2.0 1.5 1.0 0.5 0.0
f1 (ppm)

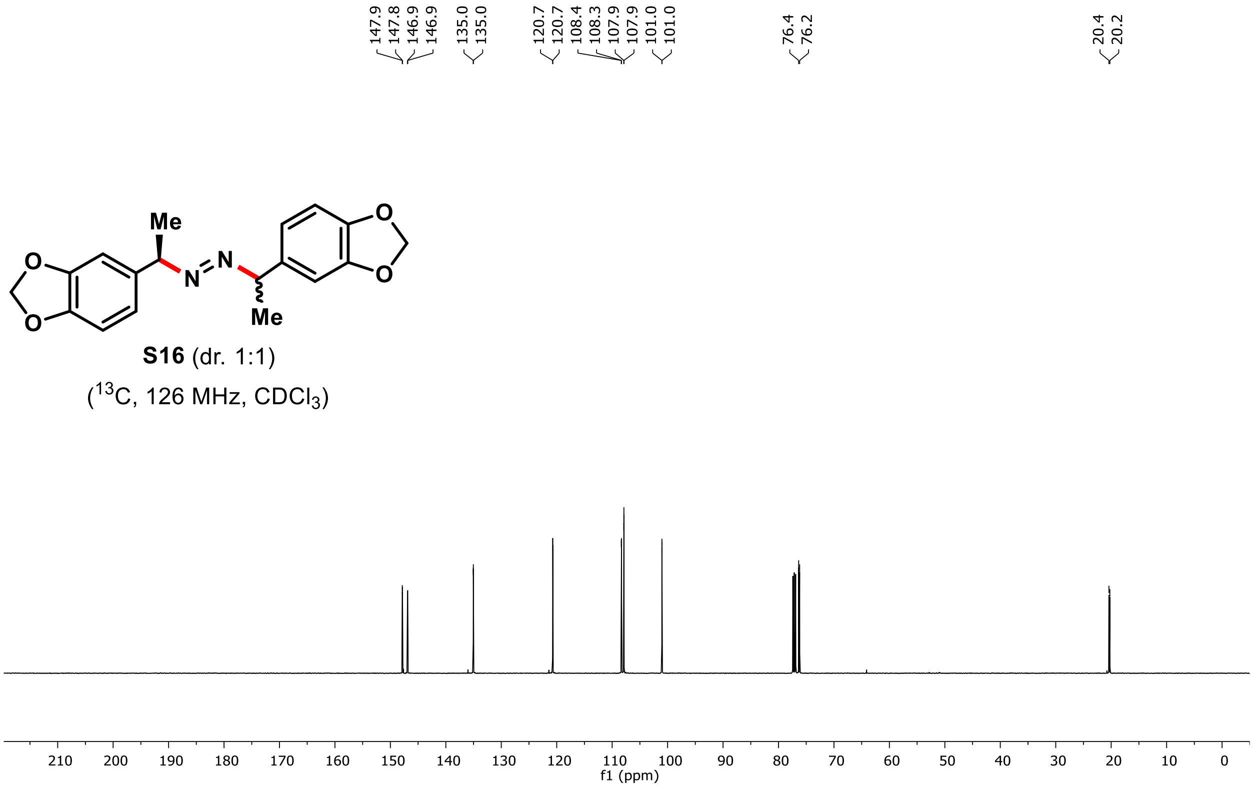

S16 (dr. 1:1)

(^{13}C, 126 MHz, $CDCl_3$)

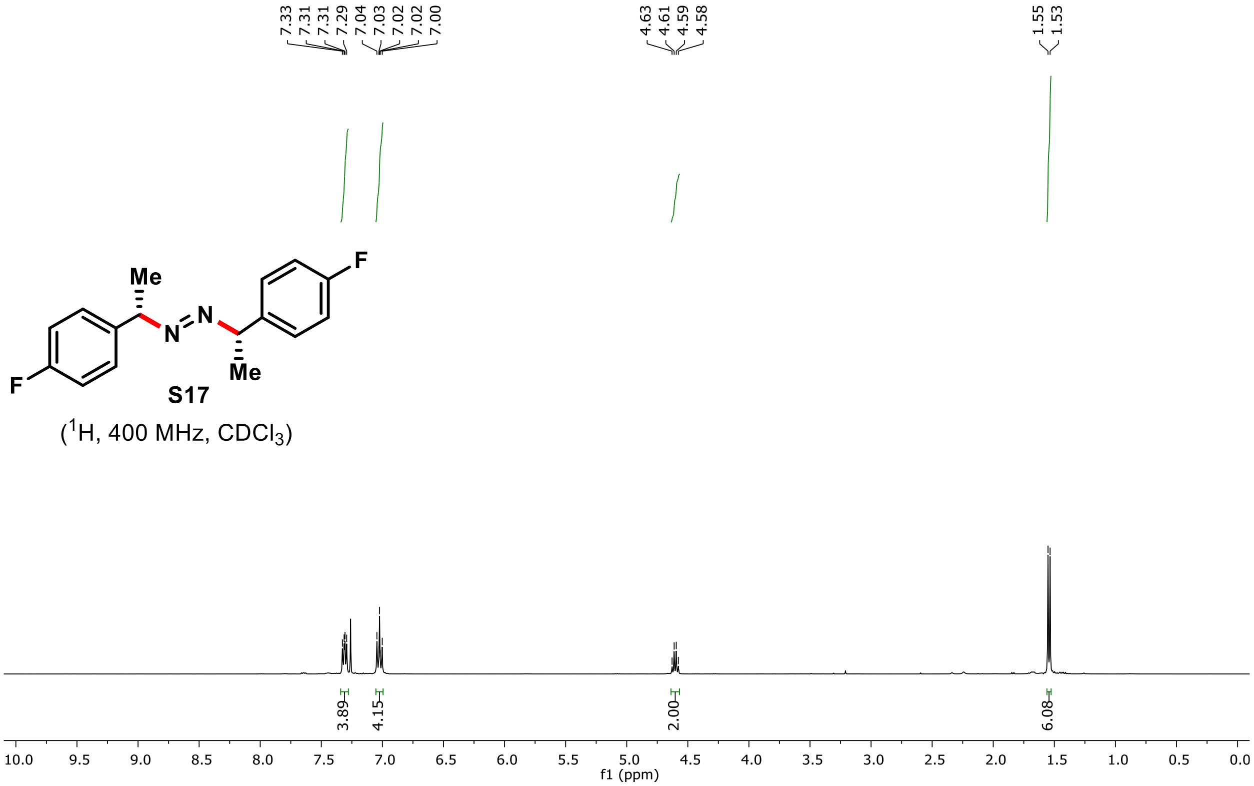

S17

(^{1}H, 400 MHz, $CDCl_3$)

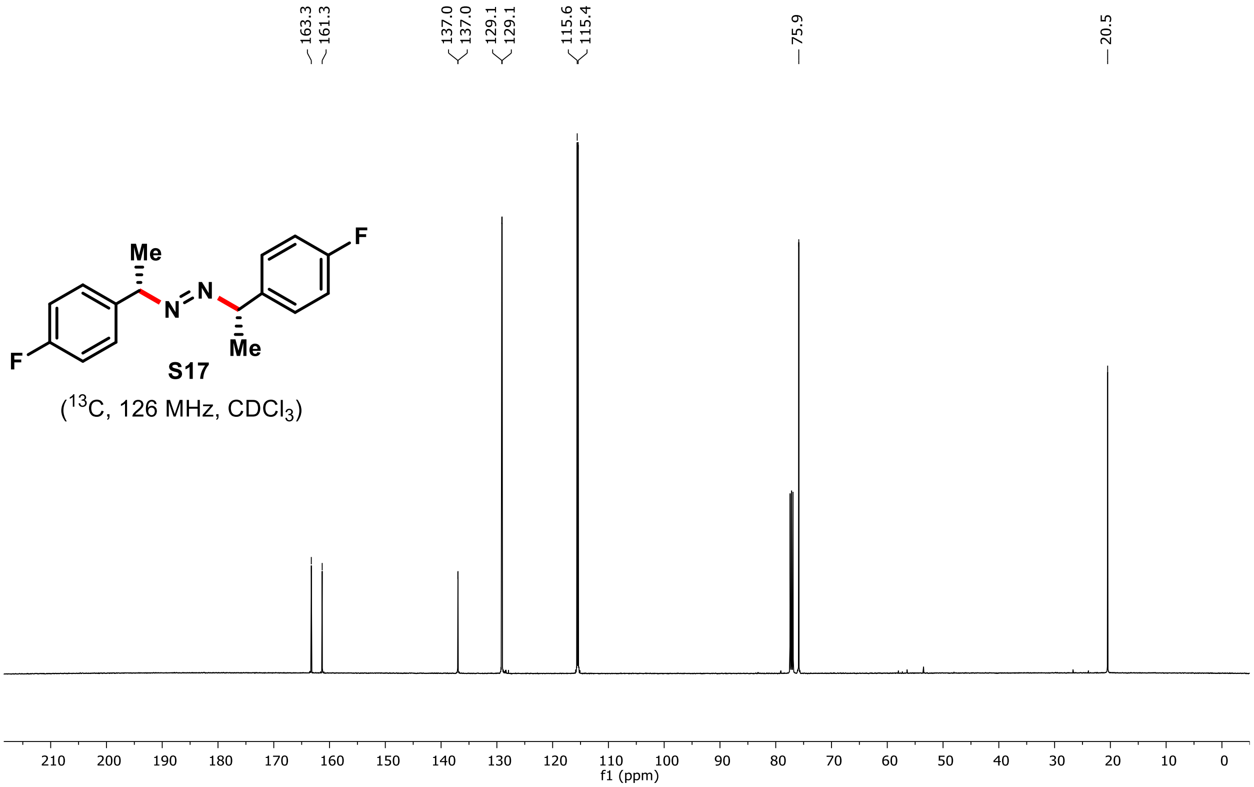
163.3
161.3
137.0
137.0
129.1
129.1
115.6
115.4
75.9
20.5
Me
N
N
F
F
Me
S17
(^{13}C, 126 MHz, $CDCl_3$)
210 200 190 180 170 160 150 140 130 120 110 100 90 80 70 60 50 40 30 20 10 0
f1 (ppm)

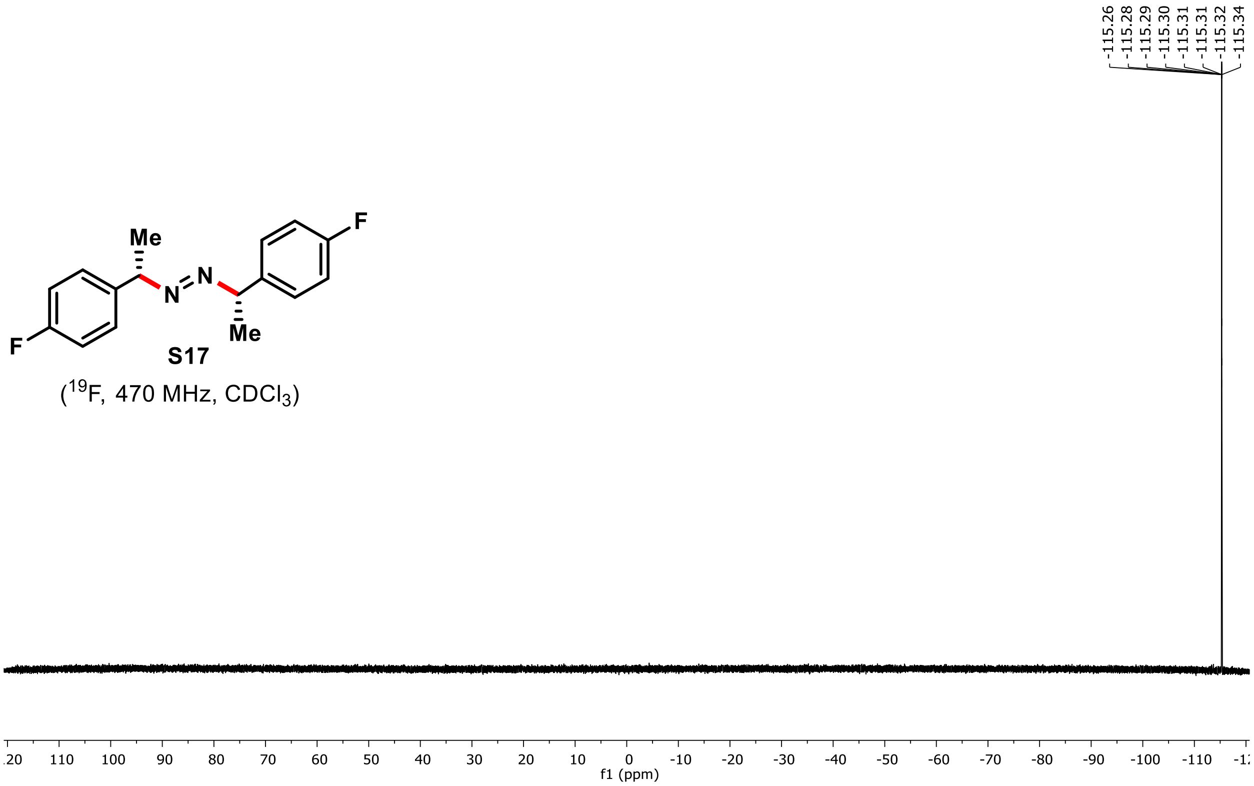
-115.26
-115.28
-115.29
-115.30
-115.31
-115.31
-115.32
-115.34
Me
N
N
F
F
Me
S17
(^{19}F, 470 MHz, $CDCl_3$)
110 100 90 80 70 60 50 40 30 20 10 0 -10 -20 -30 -40 -50 -60 -70 -80 -90 -100 -110
f1 (ppm)

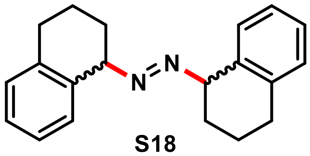

S18

(^{1}H, 400 MHz, $CDCl_3$)

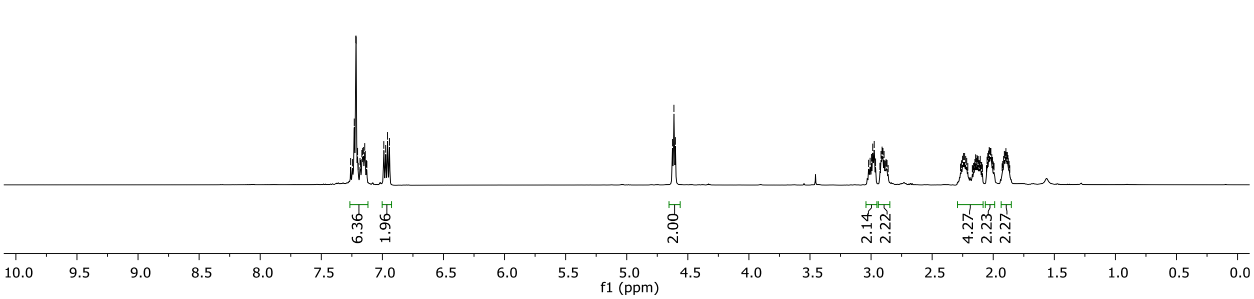

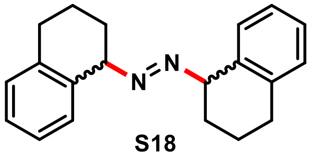

S18

(^{13}C, 126 MHz, $CDCl_3$)

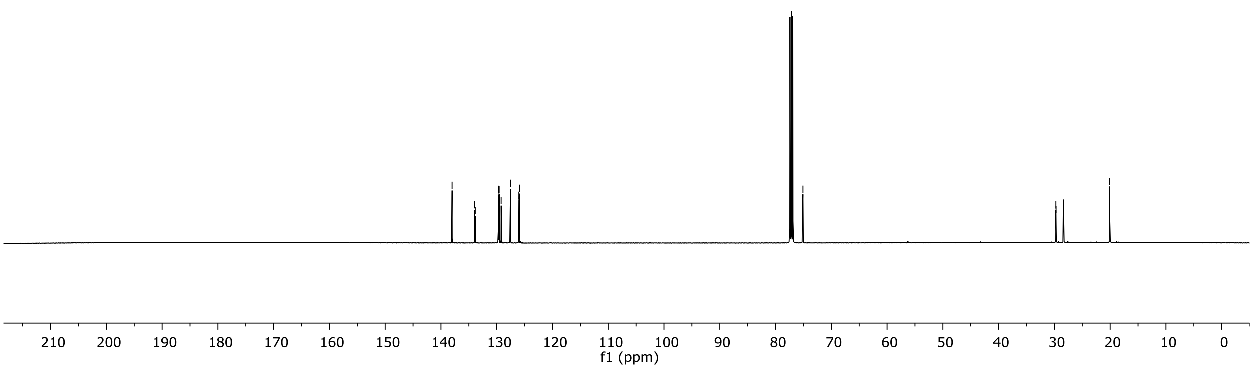

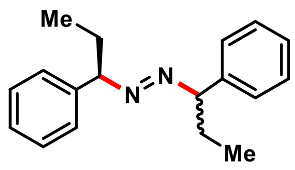

S19 (dr. 1:1)
(^{1}H, 400 MHz, $CDCl_3$)

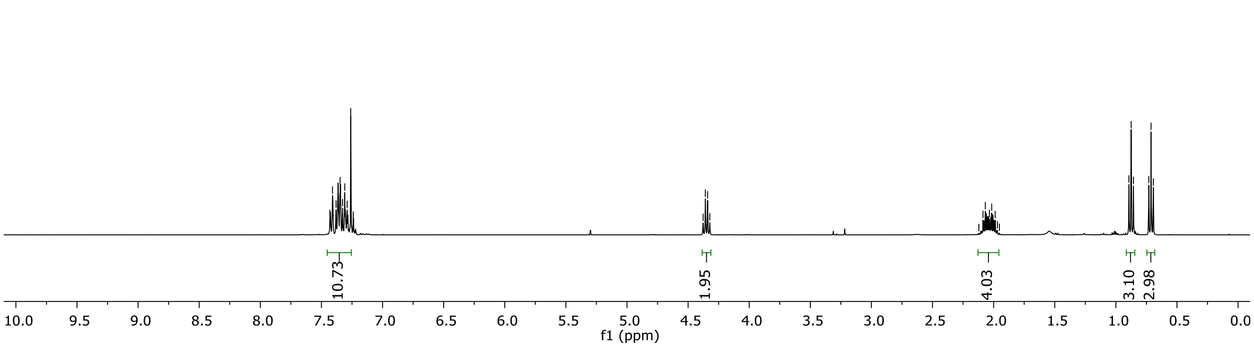

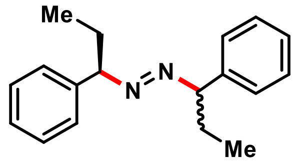

S19 (dr. 1:1)
(^{13}C, 126 MHz, $CDCl_3$)

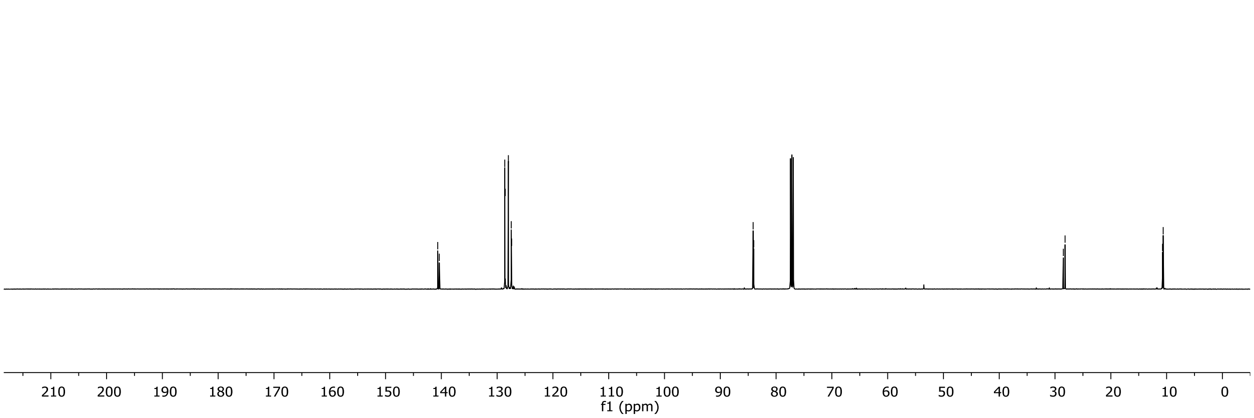

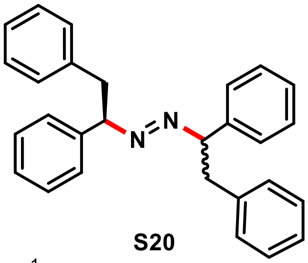

S20

(^{1}H, 400 MHz, $CDCl_3$)

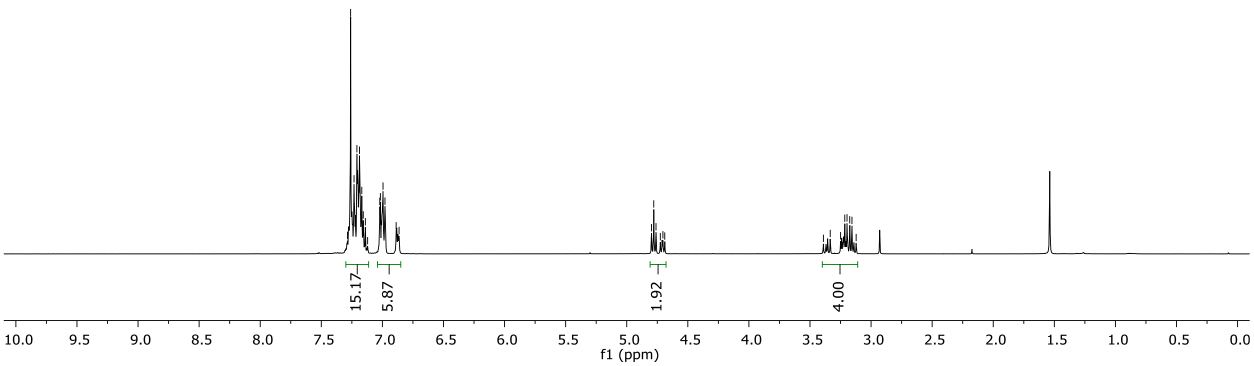

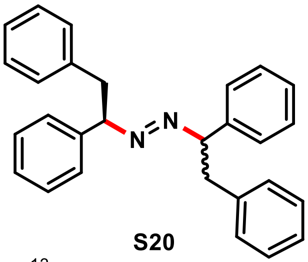

S20

(^{13}C, 126 MHz, $CDCl_3$)

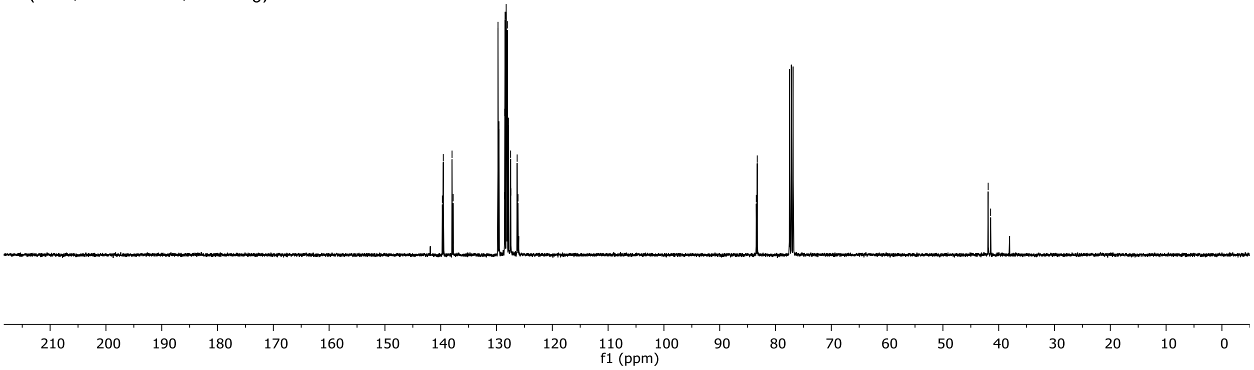

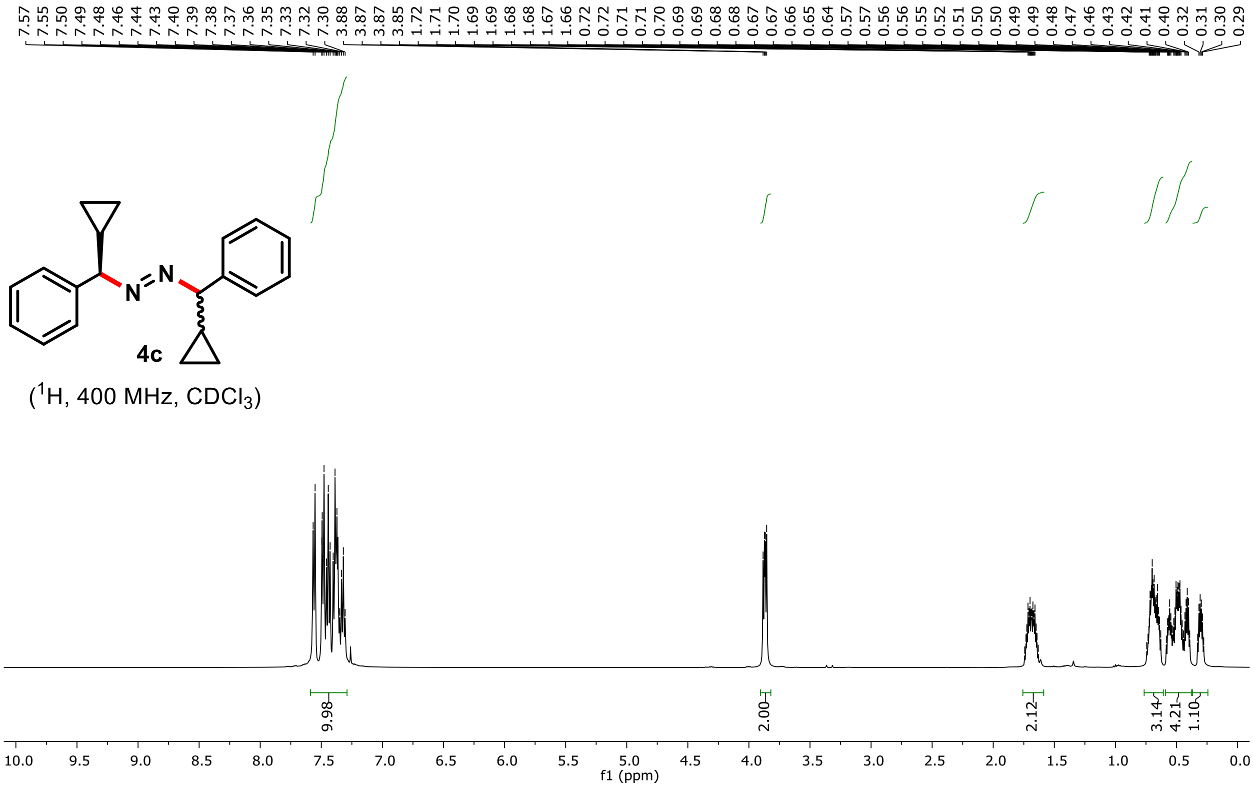
4c
(^{1}H, 400 MHz, CDCl$_3$)
9.98
2.00
2.12
3.14
4.21
1.10
f1 (ppm)

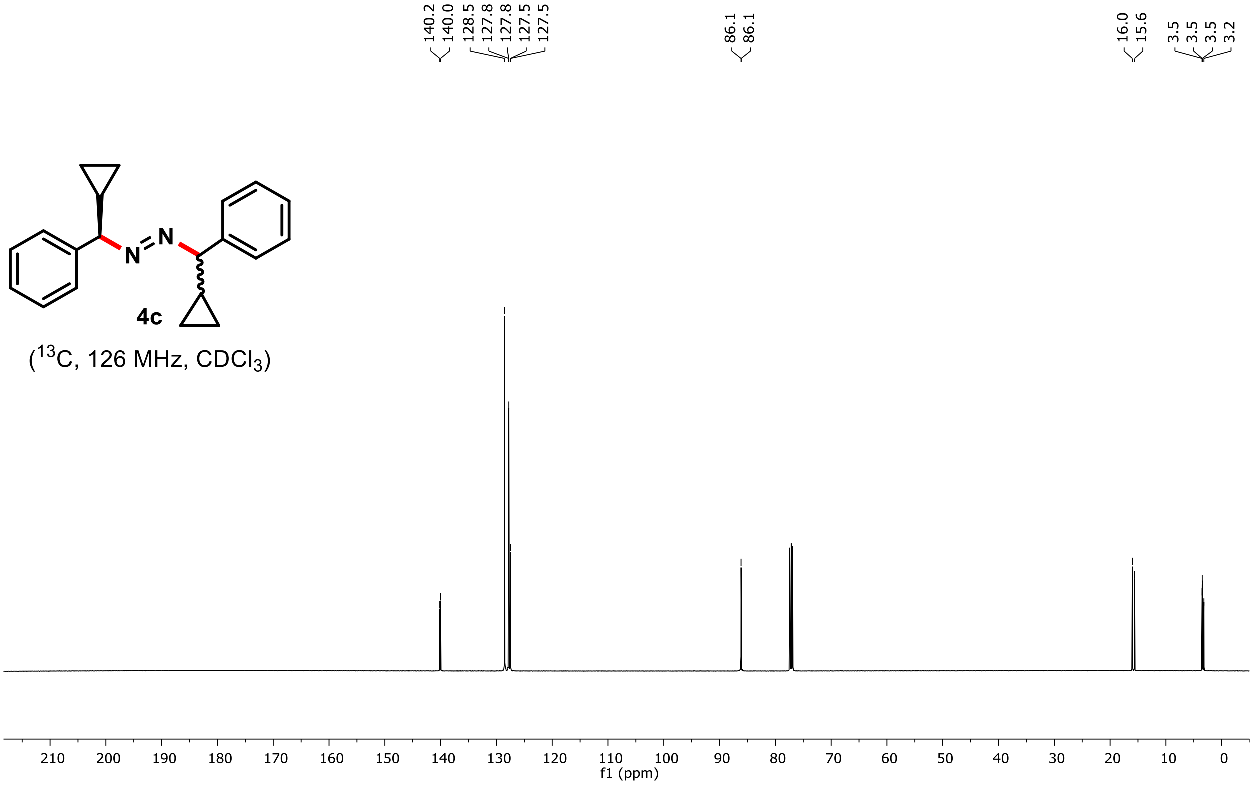
4c
(^{13}C, 126 MHz, CDCl$_3$)
140.2
140.0
128.5
127.8
127.8
127.5
127.5
86.1
86.1
16.0
15.6
3.5
3.5
3.5
3.2
f1 (ppm)

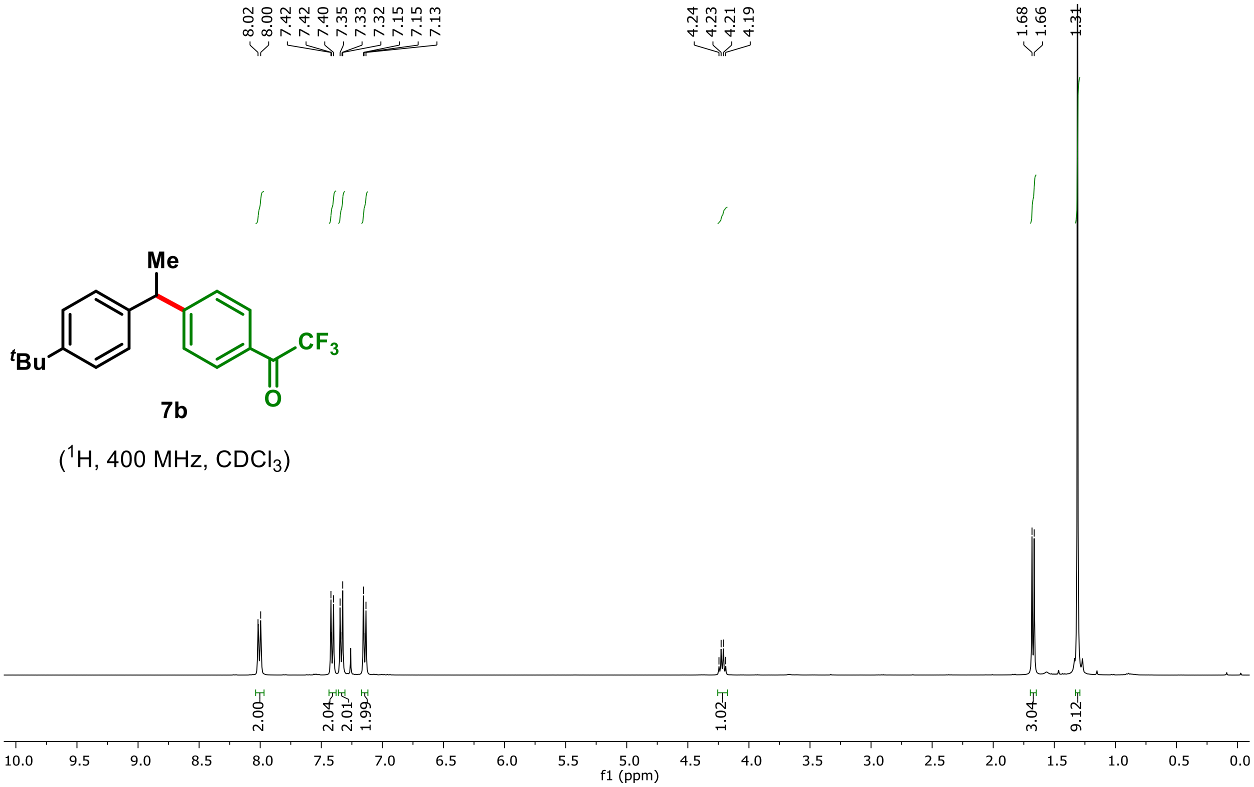
8.02
8.00
7.42
7.42
7.40
7.35
7.33
7.32
7.15
7.15
7.13
4.24
4.23
4.21
4.19
1.68
1.66
1.31
Me
tBu
CF3
O
7b
(1H, 400 MHz, CDCl3)
2.00
2.04
2.01
1.99
1.02
3.04
9.12
10.0 9.5 9.0 8.5 8.0 7.5 7.0 6.5 6.0 5.5 5.0 4.5 4.0 3.5 3.0 2.5 2.0 1.5 1.0 0.5 0.0
f1 (ppm)

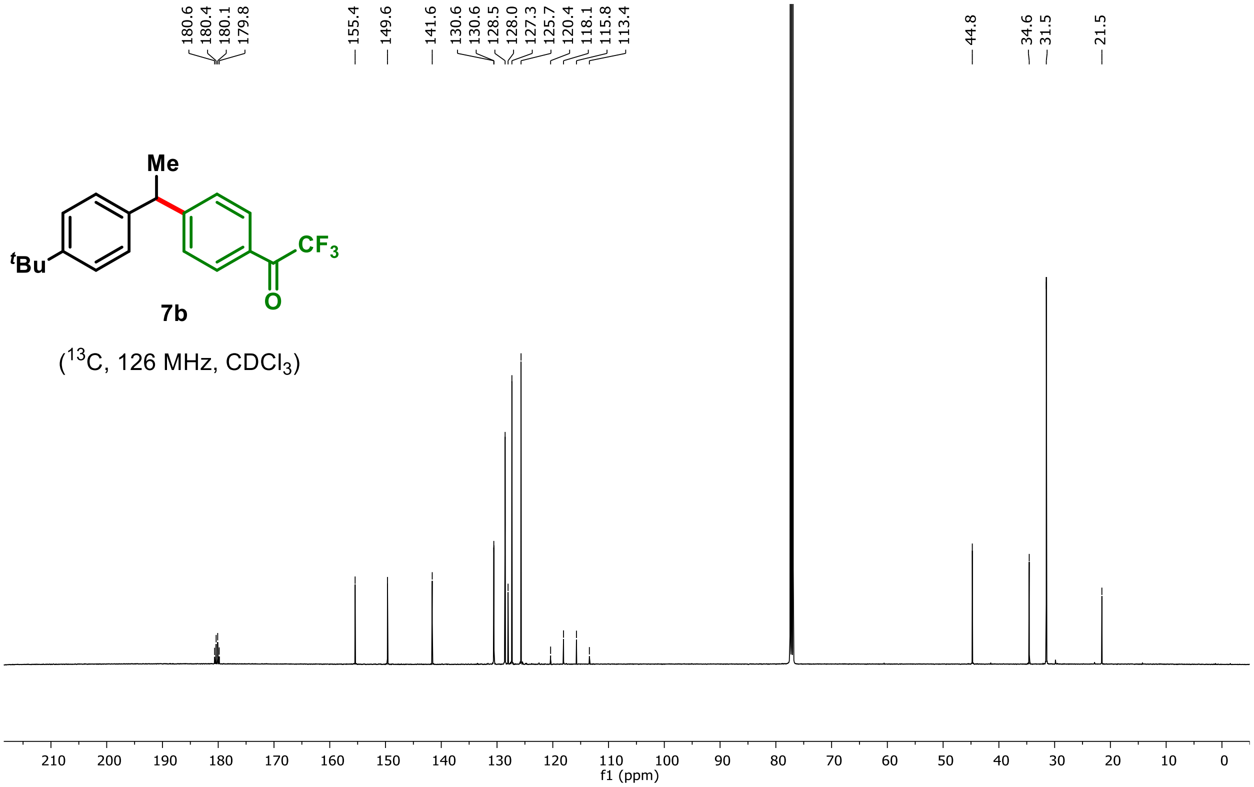
180.6
180.4
180.1
179.8
155.4
149.6
141.6
130.6
130.6
128.5
128.0
127.3
125.7
120.4
118.1
115.8
113.4
44.8
34.6
31.5
21.5
Me
tBu
CF3
O
7b
(13C, 126 MHz, CDCl3)
210 200 190 180 170 160 150 140 130 120 110 100 90 80 70 60 50 40 30 20 10 0
f1 (ppm)

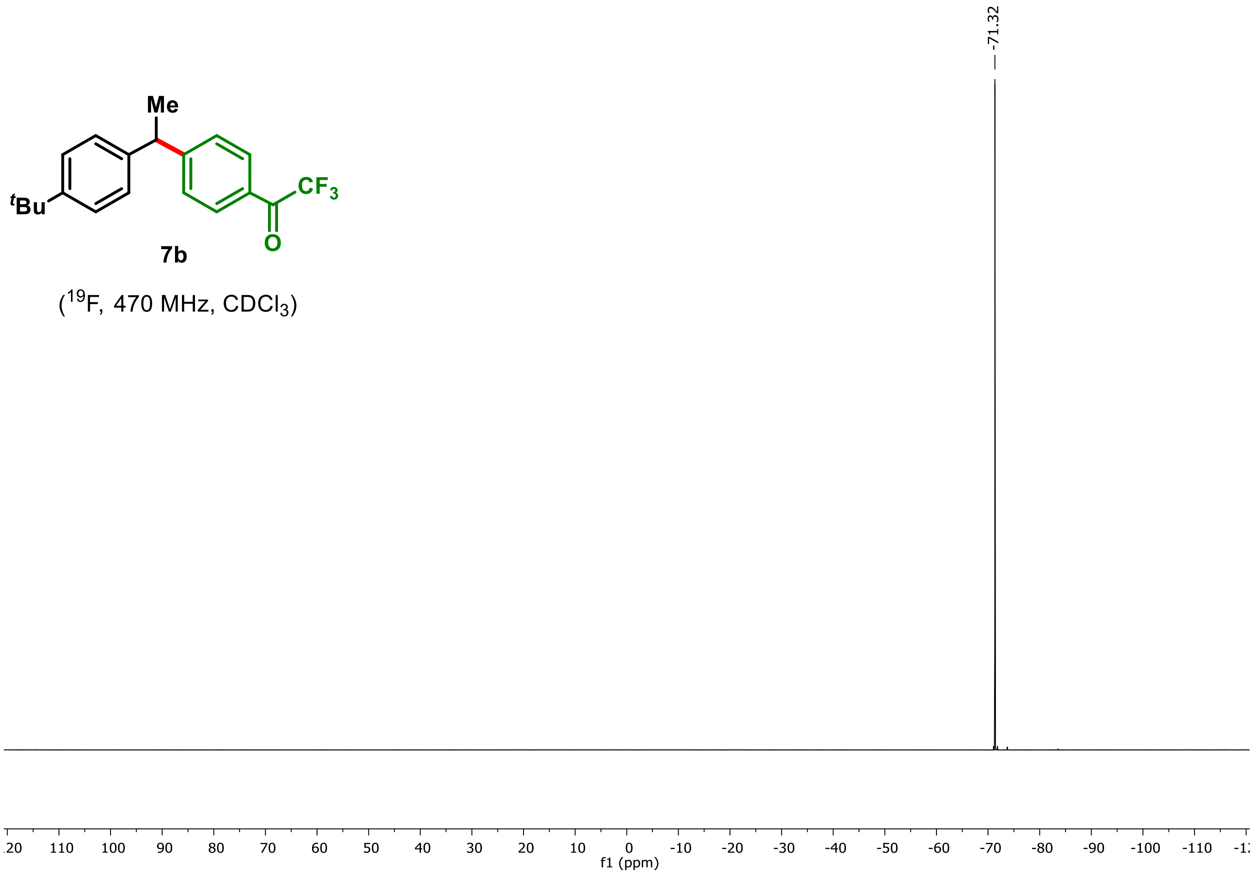

7b

(^{19}F, 470 MHz, $CDCl_3$)

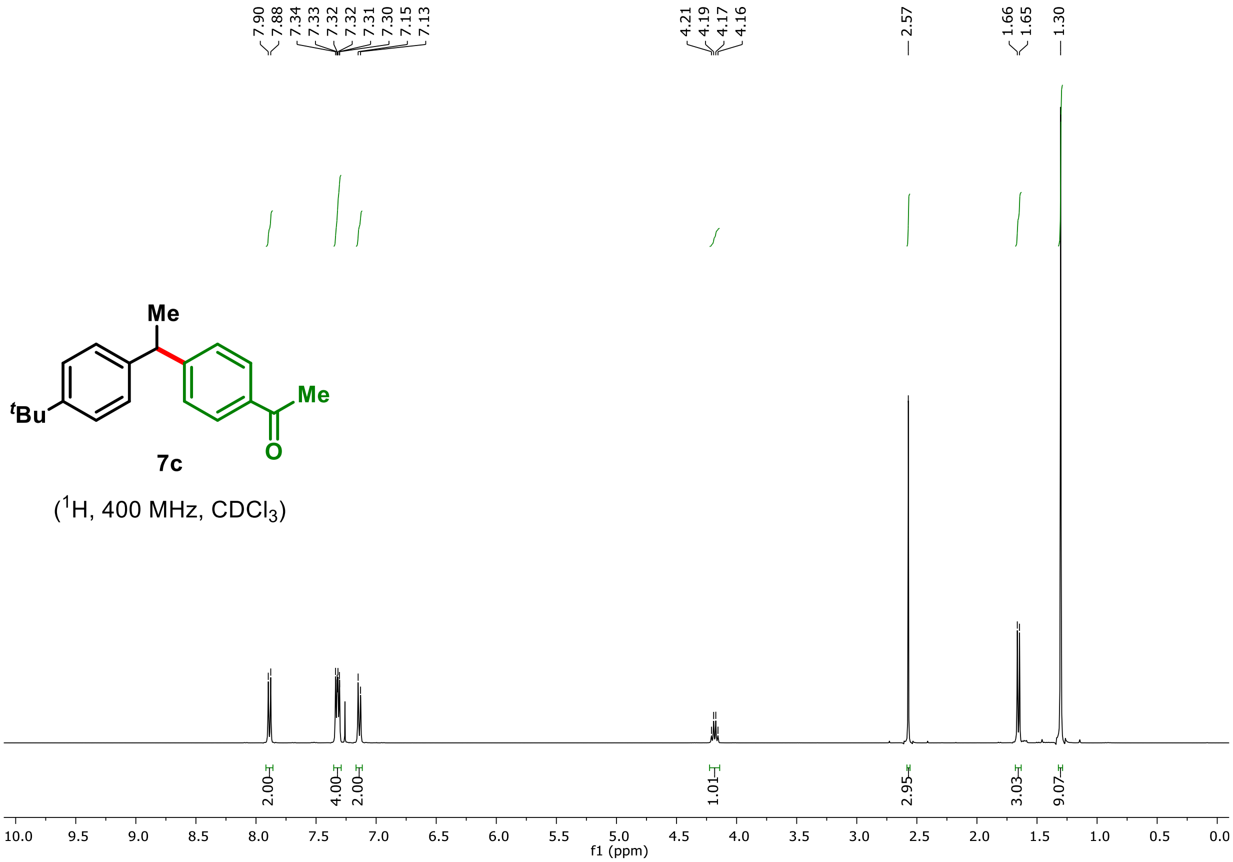

7c

(^{1}H, 400 MHz, $CDCl_3$)

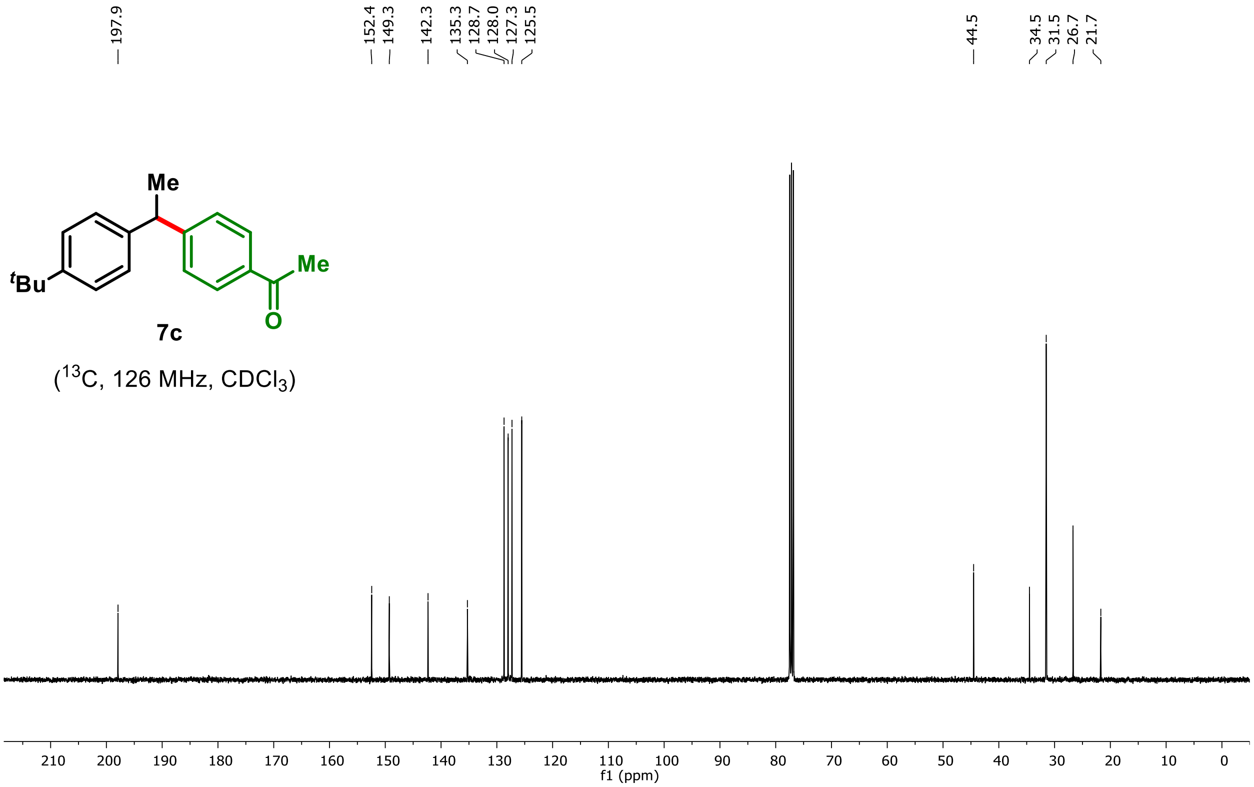
197.9
152.4
149.3
142.3
135.3
128.7
128.0
127.3
125.5
44.5
34.5
31.5
26.7
21.7
Me
tBu
Me
O
7c
(13C, 126 MHz, CDCl3)
f1 (ppm)

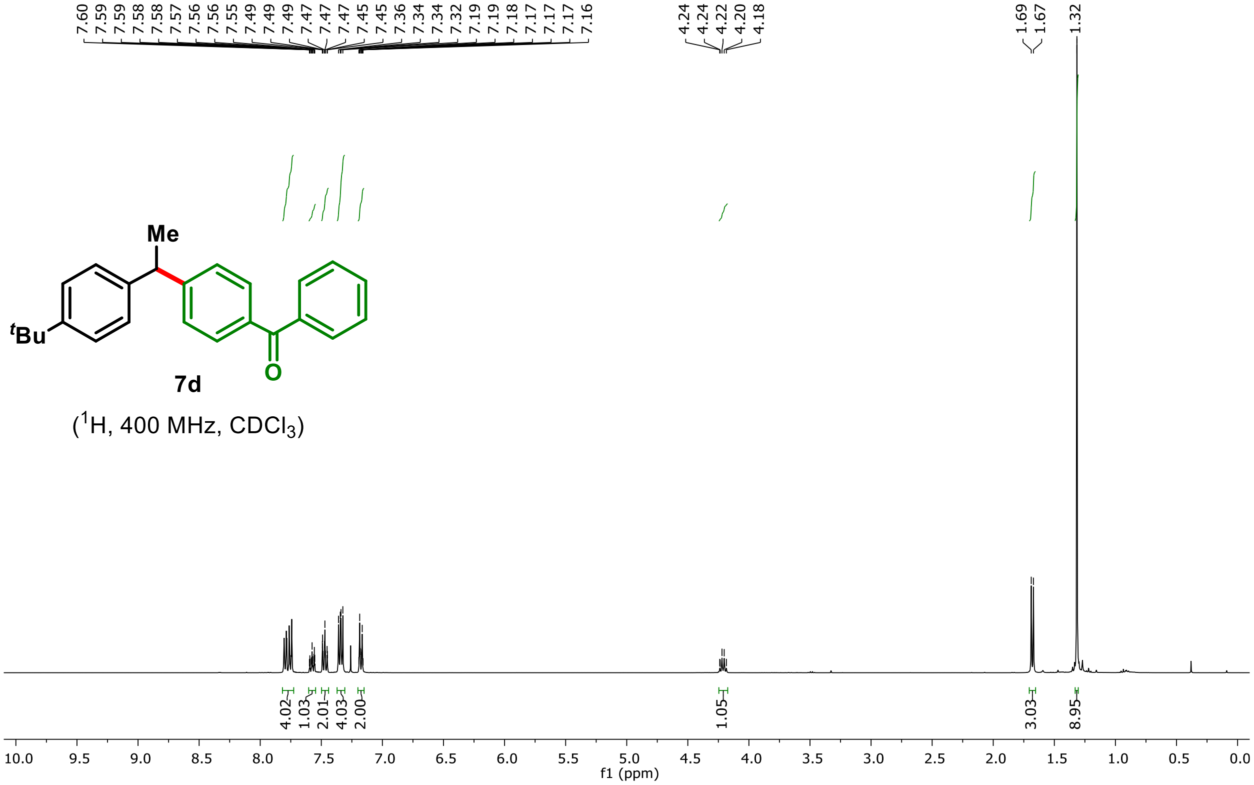
7.60
7.59
7.59
7.58
7.57
7.56
7.56
7.55
7.49
7.49
7.47
7.47
7.47
7.45
7.45
7.36
7.34
7.34
7.32
7.19
7.19
7.18
7.17
7.17
7.17
7.16
4.24
4.24
4.22
4.20
4.18
1.69
1.67
1.32
Me
tBu
O
7d
(1H, 400 MHz, CDCl3)
4.02
1.03
2.01
4.03
2.00
1.05
3.03
8.95
f1 (ppm)

7d

(^{13}C, 126 MHz, $CDCl_3$)

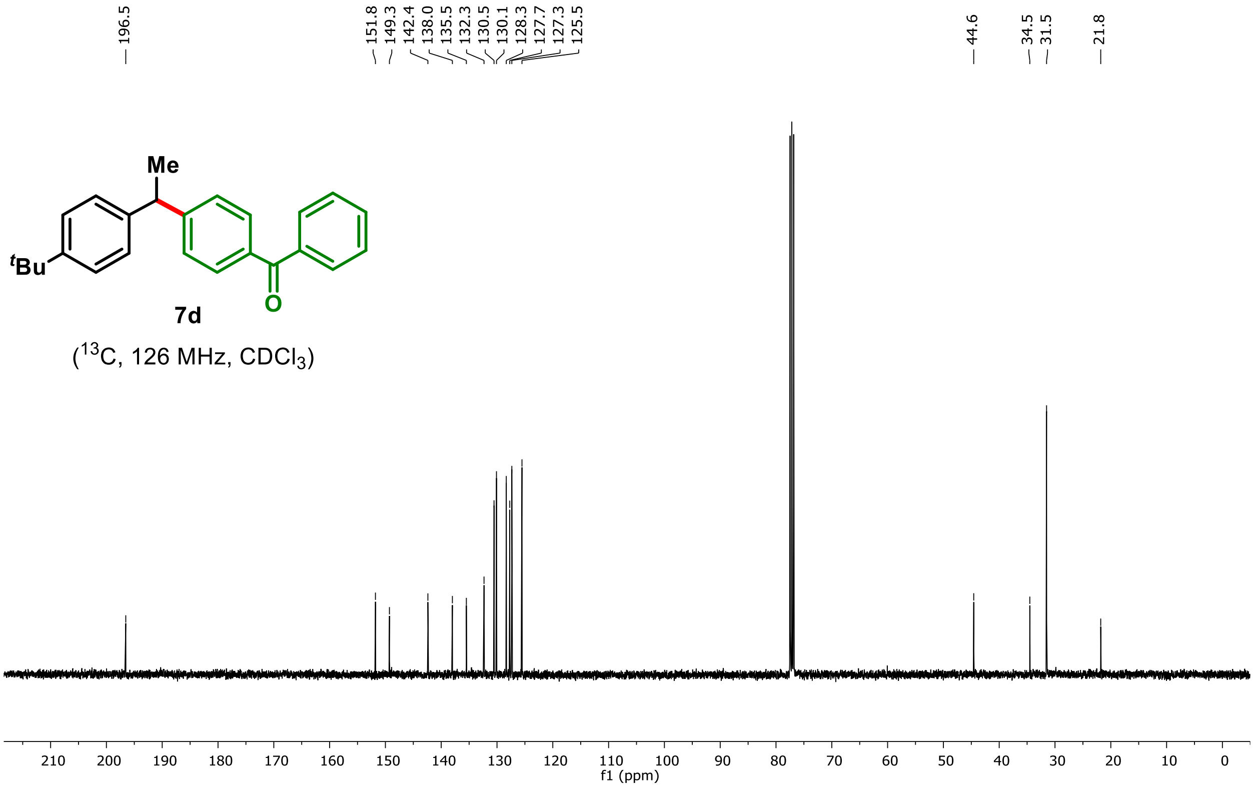

7e

(^{1}H, 400 MHz, $CDCl_3$)

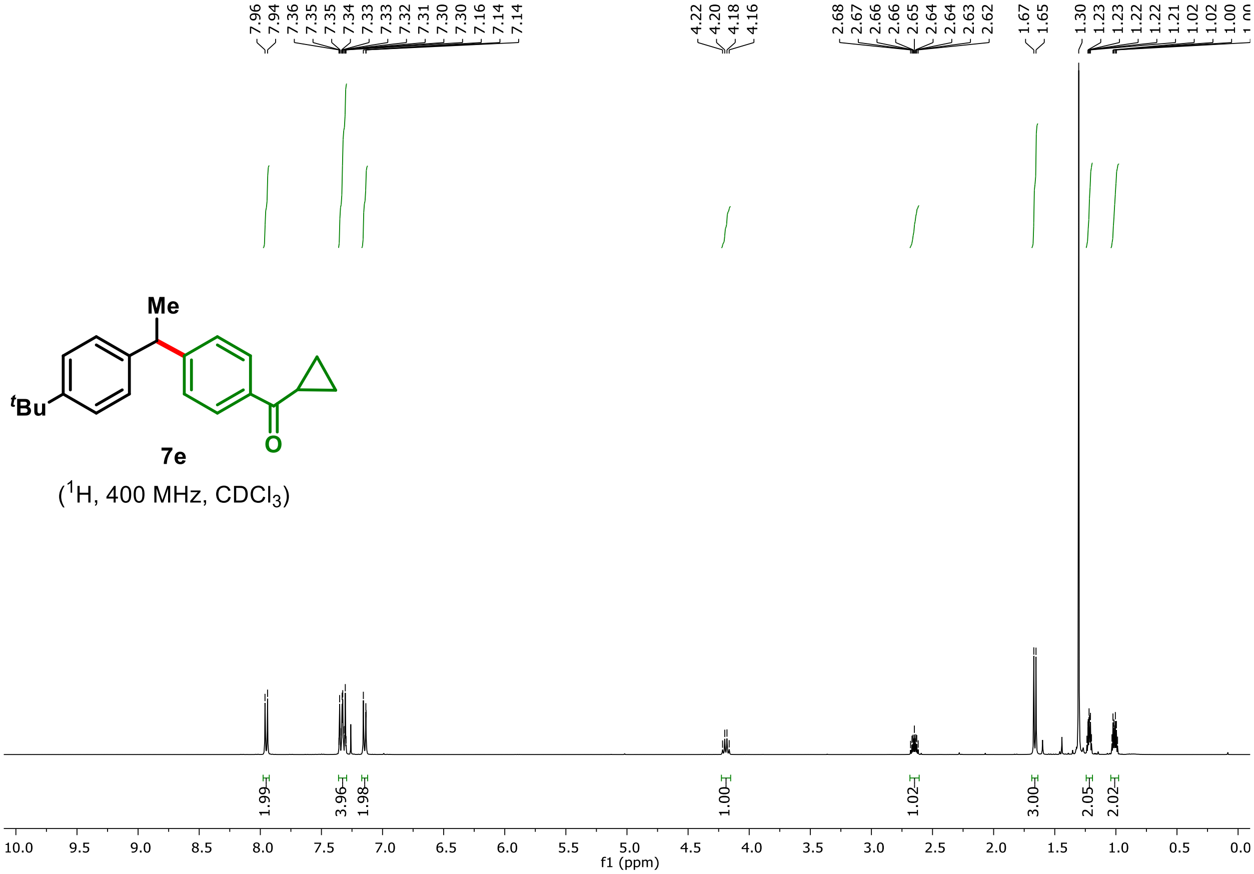

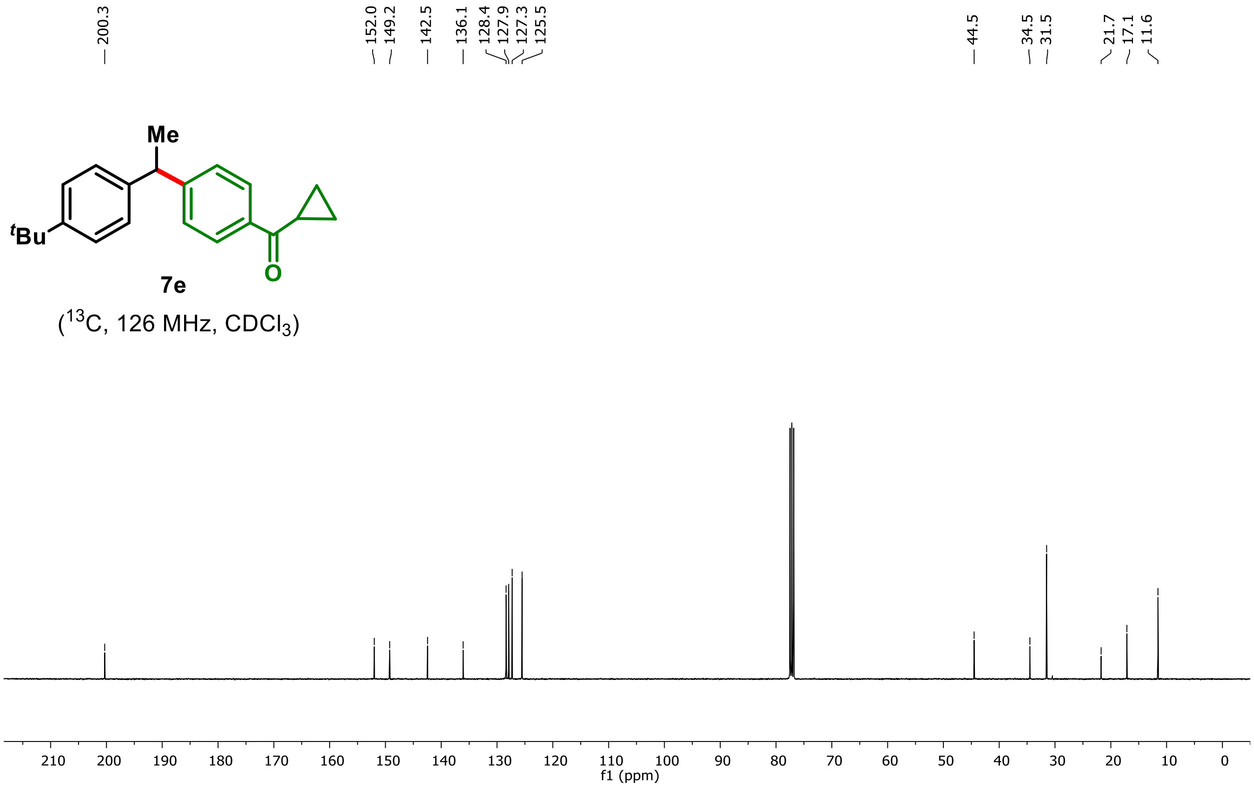

7e

(^{13}C, 126 MHz, $CDCl_3$)

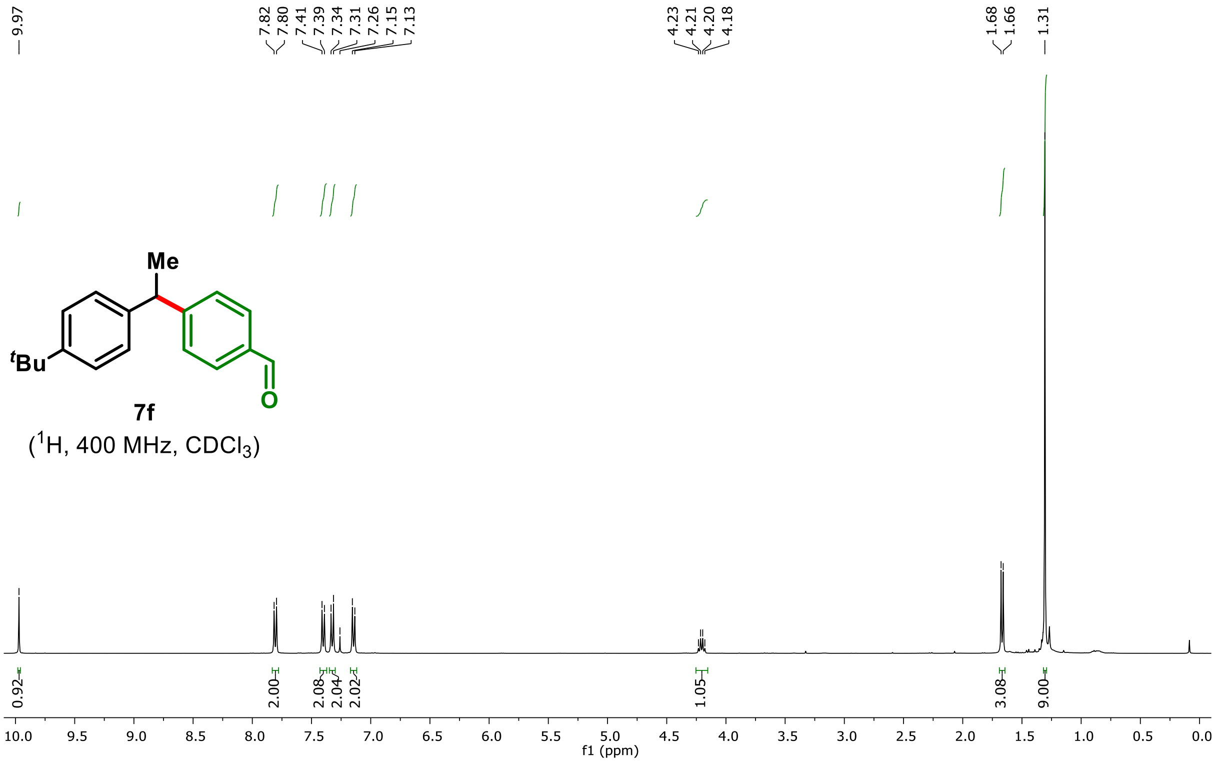

7f

(^{1}H, 400 MHz, $CDCl_3$)

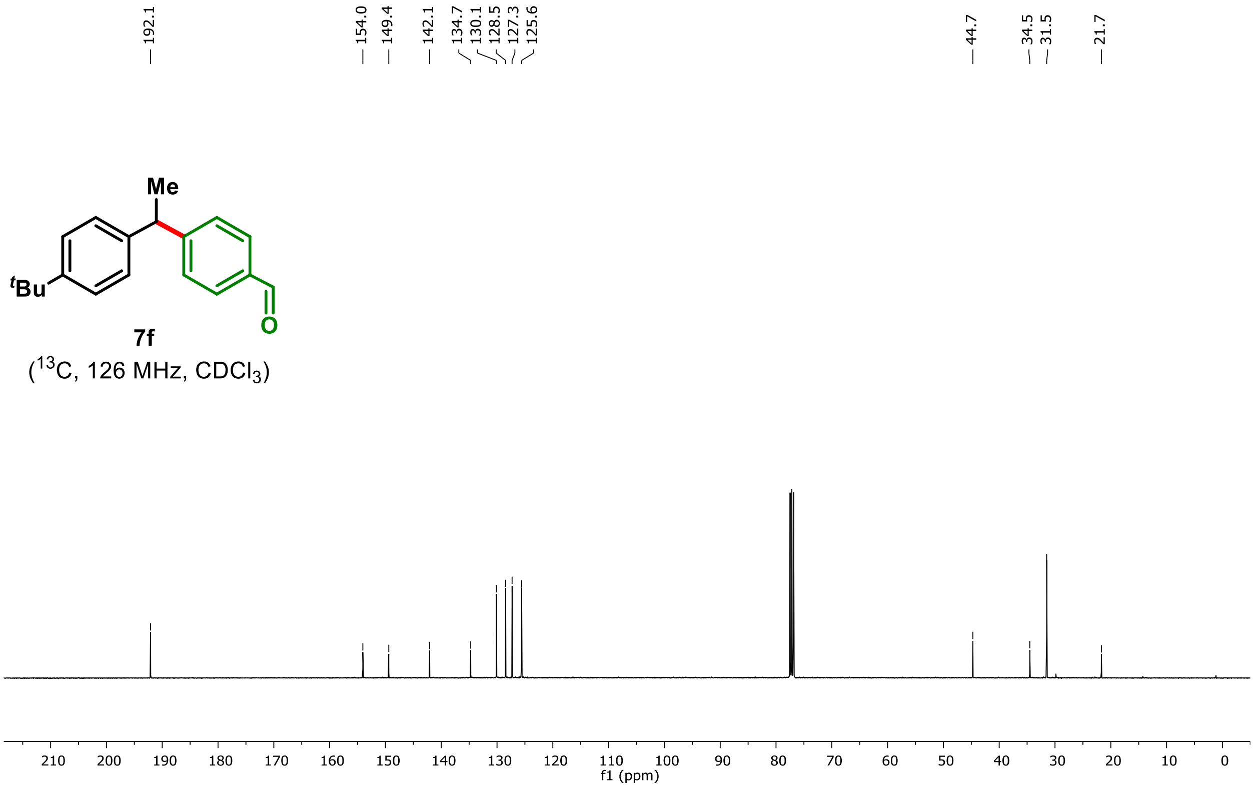

7f

(^{13}C, 126 MHz, $CDCl_3$)

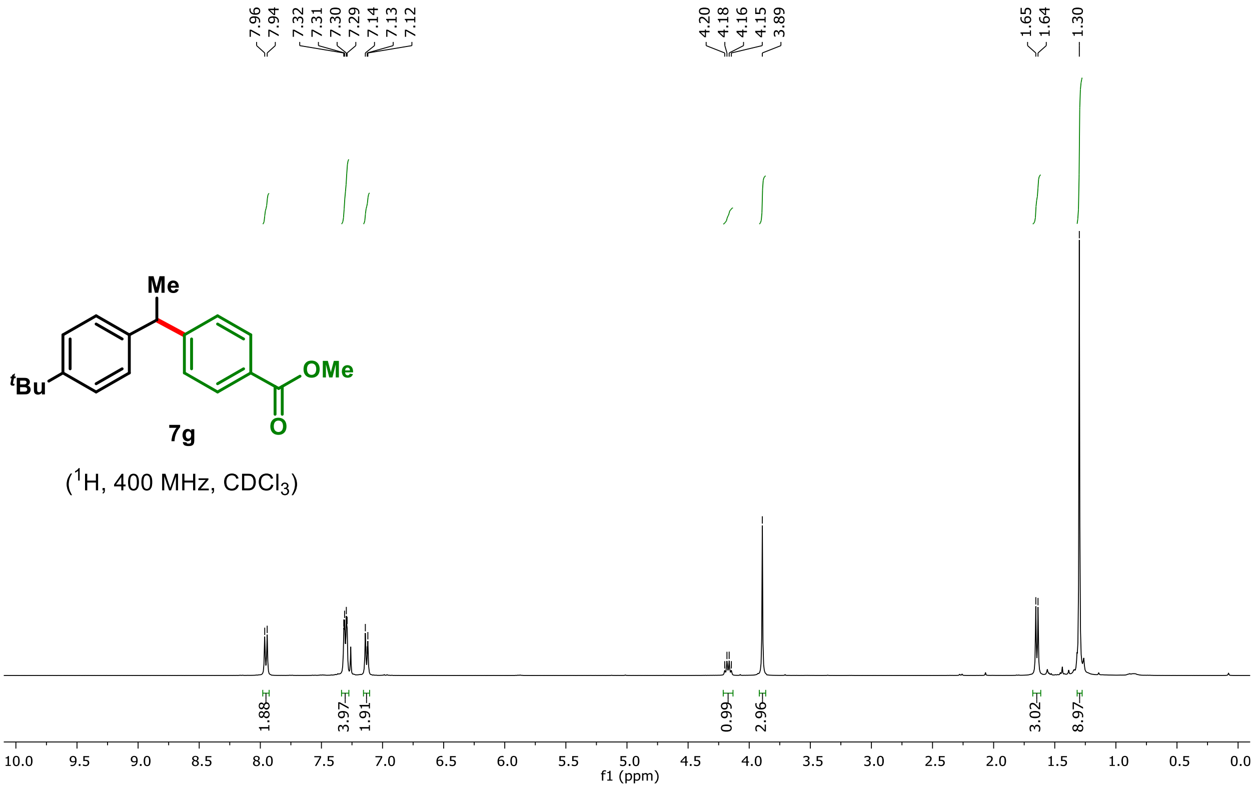

7g

(^{1}H, 400 MHz, $CDCl_3$)

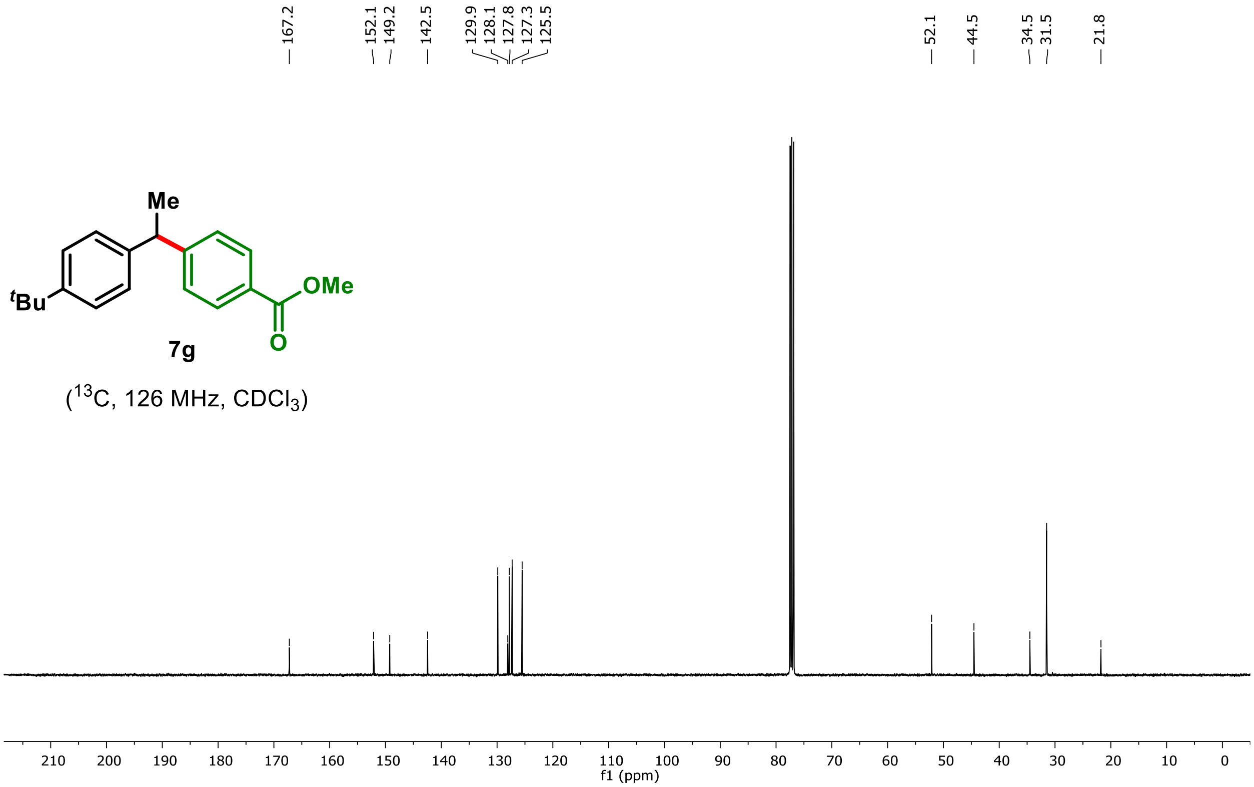

167.2
152.1
149.2
142.5
129.9
128.1
127.8
127.3
125.5
52.1
44.5
34.5
31.5
21.8
Me
tBu
OMe
O
7g
(13C, 126 MHz, CDCl3)
210 200 190 180 170 160 150 140 130 120 110 100 90 80 70 60 50 40 30 20 10 0
f1 (ppm)

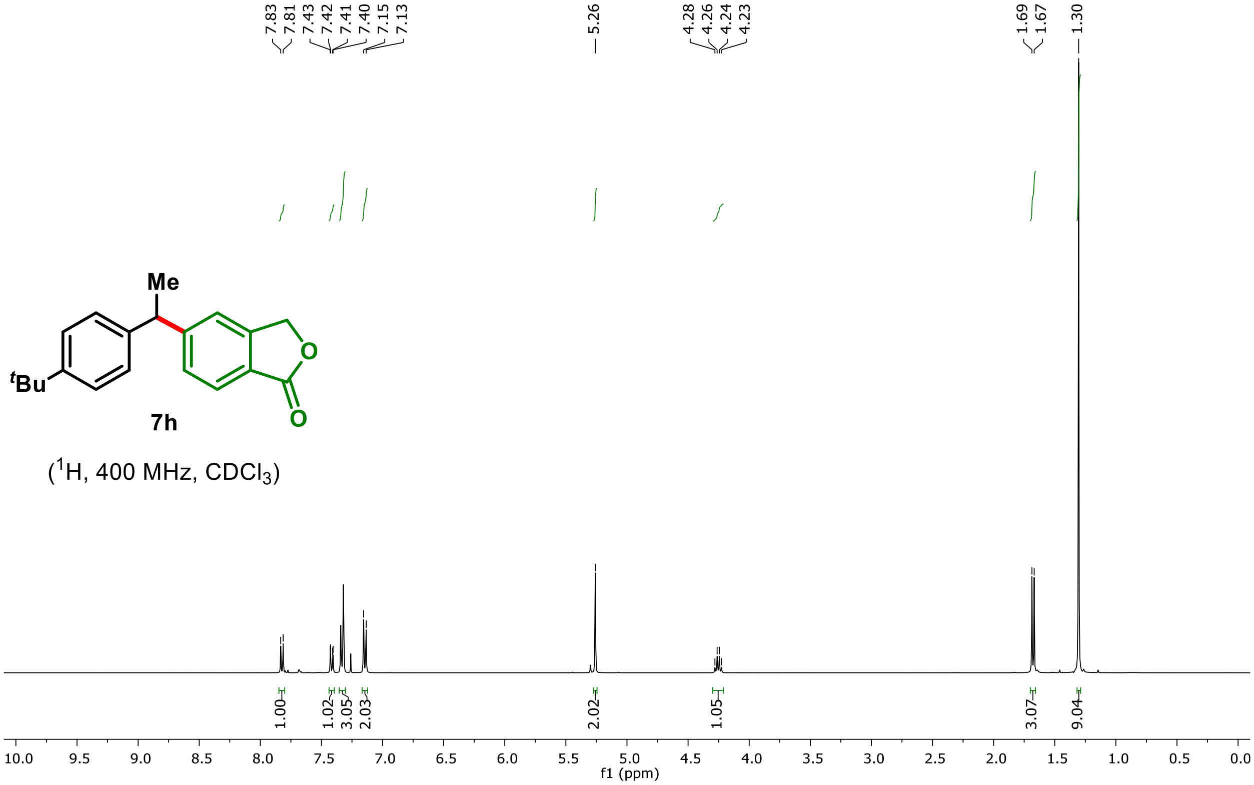

7.83
7.81
7.43
7.42
7.41
7.40
7.15
7.13
5.26
4.28
4.26
4.24
4.23
1.69
1.67
1.30
Me
tBu
O
O
7h
(1H, 400 MHz, CDCl3)
1.00
1.02
3.05
2.03
2.02
1.05
3.07
9.04
10.0 9.5 9.0 8.5 8.0 7.5 7.0 6.5 6.0 5.5 5.0 4.5 4.0 3.5 3.0 2.5 2.0 1.5 1.0 0.5 0.0
f1 (ppm)

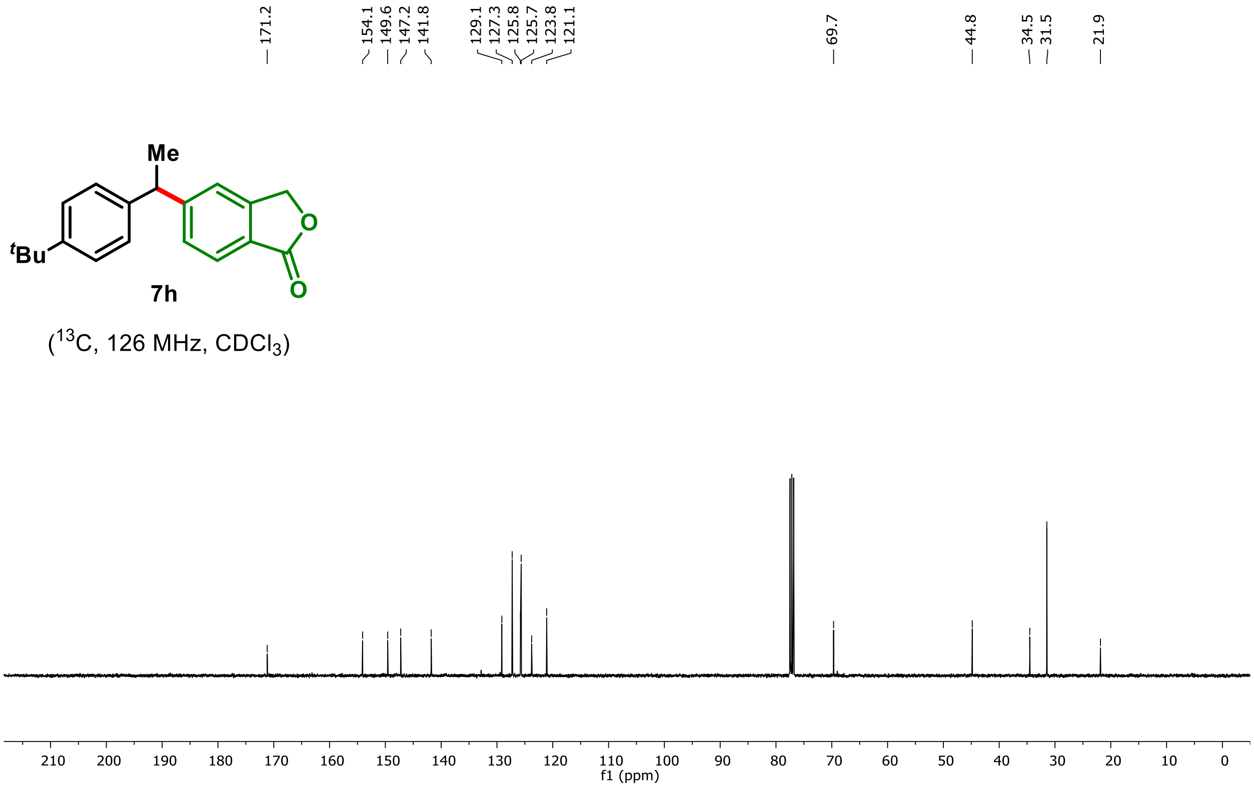
171.2
154.1
149.6
147.2
141.8
129.1
127.3
125.8
125.7
123.8
121.1
69.7
44.8
34.5
31.5
21.9
Me
^{t}Bu
O
O
7h
(^{13}C, 126 MHz, $CDCl_3$)
210 200 190 180 170 160 150 140 130 120 110 100 90 80 70 60 50 40 30 20 10 0
f1 (ppm)

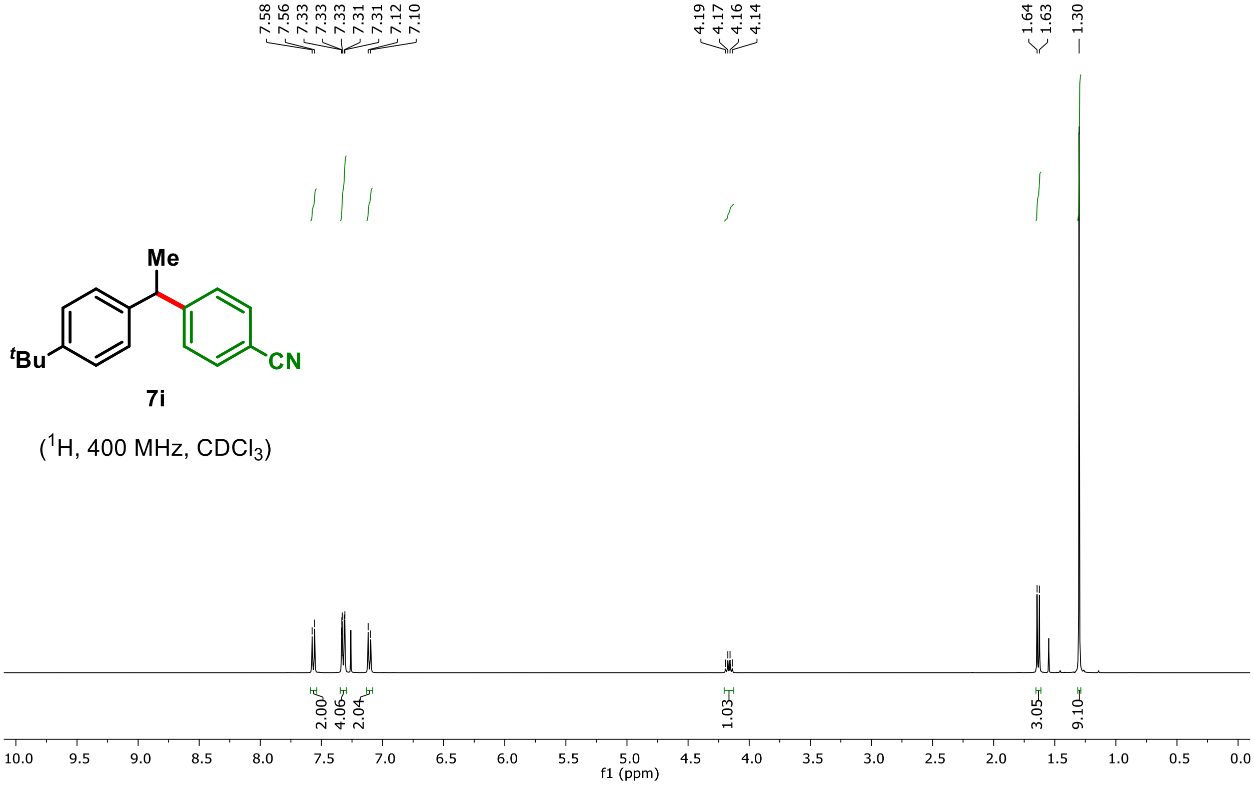
7.58
7.56
7.33
7.33
7.33
7.31
7.31
7.12
7.10
4.19
4.17
4.16
4.14
1.64
1.63
1.30
Me
^{t}Bu
CN
7i
(^{1}H, 400 MHz, $CDCl_3$)
2.00
4.06
2.04
1.03
3.05
9.10
10.0 9.5 9.0 8.5 8.0 7.5 7.0 6.5 6.0 5.5 5.0 4.5 4.0 3.5 3.0 2.5 2.0 1.5 1.0 0.5 0.0
f1 (ppm)

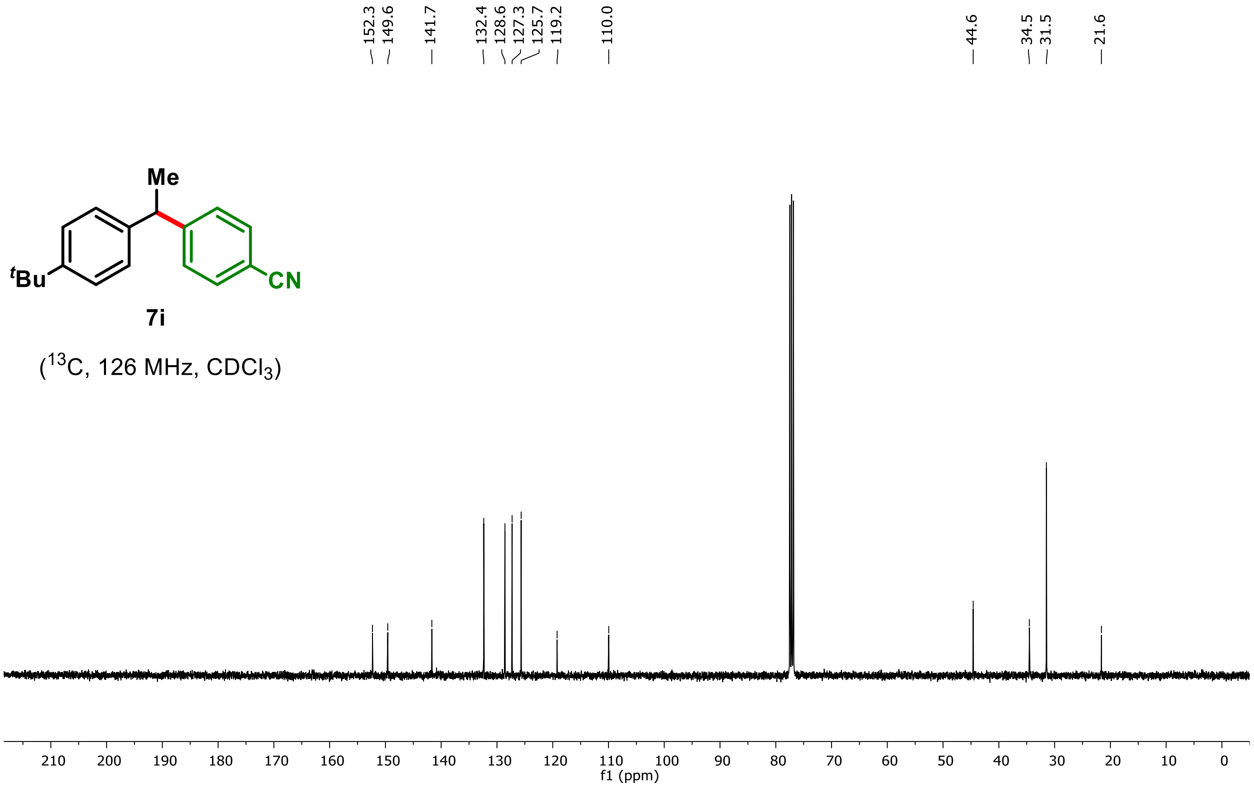
152.3
149.6
141.7
132.4
128.6
127.3
125.7
119.2
110.0
44.6
34.5
31.5
21.6
Me
tBu
CN
7i
(13C, 126 MHz, CDCl3)
f1 (ppm)

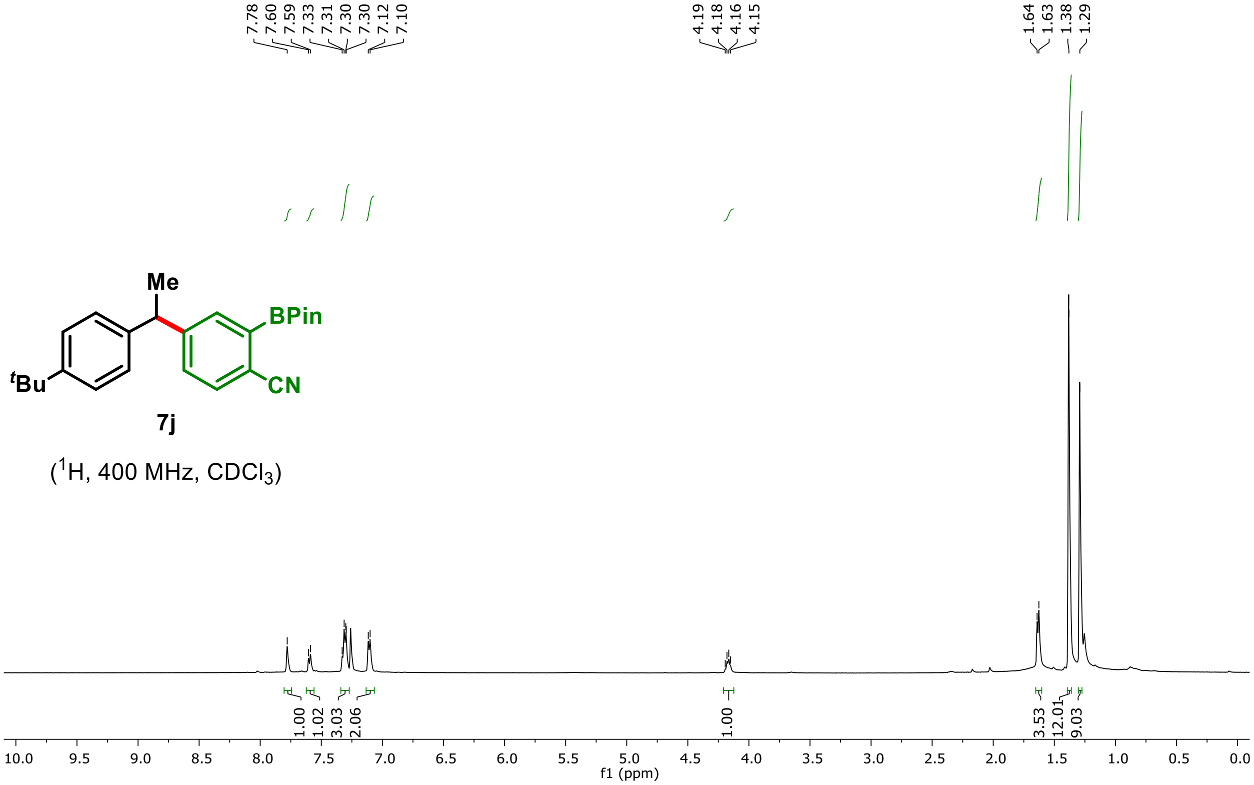
7.78
7.60
7.59
7.33
7.31
7.30
7.30
7.12
7.10
4.19
4.18
4.16
4.15
1.64
1.63
1.38
1.29
Me
BPin
tBu
CN
7j
(1H, 400 MHz, CDCl3)
1.00
1.02
3.03
2.06
1.00
3.53
12.01
9.03
f1 (ppm)

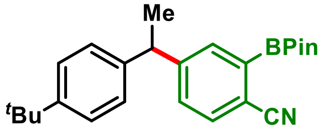

7j

(^{13}C, 126 MHz, $CDCl_3$)

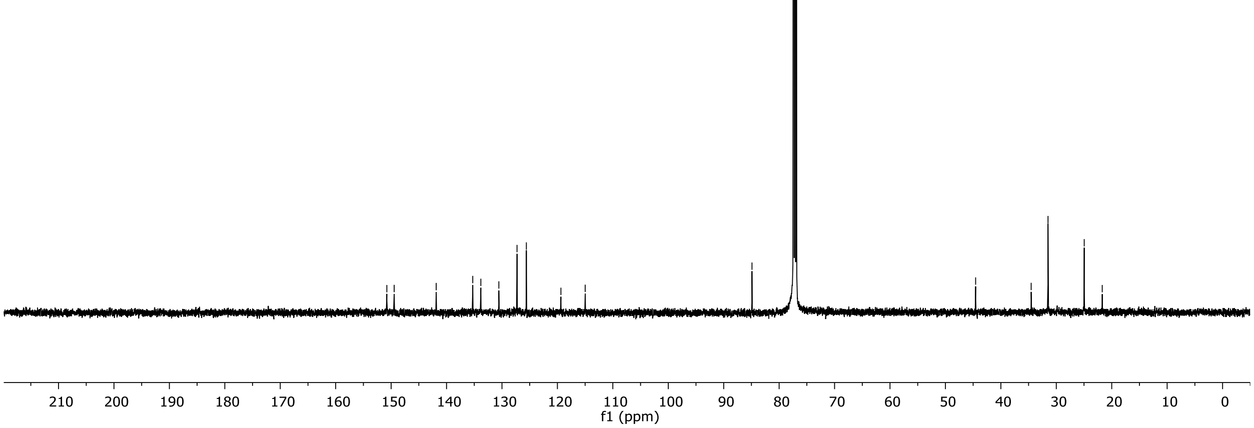

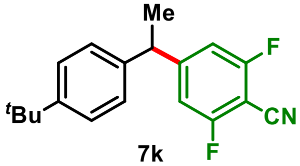

7k

(^{1}H, 400 MHz, $CDCl_3$)

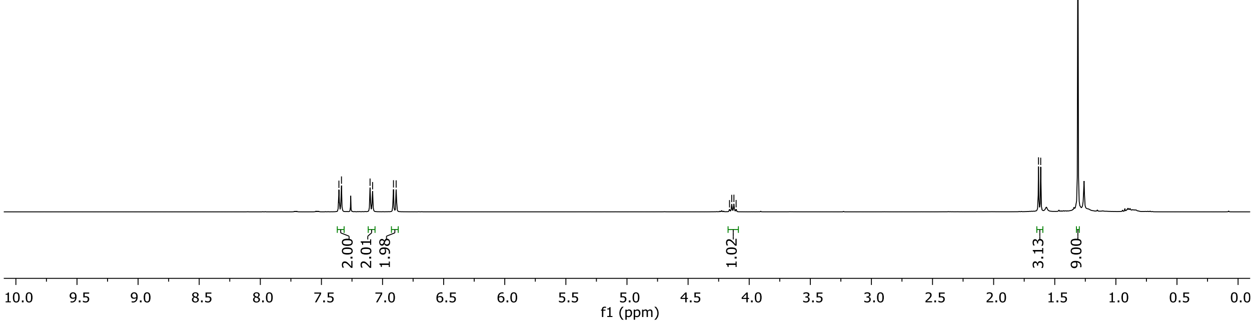

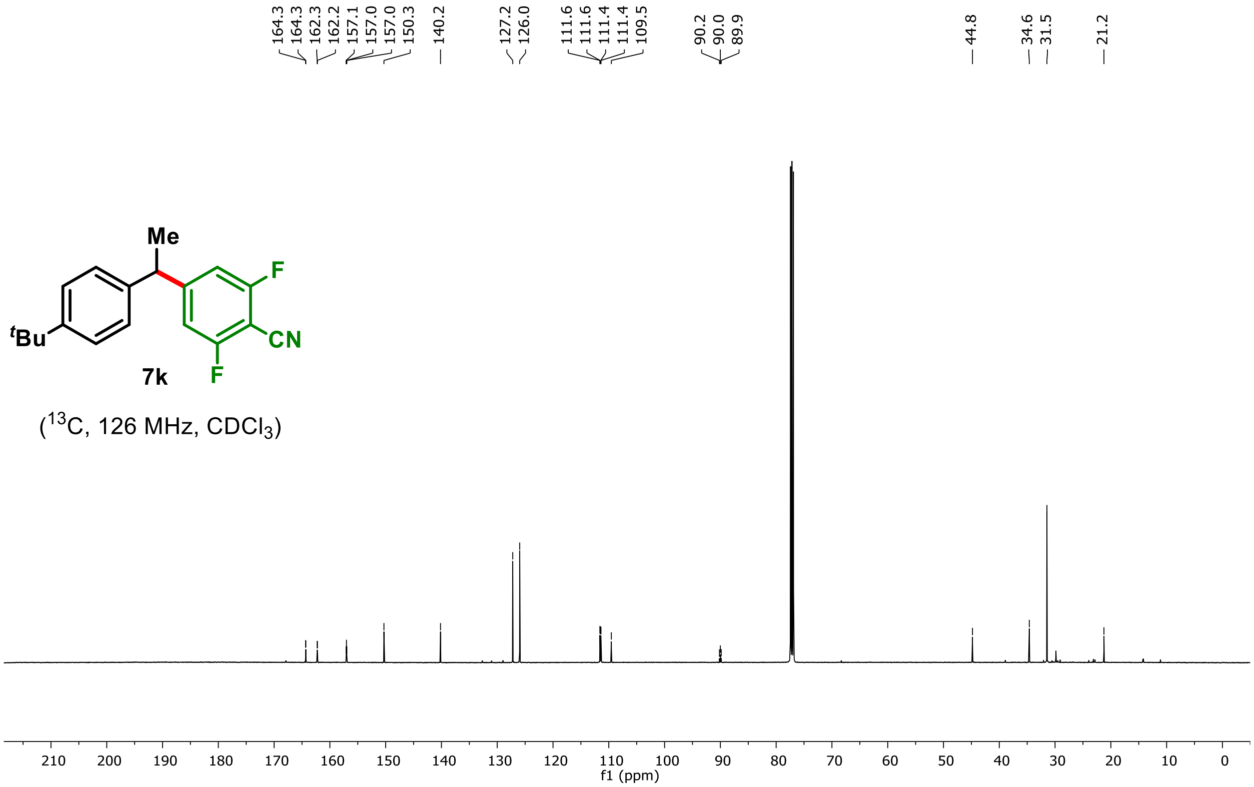

7k

(^{13}C, 126 MHz, $CDCl_3$)

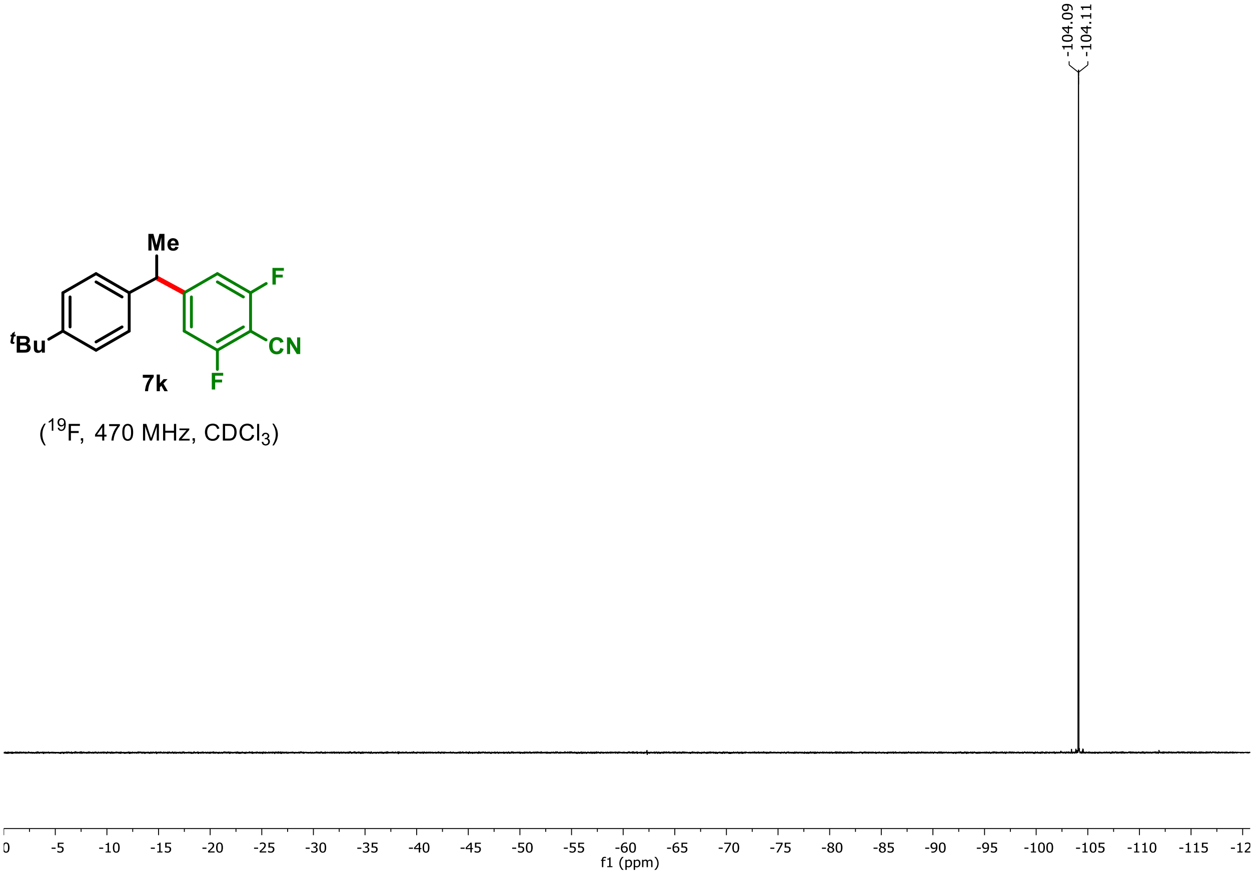

7k

(^{19}F, 470 MHz, $CDCl_3$)

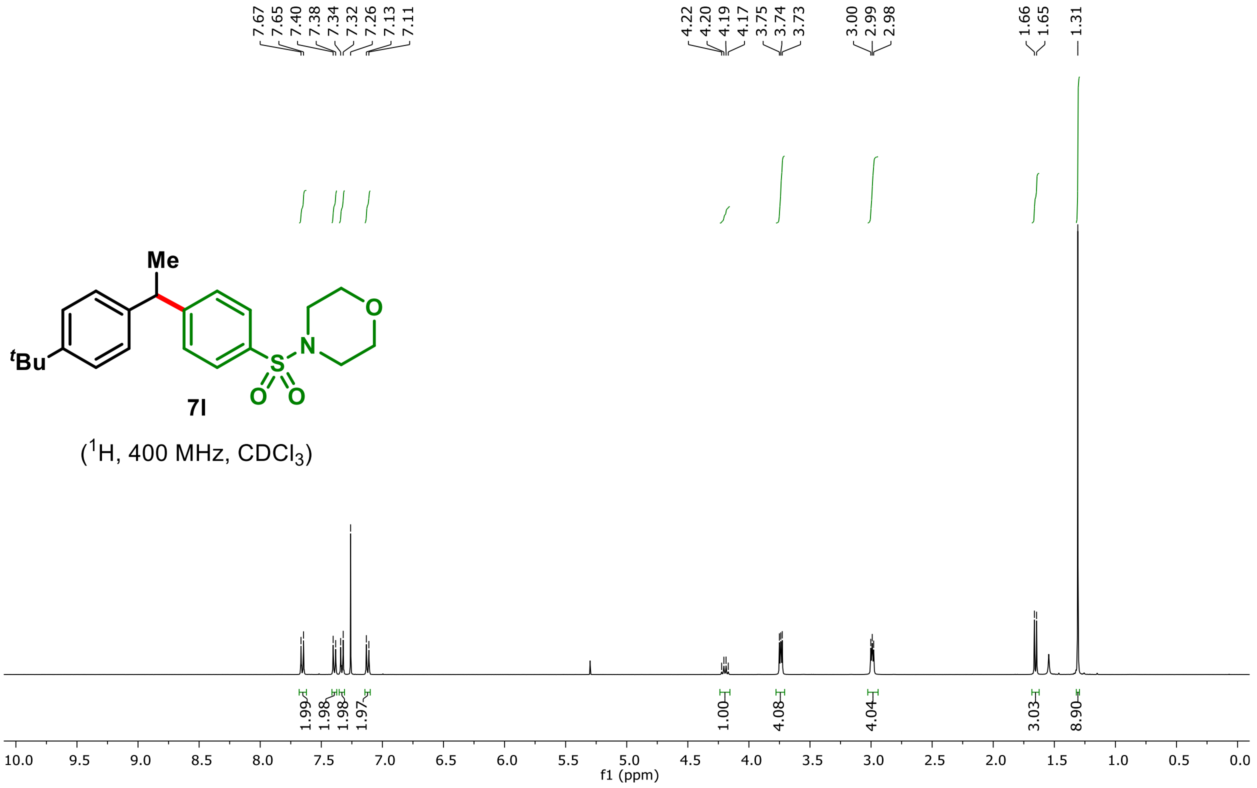
7.67
7.65
7.40
7.38
7.34
7.32
7.26
7.13
7.11
4.22
4.20
4.19
4.17
3.75
3.74
3.73
3.00
2.99
2.98
1.66
1.65
1.31
Me
tBu
N
O
S
O
O
7l
(1H, 400 MHz, CDCl3)
1.99
1.98
1.98
1.97
1.00
4.08
4.04
3.03
8.90
f1 (ppm)

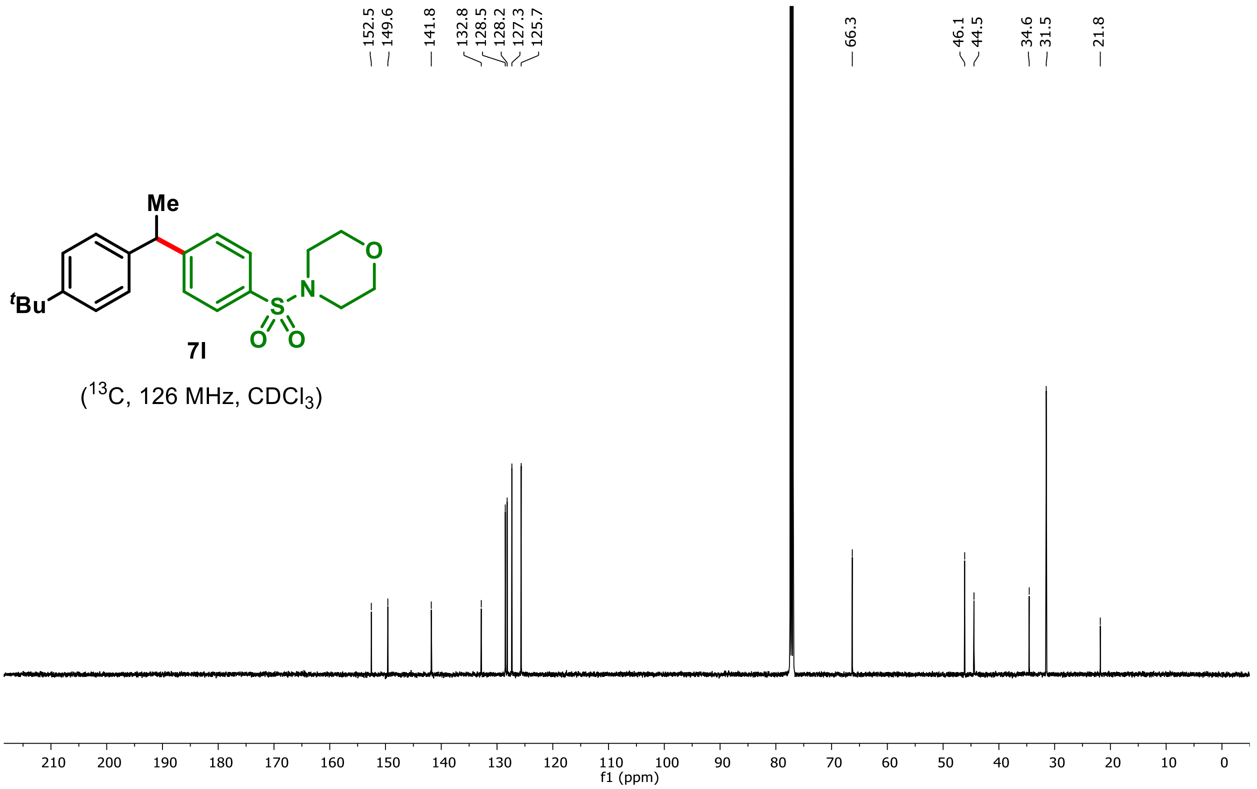
152.5
149.6
141.8
132.8
128.5
128.2
127.3
125.7
66.3
46.1
44.5
34.6
31.5
21.8
Me
tBu
N
O
S
O
O
7l
(13C, 126 MHz, CDCl3)
f1 (ppm)

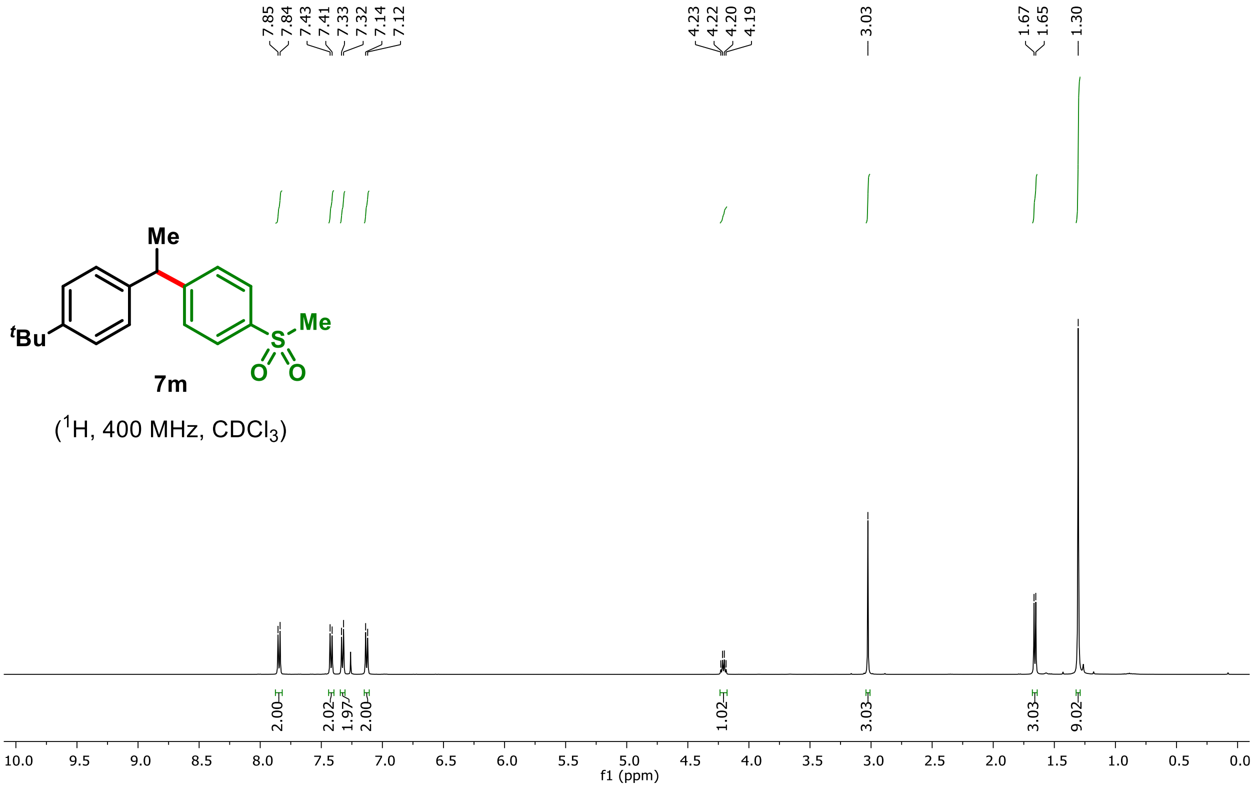
Me
tBu
S
Me
O O
7m
(1H, 400 MHz, CDCl3)
7.85 7.84 7.43 7.41 7.33 7.32 7.14 7.12
4.23 4.22 4.20 4.19
3.03
1.67 1.65
1.30
2.00 2.02 1.97 2.00 1.02 3.03 3.03 9.02
10.0 9.5 9.0 8.5 8.0 7.5 7.0 6.5 6.0 5.5 5.0 4.5 4.0 3.5 3.0 2.5 2.0 1.5 1.0 0.5 0.0
f1 (ppm)

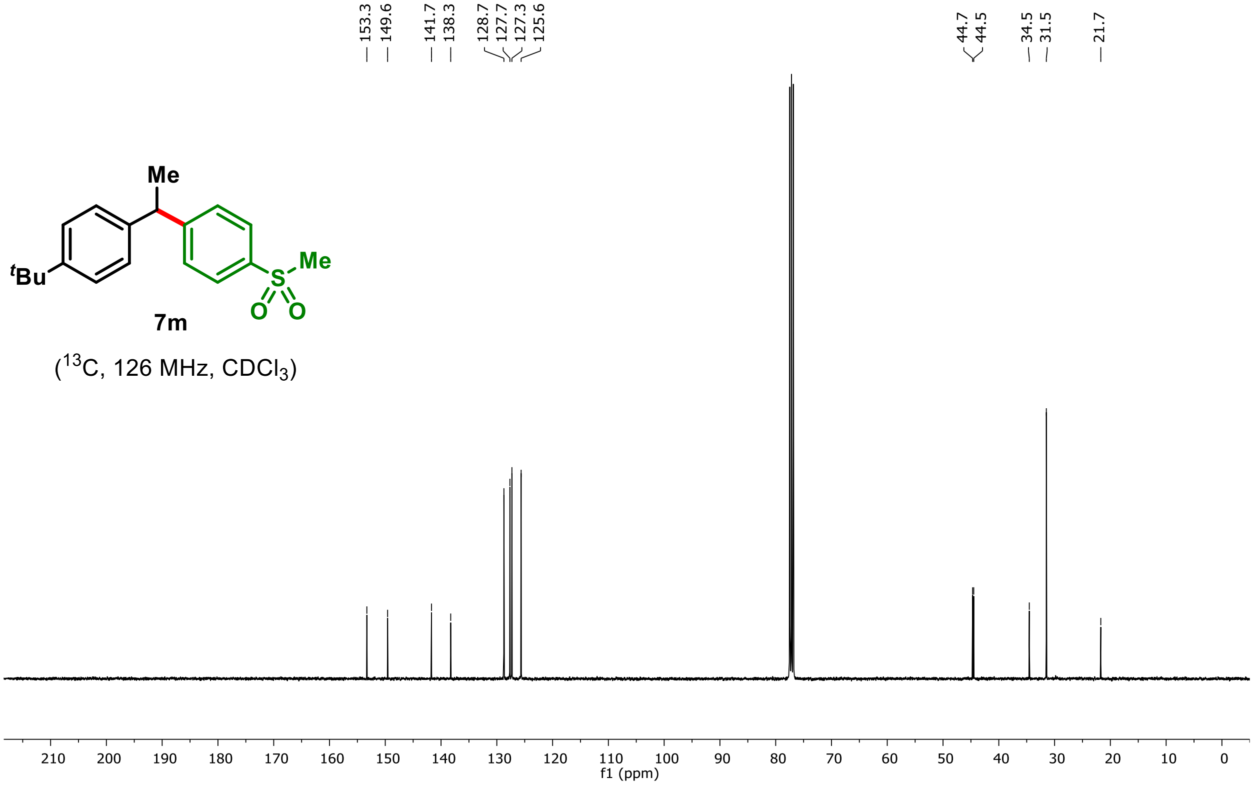
Me
tBu
S
Me
O O
7m
(13C, 126 MHz, CDCl3)
153.3 149.6 141.7 138.3 128.7 127.7 127.3 125.6
44.7 44.5 34.5 31.5 21.7
210 200 190 180 170 160 150 140 130 120 110 100 90 80 70 60 50 40 30 20 10 0
f1 (ppm)

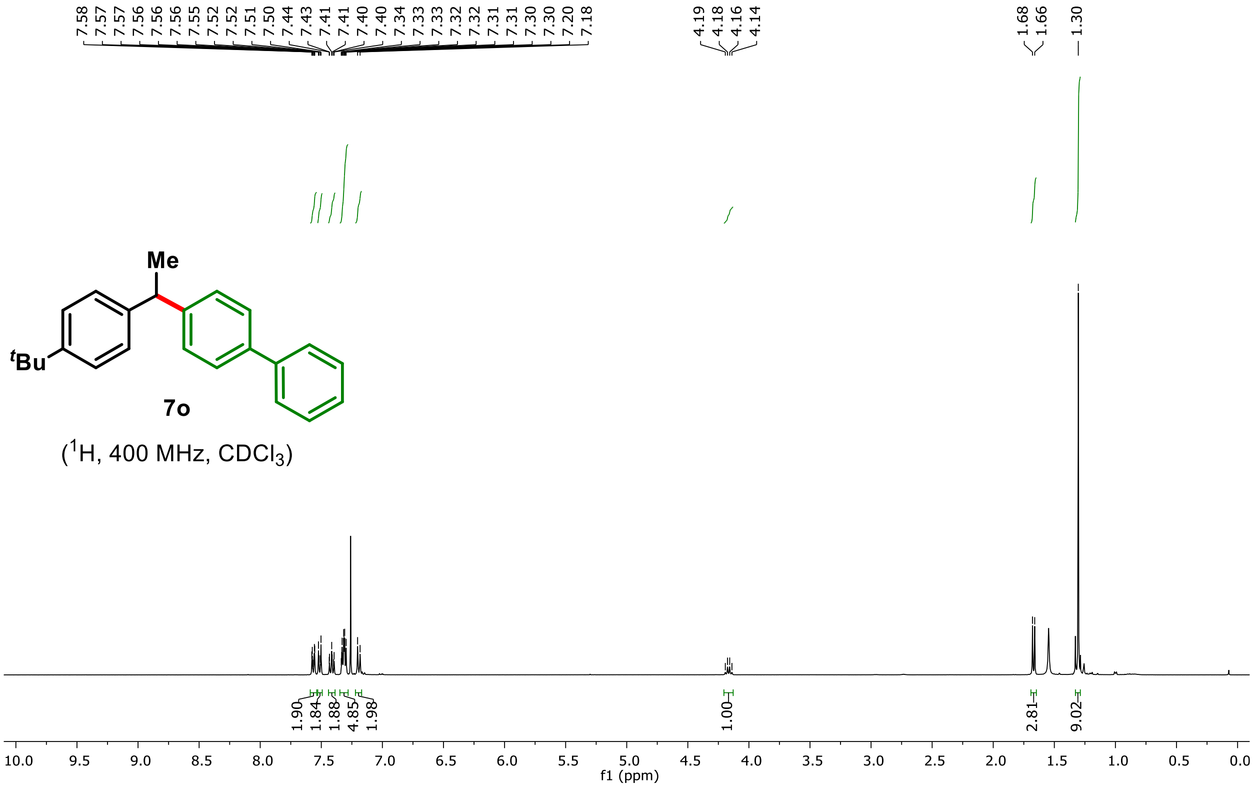
7.58 7.57 7.57 7.56 7.56 7.55 7.52 7.52 7.51 7.50 7.44 7.43 7.41 7.41 7.40 7.40 7.34 7.33 7.33 7.32 7.32 7.31 7.31 7.30 7.30 7.20 7.18
4.19 4.18 4.16 4.14
1.68 1.66
1.30
Me
tBu
7o
(1H, 400 MHz, CDCl3)
1.90 1.84 1.88 4.85 1.98
1.00
2.81
9.02
10.0 9.5 9.0 8.5 8.0 7.5 7.0 6.5 6.0 5.5 5.0 4.5 4.0 3.5 3.0 2.5 2.0 1.5 1.0 0.5 0.0
f1 (ppm)

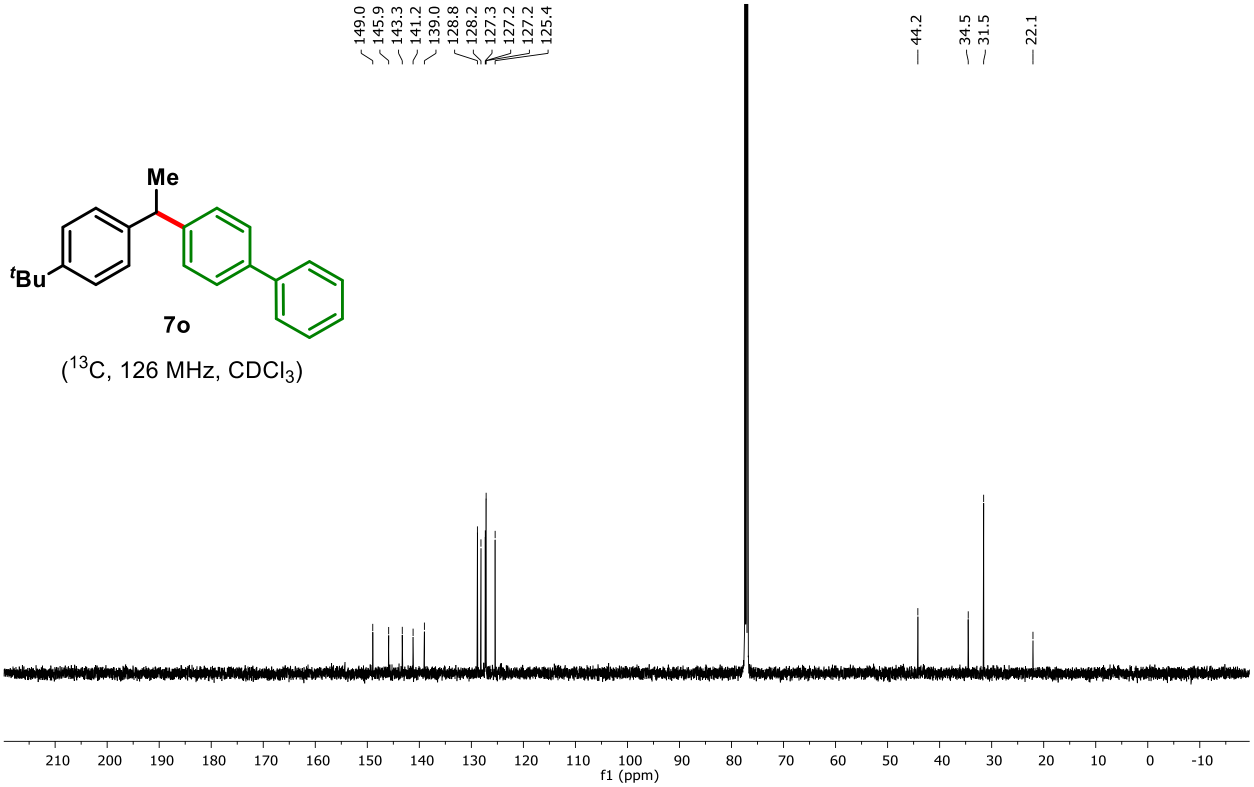
149.0 145.9 143.3 141.2 139.0 128.8 128.2 127.3 127.2 127.2 125.4
44.2
34.5
31.5
22.1
Me
tBu
7o
(13C, 126 MHz, CDCl3)
210 200 190 180 170 160 150 140 130 120 110 100 90 80 70 60 50 40 30 20 10 0 -10
f1 (ppm)

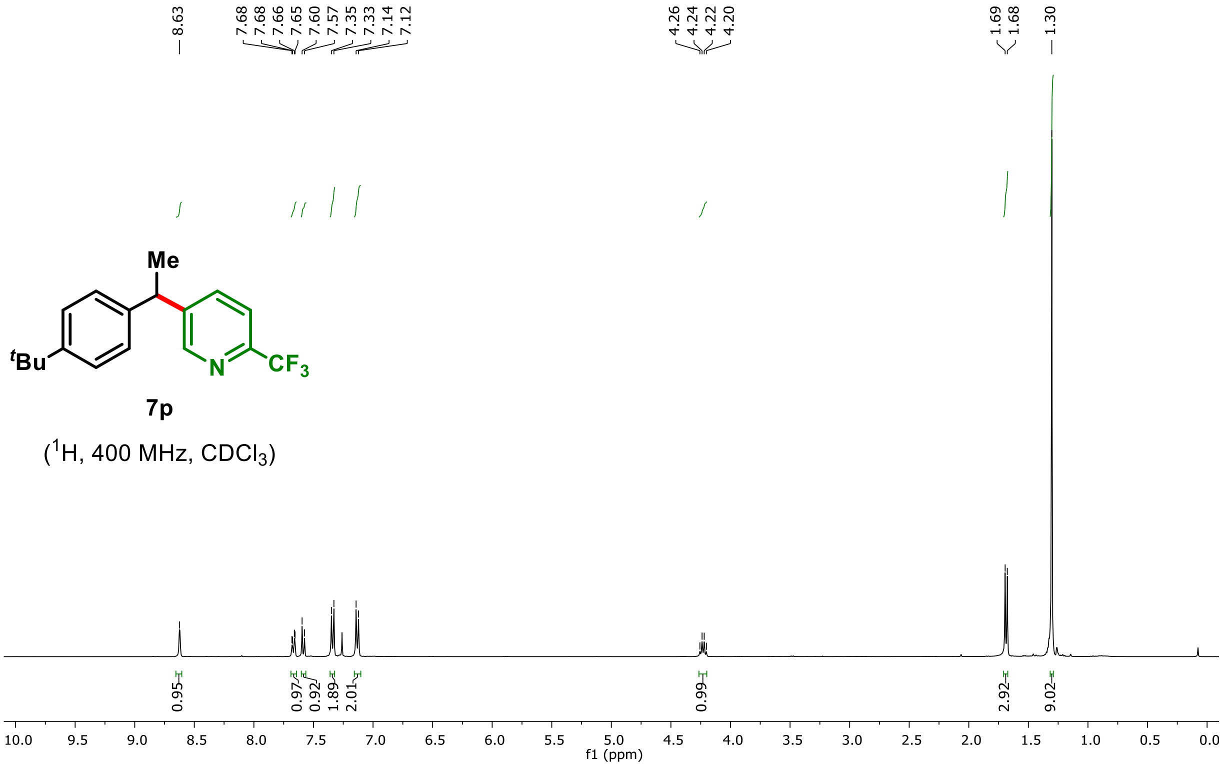
8.63
7.68 7.68 7.66 7.65 7.60 7.57 7.35 7.33 7.14 7.12
4.26 4.24 4.22 4.20
1.69 1.68
1.30
Me
tBu
N
CF3
7p
(1H, 400 MHz, CDCl3)
0.95 0.97 0.92 1.89 2.01 0.99 2.92 9.02
f1 (ppm)

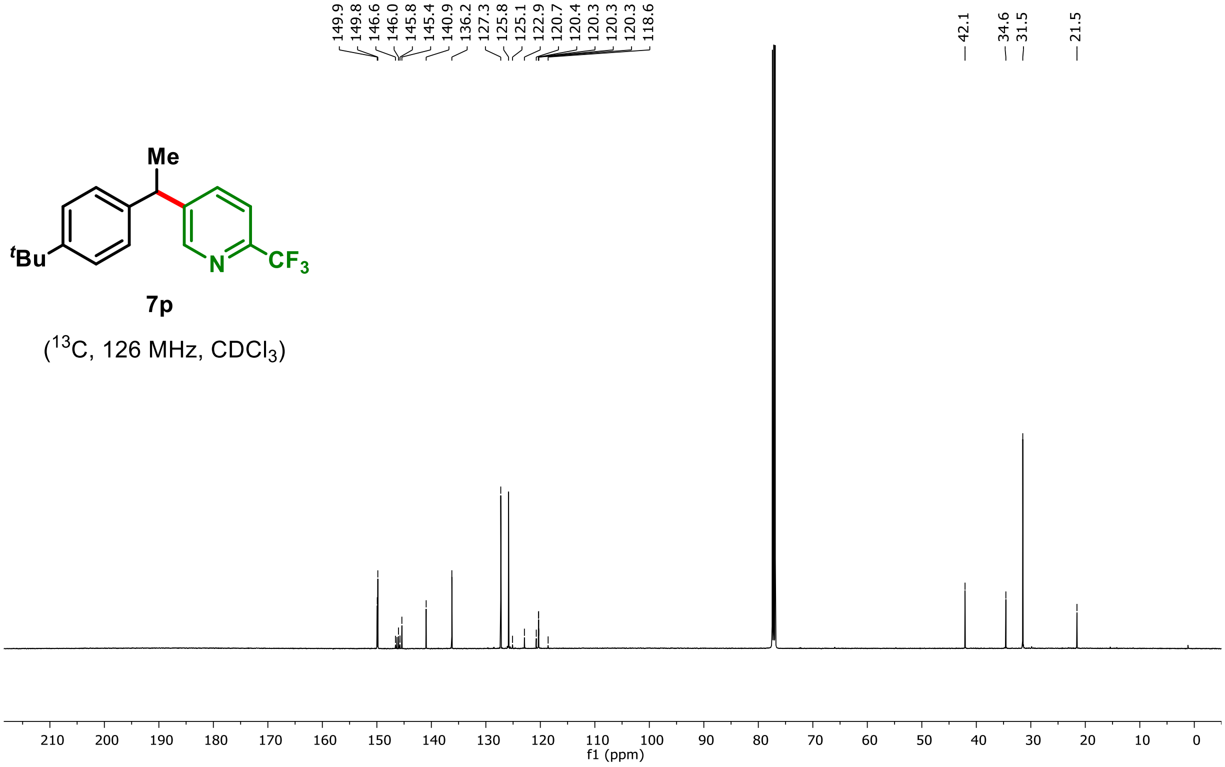
149.9 149.8 146.6 146.0 145.8 145.4 140.9 136.2 127.3 125.8 125.1 122.9 120.7 120.4 120.3 120.3 120.3 118.6
42.1
34.6
31.5
21.5
Me
tBu
N
CF3
7p
(13C, 126 MHz, CDCl3)
f1 (ppm)

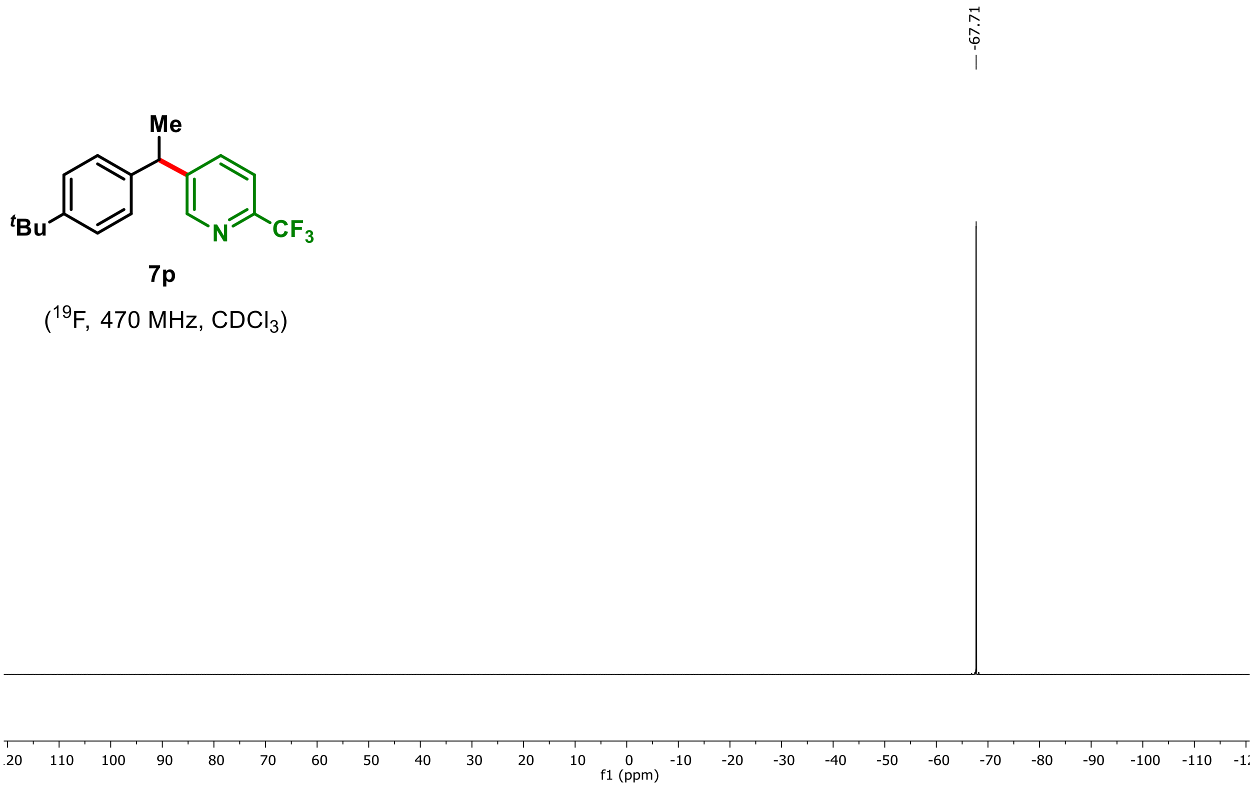

7p

(^{19}F, 470 MHz, $CDCl_3$)

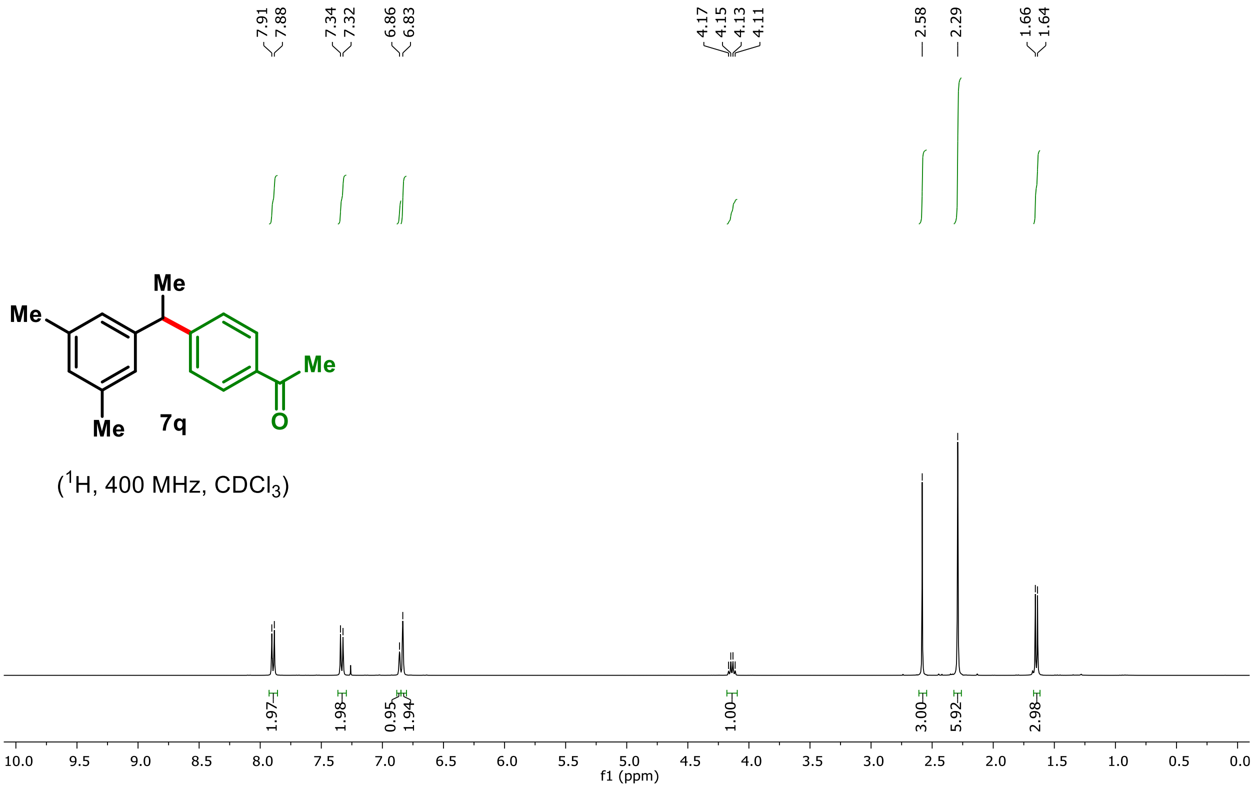

7q

(^{1}H, 400 MHz, $CDCl_3$)

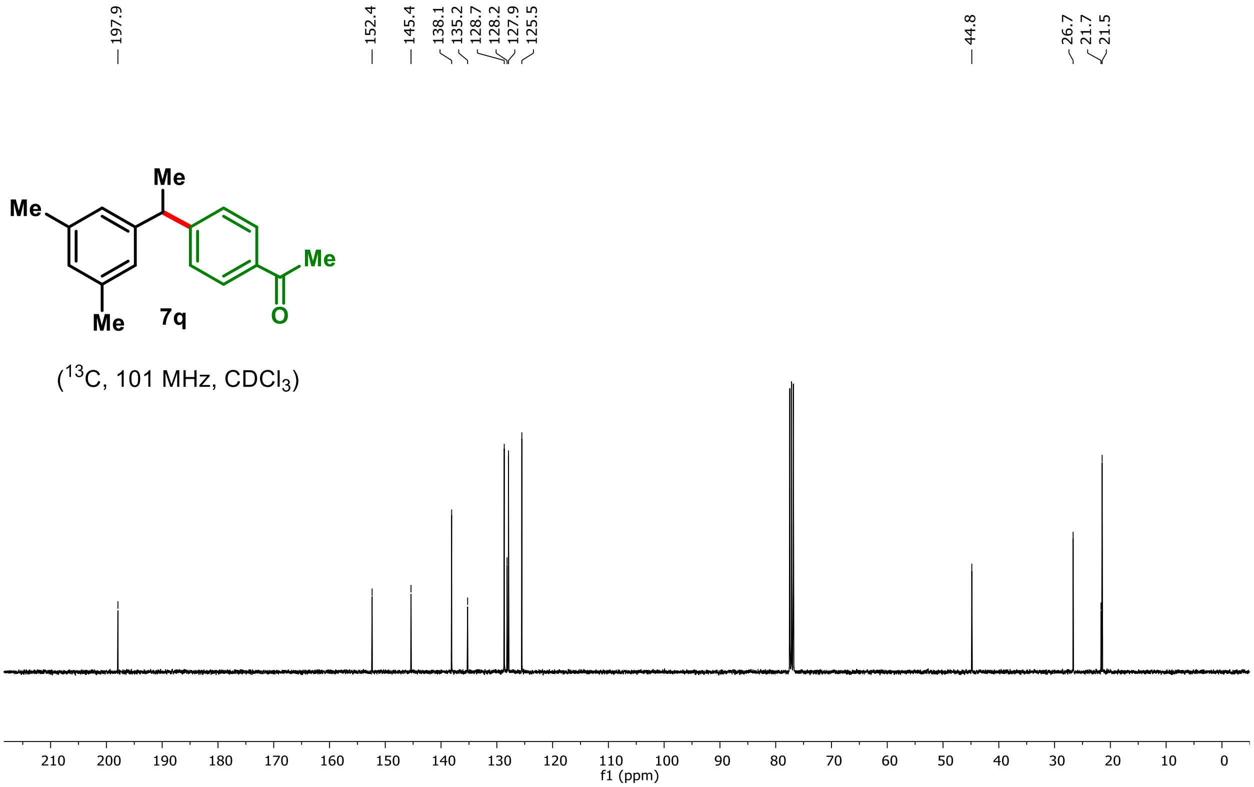
197.9
152.4
145.4
138.1
135.2
128.7
128.2
127.9
125.5
44.8
26.7
21.7
21.5
Me
Me
Me
Me
O
7q
(^{13}C, 101 MHz, $CDCl_3$)
210 200 190 180 170 160 150 140 130 120 110 100 90 80 70 60 50 40 30 20 10 0
f1 (ppm)

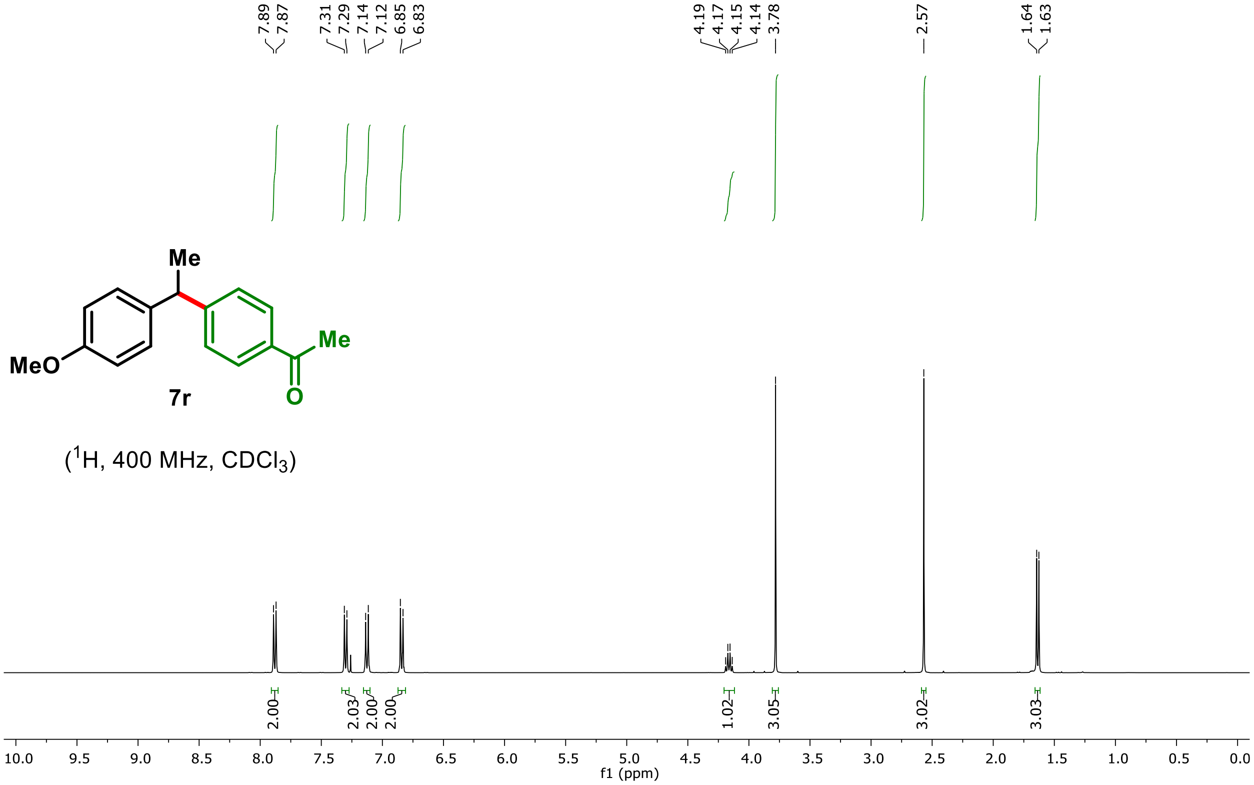
7.89
7.87
7.31
7.29
7.14
7.12
6.85
6.83
4.19
4.17
4.15
4.14
3.78
2.57
1.64
1.63
Me
MeO
Me
O
7r
(^{1}H, 400 MHz, $CDCl_3$)
2.00
2.03
2.00
2.00
1.02
3.05
3.02
3.03
10.0 9.5 9.0 8.5 8.0 7.5 7.0 6.5 6.0 5.5 5.0 4.5 4.0 3.5 3.0 2.5 2.0 1.5 1.0 0.5 0.0
f1 (ppm)

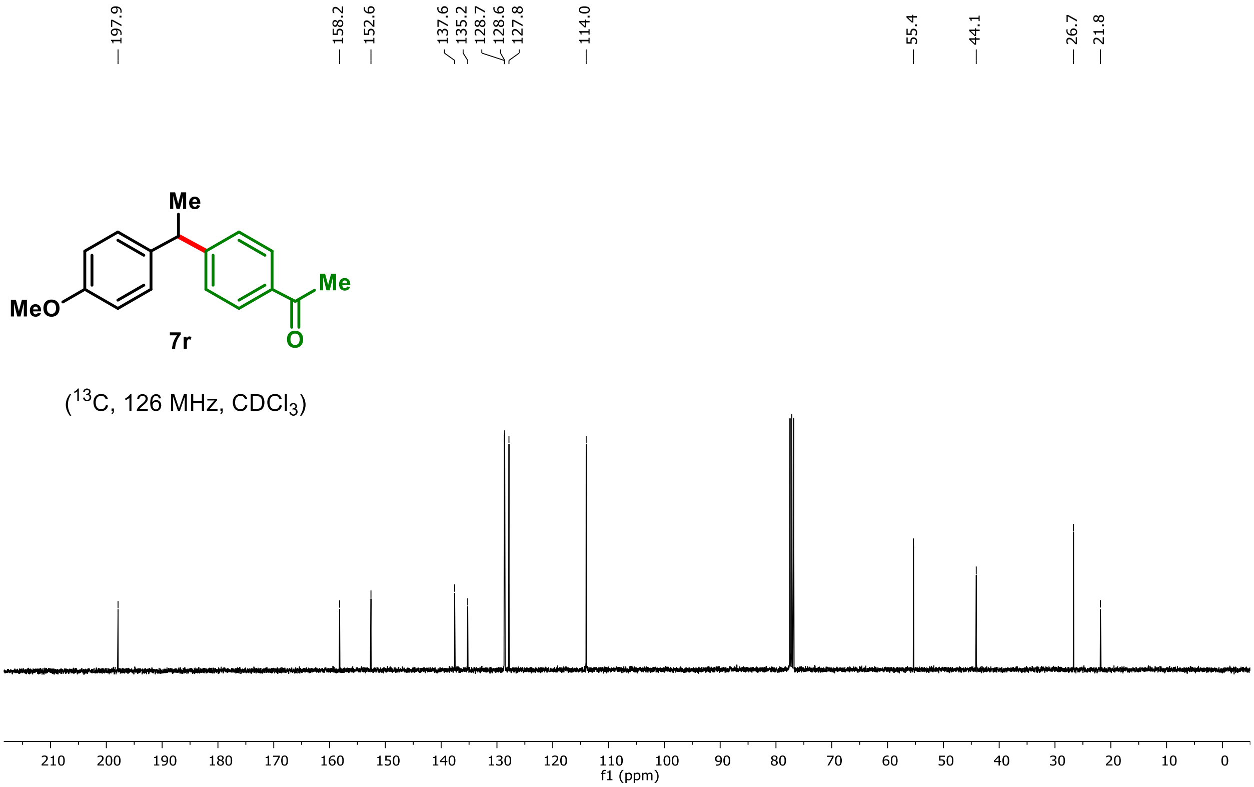

197.9
158.2
152.6
137.6
135.2
128.7
128.6
127.8
114.0
55.4
44.1
26.7
21.8
Me
MeO
Me
O
7r
(^{13}C, 126 MHz, $CDCl_3$)
210 200 190 180 170 160 150 140 130 120 110 100 90 80 70 60 50 40 30 20 10 0
f1 (ppm)

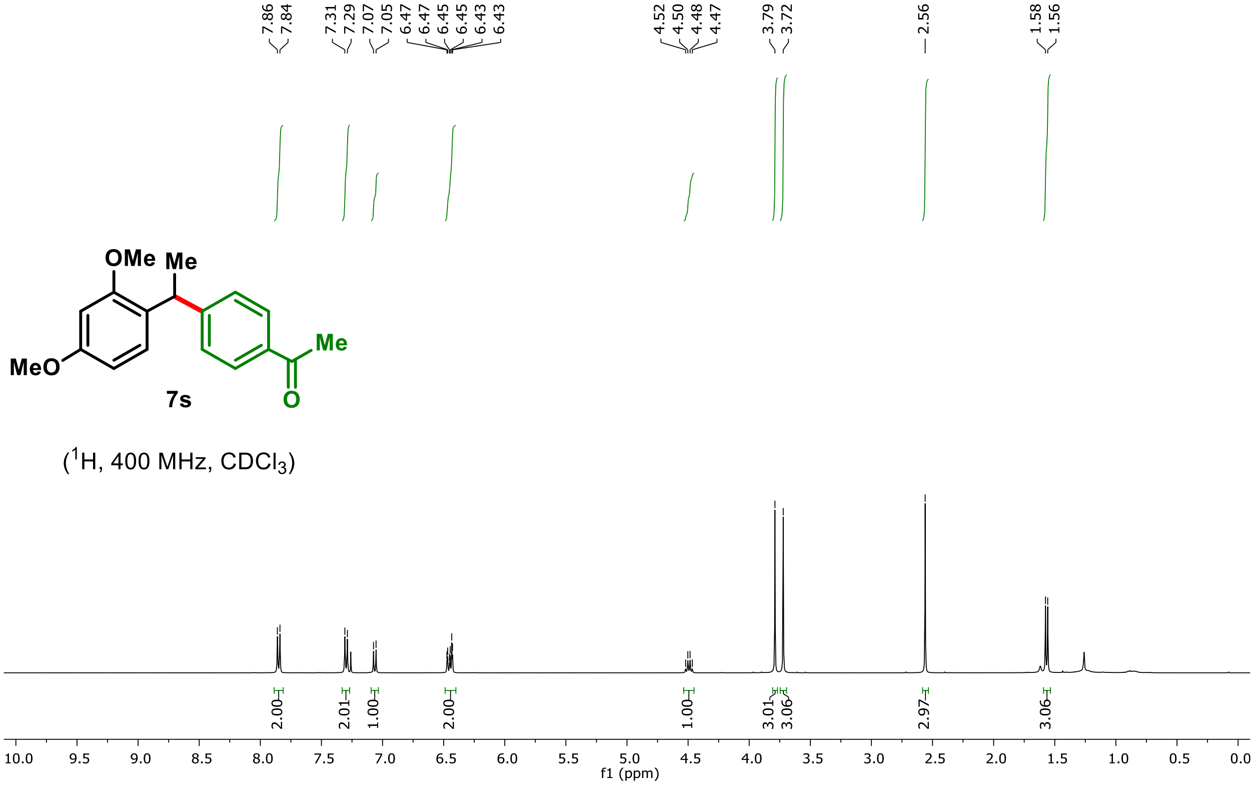

7.86
7.84
7.31
7.29
7.07
7.05
6.47
6.47
6.45
6.45
6.43
6.43
4.52
4.50
4.48
4.47
3.79
3.72
2.56
1.58
1.56
OMe
Me
MeO
Me
O
7s
(^{1}H, 400 MHz, $CDCl_3$)
2.00
2.01
1.00
2.00
1.00
3.01
3.06
2.97
3.06
10.0 9.5 9.0 8.5 8.0 7.5 7.0 6.5 6.0 5.5 5.0 4.5 4.0 3.5 3.0 2.5 2.0 1.5 1.0 0.5 0.0
f1 (ppm)

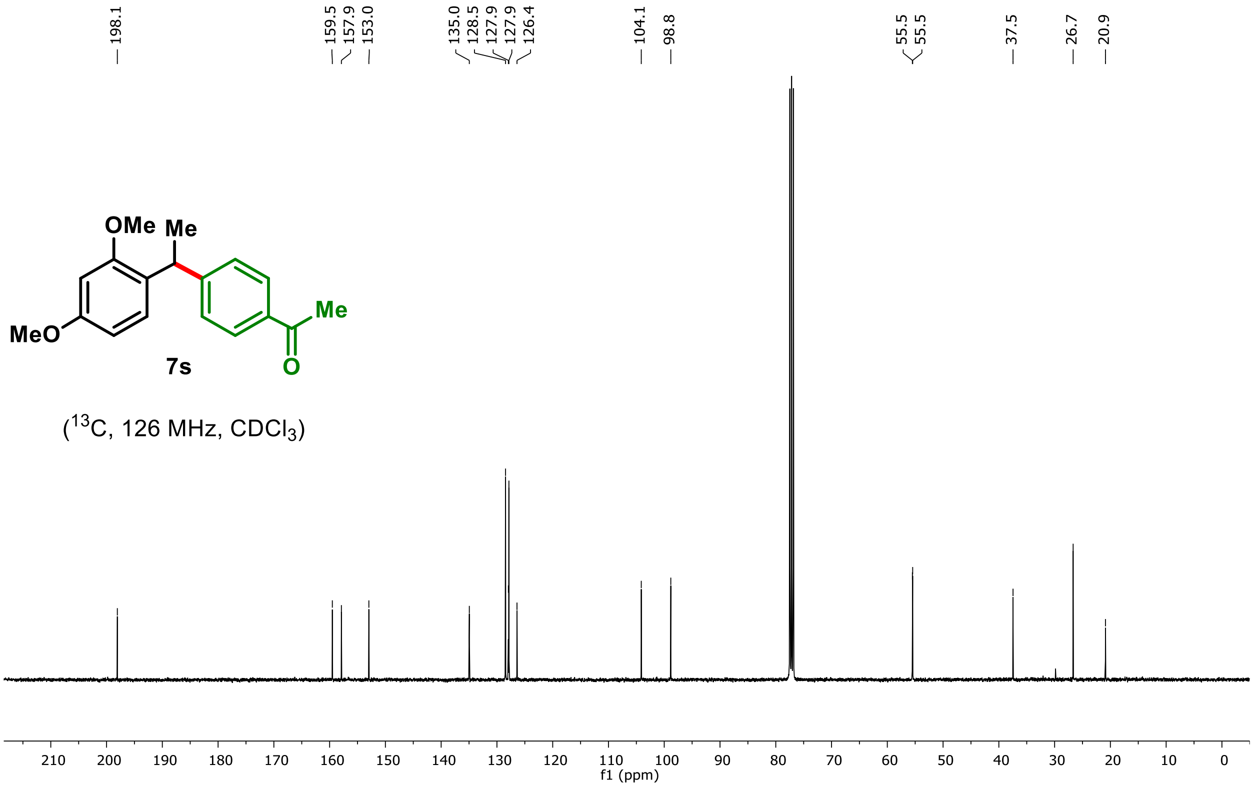

7s

(^{13}C, 126 MHz, $CDCl_3$)

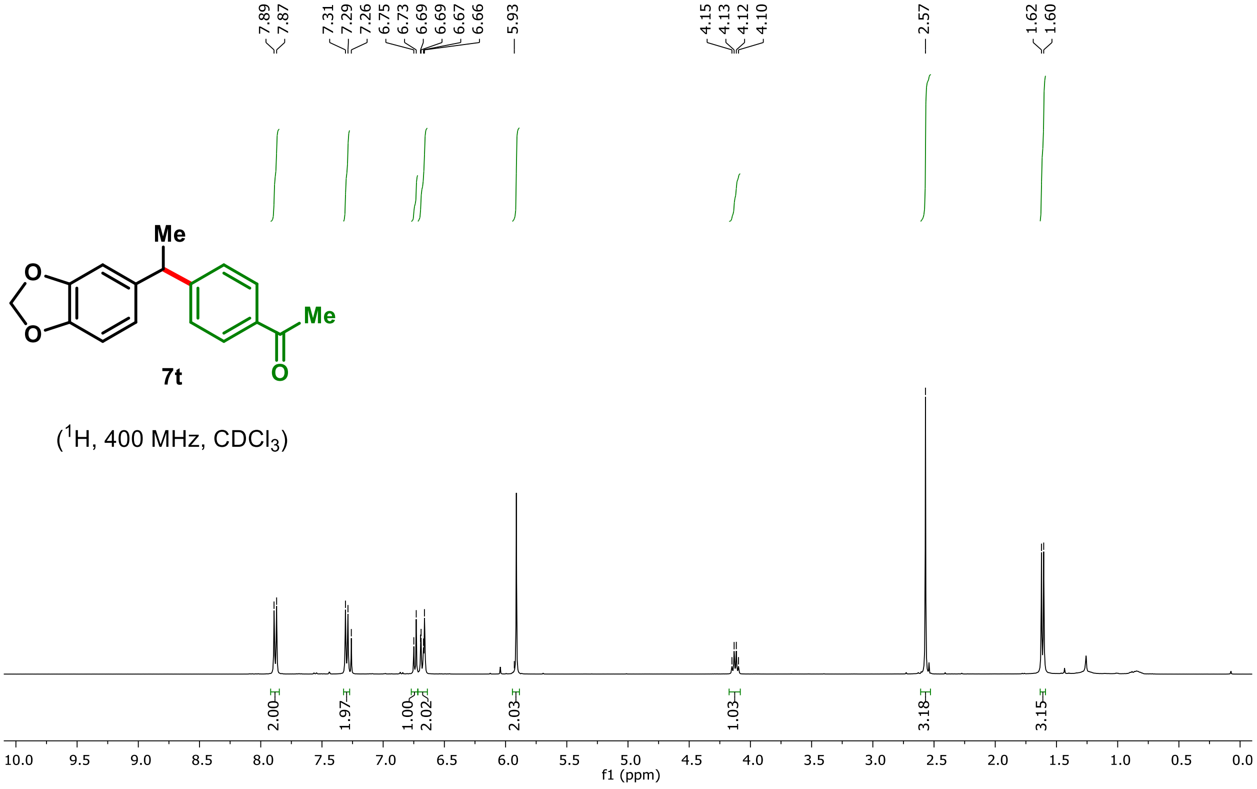

7t

(^{1}H, 400 MHz, $CDCl_3$)

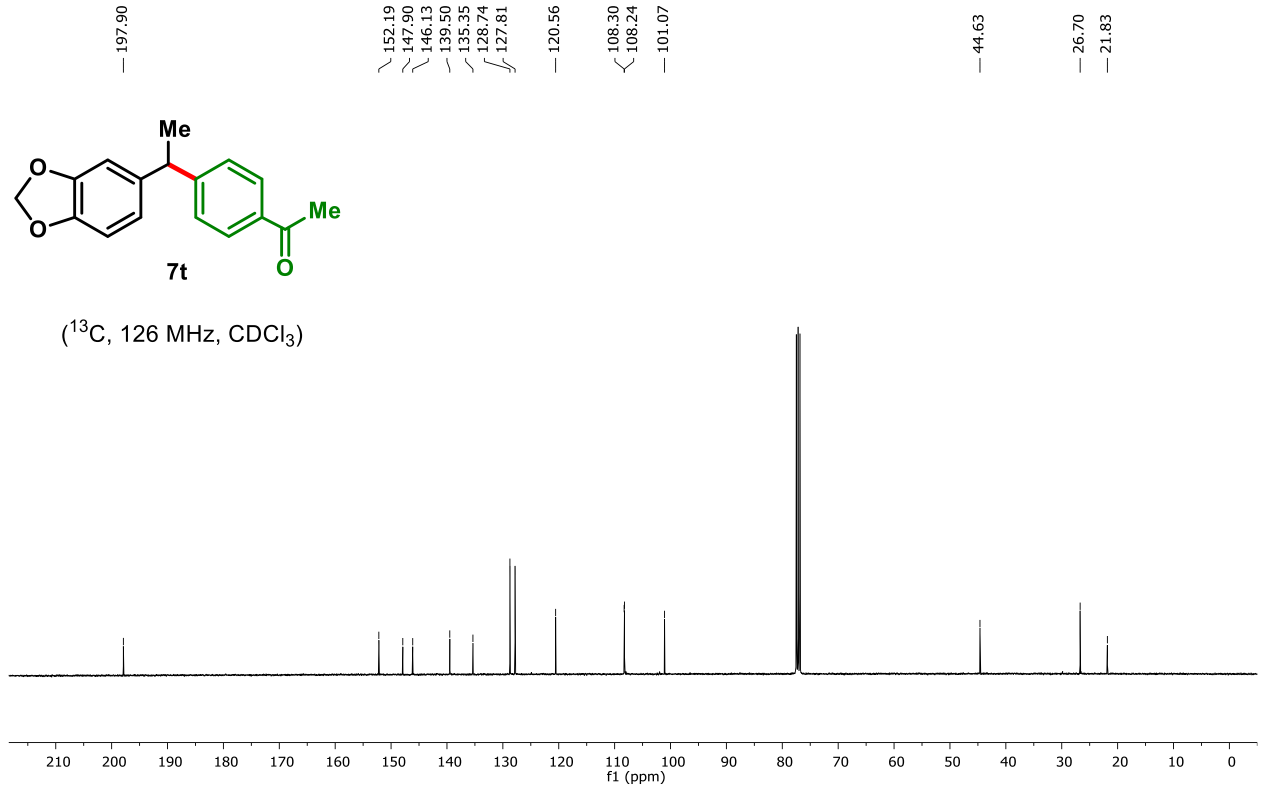
197.90
152.19
147.90
146.13
139.50
135.35
128.74
127.81
120.56
108.30
108.24
101.07
44.63
26.70
21.83
Me
O
O
Me
O
7t
(13C, 126 MHz, CDCl3)
f1 (ppm)

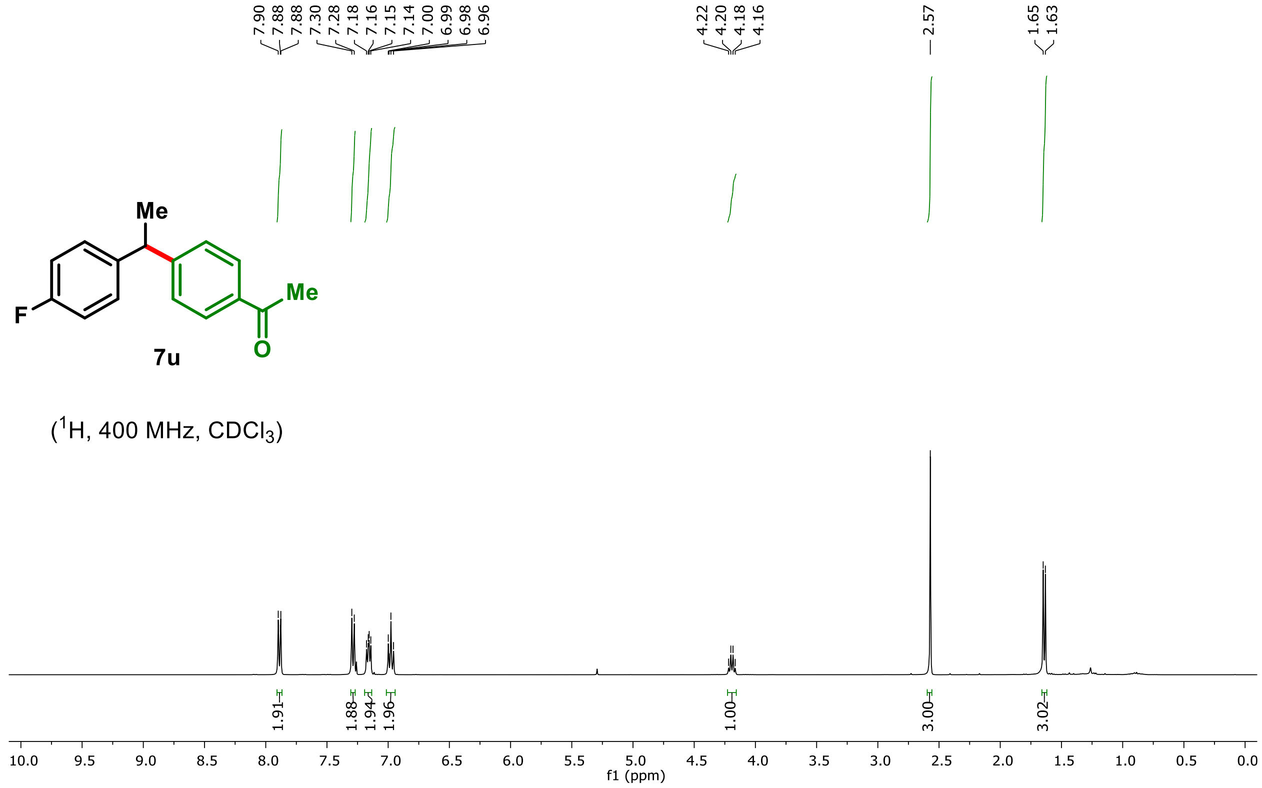
7.90
7.88
7.88
7.30
7.28
7.18
7.16
7.15
7.14
7.00
6.99
6.98
6.96
4.22
4.20
4.18
4.16
2.57
1.65
1.63
Me
F
Me
O
7u
(1H, 400 MHz, CDCl3)
1.91
1.88
1.94
1.96
1.00
3.00
3.02
f1 (ppm)

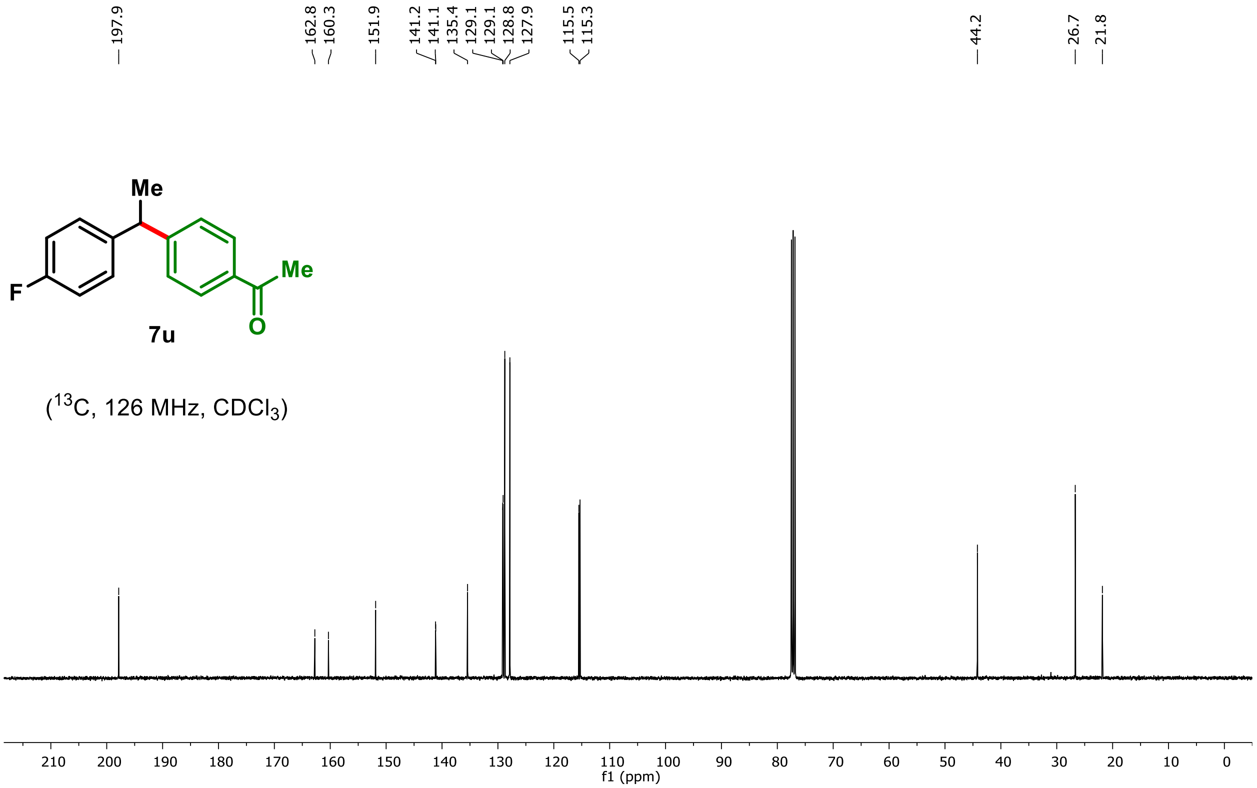
197.9
162.8
160.3
151.9
141.2
141.1
135.4
129.1
129.1
128.8
127.9
115.5
115.3
44.2
26.7
21.8
Me
Me
F
O
7u
(^{13}C, 126 MHz, CDCl$_3$)
210 200 190 180 170 160 150 140 130 120 110 100 90 80 70 60 50 40 30 20 10 0
f1 (ppm)

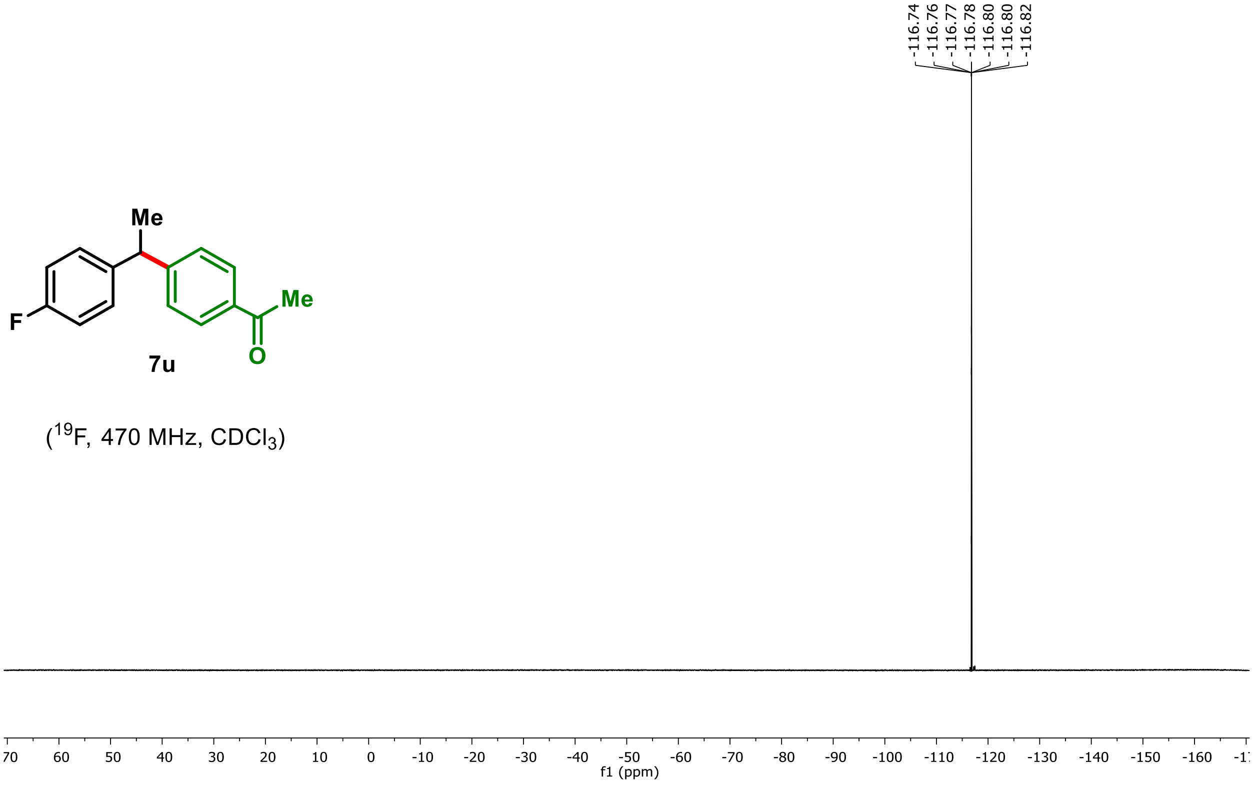
-116.74
-116.76
-116.77
-116.78
-116.80
-116.80
-116.82
Me
Me
F
O
7u
(^{19}F, 470 MHz, CDCl$_3$)
70 60 50 40 30 20 10 0 -10 -20 -30 -40 -50 -60 -70 -80 -90 -100 -110 -120 -130 -140 -150 -160
f1 (ppm)

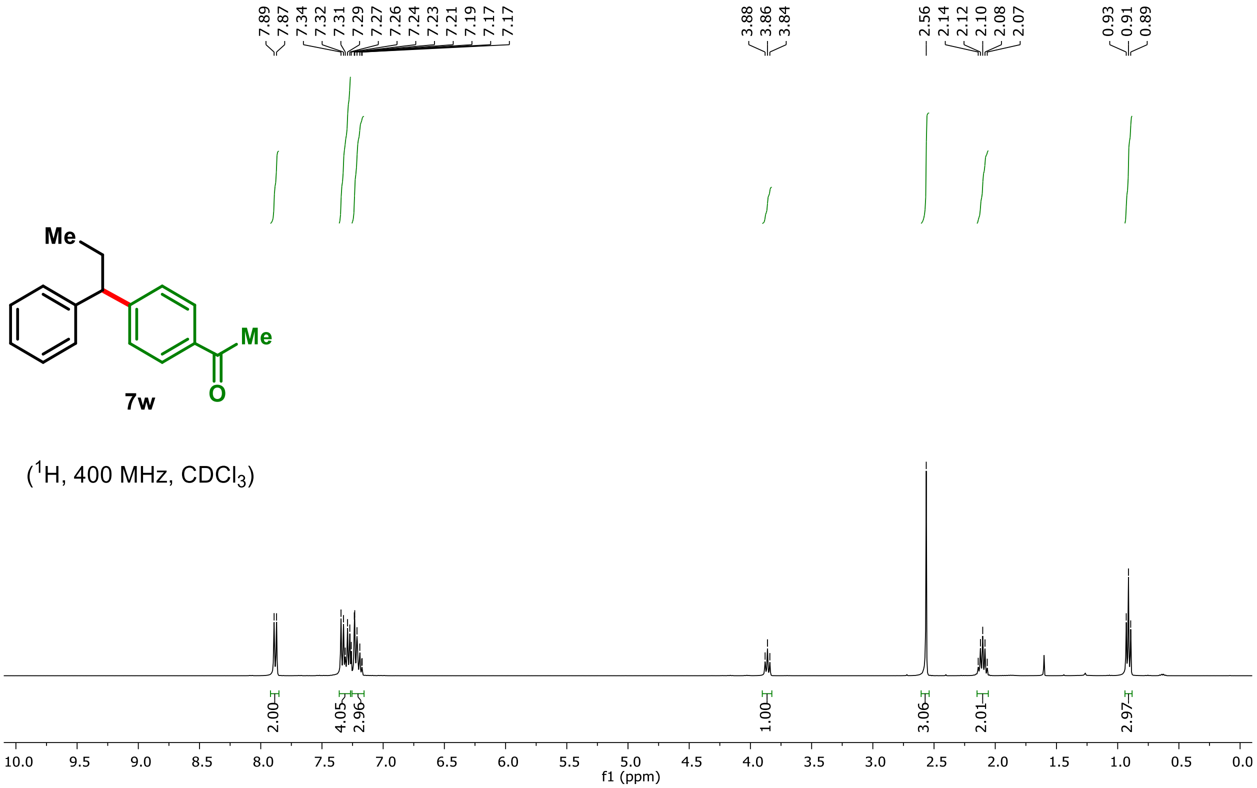
7.89 7.87 7.34 7.32 7.31 7.29 7.27 7.26 7.24 7.23 7.21 7.19 7.17 7.17
3.88 3.86 3.84
2.56 2.14 2.12 2.10 2.08 2.07
0.93 0.91 0.89
Me
Me
O
7w
(^{1}H, 400 MHz, $CDCl_3$)
2.00 4.05 2.96 1.00 3.06 2.01 2.97
f1 (ppm)

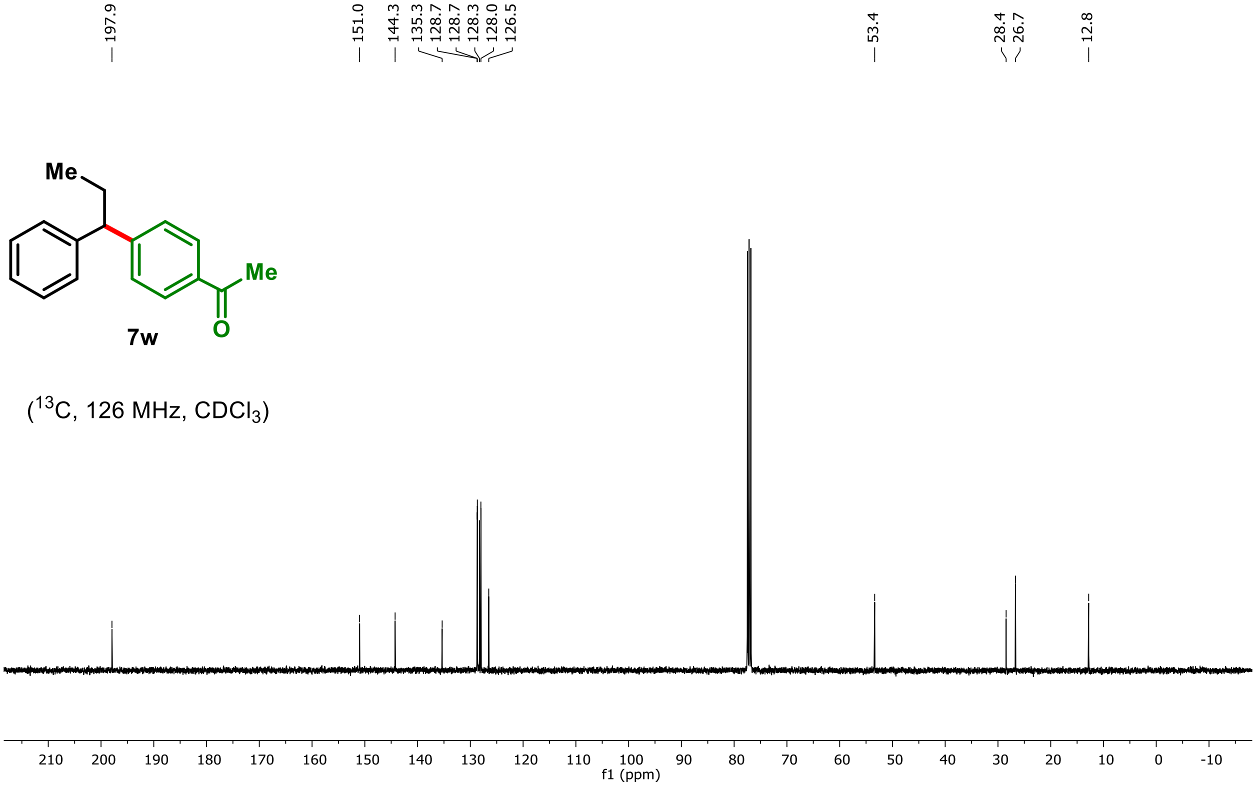
197.9
151.0 144.3 135.3 128.7 128.7 128.3 128.0 126.5
53.4
28.4 26.7
12.8
Me
Me
O
7w
(^{13}C, 126 MHz, $CDCl_3$)
f1 (ppm)

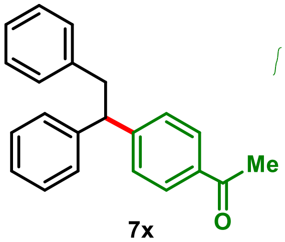

7x

(^{1}H, 400 MHz, $CDCl_3$)

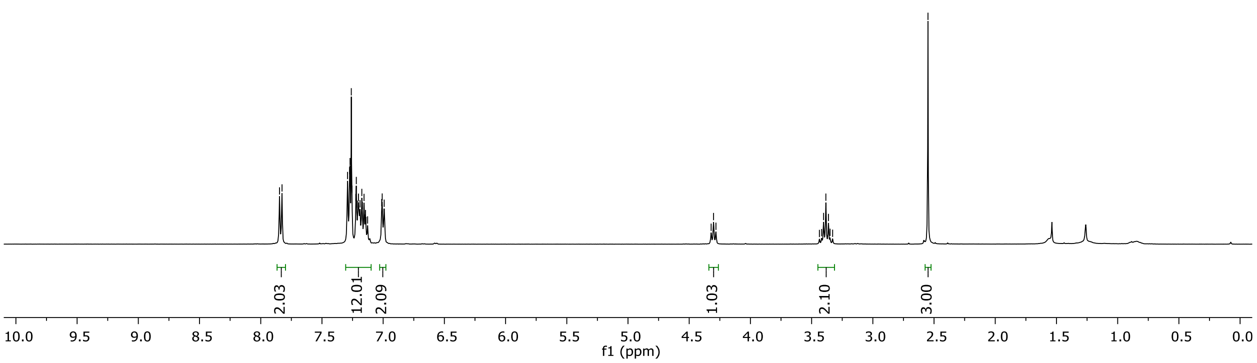

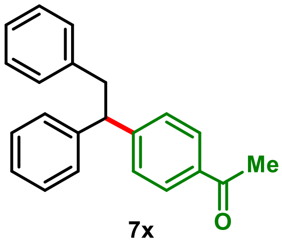

7x

(^{13}C, 126 MHz, $CDCl_3$)

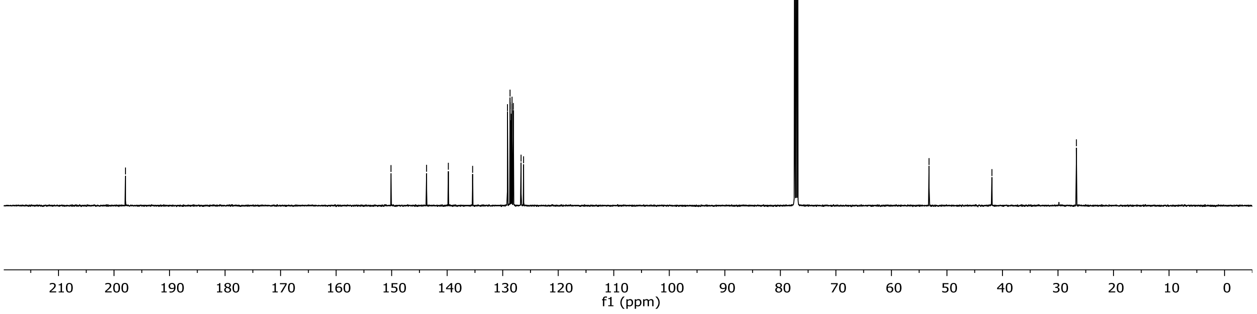

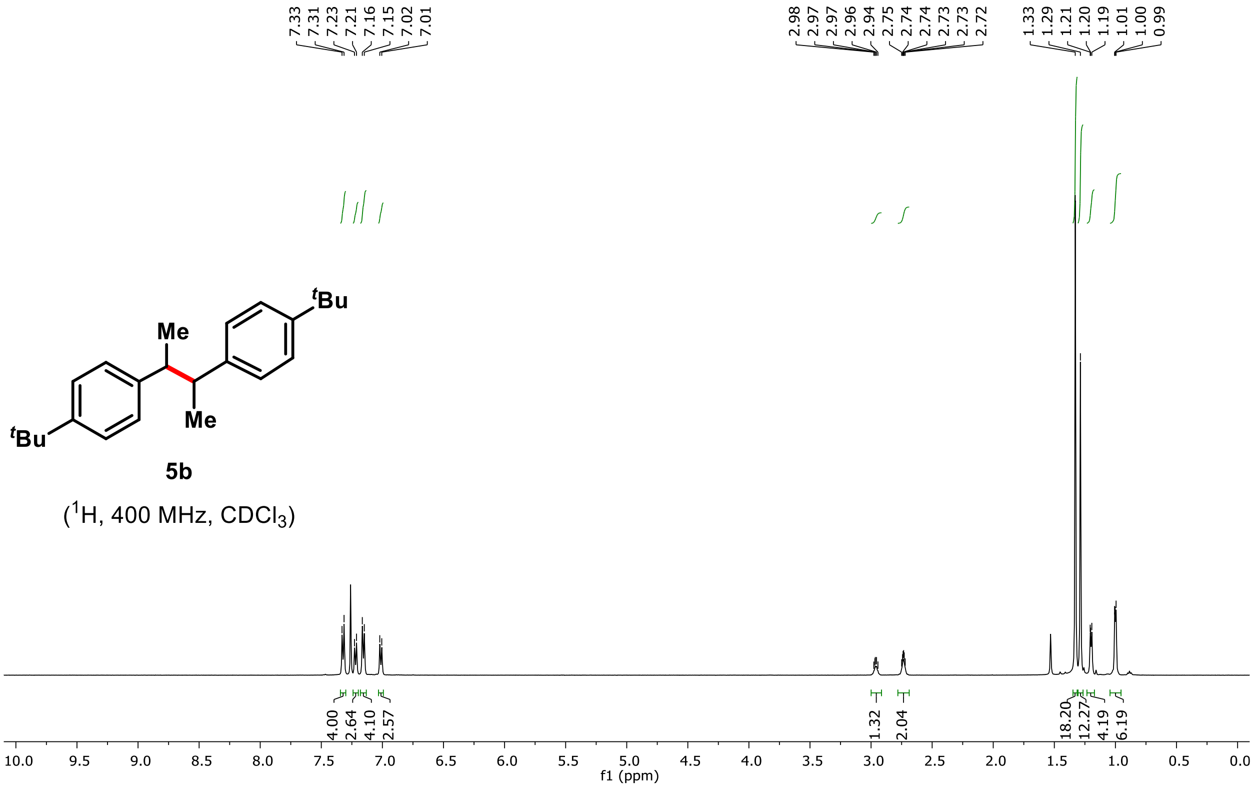

5b

(^{1}H, 400 MHz, $CDCl_3$)

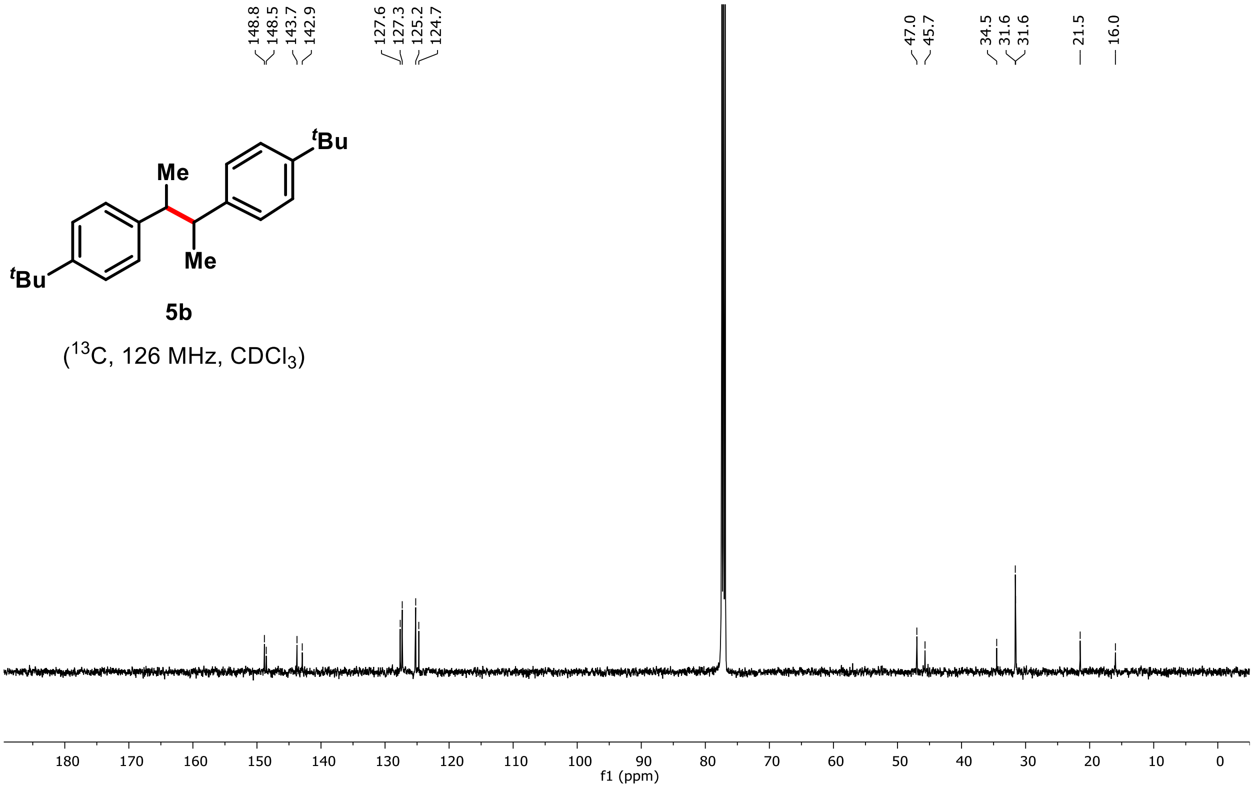

5b

(^{13}C, 126 MHz, $CDCl_3$)

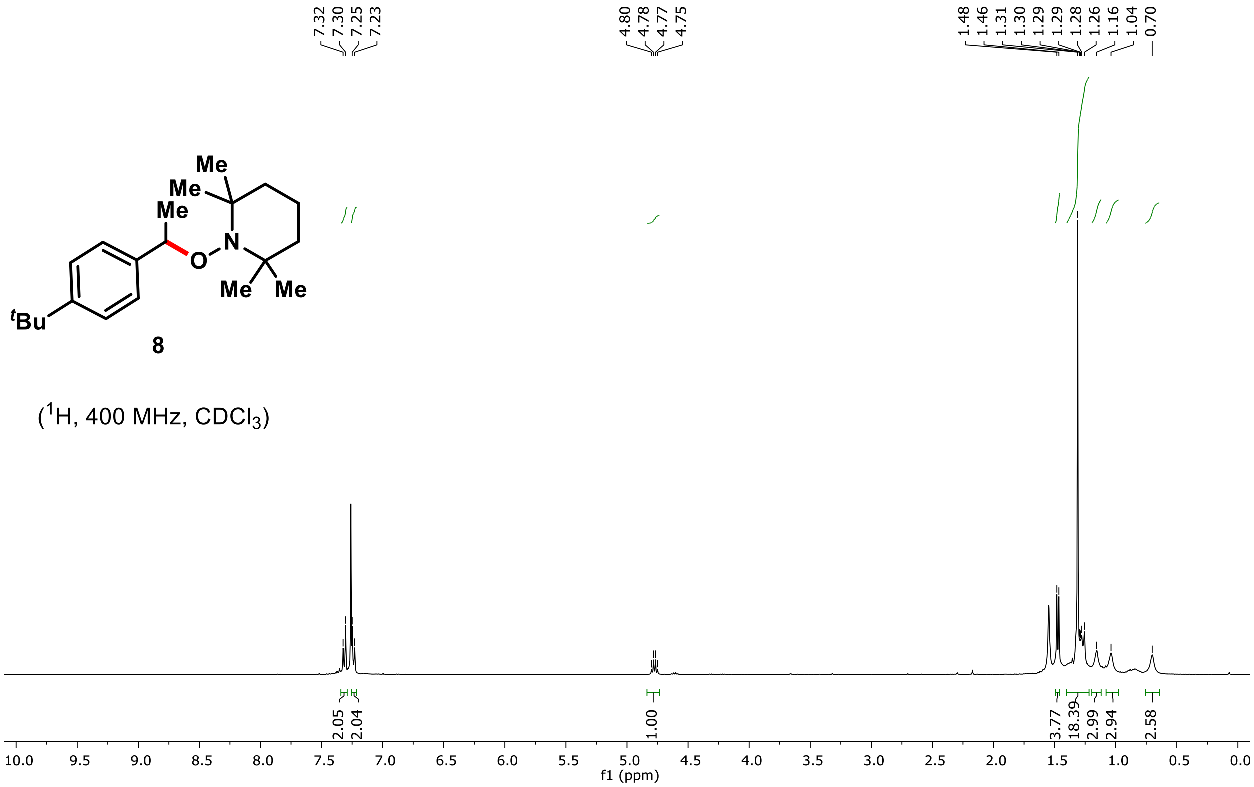
7.32
7.30
7.25
7.23
4.80
4.78
4.77
4.75
1.48
1.46
1.31
1.30
1.29
1.29
1.28
1.26
1.16
1.04
0.70
Me
Me
Me
O
N
Me
Me
tBu
8
(1H, 400 MHz, CDCl3)
2.05
2.04
1.00
3.77
18.39
2.99
2.94
2.58
f1 (ppm)

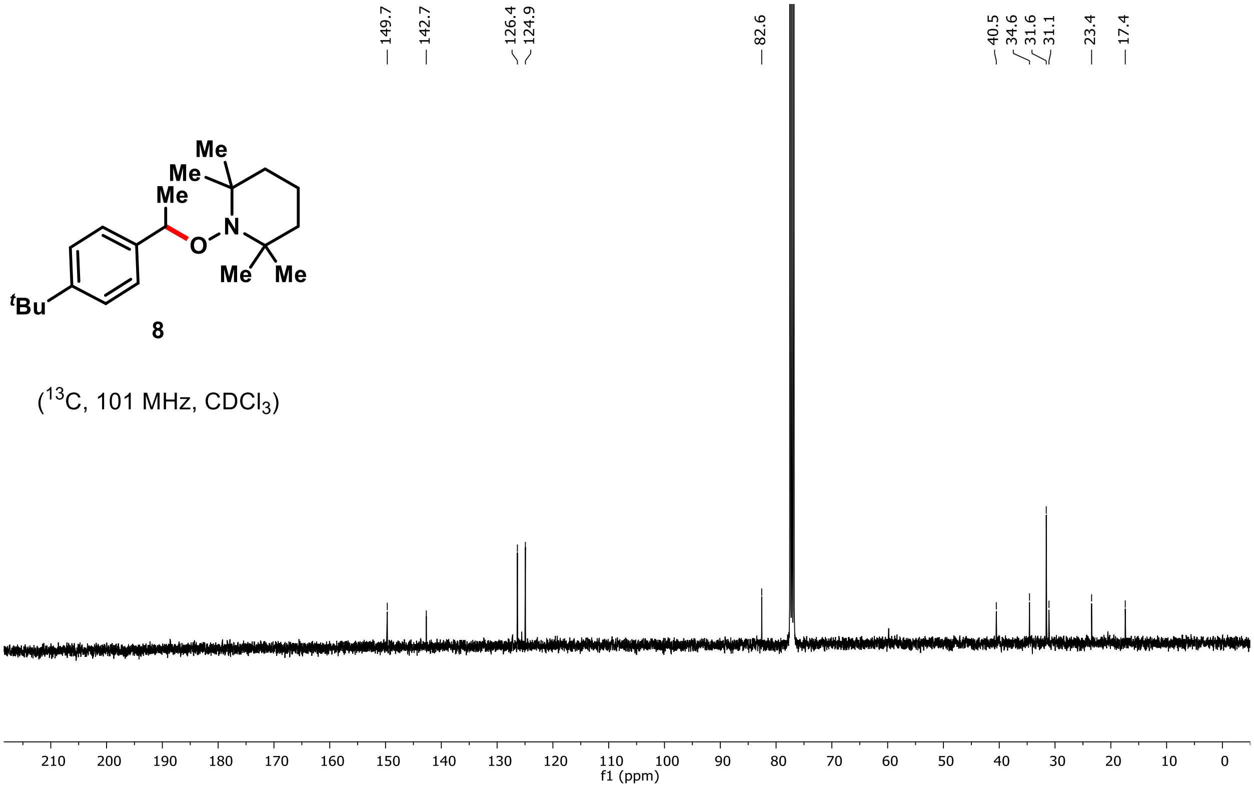
149.7
142.7
126.4
124.9
82.6
40.5
34.6
31.6
31.1
23.4
17.4
Me
Me
Me
O
N
Me
Me
tBu
8
(13C, 101 MHz, CDCl3)
f1 (ppm)

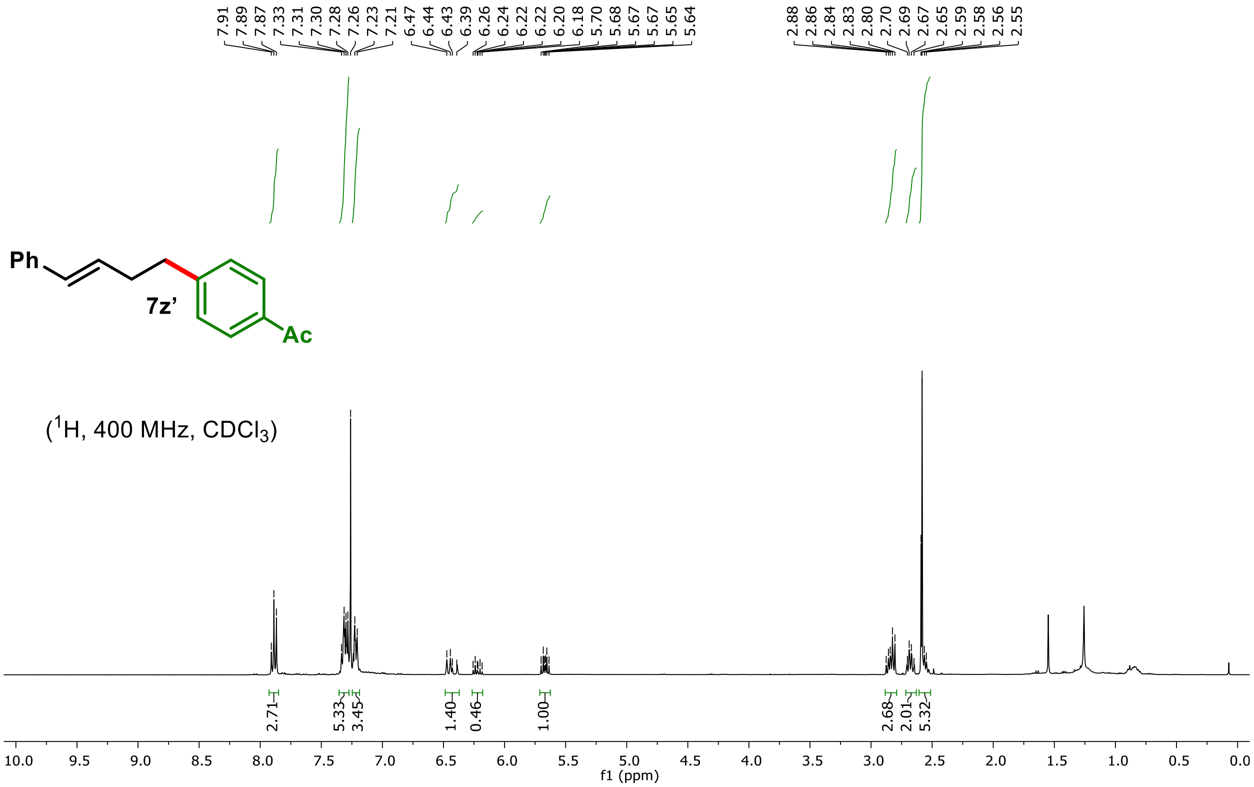
7.91
7.89
7.87
7.33
7.31
7.30
7.28
7.26
7.23
7.21
6.47
6.44
6.43
6.39
6.26
6.24
6.22
6.22
6.20
6.18
5.70
5.68
5.67
5.67
5.65
5.64
2.88
2.86
2.84
2.83
2.80
2.70
2.69
2.67
2.65
2.59
2.58
2.56
2.55
Ph
7z'
Ac
(1H, 400 MHz, CDCl3)
2.71
5.33
3.45
1.40
0.46
1.00
2.68
2.01
5.32
f1 (ppm)

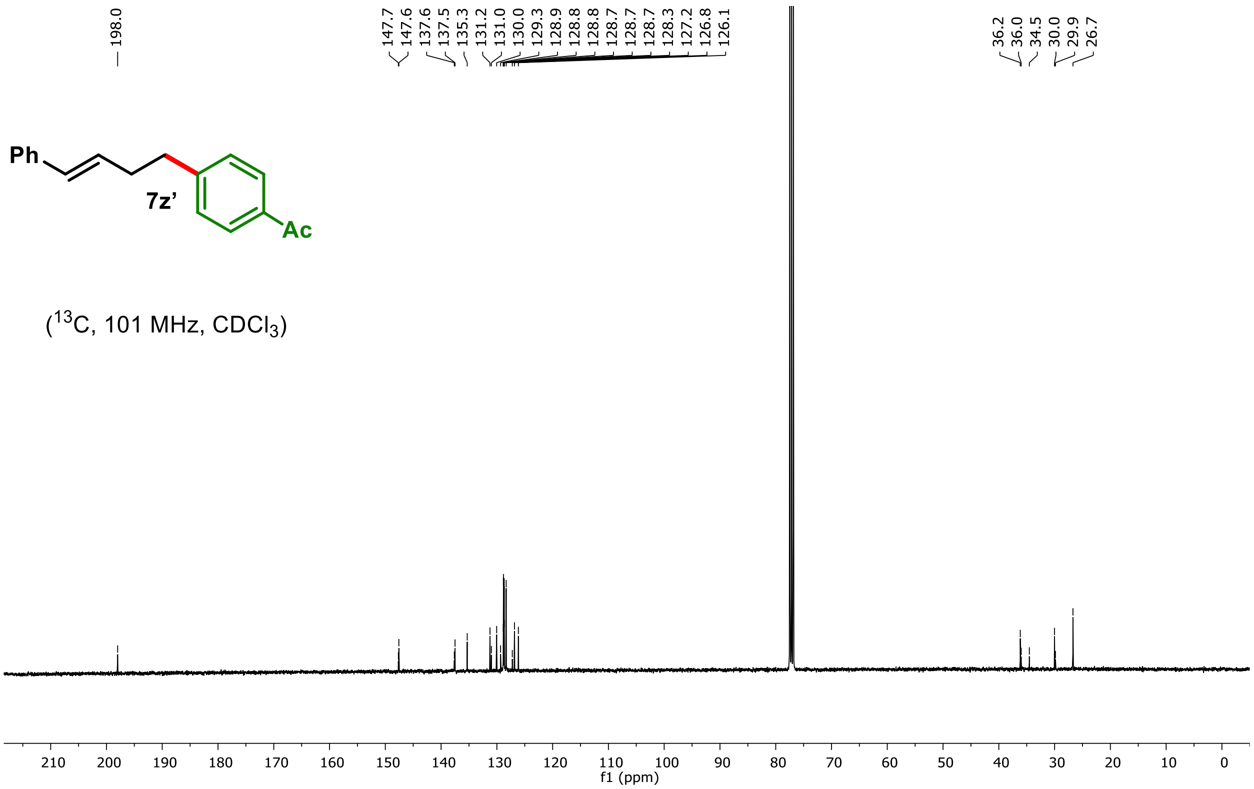
198.0
147.7
147.6
137.6
137.5
135.3
131.2
131.0
130.0
129.3
128.9
128.8
128.8
128.7
128.7
128.7
128.3
127.2
126.8
126.1
36.2
36.0
34.5
30.0
29.9
26.7
Ph
7z'
Ac
(13C, 101 MHz, CDCl3)
f1 (ppm)

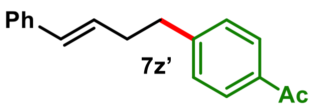

7z'

(COSY, 400 MHz, $CDCl_3$)

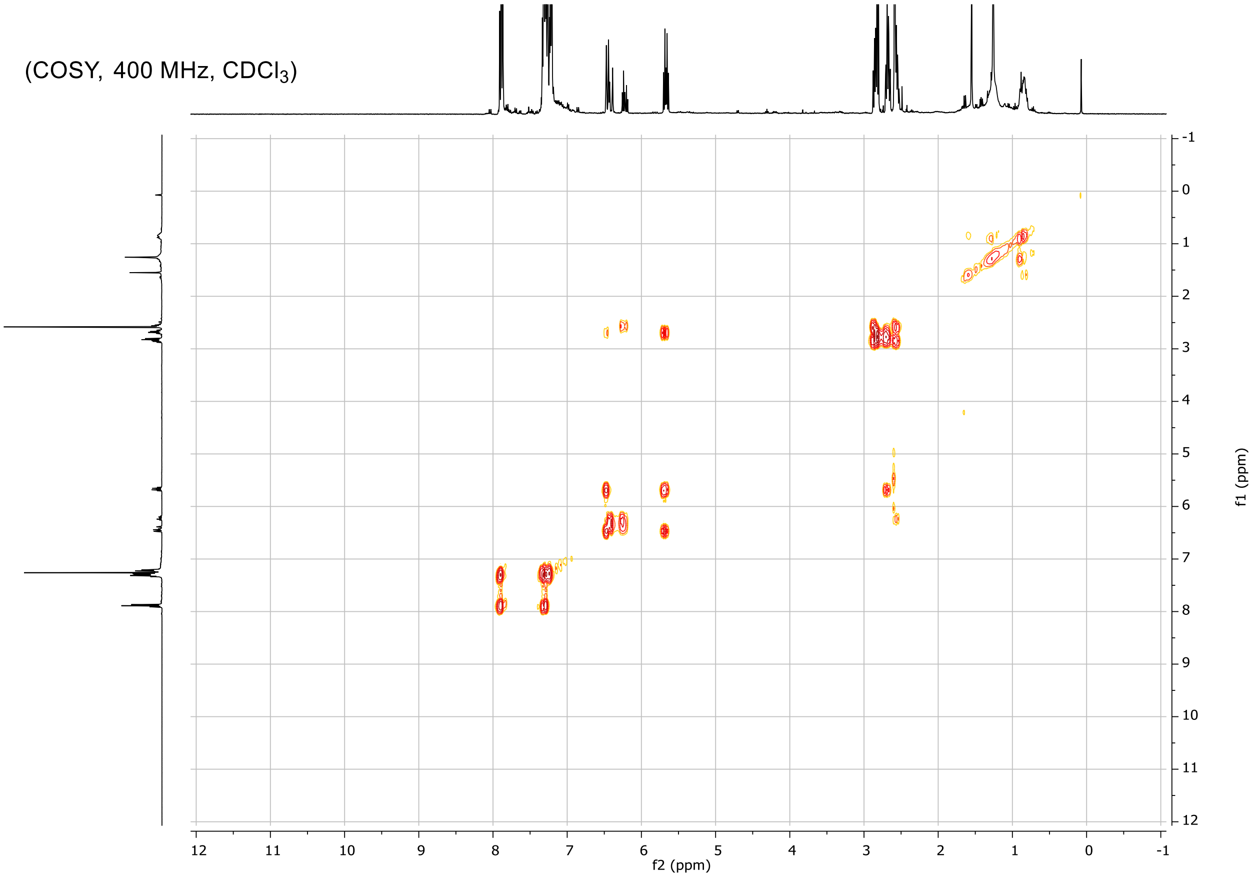

Supplement: Supplementary file 1 — cs3c01981_si_001.pdf [file cs3c01981_si_001.pdf]
